# Going beyond Binary: Rapid Identification of Protein–Protein Interaction Modulators Using a Multifragment Kinetic Target-Guided Synthesis Approach

**DOI:** 10.1021/acs.jmedchem.3c00108

**Published:** 2023-03-31

**Authors:** Katya Nacheva, Sameer S. Kulkarni, Mintesinot Kassu, David Flanigan, Andrii Monastyrskyi, Iredia D. Iyamu, Kenichiro Doi, Megan Barber, Niranjan Namelikonda, Jeremiah D. Tipton, Prakash Parvatkar, Hong-Gang Wang, Roman Manetsch

**Affiliations:** † Department of Chemistry, University of South Florida, Tampa, Florida 33620, United States; ‡ Department of Chemistry and Chemical Biology, Northeastern University, Boston, Massachusetts 02115, United States; § Department of Sciences, Hillsborough Community College, Tampa, Florida 33619, United States; ∥ Department of Pediatrics, Division of Pediatric Hematology and Oncology, Penn State College of Medicine, Hershey, Pennsylvania 17033, United States; ⊥ Proteomics and Mass Spectrometry Core Facility, University of South Florida, Tampa, Florida 33620, United States; # Department of Pharmaceutical Sciences, Northeastern University, Boston, Massachusetts 02115, United States; @ Center for Drug Discovery, Northeastern University, Boston, Massachusetts 02115, United States; ∇ Barnett Institute of Chemical and Biological Analysis, Northeastern University, Boston, Massachusetts 02115, United States

## Abstract

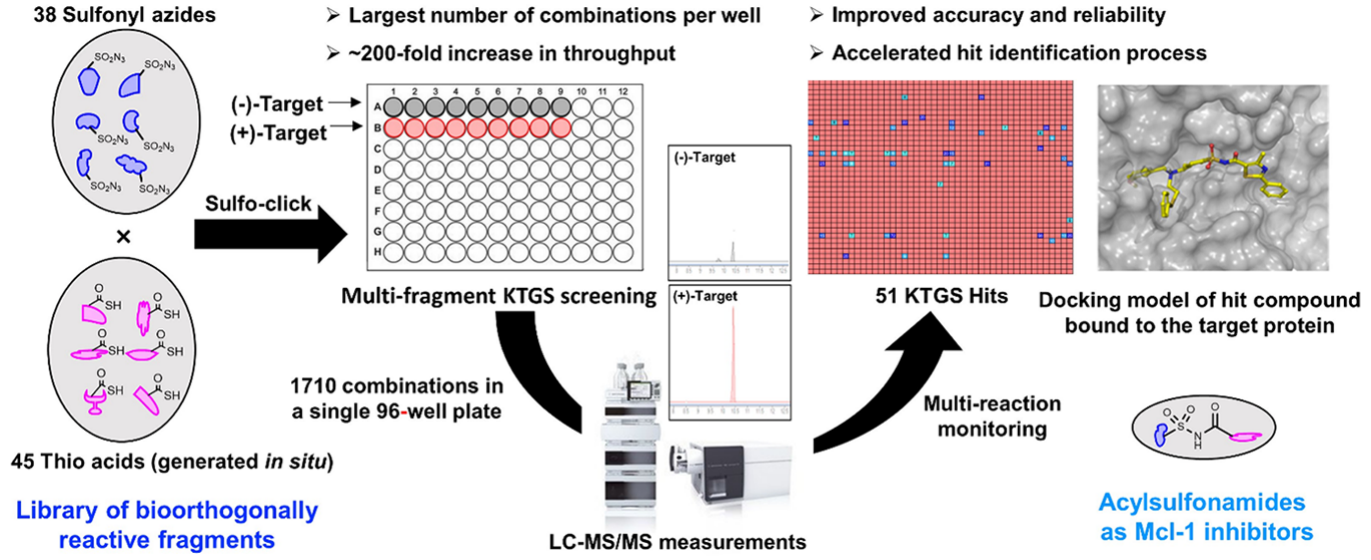

Kinetic target-guided synthesis (KTGS) is a powerful screening approach that enables identification of small molecule modulators for biomolecules. While many KTGS variants have emerged, a majority of the examples suffer from limited throughput and a poor signal/noise ratio, hampering reliable hit detection. Herein, we present our optimized multifragment KTGS screening strategy that tackles these limitations. This approach utilizes selected reaction monitoring liquid chromatography tandem mass spectrometry for hit detection, enabling the incubation of 190 fragment combinations per screening well. Consequentially, our fragment library was expanded from 81 possible combinations to 1710, representing the largest KTGS screening library assembled to date. The expanded library was screened against Mcl-1, leading to the discovery of 24 inhibitors. This work unveils the true potential of KTGS with respect to the rapid and reliable identification of hits, further highlighting its utility as a complement to the existing repertoire of screening methods used in drug discovery.

## Introduction

Protein–protein interactions (PPIs) are essential for the regulation of numerous cellular functions linked to signal transduction, gene transcription, initiating programmed cell death (apoptosis), and others.^1−3^ The viability of living cells is directly dependent on the precise execution of these pathways, whereby pathway abnormalities may lead to various diseases such as autoimmune disorders, Alzheimer’s disease, or cancer.^4^ In the past two decades, small molecules have been recognized for their ability to modulate or perturb a specific PPI, therefore possessing great therapeutic potential.^1,5,6^ PPIs are generally weak, are dynamic in nature, and involve large, planar, binding sites that are distinct from enzymatic targets that are typically smaller and concave in shape.^7^ On the contrary, fragment-based drug design (FBDD) strategies have increasingly demonstrated remarkable utility in drug discovery efforts, offering higher hit rates of fragments (low-molecular weight compounds, typically with MW < 250 Da) compared to traditional high-throughput screening that handles larger molecules (MW > 250 Da). Importantly, fragments occupying specific subpockets within the binding site can be linked to derive high-affinity inhibitors, allowing greater flexibility in terms of exploring a wider chemical space. This is evidenced by the number of drug candidates derived from FBDD entering clinical trials (40 compounds) wherein two candidates have been approved.^8^ However, despite great advances in FBDD for the identification of ligand-efficient binders modulating or perturbing specific PPIs, fragment evolution via conventional optimization approaches is still not straightforward and requires extensive synthetic efforts.^7^

Among all fragment identification and evolution approaches, kinetic target-guided synthesis (KTGS) aims to accelerate the identification of medium- to high-affinity ligands by combining screening and synthesis of libraries of low-molecular weight compounds in one step. In KTGS, the biological target templates the irreversible assembly of its own bidentate ligand from a library of bioorthogonally reactive building blocks. Unlike other FBDD strategies, KTGS merges fragment identification and fragment evolution processes into a single step, thereby avoiding time- and resource-consuming syntheses of compounds that may not possess biological activity in follow-up confirmatory studies.

Thus far, KTGS has mainly been utilized to identify enzyme inhibitors. Such targets include acetylcholinesterase, carbonic anhydrase, HIV-1 protease, and other enzymes.^9^ Although limited, KTGS reports on non-enzymatic targets have also been described, including those that involve targeting biomolecules such as DNA and RNA.^10^ The ligation chemistry utilized in the vast majority of these reports is the Huisgen cycloaddition reaction between alkynes and azides to form 1,2,3-triazoles. Nevertheless, ligation chemistries such as amidation of activated esters, thia-Michael addition, and nucleophilic ring opening of epoxides have been implemented, as well. More recently, the three-component Mannich ligation and the four-component Ugi reaction were also successfully used to identify inhibitors of the STAT5 transcription factor and endothiapepsin, respectively.^10h,11^ To the best of our knowledge, we are the first to report a KTGS variant that can be extended to identify PPIMs. In proof-of-concept studies based on previous reports by Abbott Laboratories,^12−14^ Manetsch and co-workers demonstrated for the first time that the sulfo-click reaction, an amidation reaction between thio acids and sulfonyl azides, is suitable for a KTGS approach targeting Bcl-X_L_, one of the antiapoptotic members of the Bcl-2 family.^15^ A set of nine thio acids and nine sulfonyl azides were incubated for 6 h at 37 °C as binary mixtures in the presence and absence of Bcl-X_L_. The resulting incubation samples were analyzed for acylsulfonamide product formation by liquid chromatography combined with mass spectrometry using the selected ion mode (LC-MS-SIM). A comparison of the traces led to the discovery of three new hit combinations (**SZ7TA2**, **SZ9TA1**, and **SZ9TA5**), in addition to **SZ4TA2**, which was previously reported by Abbott Laboratories.^14^ The four KTGS hits were the most potent compounds tested, disrupting the Bcl-X_L_–BH3 interaction by ≥60% at a compound concentration of 50 μM, while randomly selected acylsulfonamides showed an activity of ≤15%. Several other successful KTGS reports involving targets that participate in PPIs have since been reported.^10c,10e−10i^

While the KTGS approach has led to promising results, the method usually suffers from limited throughput due to the binary nature of the screening platform. As a result, performing protein-templated incubations with more than two bioorthogonally reactive fragments in a single well has been of great interest for improving the efficiency of KTGS further to access a larger chemical space and holds potential to dramatically increase the throughput of the KTGS screening platform. Although several reports underscore the feasibility of conducting multiple protein-templated reactions in one well,^9b,9d,9f,9g,9k,9p−9s,10b,10g,10h,15a^ identification of hit combinations, especially in incubation samples containing a large number of reactive building blocks, directly correlates to the LC-MS sensitivity for quantifying the protein templation effect. Specifically, the need to accurately determine the kinetics of the incubations with and without the protein target sets the analytical bar high especially for the protein-free incubation.^15a^ As all possible protein-templated products from a large library of reactive building blocks vary in terms of structure, they also vary in terms of ionization. Therefore, in comparison to binary mixtures, multifragment KTGS screening is far more challenging, as the simultaneous determination of multiple kinetics of the incubations with and without protein is an absolute requirement. During preliminary studies, we demonstrated that co-incubation of one thio acid with six sulfonyl azides permitted successful detection of the known Bcl-X_L_ hit combination **SZ4TA2** using the LC-MS-SIM screening approach.^15a^ However, the attempt to perform the experiment with nine building blocks incubated at the same time (three thio acids and six sulfonyl azides) failed to provide definitive results. Nevertheless, we hypothesized that the obstacles imparted by instrumental limitations could be overcome by the use of a more advanced mass spectrometry technology. Triple quadrupole mass spectrometry (TQMS) offers a better signal/noise ratio (S/N) than single quadrupole instruments, resulting in a significantly lower limit of detection and quantification, which is particularly beneficial for the detection of products in the absence of a protein target. As opposed to LC-MS-SIM, sample analysis by TQMS simultaneously monitors one specific precursor ion and one of its specific fragmentation product ions [selected reaction monitoring (SRM)] or one specific precursor ion and multiple fragmentation product ions [multiple reaction monitoring (MRM)]. Furthermore, the SRM and MRM modes can easily distinguish between precursor ions with the same *m*/*z* ratios as they generate different, specific fragmentation product ions. Conceivably, employing the sulfo-click multifragment screening approach, coupled with TQMS, presents an exciting opportunity to immensely improve the throughput and take the KTGS approach to the next level of efficiency. To the best of our knowledge, the MRM LC-MS/MS method has been used with fragmentation only twice in reported KTGS experiments: one impressive cell-based KTGS example against bovine carbonic anhydrase II (bCAII)^9t^ and another attempted KTGS example against urokinase plasminogen activator (uPA).^11^ However, inherent limitations are evident in both examples. While Antti and Sellstedt successfully demonstrated that cell-based KTGS is feasible, the bCAII proof-of-concept study employed only one pair of fragments in the template-mediated approach, severely limiting the throughput. On the contrary, Veken’s efforts to employ KTGS utilizing a multicomponent Groebke–Blackburn–Bienaymé ligation reaction coupled with MRM LC-MS/MS to target uPA failed to occur with the fragments and conditions utilized. Interestingly, Deprez-Poulain and co-workers utilized MRM LC-MS/MS detection in a recent KTGS screen against ERAP2 without taking advantage of the fragmentation feature.^9w^

Herein, we present our findings in terms of optimization of the parallel screening process of multiple PPIs via sulfo-click KTGS utilizing TQMS. Furthermore, we report results obtained from the optimized multifragment screening of an expanded library of 1710 possible acylsulfonamide combinations against Mcl-1, a member of the Bcl-2 protein family. This work represents a remarkable 21-fold increase in the number of screened combinations from our previous report against Bcl-X_L_ (81 combinations^15b^) and the largest number of combinations screened in a multifragment format using the KTGS approach against any target to date. Moreover, this approach also led to the identification of multiple low-micromolar affinity acylsulfonamides as Mcl-1 inhibitors, highlighting the power and utility of this highly efficient screening platform.

## Results and Discussion

### Optimization of Multifragment KTGS Screening against Bcl-X_L_

Inspired by the successfully implemented multifragment screening for the identification of a known Bcl-X_L_ inhibitor, we aimed to optimize the throughput of the sulfo-click KTGS reaction by employing a multifragment screening technique with a TQMS instrument. Using this technique, we intended to probe a larger chemical space by expanding the existing library of 81 fragment combinations and to seek new PPIMs against Mcl-1 by applying the optimized experimental conditions to a newly developed library of reactive fragments.

Using SRM or MRM requires knowledge of not only the *m*/*z* ratio of the precursor ion but also the corresponding fragmentation product ion or ions generated at particular collision energies (electronvolts). To correlate the magnitude of collision energies to specific fragmentation pathways, a direct infusion experiment was performed in which a methanolic solution of an acylsulfonamide was injected into the collision cell, providing information about the fragmentation patterns at various collision energies. This experiment was performed with multiple acylsulfonamides (**SZ1TA3**, **SZ2TA2**, **SZ7TA2**, **SZ2TA4**, **SZ9TA7**, **SZ7TA7**, **SZ6TA7**, **SZ9TA1**, and **SZ8TA8**) and consistently demonstrated that the acylsulfonamide bond was the primary fragmentation site leading to the corresponding acylium ion (see Figure S1). In addition, the acylium ion was the most abundant species among all other fragmentation product ions. The reproducibility of this phenomenon allows one to reliably predict the resulting product ions from any acylsulfonamide by simply analyzing the structure. This, in turn, abolishes the necessity of running MS/MS optimization screens to predict fragmentation patterns, thereby saving the researcher a significant amount of time when analyzing large libraries. Therefore, the fragmentation pathway leading to the acylium ion presented an advantageous pattern that could be used to establish a practical method of setting up and analyzing multifragment incubations with TQMS using multiple SRMs. The feasibility of KTGS using multiple SRMs with a TQMS instrument was investigated for the screening of the previously reported library of nine sulfonyl azides (**SZ1**–**SZ9**) and nine thio acids (**TA1**–**TA9**) containing four known Bcl-X_L_ inhibitory compounds.^15b^ For the purpose of optimization, the entire fragment library was arranged in various screening batches that differ in terms of the theoretical number of possible acylsulfonamide products but contain at least one of the previously reported KTGS hit acylsulfonamides. Starting with nine fragment combinations in one well, we quickly progressed to 81 fragment combinations per well (see Table S1) without sacrificing the quality of the LC-MS/MS traces or the detection of previously identified KTGS hit acylsulfonamides. The KTGS incubations were set up following our most recent protocol involving fluorenyl-methyl thioesters (**TE**s) that are readily converted *in situ* to the corresponding thio acids (**TA**s) immediately prior to the screening.^15c^ Each of the previously reported KTGS hit combinations was redetected in the multifragment screening and further validated by comparing its peak retention time with that of the corresponding synthetic sample.

**Figure 1:**
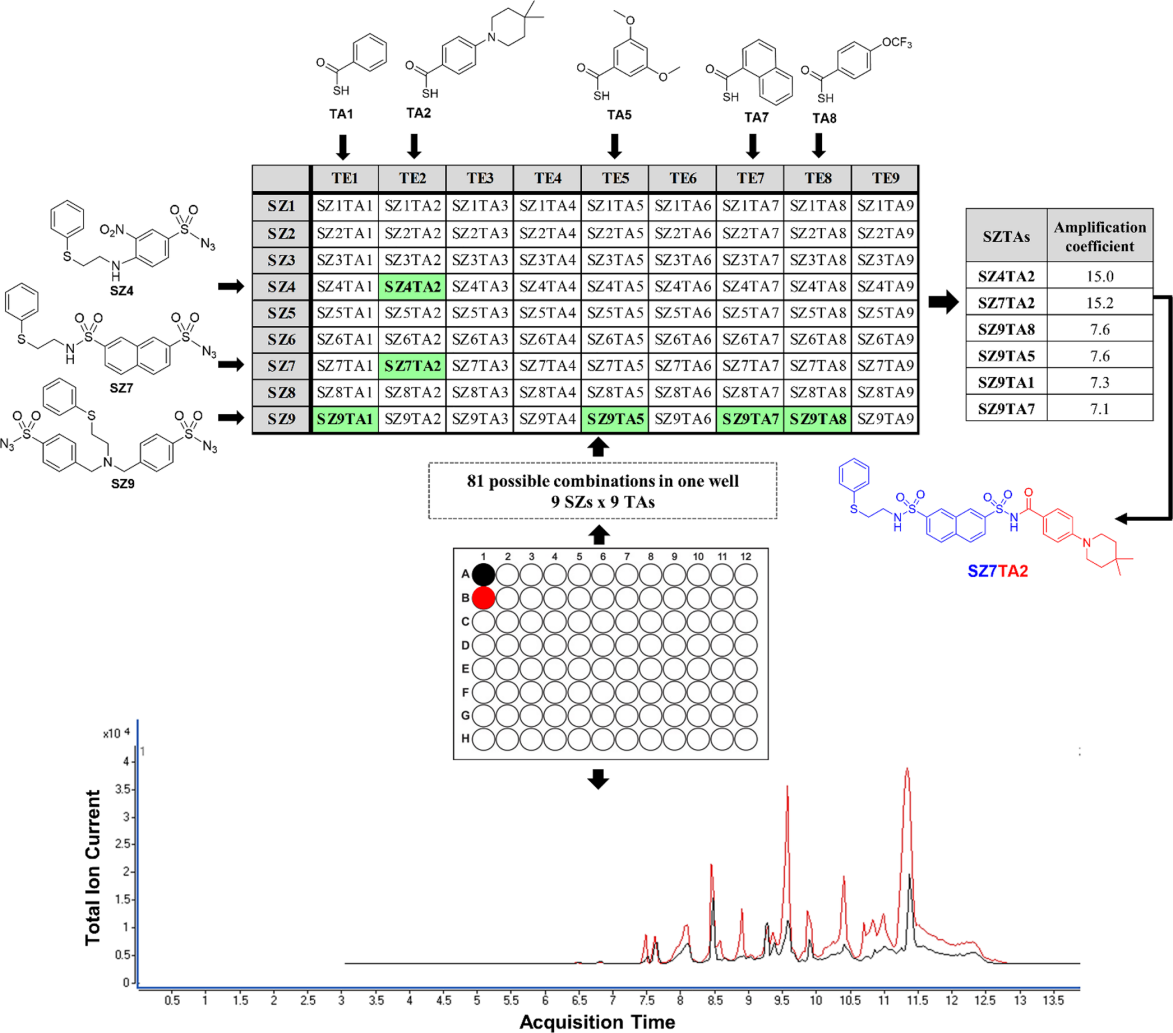
Experimental setup and LC-MS/MS analysis of a multifragment kinetic target-guided synthesis (KTGS) incubation with a library of nine sulfonyl azides (**SZ1**–**SZ9**) and nine thio acids (**TA1**–**TA9**) against Bcl-X_L_. All thio acids were generated from the corresponding fluorenyl-methyl thioester just prior to screening. The compounds colored green represent KTGS hit acylsulfonamides **SZ4TA2**, **SZ7TA2**, **SZ9TA1**, **SZ9TA5**, **SZ9TA7**, and **SZ9TA8**. Hit acylsulfonamides **SZ4TA2**, **SZ7TA2**, **SZ9TA1**, and **SZ9TA5** were previously identified in a KTGS approach using LC-MS-SIM analysis of binary mixtures of reactive fragments,^15b^ whereas compounds **SZ9TA7** and **SZ9TA8** were additional hit compounds identified in the newly developed multifragment KTGS approach with TQMS analysis. The LC-MS/MS chromatograms are shown as the total ion current (TIC) of one of the incubation samples without Bcl-X_L_ (black) and one with Bcl-X_L_ (red).

Of these studies, the most impressive results were obtained with incubations containing the entire library of reactive fragments leading to 81 possible sulfonyl azides in one well. The incubation conditions resembled to those established in the binary reactions [2 μM Bcl-X_L_ in phosphate buffer (pH 7.4), 20 μM sulfonyl azides, 20 μM thio acids, and 37 °C].^15b^ With the exception of the use of **TE**s rather than **TA**s, we determined by exploratory experimentation that it is best to compensate for the significantly higher fragment numbers by increasing the protein concentration from 2 to 5 μM and prolonging the incubation time from 6 to 8 h. Importantly, with TQMS using multiple SRMs, simultaneous monitoring of all 81 potential acylsulfonamides was conducted in a single KTGS incubation well containing the protein target Bcl-X_L_ (Figure 1).

With the multifragment KTGS approach, the number of incubation samples to be analyzed by LC-MS was drastically reduced from 162 using LC/MS-SIM in the binary fragment setup (81 incubation mixtures with one **TA** and one **SZ** in the presence of Bcl-X_L_ and 81 incubation mixtures with one **TA** and one **SZ** in the absence of Bcl-X_L_) to merely two samples using LC-MS/MS (one incubation mixture with all reactive fragments in the presence of Bcl-X_L_ and one incubation mixture with all reactive fragments in the absence of Bcl-X_L_). In parallel, a second well was set up in the same manner but without the protein target as a control incubation. Extraordinarily, the experimental time, the amount of protein required, and the amounts of reactive fragments were all significantly reduced, streamlining the overall process.

The LC-MS/MS traces were carefully analyzed for the formation of acylsulfonamides for each **SZ**/**TA** combination. The amplification coefficient (AC) of each combination of reactive fragments has been calculated as the ratio between the product peak area in the incubation sample containing the protein target and the product peak area for the incubation sample lacking the protein target . Acylsulfonamides exhibiting an amplification coefficient (AC) of ≥6.5 were marked as KTGS hit compounds.

**Figure 2:**
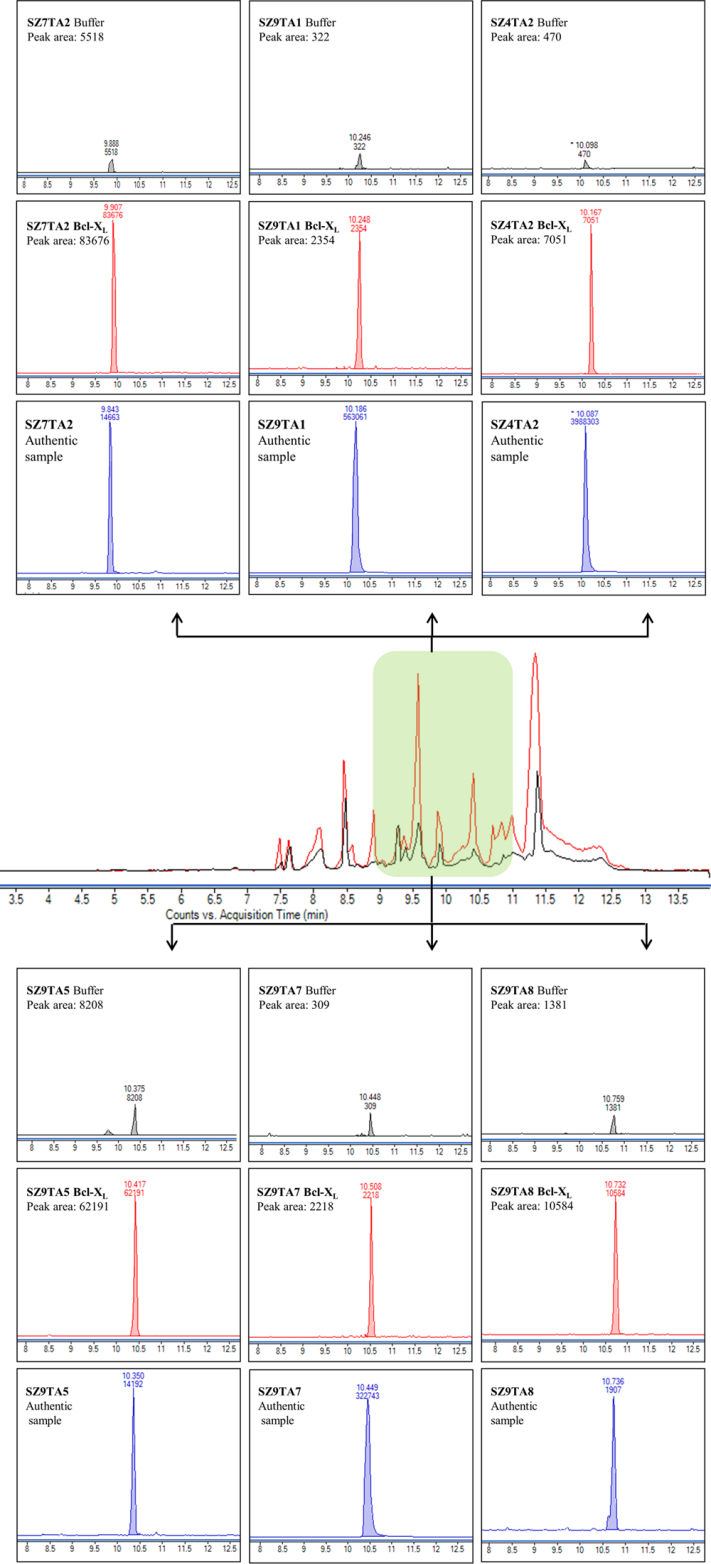
Proof-of-concept study to identify PPIMs of Bcl-X_L_ via multifragment kinetic target-guided synthesis (KTGS) incubations containing nine thio acids **TA1**–**TA9** and nine sulfonyl azides **SZ1**–**SZ9** in one well. All fragment combinations were incubated in phosphate buffer (pH 7.4) containing Bcl-X_L_ (5 μM) and in phosphate buffer alone (as a control) for 8 h. The incubation samples (one with Bcl-X_L_ and one without Bcl-X_L_) were analyzed by LC-MS/MS, monitoring acylium ions derived from the fragmentation of all theoretically possible acylsulfonamide precursor ions (one specific product ion for each acylsulfonamide product). The LC-MS/MS traces for each individual fragment combination were extracted, providing one chromatogram of the acylsulfonamide product of the protein-templated reaction (red) and one chromatogram of the acylsulfonamide product of the protein-free incubation (gray). Of all possible fragment combinations, only **SZ7TA2**, **SZ9TA1**, **SZ4TA2**, **SZ9TA5**, **SZ9TA7**, and **SZ9TA8** led to an increased amount of acylsulfonamide in the Bcl-X_L_-containing samples compared to incubation without the protein. For confirmatory purposes, synthesized hit acylsulfonamides were analyzed under identical conditions (blue) to confirm their retention times with the retention times of the protein-templated incubations.

As a confirmatory step, all KTGS hit compounds were further re-evaluated by comparing their peak retention time with the retention time of the corresponding acylsulfonamides, which were chemically synthesized and characterized by ^1^H NMR, ^13^C NMR, and HRMS. For the incubation sample containing the nine thio acids (**TA1**–**TA9**) and nine sulfonyl azides (**SZ1**–**SZ9**) in the presence of Bcl-X_L_, six acylsulfonamides (**SZ4TA2**, **SZ7TA2**, **SZ9TA5**, **SZ9TA1**, **SZ9TA7**, and **SZ9TA8**) were confirmed as KTGS hits (Figure 2). This highly sensitive, multifragment approach identified two new hits (**SZ9TA7** and **SZ9TA8**) along with the four hits originally reported in our binary fragment screen.^15b^ To our delight, **SZ9TA7** was one of several nonhit combinations tested for activity in the original report and was found to disrupt the Bcl-X_L_/Bak BH3 interaction with 45% inhibition at a concentration of 50 μM.

### Multifragment KTGS Screen against Mcl-1

**Figure 3:**
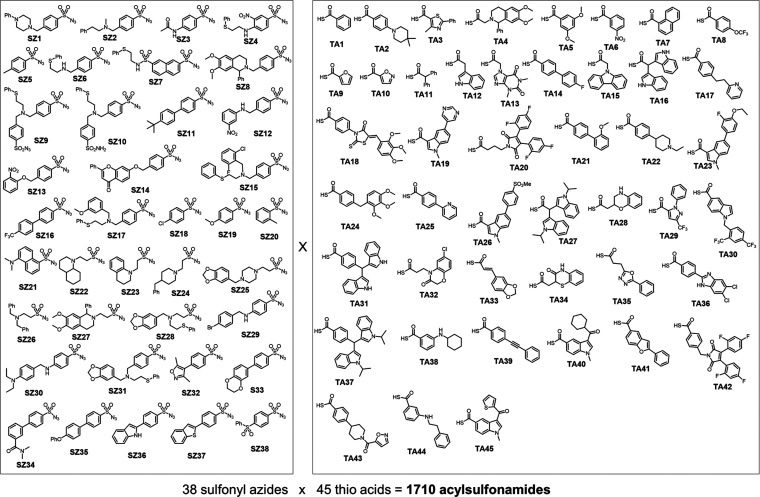
Chemical structures of the 38 sulfonyl azides (**SZ1**–**SZ38**) and 45 thio acids (**TA1**–**TA45**) used for multifragment kinetic target-guided synthesis (KTGS) screening against Mcl-1. Thio acids are generated from corresponding fluorenyl-methyl thio esters prior to screening.

Because Bcl-2 and Bcl-X_L_ are considered to be the central regulators of apoptosis, compounds binding to these targets were initially investigated. However, on the basis of clinical trials and other experiments conducted using ABT-737 and ABT-263, Mcl-1 (another member of the antiapoptotic Bcl-2 family) emerged as a crucial target contributing to cancer cell proliferation. In particular, resistance to high-affinity small molecule BH3 mimetics and selective inhibitors of Bcl-2/Bcl-X_L_ such as ABT-737 and navitoclax (ABT-263) is associated with the overexpression of Mcl-1 in multiple tumor types, including multiple myeloma. As a result, PPIMs targeting Mcl-1 selectively or in addition to the other Bcl-2 family members would be highly desired. Although multiple Mcl-1 inhibitors have been reported to date, only a handful of groups, including Pellecchia, Fesik, Zhu, Zhou, and Takeda Pharmaceutical have identified acylsulfonamides as modulators of this important target.^16^ Inspired by these reports, we decided to perform KTGS screening against Mcl-1 for the identification of novel acylsulfonamide PPIMs. For this purpose, the library of building blocks was also increased to 38 sulfonyl azides and 45 thio acids leading to a total of 1710 possible acylsulfonamides (Figure 3). Structural motifs of previously reported PPIMs were incorporated in the design of these new reactive fragments, provided that the synthetic route demanded fewer than six linear steps. The library of reactive fragments is comprised of various heterocyclic compounds such as indole and bis-indole systems, biphenyls linked to alkyl chains, various N- and O-heterocyclic or heteroaromatic scaffolds, and others. These molecules were functionalized with either a sulfonyl azide or a fluorenyl-methyl-protected thio acid to circumvent challenges associated with the limited stability or difficult isolation of thio acids. To better understand the kinetics of the amidation reaction in the presence and absence of the protein target, concentrations of acylsulfonamides in incubation mixtures varying in terms of fragment combinations as well as numbers of fragment combinations were determined after 4, 8, 10, 12, and 24 h. One set of incubations contained 38 sulfonyl azides and five thio acids (190 fragment combinations), while the other set was comprised of 31 sulfonyl azides and 10 thio acids (310 fragment combinations). While a steady increase in the extent of acylsulfonamide formation was observed with an extended incubation time, a period of at least 8–10 h was necessary to reliably measure small quantities of acylsulfonamides in the protein-free mixtures. Peak areas and peak shapes, including S/N ratios, were better in the incubations containing 190 fragment combinations than in the incubations with 310 fragment combinations. These observations are likely related to the reduced instrument dwell time allotted for each acylsulfonamide product as well as the detection of a reduced number of their specific acylium fragmentation ions. Therefore, it was concluded that 190 fragment combinations in one well, comprised of five thio acids and 38 sulfonyl azides, was a manageable number of masses to observe simultaneously, providing reliable and reproducible results with acceptable S/N ratios and good quality LC-MS/MS traces. Inspired by these preliminary results, we screened the library of building blocks in a 190-fragment combination per well format against Mcl-1 (see Figure S2). The reactive fragments were organized into nine wells, each equipped with five thio acids (**TA**s) and all 38 sulfonyl azides (**SZ**s) (20 μM final concentration of each fragment) along with Mcl-1 [10 μM concentration in phosphate buffer (pH 7.4)]. These multifragment mixtures were also incubated in a phosphate buffer in the absence of Mcl-1. All samples were incubated at 37 °C for 10 h before subsequent TQMS LC-MS/MS analysis. A comparison of the obtained chromatograms in the presence and absence of Mcl-1 revealed 90 potential KTGS hit combinations, bearing AC values in the range of 3–57. A narrower selection was performed in which acylsulfonamides with AC values of ≥6.5 were chosen for subsequent validation through a second KTGS experiment utilizing binary incubation mixtures followed by a comparison of peak retention times to that of the corresponding authentic sample. Thus, 60 acylsulfonamides met the 6.5 AC criteria and were selected for the second tier of evaluation. These 60 KTGS hit combinations were incubated as binary reaction mixtures, and another selection criterion was applied in which only the KTGS hit combinations with AC values of ≥4 in the binary experiment were chosen. As a result, 51 acylsulfonamides were subsequently authenticated by comparison of peak retention times with that of the corresponding synthesized sample.

### Fluorescence Polarization Data of KTGS Hits

The 51 down-selected acylsulfonamides were resynthesized and tested for the ability to disrupt Mcl-1/BH3 interactions using a conventional fluorescence polarization (FP) assay utilizing GST-Mcl-1 and the fluorescein-labeled Bim BH3 peptide (see Table S3). A large number of the tested acylsulfonamides displayed inhibition percentages against the biological target in the range of 80–100% (28 compounds) at a concentration of 50 μM, with all remaining compounds having <79% inhibition. The acylsulfonamides were then subjected to dose–response studies using FP, which revealed 24 active compounds with nine exhibiting single-digit μM IC_50_ values, seven with affinity values ranging from 10 to 20 μM, and seven with IC_50_ values in the range of 20–70 μM (Table 1). To probe whether these molecules would display any biological activity against Bcl-X_L_, they were subjected to the FP assay designed using GST-Bcl-X_L_ and fluorescein-labeled Bak BH3 peptide.^15a,15b^ Selectivity indices, defined as the quotient of the IC_50_ toward Bcl-X_L_ over the IC_50_ toward Mcl-1 , were calculated to gauge the selectivity of hits toward Mcl-1 when compared to Bcl-X_L_. Of the compounds tested, 13 were identified as selective toward Mcl-1, with selectivity indices ranging from 1.5 to 8.1. Notably, acylsulfonamides **SZ31TA15**, **SZ12TA42**, and **SZ31TA24** showed affinity for Bcl-X_L_ below the detectable threshold. It is also worth mentioning that through this study, a selective Bcl-X_L_ inhibitor, **SZ4TA17**, with an IC_50_ of 2.1 μM was identified (Mcl-1 IC_50_ = 61 μM).

**Table 1:** FP Data of the 24 Bioactive Hits Identified from a Multifragment KTGS Screen against Mcl-1^a^

compound	IC_50_ on Bcl-X_L_ (μM)	IC_50_ on Mcl-1 (μM)	selectivity index
**SZ17TA3**	50	8.6	5.8
**SZ15TA3**	36.4	5.8	6.3
**SZ17TA7**	72	20.1	3.6
**SZ15TA7**	NA	15.4	NA
**SZ31TA15**	ND	14	NA
**SZ9TA7**	66.3	8.2	8.1
**SZ31TA3**	NA	9.4	NA
**SZ9TA1**	28.8	19.8	1.5
**SZ15TA1**	29.1	9.7	3.0
**SZ15TA5**	53	13.4	4.0
**SZ11TA40**	NA	21.9	NA
**SZ17TA8**	NA	8.4	NA
**SZ9TA5**	36	8.1	4.4
**SZ31TA8**	NA	5.9	NA
**SZ15TA8**	47	7.6	6.2
**SZ9TA17**	NA	20.4	NA
**SZ4TA30**	>50	26.1	NA
**SZ4TA17**	2.08	60.6	0.03
**SZ15TA25**	54.9	15.3	3.6
**SZ16TA44**	NA	31.5	NA
**SZ15TA17**	55.9	20.2	2.8
**SZ31TA24**	ND	20	NA
**SZ32TA42**	43.8	19.1	2.3
**SZ12TA42**	ND	13.6	NA

a NA = not available. ND = not detected.

**Figure 4:**
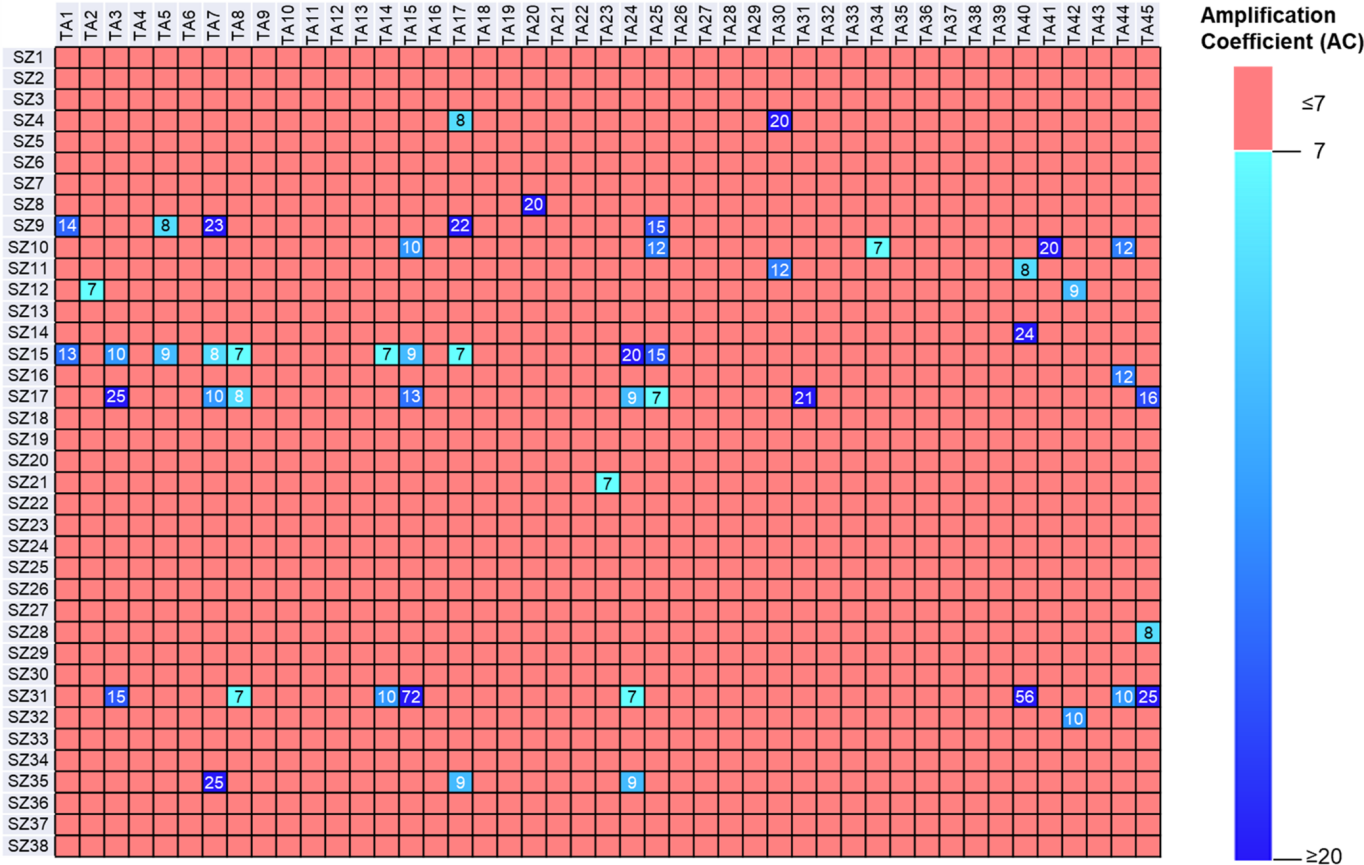
Matrix displaying the 51 acylsulfonamide hits and their amplification coefficients  from a multifragment kinetic target-guided synthesis (KTGS) screen against Mcl-1. 45 thio acids (**TA1**–**TA45**) and 38 sulfonyl azides (**SZ1**–**SZ38**) were used in the screen, leading to a total of 1710 possible acylsulfonamide combinations (hit rate of ∼3%).

Careful observation of various acylsulfonamides derived from different building blocks provided useful structural information leading to the conclusion that particular fragments were preferentially selected by Mcl-1 and participated in the formation of a larger number of acylsulfonamide hits. This is highlighted in Figure 4, where the recurring fragments in the hit list can be easily identified. Importantly, these data provide initial structure–activity relationship (SAR) information prior to the synthesis of any of the compounds, whereas a traditional SAR would require synthesis of all hits before subsequent biological evaluation. Specifically, screening well 1 (containing **SZ1**-**31** × **TA1**-**5**), well 2 (containing **SZ1**-**31** × **TA6**-**10**), and well 6 (containing **SZ1**-**31** × **TA21**-**25**) stood out, as these wells produced a greater number of KTGS hits that also displayed the best inhibition values. Thus, acylsulfonamides generated from sulfonyl azides bearing bis-benzylic tertiary amines (**SZ15**, **SZ17**, **SZ31**, **SZ9**, and **SZ10**) as well as those possessing biaryl scaffolds (**SZ35** and **SZ11**) were predominant fragments involved in the formation of the KTGS hit combinations. In contrast, sulfonyl azides that were comparatively smaller (**SZ3**, **SZ5**, **SZ18**, **SZ19**, and **SZ20**) or contained alkyl fragments (**SZ22** and **SZ24**) were found to be not templated by the protein target. On the contrary, thio acids such as **TA3**, **TA7**, and **TA8** were found to be favored during the KTGS screen, although thio acids **TA1**, **TA5**, **TA17**, and **TA25** also featured in some of the PPIMs. Interestingly, heterocyclic thio acids (**TA9**, **TA10**, **TA26**, **TA32**, and **TA33**) did not contribute to the KTGS hits.

Notably, when sulfonyl azide fragments **SZ1**–**SZ31** and the first 10 thioacids (**TA1**–**TA10**) were tested individually in the fluorescence polarization assay at a concentration of 100 μM, <5% inhibition was detected for all fragments with the exception of four sulfonyl azides: **SZ4** (23%), **SZ9** (13%), **SZ15** (25%), and **SZ27** (14%). These results demonstrate that high-quality inhibitors can be unambiguously identified through the screening of fragments possessing weak binding affinities, remarkably reducing synthetic efforts.

### Structural Network Similarity and Docking Studies

To better visualize the structural features of the obtained hits, the 51 hit compounds were clustered (StarDrop) on the basis of two-dimensional similarity using their corresponding Murcko scaffolds and applying a Tanimoto coefficient threshold of >0.8 (Supporting Information). The process yielded eight singletons and 11 clusters, seven of which contain only two members and one larger cluster comprised of 19 hits. Careful observation of all acylsulfonamides identified through KTGS screening provided some useful structural information. For example, the large cluster of 19 compounds is populated with structures derived from sulfonyl azides bearing bis-benzylic tertiary amines **SZ15** (seven hits), **SZ17** (four hits), **SZ9** (four hits), **SZ31** (two hits), and **SZ10** (one hit) and was found to be among the most active clusters against Mcl-1. A smaller cluster of five hits was found to incorporate one thio acid, **TA15**, in all cluster members. Similarly, in all of the two-membered clusters, one **SZ** or **TA** is present in both members. One of the most active KTGS hits, **SZ17TA3**, clusters with only one other hit, while reference furan-5-carboxylic acid derivative **60** reported by the Fesik group^17^ is a singleton suggesting a rather unique structural motif for the two compounds. Interestingly, none of the alkyl sulfonyl azides (**SZ22**–**SZ28**) led to any of the KTGS hits (Figure 4). Moreover, other sulfonyl azides, being comparatively smaller, did not engage in protein templation. In addition, while some thio acids delivered higher numbers of KTGS hits (**TA23**, **TA17**, **TA19**, and others), there were several thio acids (**TA27**, **TA28**, **TA37**, and **TA38**) that showed no templation effect with Mcl-1.

**Figure 5:**
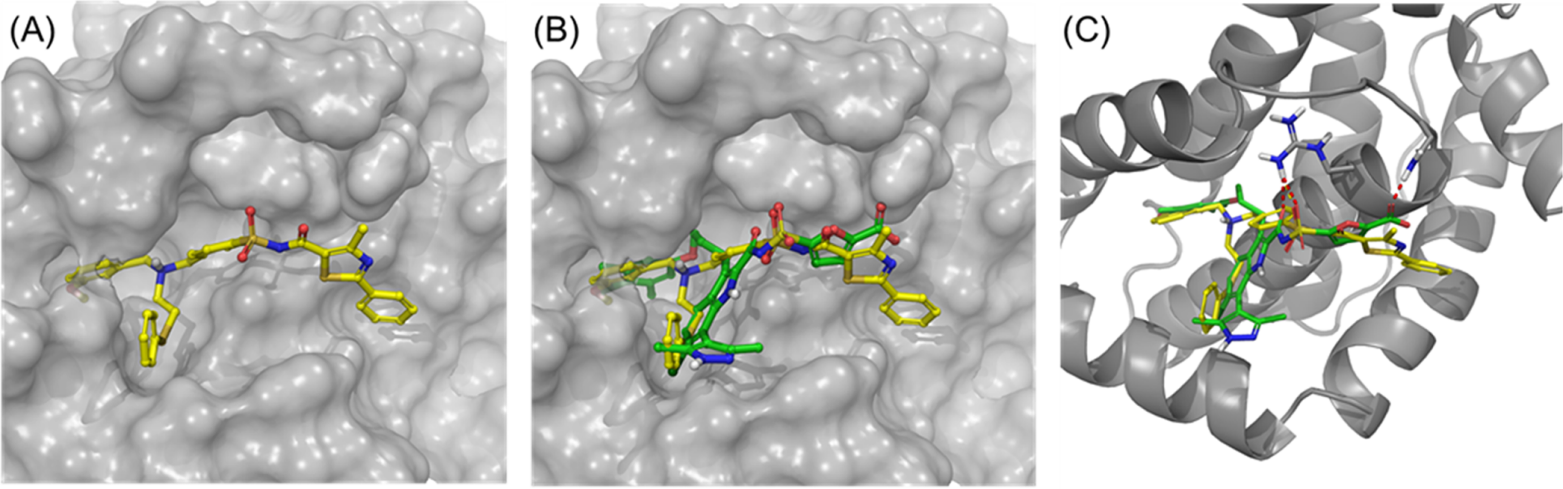
(A) Docking of **SZ17TA3** into the Mcl-1 (PDB entry 5FDR) BH3 groove. (B) Overlay of **SZ17TA3** (yellow) and furan-5-carboxylic acid derivative **60**(17) (green) in the binding pocket of Mcl-1. (C) Polar contacts of **SZ17TA3** and compound **60** to R263 and N260 illustrated by red dotted lines.

To gain further insight into the protein-templated selection of 51 acylsulfonamides among the 1710 possible combinations and elucidate the preferred mode of binding to the BH3 groove of Mcl−1, we performed docking studies (Glide, Schrodinger). Thus, all 51 KTGS hits were docked into an Mcl-1 grid obtained from a recently reported crystal structure of furan-5-carboxylic acid derivative **60** bound to Mcl-1 [Protein Data Bank (PDB) entry 5FDR] and compared docked poses between the hits and the reference. Consequently, a majority of the KTGS hits occupy both pockets P2 and P4 of the BH3 binding groove, which could explain the relative potency of the compounds as well as the preferred selection for protein templation. For example, the binding pose of one of the KTGS hits, **SZ17TA3**, alone (A) and as an overlay with reference acid **60** (B) is shown in Figure 5. The methoxybenzyl moiety of **SZ17TA3** sits deeply in the P2 hydrophobic pocket of Mcl-1, similar to the *p*-chloroaryl group of compound **60**, while the second thioether aromatic ring projects outside (Figure 5A,B). At the same time, the thio acid portion (phenylthiazole) of **SZ17TA3** extends elegantly into the P4 pocket of the protein (Figure 5A), a position occupied by the furan-5-carboxylic acid portion of **60** (Figure 5B). Similarly, the sulfonyl oxygen of **SZ17TA3** is involved in key hydrogen bond interactions with Arg263 of Mcl-1 (Figure 5C). The docking poses of **SZ17TA3** and other KTGS hits provide an excellent basis for further structure-based drug design. In the case of **SZ17TA3**, for example, one might expect an improvement in potency as a result of (i) introduction of a hydrogen bond acceptor/donor into the P4 pocket to establish an interaction with Asn260 or (ii) modification of the P2 pocket occupying methoxyphenyl ring with a halogen group to strengthen electron-withdrawing effects.

## Conclusion

KTGS offers an attractive and unique alternative to conventional drug discovery approaches because it allows the biological target to select and ligate only the building blocks that best fit into its binding sites. Improving upon our previously reported KTGS study involving binary fragment mixtures targeting the PPIs of Bcl-X_L_, we validated herein for the first time that a multifragment KTGS approach coupled with TQMS detection can be implemented for the identification of PPI modulators. The optimized screening conditions utilizing SRM to monitor multiple acylsulfonamide products were found to significantly improve the sensitivity of acylsulfonamide product detection, leading to an ∼200-fold increase in throughput, an increased coverage of chemical space with nearly 2000 possible ligation products, and improved accuracy and reliability in detecting KTGS hit compounds. Consequently, the presented multifragment approach has greatly streamlined the KTGS screening method while accelerating the hit identification process, especially when screening fragment libraries with thousands of potential ligation products. In a subsequent study, a structurally diverse library of 45 thio acids and 38 sulfonyl azides (1710 potential acylsulfonamide products) was screened against Mcl-1, generating 51 KTGS hits. Remarkably, fluorescence polarization (FP) studies revealed that 24 of these hit compounds displayed appreciable inhibitory activity against Mcl-1, including several candidates possessing single-digit micromolar IC_50_ values. Extensive docking studies provided further insights into the binding modes of these inhibitors, with **SZ17TA3** adopting a docking pose exhibiting a striking resemblance to the crystal structure pose of Mcl-1 inhibitor **60** when bound to Mcl-1.

Our established high-throughput strategy allows for the screening of 1710 compounds, while necessitating the synthesis of only 83 fragments. Furthermore, our multifragment approach greatly expands the selection pool per incubation sample to 190 combinations, representing the highest number of combinations from which the protein can select. The presented KTGS strategy is generally applicable and has the potential to be utilized for the screening of other protein–protein interaction targets and could also be implemented for rapid and cost-effective identification of promising hit candidates against a range of targets. While the current study employs fragments derived from structures known to partake in interactions with the target of interest, further studies aimed at applying a “shotgun strategy” utilizing the same 83-member fragment library to other targets are ongoing.

## Experimental Section

### General Information

All reagents and solvents were obtained from Sigma-Aldrich, Oakwood Products, Inc., or TCI America and used without further purification. Analytical thin layer chromatography (TLC) was performed on silica gel 60 F254 precoated plates (0.25 mm) from EMD Chemical Inc., and components were visualized by ultraviolet light (254 nm) and TLC staining solutions [phosphomolybdic acid (PMA), KMnO_4_ solution, and/or a Ce(SO_4_)_2_/ammonium phosphomolybdate/10% H_2_SO_4_ solution followed by heating]. Reported *R_f_* values were determined for TLC. EMD silica gel 60 (particle size of 40–63 μm) 230–400 mesh was used for column chromatography. ^1^H NMR spectra were recorded at ambient temperature on a 250 MHz Bruker, 400 MHz Varian, 500 MHz Varian, or 600 MHz Varian NMR spectrometer in the indicated solvent. All ^1^H NMR experiments are reported in δ units, parts per million downfield of TMS, and were measured relative to the signals for chloroform (7.26 ppm), methanol (3.31 ppm), and dimethyl sulfoxide (2.50 ppm). ^13^C NMR spectra were recorded at ambient temperature at 62.5 MHz, 100 MHz Varian, 125 MHz, or 150 MHz in the indicated solvent. All ^13^C NMR spectra are reported in parts per million relative to the signals for chloroform (77.16 ppm), methanol (49 ppm), or dimethyl sulfoxide (39.5 ppm) with ^1^H decoupled observation. ^1^H NMR data are reported as follows: chemical shift (δ), multiplicity (s, singlet; d, doublet; t, triplet; q, quartet; p, pentet; sext, sextet; sept, septet; oct, octet; m, multiplet), integration, and coupling constant (Hz). ^13^C NMR analyses are reported in terms of chemical shift. NMR data were analyzed by using MestReNova Software version 6.0.2-5475. The purity of the final compounds was determined to be ≥95% by high-pressure liquid chromatography (HPLC) using an Agilent 1200 LC instrument coupled to an Agilent G1946D MSD-VL instrument with electrospray ionization. Low-resolution mass spectra were recorded on an Agilent G1946D MSD-VL instrument with electrospray ionization, whereas high-resolution mass spectra (HRMS) were recorded on an Agilent 6540 LC/MSD TOF system.

### General Protocol for KTGS Incubations of Multiple Building Block Combinations with Bcl-X_L_

As a proof-of-concept study, KTGS screening of the previously reported library of nine thio acids and nine sulfonyl azides against Bcl-X_L_ was accomplished in one well.^15b^ An additional incubation sample consisting of the building blocks in buffer was utilized for the control experiment in the absence of the target protein, Bcl-X_L_.

#### Preparation of Stock Solutions Containing Nine Sulfonyl Azide Building Blocks

Each sulfonyl azide was first prepared as a 2 mM solution in methanol. Next, equal amounts (100 μL) of these 2 mM methanolic solutions were combined in one vial. The solvent was completely evaporated off, and the residue was subsequently solubilized with 100 μL of fresh methanol providing a stock solution of a mixture of nine sulfonyl azides (each sulfonyl azide at concentration of 2 mM).

#### Preparation of Stock Solutions Containing Nine Thio Acid Building Blocks

The stock solutions containing mixtures of nine thio acids were prepared in a two-step process. In the first step, fluorenylmethyl thioesters were deprotected individually to yield the corresponding thio acids. Approximately 500 μg of a single thio ester was weighed out in a 1.5 mL Eppendorf tube and mixed with a freshly prepared deprotection solution consisting of 5% piperidine in anhydrous DMF. The exact amount of deprotecting solution was calculated according to a previously reported protocol;^15c^ 1.0 μL of the deprotecting solution (5% piperidine in anhydrous DMF) was used for 4.7 μmol of thioester. Each reaction mixture was kept at room temperature for approximately 5 min to complete the deprotection reaction generating the corresponding thio acid. Subsequently, without further purification, each reaction mixture was diluted in the Eppendorf tube with methanol yielding a methanolic 20 mM thio acid solution. Finally, equal amounts of methanolic 20 mM thio acid stock solutions were mixed and further diluted with methanol to obtain a stock solution of a mixture of nine thio acids (each thio acid at 2 mM), which was further used for the preparation of the KTGS incubation solution.

#### Preparation of Phosphate Buffer and Protein Stock Solutions

A phosphate buffer (pH 7.4; 58 mM Na_2_HPO_4_, 17 mM NaH_2_PO_4_, 68 mM NaCl, and 1 mM NaN_3_) and a 5 μM Bcl-X_L_ stock solution in phosphate buffer (pH 7.4; 58 mM Na_2_HPO_4_, 17 mM NaH_2_PO_4_, 68 mM NaCl, and 1 mM NaN_3_) were prepared to conduct the KTGS incubation reactions.

#### KTGS Incubation Reactions

The incubation reaction mixtures were prepared in a 96-well plate by adding 1 μL of the stock solution containing nine thio acids (each thio acid at 2 mM) and 1 μL of the stock solution containing nine sulfonyl azides (each sulfonyl azide of 2 mM) to 98 μL of the target protein solution (5 μM Bcl-X_L_ in phosphate buffer). For the control incubations in the absence of the protein target, 1 μL of the stock solution containing nine thio acids (each thio acid at 2 mM) and 1 μL of the stock solution containing nine sulfonyl azides (each sulfonyl azide at 2 mM) were added to 98 μL of the phosphate buffer solution alone (protein target missing). The 96-well plate was sealed and incubated at 37 °C for 10–12 h. The incubation mixtures were then subjected to LC-MS/MS (triple quadrupole mass spectrometry detector in MRM mode, MRM following 190 parent ions in Q1 and the corresponding five acylium ions in Q3), utilizing a Kinetex PFP column [2.6 μm, 100 Å (4.6 mm × 50 mm)] preceeded by a Phenomenex security guard C18 cartridge. Ten microliters of the incubation samples was directly injected and eluted at 37 °C using a gradient (Table S2).

### General Protocol for KTGS Incubations of Multiple Building Block Combinations with Mcl-1

The KTGS incubations of the entire library of 45 thio acid and 38 sulfonyl azide building blocks were accomplished by dividing the 1710 possible building block combinations into nine individual incubation mixtures. Each of these nine incubation mixtures was comprised of five thio acids and 38 sulfonyl azides (Figure S2).

#### Preparation of Stock Solutions Containing 38 Sulfonyl Azide Building Blocks

Each sulfonyl azide was first prepared as a 2 mM solution in methanol. Next, equal amounts (100 μL) of these 2 mM methanolic solutions were combined in one vial. The solvent was completely evaporated off, and the residue was subsequently solubilized with 100 μL of fresh methanol providing a stock solution of a mixture of 38 sulfonyl azides (each sulfonyl azide at a concentration of 2 mM).

### Preparation of Stock Solutions Containing Five Thio Acid Building Blocks

The stock solutions containing mixtures of five thio acids were prepared in a two-step process. In the first step, fluorenylmethyl thioesters were deprotected individually to yield the corresponding thio acids. Approximately 500 μg of a single thio ester was weighed out in a 1.5 mL Eppendorf tube and mixed with a freshly prepared deprotection solution consisting of 5% piperidine in anhydrous DMF. The exact amount of deprotecting solution was calculated according to a previously reported protocol;^15c^ 1.0 μL of the deprotecting solution (5% piperidine in anhydrous DMF) was used for 4.7 μmol of thioester. Each reaction mixture was kept at room temperature for approximately 5 min to complete the deprotection reaction generating the corresponding thio acid. Subsequently, without further purification, each reaction mixture was diluted in the Eppendorf tube with methanol yielding a methanolic 20 mM thio acid solution. Finally, equal amounts of methanolic 20 mM thio acid stock solutions were mixed and further diluted with methanol to obtain a stock solution of a mixture of five thio acids (each thio acid at 2 mM), which was further used for the preparation of the KTGS incubation solution.

#### Preparation of Phosphate Buffer and Protein Stock Solutions

A phosphate buffer (pH 7.4; 58 mM Na_2_HPO_4_, 17 mM NaH_2_PO_4_, 68 mM NaCl, and 1 mM NaN_3_), and a 10 μM Mcl-1 stock solution in phosphate buffer (pH 7.4; 58 mM Na_2_HPO_4_, 17 mM NaH_2_PO_4_, 68 mM NaCl, and 1 mM NaN_3_) were prepared to conduct all KTGS incubation reactions.

#### KTGS Incubation Reactions

The incubation reaction mixtures were prepared in a 96-well plate by adding 1 μL of the stock solution containing five thio acids (each thio acid at 2 mM) and 1 μL of the stock solution containing 38 sulfonyl azides (each sulfonyl azide at 2 mM) to 98 μL of the target protein solution (10 μM Mcl-1 in phosphate buffer solution or 5 μM Bcl-X_L_ in phosphate buffer solution). For the control incubations in the absence of the protein target, 1 μL of the stock solution containing five thio acids (each thio acid at 2 mM) and 1 μL of the stock solution containing 38 sulfonyl azides (each sulfonyl azide at 2 mM) were added to 98 μL of the phosphate buffer solution alone (protein target missing). The 96-well plate was sealed and incubated at 37 °C for 10–12 h. The incubation mixtures were then subjected to LC-MS/MS (triple quadrupole mass spectrometry detector in MRM mode, MRM following 190 parent ions in Q1 and the corresponding five acylium ions in Q3), utilizing a Kinetex PFP column [2.6 μm, 100 Å (4.6 mm × 50 mm)] preceeded by a Phenomenex security guard C18 cartridge. Ten microliter portions of the incubation samples were directly injected and eluted at 37 °C using a gradient (Table S2).

### Fluorescence Polarization Studies

The detailed protocol for conducting fluorescence polarization-based competitive binding assays has been previously reported.^18^ Briefly, 20 μL of 20 nM GST-tagged mouse Mcl-1-(152–309) in PBS containing 0.005% Tween 20 was mixed with 5 μL of the acylsulfonamide at various concentrations in PBS containing 25% DMSO and 0.005% Tween 20 in the wells of a 96-well black polystyrene plate. Then, 25 μL of 10 nM FITC-Bim-BH3 in PBS containing 5% DMSO and 0.005% Tween 20 was added to each well, and the mixtures were thoroughly mixed at room temperature for 3 min at 1450 rpm. The fluorescence polarization values in millipolarization (mP) units were measured for 0.2 s at excitation and emission wavelengths of 480 and 535 nm, respectively, using a multilabel plate reader. IC_50_ values were determined by fitting the data to a sigmoidal dose–response nonlinear regression model using SigmaPlot 10.0.1. *K*_i_ values were then calculated using the equation *K*_i_ = [I]_50_/([L]_50_/*K*_d_ + *P*_0_/*K*_d_ + 1), where [I]_50_ and [L]_50_ are the free concentrations of the inhibitor and ligand, respectively, at 50% inhibition, *P*_0_ is the free concentration of protein in the absence of an inhibitor, and *K*_d_ is the dissociation constant of the Bim:GST-Mcl-1 complex and has a value of 12.4 nM.

## Supporting Information Available

Triple quadrupole fragmentation studies, synthesis of small molecule fragments and *N-*acylsulfonamides, NMR data, and HPLC traces (PDF)
Molecular formula strings (CSV)
Compound clustering results (CSV)
Additional data (PDB)

## Supplementary Material

jm3c00108_si_003.csv

jm3c00108_si_004.pdb

## Abbreviations

KTGS: kinetic target-guided synthesis
PPI: protein–protein interaction
SRM: selected reaction monitoring
MRM: multiple reaction monitoring
SIM: selected ion mode
FBDD: fragment-based drug design
TQMS: triple quadrupole mass spectrometer
TE: thio ester
TA: thio acid
SZ: sulfonyl azide
