# Supplementary material for: Going beyond Binary: Rapid Identification of Protein–Protein Interaction Modulators Using a Multifragment Kinetic Target-Guided Synthesis Approach
Source: J Med Chem. 2023 Mar 31;66(7):5196–207. doi: 10.1021/acs.jmedchem.3c00108 (PMC10620989; doi:10.1021/acs.jmedchem.3c00108)
Supplement: Supplementary file 1 — jm3c00108_si_001.pdf [file jm3c00108_si_001.pdf]

Supplementary Information

Going Beyond Binary: Rapid Identification of Protein–Protein Interaction Modulators Using a Multi-Fragment Kinetic Target-Guided Synthesis Approach

Katya Nacheva,[a#] Sameer S. Kulkarni,[a#] Mintesinot Kassu,[b] David Flanigan,[a,c] Andrii Monastyrskyi,[a] Iredia D. Iyamu,[a,b] Kenichiro Doi,[d] Megan Barber,[a] Niranjan Namelikonda,[a] Jeremiah D. Tipton,[e] Prakash Parvatkar,[a] Hong-Gang Wang,[d] Roman Manetsch,[a,b,f,g*]

[a]Department of Chemistry, University of South Florida, Tampa, Florida 33620, USA.

[b]Department of Chemistry and Chemical Biology, Northeastern University, Boston, MA 02115, USA.

[c]Department of Sciences, Hillsborough Community College, Tampa, FL 33619, USA.

[d]Department of Pharmacology and Penn State Hershey Cancer Institute, Penn State College of Medicine, Hershey, PA 17033, USA.

[e]Proteomics and Mass Spectrometry Core Facility, University of South Florida, Tampa, Florida 33620, USA.

[f]Department of Pharmaceutical Sciences, Northeastern University, Boston, MA 02115, USA.

[g]Center for Drug Discovery, Northeastern University, Boston, MA 02115, USA.

[#]Equal contribution

[*]Corresponding author

e-mail: r.manetsch@northeastern.edu

Table of Contents

Triple Quadrupole Fragmentation Studies **S3**

LC-MS Traces for Multi-Fragment KTGS with Mcl-1 .... **S6**

Clustering and Docking Studies **S16**

Initial Fluorescence Polarization Data of KTGS Hits **S17**

Synthetic Procedures and Compound Characterization **S18**

HPLC Traces of Select Hit Compounds **S89**

References **S94**

1. Triple Quadrupole Fragmentation Studies

In order to establish correlation between the collision energies and the fragmentation pathways several acylsulfonamides, **SZ1TA3**, **SZ2TA2**, **SZ7TA2**, **SZ2TA4**, **SZ9TA7**, **SZ7TA7**, **SZ6TA7**, **SZ9TA1**, and **SZ8TA8**, were subjected to direct infusion injections and the collision-activated defragmentation curves were recorded (**Figure S1**). The corresponding acylium ion was present in all investigated acylsulfonamides and an average collision energy of 30 – 35 V was determined for the nine compounds tested (**Table S1**).

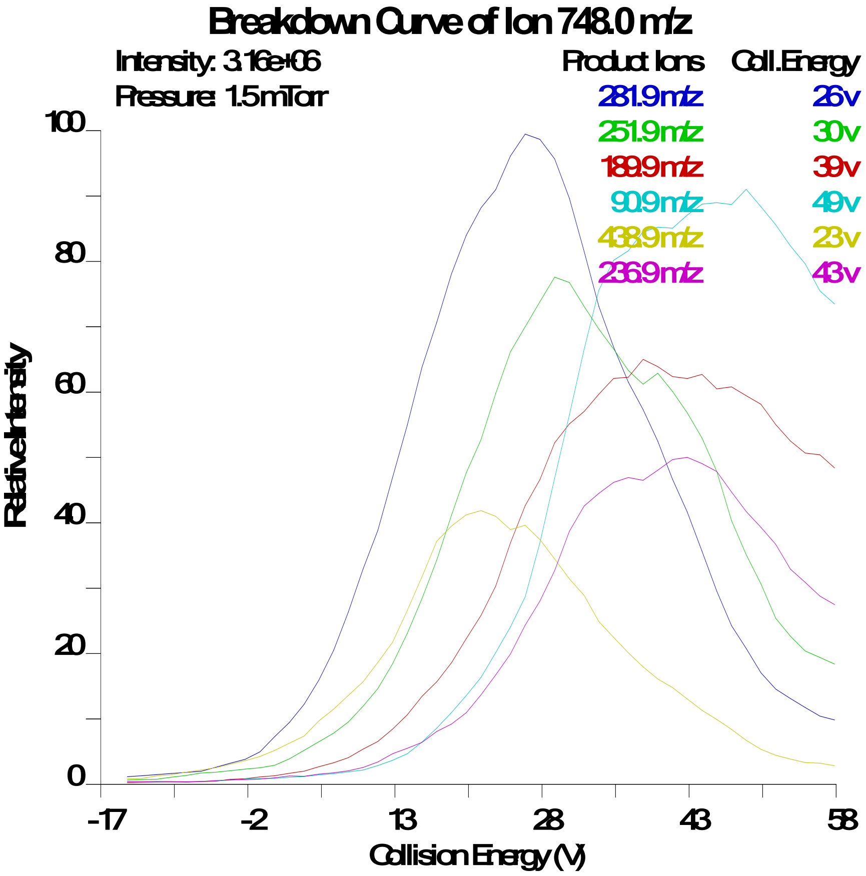

Figure S1. Collision-activated defragmentation curve of acylsulfonamide **SZ8TA4**. Note, of all possible fragmentation pathways, the one leading to an acylium ion has the highest relative ion abundance and requires the lowest collision energy (among the most abundant ions). The relative abundance of each ion represents the number of times an ion with a certain m/z ratio is detected.

Table S1. Collision energies (eV) at which acylium ions are generated from corresponding acylsulfonamides through the defragmentation process.

Acyl sulfonamide	Collision energy (eV)
**SZ1TA3**	29
**SZ2TA2**	26
**SZ7TA2**	28
**SZ2TA4**	22
**SZ8TA4**	26
**SZ9TA7**	37
**SZ7TA7**	31
**SZ6TA7**	31
**SZ9TA1**	48
**SZ8TA8**	36

Table S2. Elution gradient employed for analysis of kinetic TGS incubations.

Time	% A*	% B*	Flow rate
0.00	90	10	0.7 mL min^{-1}
2.00	90	10	0.7 mL min^{-1}
15.00	5	95	0.7 mL min^{-1}
17.00	90	10	0.7 mL min^{-1}

* eluent A: H_2O (0.05% TFA); eluent B: CH_3CN (0.05% TFA)

	Well 1					Well 2					Well 3				
	TA1	TA2	TA3	TA4	TA5	TA6	TA7	TA8	TA9	TA10	TA11	TA12	TA13	TA14	TA15
SZ1	436.2	547.3	533.2	641.3	496.2	481.1	486.2	520.1	426.1	427.1	526.2	489.2	552.2	530.2	539.2
SZ2	409.2	520.3	506.1	614.3	469.2	454.1	459.2	493.1	399.1	400.1	499.2	462.2	525.2	503.2	512.2
SZ3	319.1	430.2	416.1	524.2	379.1	364.1	369.1	403.0	309.0	310.0	409.1	372.1	435.1	413.1	422.1
SZ4	458.1	569.2	555.1	663.2	518.1	503.1	508.1	542.1	448.1	449.1	548.1	511.1	574.1	552.1	561.1
SZ5	276.1	387.2	373.1	481.2	336.1	321.0	326.1	360.0	266.0	267.0	366.1	329.1	392.1	370.1	379.1
SZ6	427.1	538.2	524.1	632.2	487.1	472.1	477.1	511.1	417.1	418.1	517.2	480.1	543.1	521.1	530.1
SZ7	527.1	638.2	624.1	732.2	587.1	572.1	577.1	611.1	517.0	518.0	617.1	580.1	643.1	621.1	630.1
SZ8	543.2	654.3	640.2	748.3	603.2	588.2	593.2	627.2	533.2	534.2	633.2	596.2	659.2	637.2	646.2
SZ9	622.1	733.2	719.1	827.2	682.1	667.1	672.1	706.1	612.1	613.1	712.2	675.1	738.2	716.1	725.2
SZ10	596.1	707.2	693.1	801.2	656.1	641.1	646.1	680.1	586.1	587.1	686.2	649.2	712.2	690.1	699.2
SZ11	394.1	505.2	491.1	599.3	454.2	439.1	444.2	478.1	384.1	385.1	484.2	447.2	510.2	488.2	497.2
SZ12	412.1	523.2	509.1	617.2	472.1	457.1	462.1	496.1	402.1	403.1	502.1	465.1	528.1	506.1	515.1
SZ13	413.1	524.2	510.1	618.2	473.1	458.1	463.1	497.1	403.1	404.0	503.1	466.1	529.1	507.1	516.1
SZ14	512.1	623.2	609.1	717.2	572.1	557.1	562.1	596.1	502.1	503.1	602.2	565.1	628.1	606.1	615.2
SZ15	569.1	680.2	666.1	774.2	629.1	614.1	619.1	653.1	559.1	560.1	659.2	622.1	685.1	663.1	672.1
SZ16	406.1	517.2	503.1	611.2	466.1	451.0	456.1	490.0	396.0	397.0	496.1	459.1	522.1	500.1	509.1
SZ17	547.2	658.3	644.2	752.3	607.2	592.1	597.2	631.1	537.1	538.1	637.2	600.2	663.2	641.2	650.2
SZ18	296.0	407.1	393.0	501.1	356.0	341.0	346.0	380.0	286.0	287.0	386.1	349.0	412.0	390.0	399.0
SZ19	292.1	403.2	389.1	497.2	352.1	337.0	342.1	376.0	282.0	283.0	382.1	345.1	408.1	386.1	395.1
SZ20	276.1	387.2	373.1	481.2	336.1	321.0	326.1	360.0	266.0	267.0	366.1	329.1	392.1	370.1	379.1
SZ21	355.1	466.2	452.1	560.2	415.1	400.1	405.1	439.1	345.1	346.1	445.2	408.1	471.1	449.1	458.1
SZ22	351.2	462.3	448.2	556.3	411.2	396.2	401.2	435.1	341.1	342.1	441.2	404.2	467.2	445.2	454.2
SZ23	345.1	456.2	442.1	550.2	405.1	390.1	395.1	429.1	335.1	336.1	435.2	398.1	461.2	439.1	448.2
SZ24	387.2	498.3	484.2	592.3	447.2	432.2	437.2	471.1	377.1	378.1	477.2	440.2	503.2	481.2	490.2
SZ25	432.2	543.3	529.2	637.3	492.2	477.1	482.2	516.1	422.1	423.1	522.2	485.2	548.2	526.2	535.2
SZ26	409.2	520.3	506.1	614.3	469.2	454.1	459.2	493.1	399.1	400.1	499.2	462.2	525.2	503.2	512.2
SZ27	481.2	592.3	578.2	686.3	541.2	526.2	531.2	565.2	471.2	472.1	571.2	534.2	597.2	575.2	584.2
SZ28	499.1	610.2	596.1	704.2	559.1	544.1	549.1	583.1	489.1	490.1	589.2	552.2	615.2	593.2	602.2
SZ29	445.0	556.1	542.0	650.1	505.0	490.0	495.0	529.0	435.0	436.0	535.1	498.0	561.0	539.0	548.1
SZ30	438.2	549.3	535.2	643.3	498.2	483.2	488.2	522.2	428.2	429.2	528.2	491.2	554.2	532.2	541.2
SZ31	561.1	672.2	658.1	766.3	621.2	606.1	611.2	645.1	551.1	552.1	651.2	614.2	677.2	655.2	664.2
SZ32	357.1	468.2	454.1	562.2	417.1	402.1	407.1	441.1	347.1	348.1	447.1	410.1	473.1	451.1	460.1
SZ33	396.1	507.2	493.1	601.2	456.1	441.1	446.1	480.1	386.1	387.1	486.1	449.1	512.1	490.1	499.1
SZ34	409.1	520.2	506.1	614.2	469.1	454.1	459.1	493.1	399.1	400.1	499.2	462.1	525.1	503.1	512.2
SZ35	430.1	541.2	527.1	635.2	490.1	475.1	480.1	514.1	420.1	421.1	520.2	483.1	546.1	524.1	533.1
SZ36	377.1	488.2	474.1	582.2	437.1	422.1	427.1	461.1	367.1	368.1	467.1	430.1	493.1	471.1	480.1
SZ37	394.0	505.2	491.0	599.2	454.1	439.0	444.1	478.0	384.0	385.0	484.1	447.1	510.1	488.1	497.1
SZ38	402.0	513.1	499.0	607.1	462.1	447.0	452.1	486.0	392.0	393.0	492.1	455.1	518.1	496.1	505.1

	Well 4					Well 5					Well 6				
	TA16	TA17	TA18	TA19	TA20	TA21	TA22	TA23	TA24	TA25	TA26	TA27	TA28	TA29	TA30
SZ1	604.2	541.2	745.2	567.2	721.2	542.2	547.3	627.2	616.2	513.2	643.2	688.3	521.2	570.2	701.2
SZ2	577.2	514.2	718.2	540.2	694.2	515.2	520.3	600.2	589.2	486.2	616.2	661.3	494.2	543.2	674.2
SZ3	487.1	424.1	628.1	450.1	604.1	425.1	430.2	510.1	499.1	396.1	526.1	571.2	404.1	453.1	584.1
SZ4	626.1	563.1	767.1	589.1	743.1	564.1	569.2	649.2	638.2	535.1	665.1	710.2	543.1	592.1	723.1
SZ5	444.1	381.1	585.1	407.1	561.1	382.1	387.2	467.1	456.1	353.1	483.1	528.2	361.1	410.1	541.1
SZ6	595.2	532.2	736.1	558.2	712.1	533.1	538.2	618.2	607.2	504.1	634.1	679.3	512.1	561.1	692.1
SZ7	695.1	632.1	836.1	658.1	812.1	633.1	638.2	718.1	707.1	604.1	734.1	779.2	612.1	661.1	792.1
SZ8	711.3	648.2	852.2	674.2	828.2	649.2	654.3	734.3	723.3	620.2	750.2	795.4	628.2	677.2	808.2
SZ9	790.2	727.2	931.1	753.2	907.2	728.2	733.2	813.2	802.2	699.1	829.2	874.3	707.1	756.1	887.2
SZ10	764.2	701.2	905.1	727.2	881.2	702.2	707.2	787.2	776.2	673.2	803.2	848.3	681.1	730.1	861.2
SZ11	562.2	499.2	703.2	525.2	679.2	500.2	505.2	585.2	574.2	471.2	601.2	646.3	479.2	528.1	659.2
SZ12	580.2	517.1	721.1	543.1	697.1	518.1	523.2	603.2	592.2	489.1	619.1	664.3	497.1	546.1	677.1
SZ13	581.1	518.1	722.1	544.1	698.1	519.1	524.2	604.1	593.2	490.1	620.1	665.2	498.1	547.1	678.1
SZ14	680.2	617.2	821.1	643.2	797.2	618.2	623.2	703.2	692.2	589.1	719.1	764.3	597.1	646.1	777.1
SZ15	737.2	674.2	878.1	700.2	854.1	675.1	680.2	760.2	749.2	646.1	776.1	821.3	654.1	703.1	834.1
SZ16	574.1	511.1	715.1	537.1	691.1	512.1	517.2	597.1	586.1	483.1	613.1	658.2	491.1	540.1	671.1
SZ17	715.2	652.2	856.2	678.2	832.2	653.2	658.3	738.2	727.2	624.2	754.2	799.3	632.2	681.2	812.2
SZ18	464.1	401.1	605.0	427.1	581.0	402.0	407.1	487.1	476.1	373.0	503.0	548.2	381.0	430.0	561.0
SZ19	460.1	397.1	601.1	423.1	577.1	398.1	403.2	483.1	472.1	369.1	499.1	544.2	377.1	426.1	557.1
SZ20	444.1	381.1	585.1	407.1	561.1	382.1	387.2	467.1	456.1	353.1	483.1	528.2	361.1	410.1	541.1
SZ21	523.2	460.2	664.1	486.2	640.1	461.1	466.2	546.2	535.2	432.1	562.1	607.3	440.1	489.1	620.1
SZ22	519.2	456.2	660.2	482.2	636.2	457.2	462.3	542.2	531.2	428.2	558.2	603.3	436.2	485.2	616.2
SZ23	513.2	450.2	654.1	476.2	630.2	451.2	456.2	536.2	525.2	422.1	552.2	597.3	430.1	479.1	610.2
SZ24	555.2	492.2	696.2	518.2	672.2	493.2	498.3	578.2	567.2	464.2	594.2	639.3	472.2	521.2	652.2
SZ25	600.2	537.2	741.2	563.2	717.2	538.2	543.3	623.2	612.2	509.2	639.2	684.3	517.2	566.2	697.2
SZ26	577.2	514.2	718.2	540.2	694.2	515.2	520.3	600.2	589.2	486.2	616.2	661.3	494.2	543.2	674.2
SZ27	649.2	586.2	790.2	612.2	766.2	587.2	592.3	672.2	661.3	558.2	688.2	733.3	566.2	615.2	746.2
SZ28	667.2	604.2	808.1	630.2	784.2	605.2	610.2	690.2	679.2	576.2	706.2	751.3	584.1	633.1	764.2
SZ29	613.1	550.1	754.0	576.1	730.1	551.1	556.1	636.1	625.1	522.0	652.0	697.2	530.0	579.0	710.0
SZ30	606.2	543.2	747.2	569.2	723.2	544.2	549.3	629.3	618.3	515.2	645.2	690.3	523.2	572.2	703.2
SZ31	729.2	666.2	870.2	692.2	846.2	667.2	672.2	752.2	741.2	638.2	768.2	813.3	646.2	695.2	826.2
SZ32	525.2	462.1	666.1	488.1	642.1	463.1	468.2	548.2	537.2	434.1	564.1	609.2	442.1	491.1	622.1
SZ33	564.2	501.1	705.1	527.1	681.1	502.1	507.2	587.2	576.2	473.1	603.1	648.2	481.1	530.1	661.1
SZ34	577.2	514.2	718.1	540.2	694.2	515.2	520.2	600.2	589.2	486.1	616.1	661.3	494.1	543.1	674.1
SZ35	598.2	535.2	739.1	561.2	715.1	536.1	541.2	621.2	610.2	507.1	637.1	682.3	515.1	564.1	695.1
SZ36	545.2	482.1	686.1	508.1	662.1	483.1	488.2	568.2	557.2	454.1	584.1	629.3	462.1	511.1	642.1
SZ37	562.1	499.1	703.1	525.1	679.1	500.1	505.2	585.1	574.1	471.1	601.1	646.2	479.1	528.1	659.1
SZ38	570.1	507.1	711.1	533.1	687.1	508.1	513.1	593.1	582.1	479.1	609.1	654.2	487.1	536.0	667.1

	Well 7					Well 8					Well 9				
	TA31	TA32	TA33	TA34	TA35	TA36	TA37	TA38	TA39	TA40	TA41	TA42	TA43	TA44	TA45
SZ1	680.3	569.2	506.2	537.2	532.2	620.1	764.4	533.3	550.2	599.3	552.2	769.2	614.2	555.2	599.2
SZ2	653.3	542.1	479.2	510.1	505.2	593.1	737.3	506.2	523.2	572.3	525.2	742.2	587.2	528.2	572.2
SZ3	563.2	452.1	389.1	420.1	415.1	503.0	647.3	416.2	433.1	482.2	435.1	652.1	497.1	438.1	482.1
SZ4	702.2	591.1	528.1	559.1	554.1	642.0	786.3	555.2	572.1	621.2	574.1	791.1	636.2	577.2	621.1
SZ5	520.2	409.1	346.1	377.1	372.1	460.0	604.3	373.2	390.1	439.2	392.1	609.1	454.1	395.1	439.1
SZ6	671.2	560.1	497.1	528.1	523.1	611.1	755.3	524.2	541.2	590.2	543.1	760.1	605.2	546.2	590.1
SZ7	771.2	660.1	597.1	628.1	623.1	711.0	855.3	624.2	641.1	690.2	643.1	860.1	705.1	646.1	690.1
SZ8	787.3	676.2	613.2	644.2	639.2	727.1	871.4	640.3	657.2	706.3	659.2	876.2	721.3	662.3	706.2
SZ9	866.2	755.1	692.1	723.1	718.1	806.1	950.3	719.2	736.2	785.2	738.1	955.2	800.2	741.2	785.1
SZ10	840.2	729.1	666.1	697.1	692.2	780.1	924.3	693.2	710.2	759.2	712.2	929.2	774.2	715.2	759.1
SZ11	638.2	527.1	464.1	495.1	490.2	578.1	722.3	491.2	508.2	557.2	510.2	727.2	572.2	513.2	557.1
SZ12	656.2	545.1	482.1	513.1	508.1	596.0	740.3	509.2	526.1	575.2	528.1	745.1	590.2	531.2	575.1
SZ13	657.2	546.1	483.1	514.1	509.1	597.0	741.3	510.2	527.1	576.2	529.1	746.1	591.1	532.1	576.1
SZ14	756.2	645.1	582.1	613.1	608.1	696.1	840.3	609.2	626.2	675.2	628.1	845.2	690.2	631.2	675.1
SZ15	813.2	702.1	639.1	670.1	665.1	753.1	897.3	666.2	683.2	732.2	685.1	902.1	747.2	688.2	732.1
SZ16	650.2	539.1	476.1	507.1	502.1	590.0	734.3	503.2	520.1	569.2	522.1	739.1	584.1	525.1	569.1
SZ17	791.3	680.2	617.2	648.2	643.2	731.1	875.4	644.3	661.2	710.3	663.2	880.2	725.2	666.2	710.2
SZ18	540.1	429.0	366.0	397.0	392.0	480.0	624.2	393.1	410.1	459.1	412.0	629.0	474.1	415.1	459.0
SZ19	536.2	425.0	362.1	393.1	388.1	476.0	620.3	389.1	406.1	455.2	408.1	625.1	470.1	411.1	455.1
SZ20	520.2	409.1	346.1	377.1	372.1	460.0	604.3	373.2	390.1	439.2	392.1	609.1	454.1	395.1	439.1
SZ21	599.2	488.1	425.1	456.1	451.1	539.1	683.3	452.2	469.2	518.2	471.1	688.1	533.2	474.2	518.1
SZ22	595.3	484.2	421.2	452.2	447.2	535.1	679.4	448.3	465.2	514.3	467.2	684.2	529.2	470.2	514.2
SZ23	589.2	478.1	415.1	446.1	441.2	529.1	673.3	442.2	459.2	508.2	461.1	678.2	523.2	464.2	508.1
SZ24	631.3	520.2	457.2	488.2	483.2	571.1	715.4	484.3	501.2	550.3	503.2	720.2	565.2	506.2	550.2
SZ25	676.3	565.1	502.2	533.1	528.2	616.1	760.3	529.2	546.2	595.3	548.2	765.2	610.2	551.2	595.2
SZ26	653.3	542.1	479.2	510.1	505.2	593.1	737.3	506.2	523.2	572.3	525.2	742.2	587.2	528.2	572.2
SZ27	725.3	614.2	551.2	582.2	577.2	665.1	809.4	578.3	595.2	644.3	597.2	814.2	659.2	600.2	644.2
SZ28	743.2	632.1	569.1	600.1	595.2	683.1	827.3	596.2	613.2	662.2	615.2	832.2	677.2	618.2	662.1
SZ29	689.1	578.0	515.0	546.0	541.0	629.0	773.2	542.1	559.1	608.1	561.0	778.1	623.1	564.1	608.0
SZ30	682.3	571.2	508.2	539.2	534.2	622.1	766.4	535.3	552.2	601.3	554.2	771.2	616.3	557.3	601.2
SZ31	805.2	694.1	631.1	662.1	657.2	745.1	889.3	658.2	675.2	724.2	677.2	894.2	739.2	680.2	724.2
SZ32	601.2	490.1	427.1	458.1	453.1	541.0	685.3	454.2	471.1	520.2	473.1	690.1	535.2	476.2	520.1
SZ33	640.2	529.1	466.1	497.1	492.1	580.0	724.3	493.2	510.1	559.2	512.1	729.1	574.2	515.2	559.1
SZ34	653.2	542.1	479.1	510.1	505.1	593.1	737.3	506.2	523.2	572.2	525.1	742.2	587.2	528.2	572.1
SZ35	674.2	563.1	500.1	531.1	526.1	614.1	758.3	527.2	544.2	593.2	546.1	763.1	608.2	549.2	593.1
SZ36	621.2	510.1	447.1	478.1	473.1	561.0	705.3	474.2	491.1	540.2	493.1	710.1	555.2	496.2	540.1
SZ37	638.1	527.0	464.1	495.0	490.1	578.0	722.2	491.1	508.1	557.1	510.1	727.1	572.1	513.1	557.1
SZ38	646.1	535.0	472.0	503.0	498.1	586.0	730.2	499.1	516.1	565.1	518.1	735.1	580.1	521.1	565.0

Figure S2. Molecular weights (MWs) of the 1710 possible acylsulfonamide combinations screened in a multi-fragment Kinetic Target-Guided Synthesis (KTGS) approach using 38 sulfonyl azides and 45 thio acids. The entire fragment library was divided into 9 incubation mixtures, each containing 5 thio acids and 38 sulfonyl azides.

2. LC-MS/MS Traces for Multi-Fragment KTGS with Mcl-1

Examples of LC-MS/MS traces of multi-fragment KTGS incubations with Mcl-1. Protein templation of KTGS fragments is assessed by comparing the LC-MS/MS traces of identical fragment combinations incubated with or without Mcl-1. Hit combinations contain increased amounts of acylsulfonamide products in incubations containing Mcl-1 when compared to those lacking Mcl-1. The identities of the KTGS hit combinations were further confirmed by comparing their peak retention times with the peak retention times of the corresponding synthesized acylsulfonamide products. Selected examples of such LC-MS/MS traces are shown below.

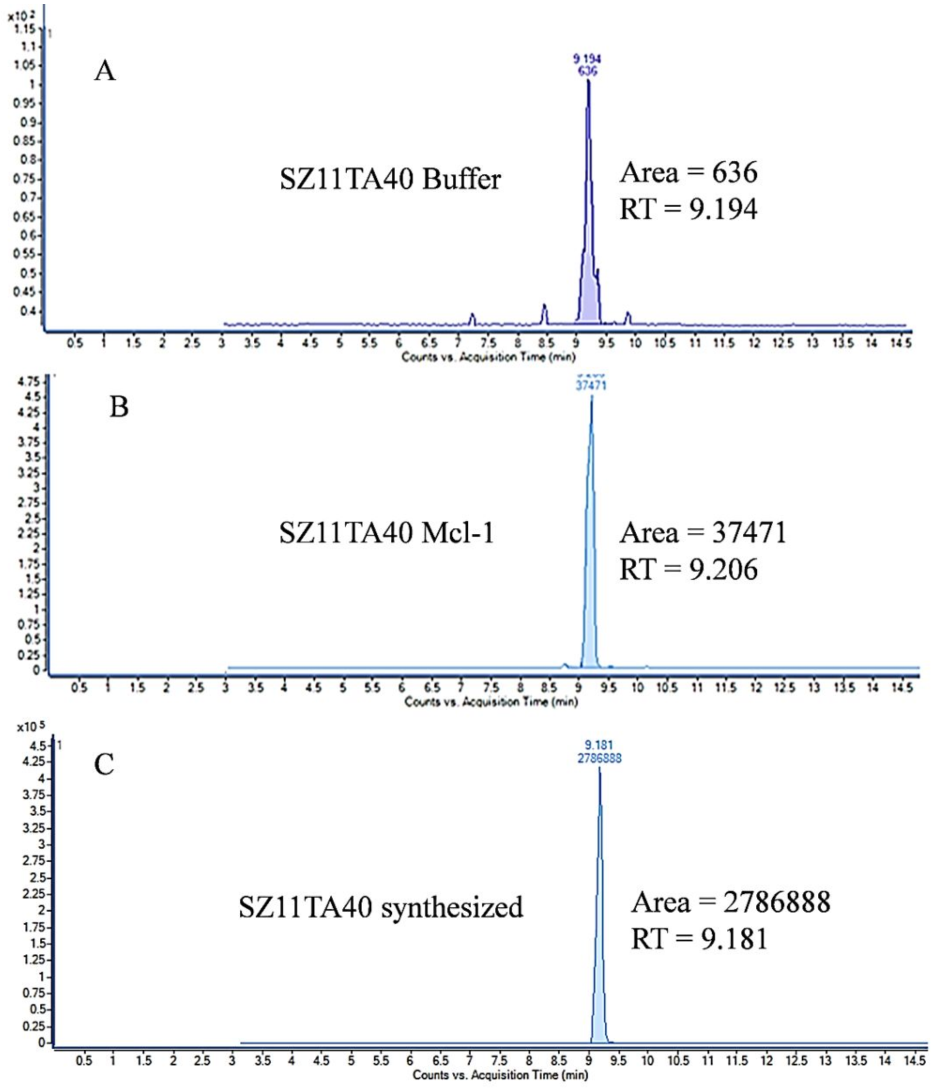

Figure S3. Identification of KTGS hit compound **SZ11TA40** by LC-MS/MS analysis of the KTGS incubation sample containing thio acids **TA36-40** and sulfonyl azides **SZ1-SZ38**. (A) The extracted LC-MS/MS trace of the hit compound **SZ11TA40** after incubation of thio acids **TA36-40** and sulfonyl azides **SZ1-SZ38** in the absence of Mcl-1. (B) The extracted LC-MS/MS trace of the hit compound **SZ11TA40** after incubation of thio acids **TA36-40** and sulfonyl azides **SZ1-SZ38** in the presence of Mcl-1 (10 µM). (C) LC-MS/MS trace of an authentic sample of **SZ11TA40** synthesized and characterized as described below in the experimental section.

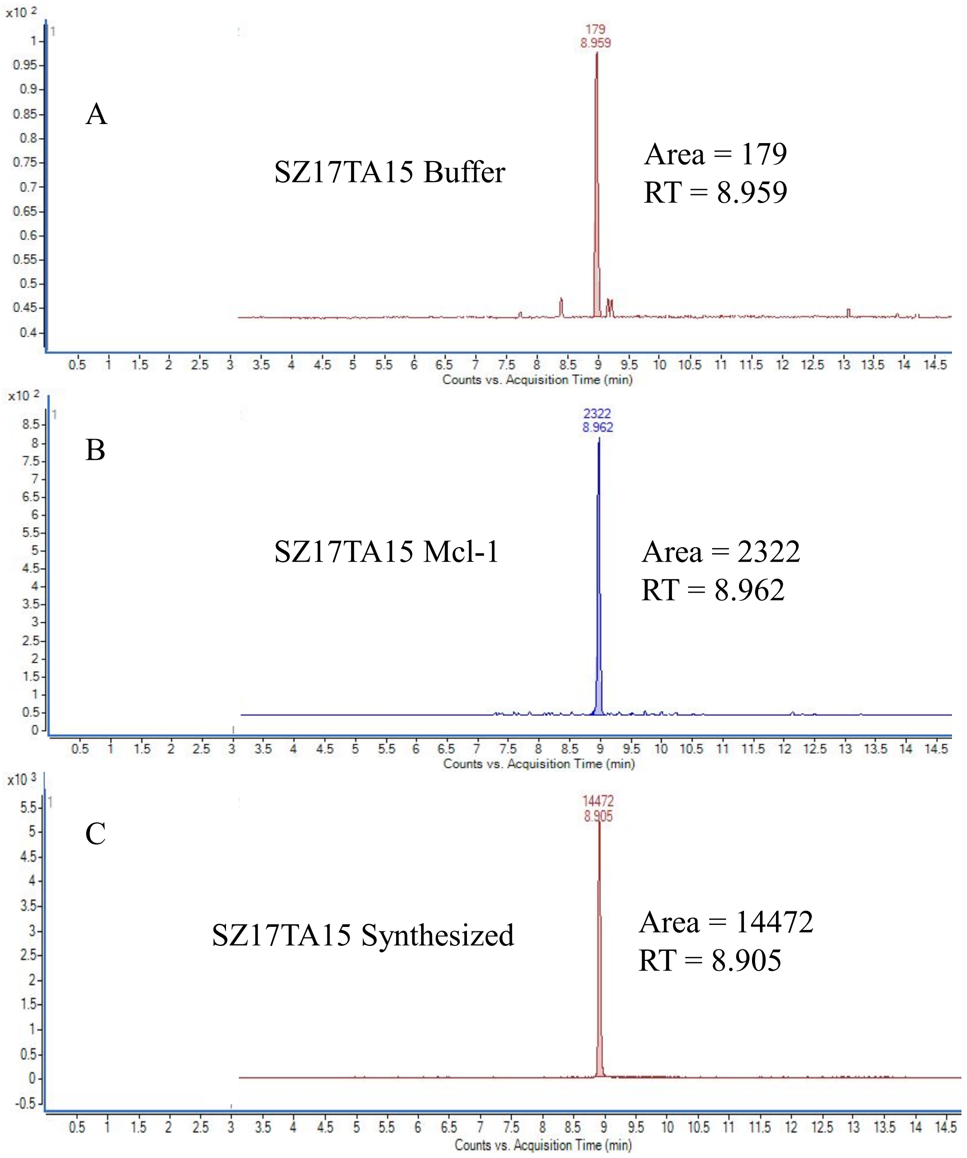

Figure S4. Identification of KTGS hit compound **SZ17TA15** by LC-MS/MS analysis of the KTGS incubation sample containing thio acids **TA11-15** and sulfonyl azides **SZ1-SZ38**. (A) The extracted LC-MS/MS trace of the hit compound **SZ17TA15** after incubation of thio acids **TA11-15** and sulfonyl azides **SZ1-SZ38** in the absence of Mcl-1. (B) The extracted LC-MS/MS trace of the hit compound **SZ11TA40** after incubation of thio acids **TA11-15** and sulfonyl azides **SZ1-SZ38** in the presence of Mcl-1 (10 μM). (C) LC-MS/MS trace of an authentic sample of **SZ17TA15** synthesized and characterized as described below in the experimental section.

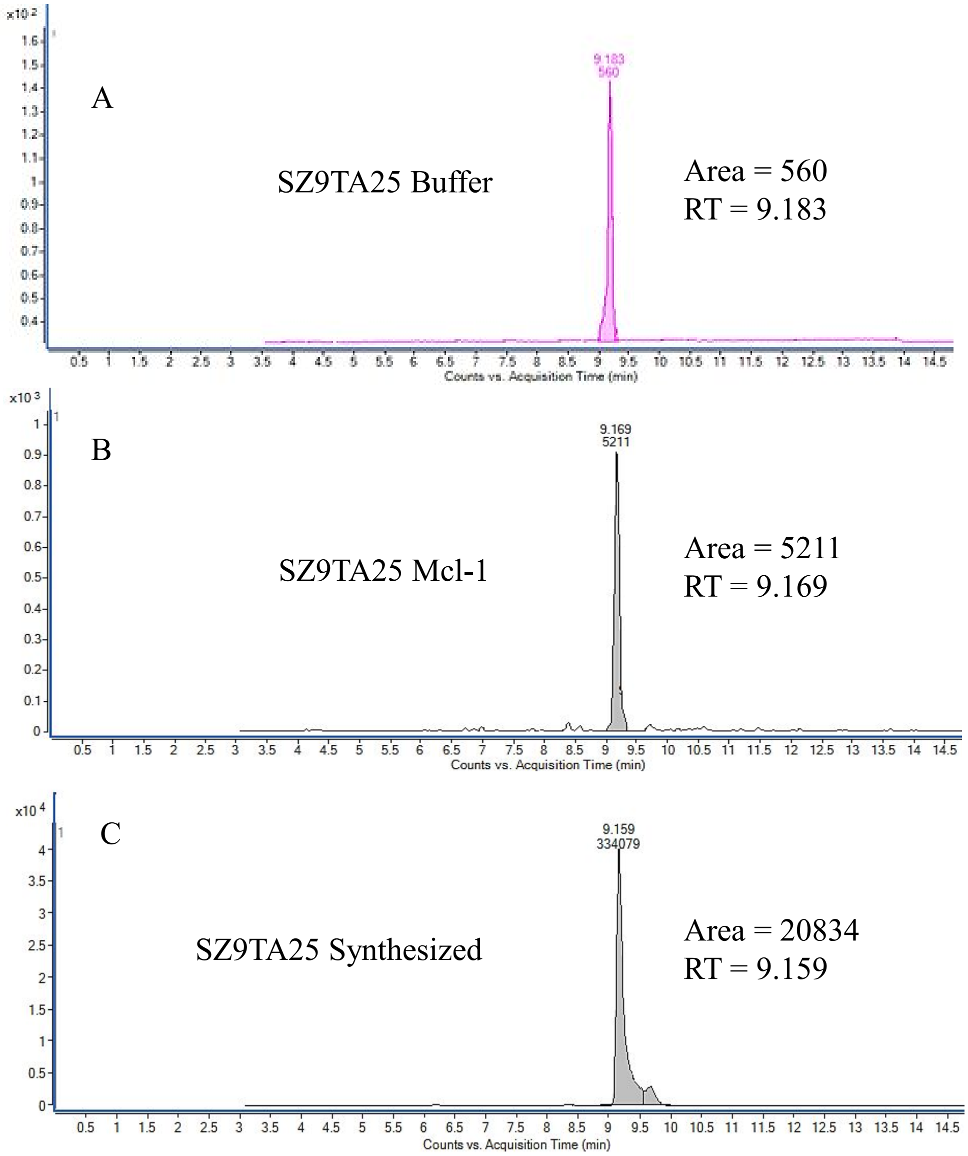

Figure S5. Identification of KTGS hit compound **SZ9TA25** by LC-MS/MS analysis of the KTGS incubation sample containing thio acids **TA21-25** and sulfonyl azides **SZ1-SZ38**. (A) The extracted LC-MS/MS trace of the hit compound **SZ9TA25** after incubation of thio acids **TA6-10** and sulfonyl azides **SZ1-SZ38** in the absence of Mcl-1. (B) The extracted LC-MS/MS trace of the hit compound after incubation of thio acids **TA21-25** and sulfonyl azides **SZ1-SZ38** in the presence of Mcl-1 (10 µM). (C) LC-MS/MS trace of an authentic sample of **SZ9TA25** synthesized and characterized as described below in the experimental section.

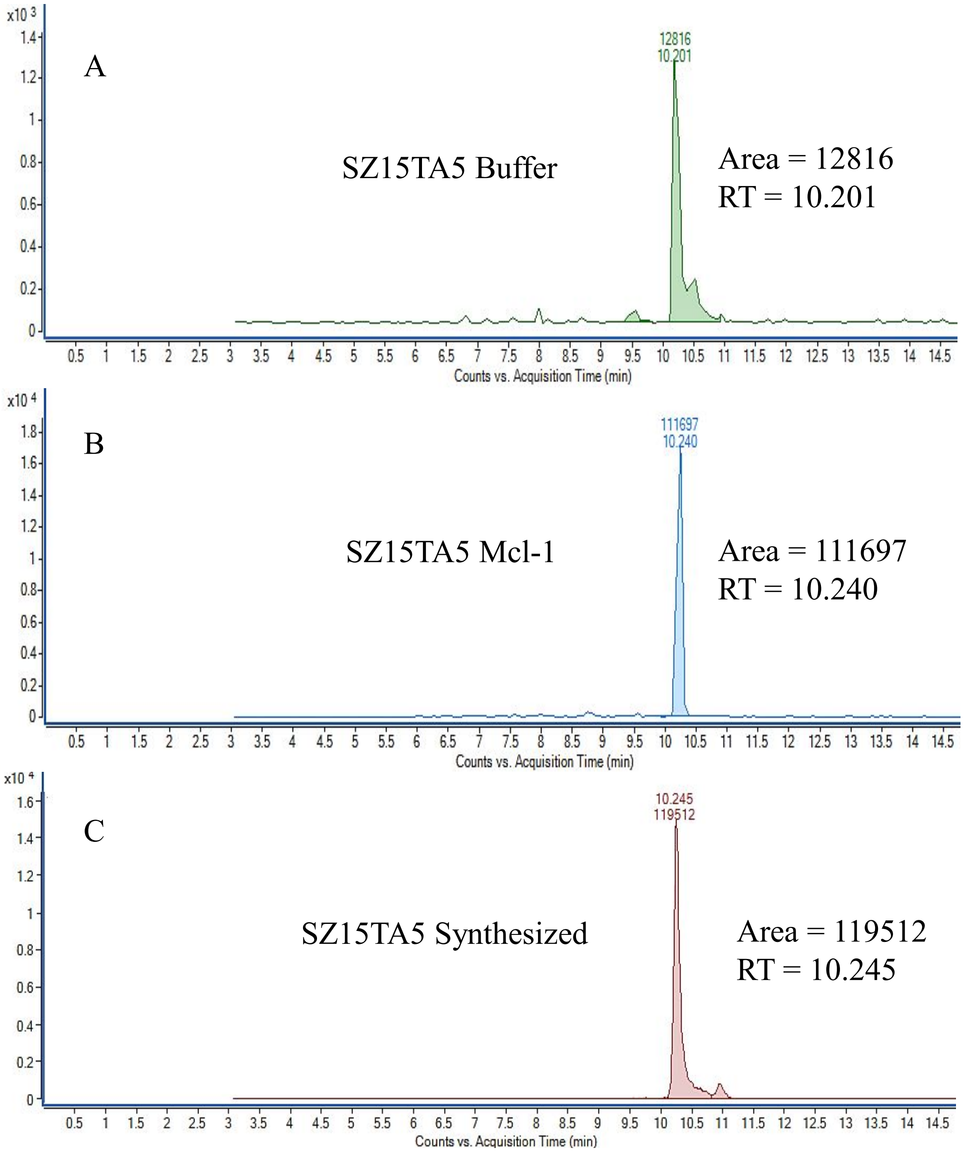

Figure S6. Identification of KTGS hit compound **SZ15TA5** by LC-MS/MS analysis of the KTGS incubation sample containing thio acids **TA1-5** and sulfonyl azides **SZ1**-**SZ38**. (A) The extracted LC-MS/MS trace of the hit compound **SZ15TA5** after incubation of thio acids **TA1-5** and sulfonyl azides **SZ1**-**SZ38** in the absence of Mcl-1. (B) The extracted LC-MS/MS trace of the hit compound after incubation of thio acids **TA1-5** and sulfonyl azides **SZ1**-**SZ38** in the presence of Mcl-1 (10 μM). (C) LC-MS/MS trace of an authentic sample of **SZ15TA5** synthesized and characterized as described below in the experimental section.

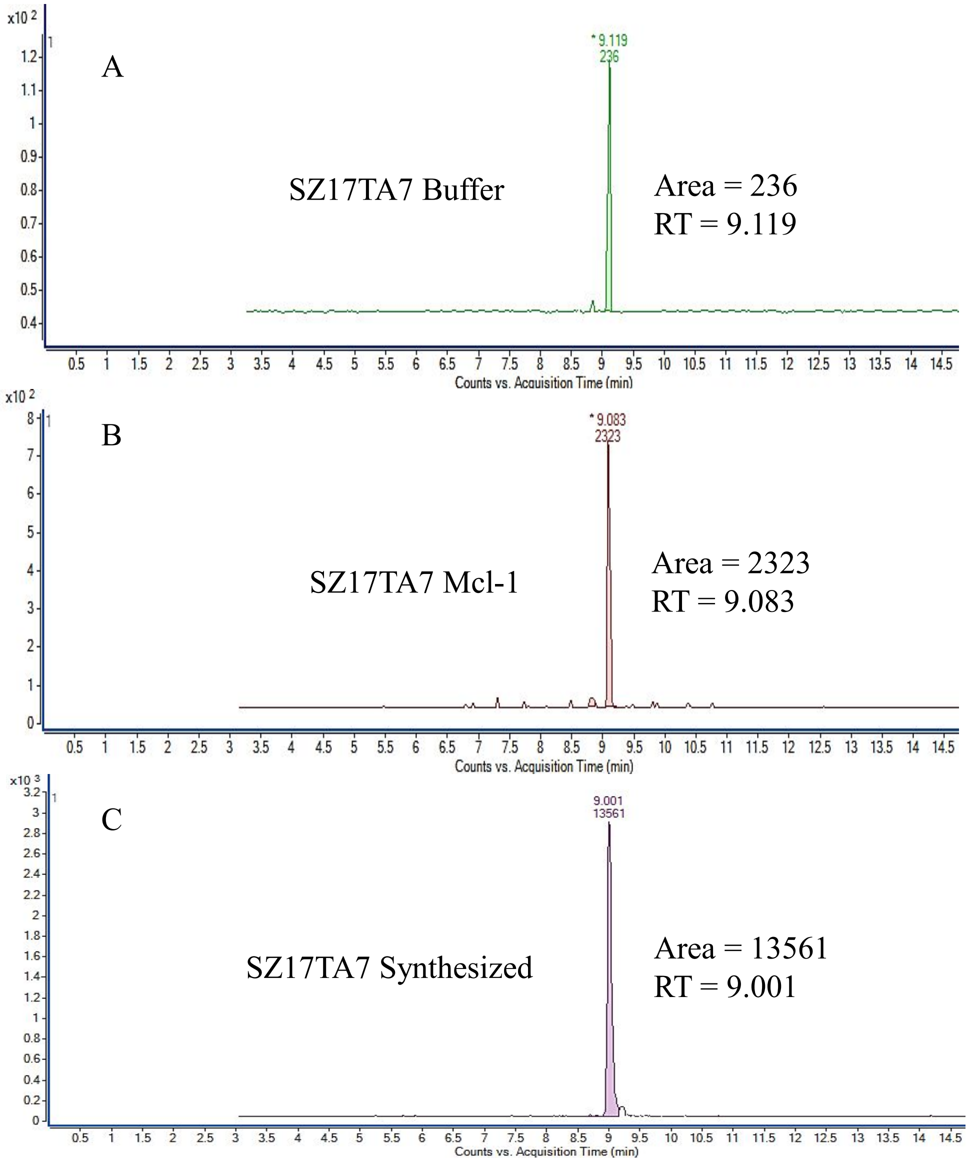

Figure S7. Identification of KTGS hit compound **SZ17TA7** by LC-MS/MS analysis of the KTGS incubation sample containing thio acids **TA6-10** and sulfonyl azides **SZ1-SZ38**. (A) The extracted LC-MS/MS trace of the hit compound **SZ17TA7** after incubation of thio acids **TA6-10** and sulfonyl azides **SZ1-SZ38** in the absence of Mcl-1. (B) The extracted LC-MS/MS trace of the hit compound after incubation of thio acids **TA6-10** and sulfonyl azides **SZ1-SZ38** in the presence of Mcl-1 (10 μM). (C) LC/MS-MS trace of an authentic sample of **SZ17TA7** synthesized and characterized as described below in the experimental section.

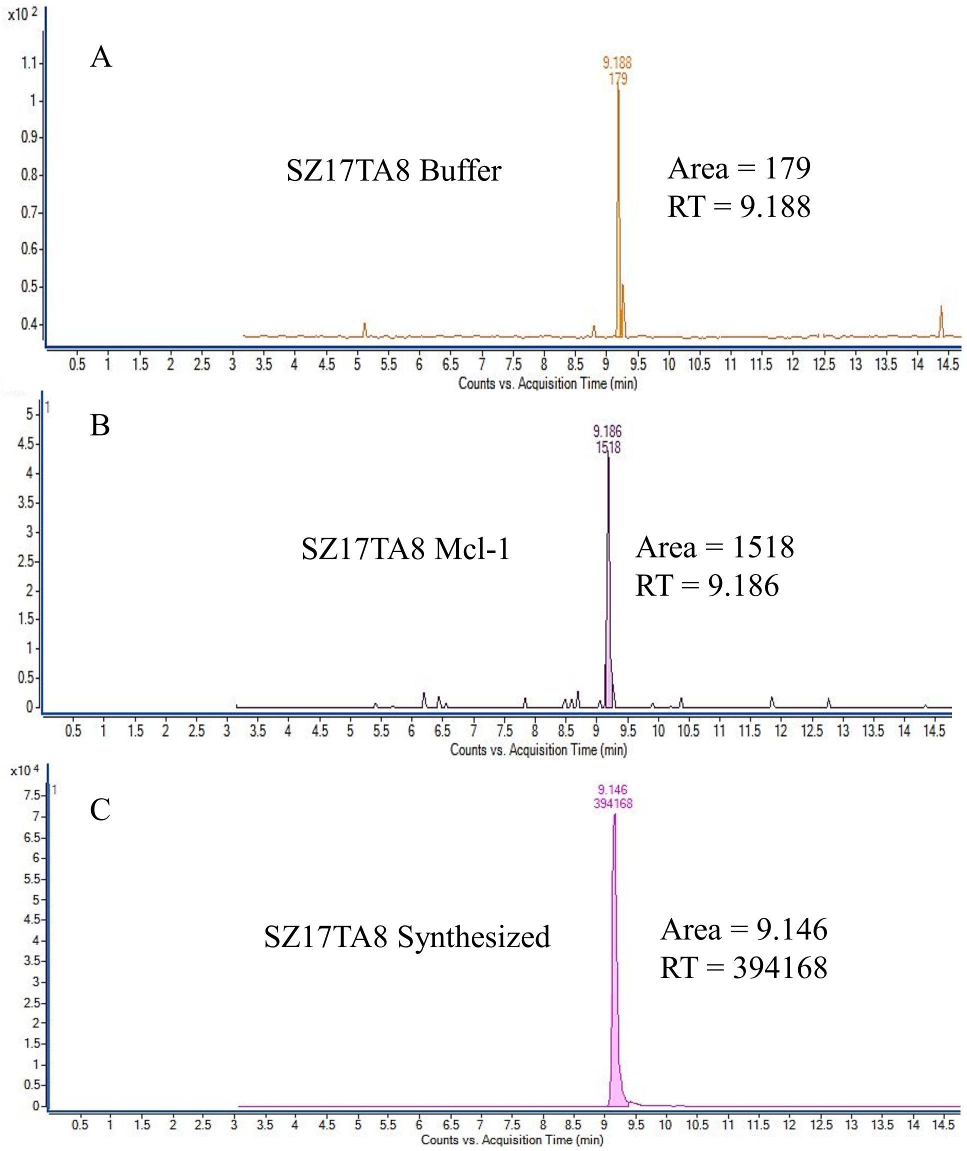

Figure S8. Identification of KTGS hit compound **SZ17TA8** by LC-MS/MS analysis of the KTGS incubation sample containing thio acids **TA6-10** and sulfonyl azides **SZ1-SZ38**. (A) The extracted LC-MS/MS trace of the hit compound **SZ17TA8** after incubation of thio acids **TA6-10** and sulfonyl azides **SZ1-SZ38** in the absence of Mcl-1. (B) The extracted LC-MS/MS trace of the hit compound after incubation of thio acids **TA6-10** and sulfonyl azides **SZ1-SZ38** in the presence of Mcl-1 (10 μM). (C) LC-MS/MS trace of an authentic sample of **SZ17TA8** synthesized and characterized as described below in the experimental section.

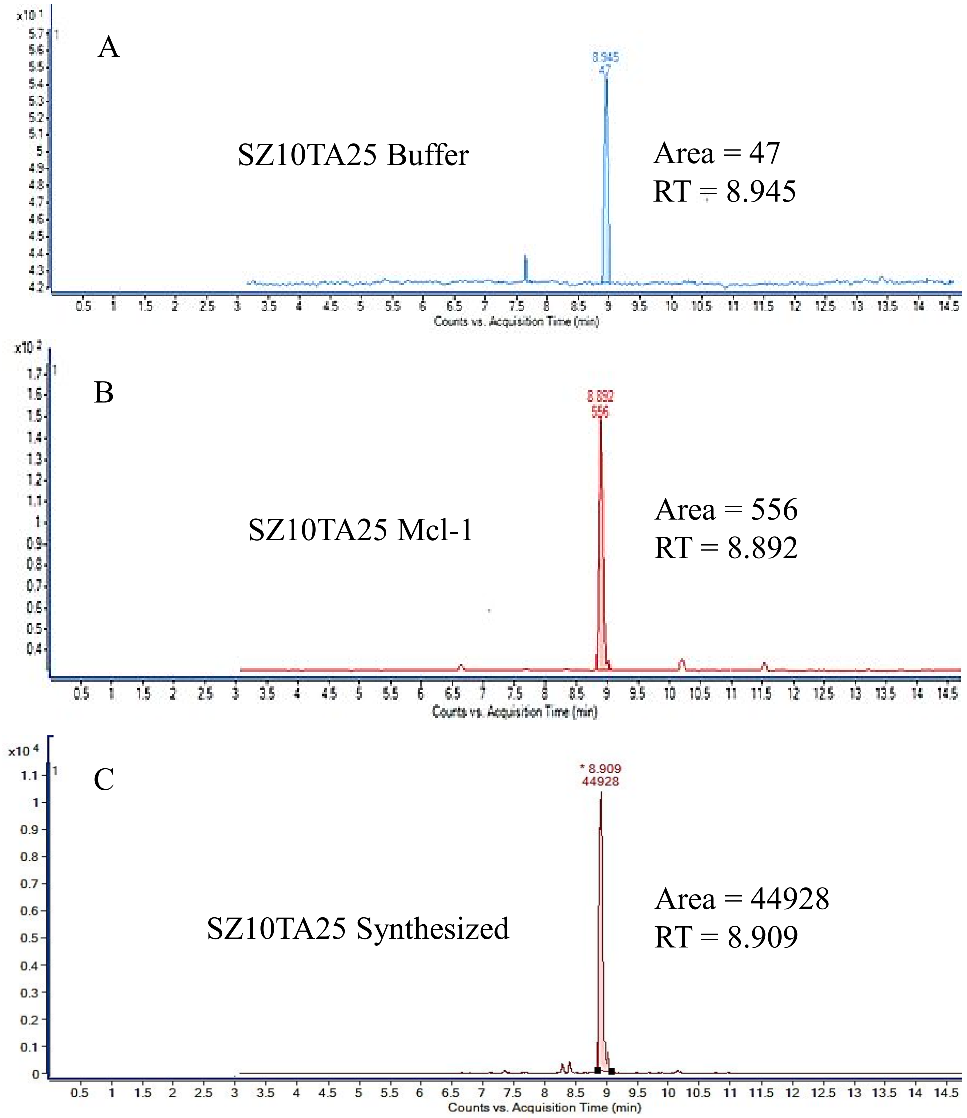

Figure S9. Identification of KTGS hit compound **SZ10TA25** by LC-MS/MS analysis of the KTGS incubation sample containing thio acids **TA21-25** and sulfonyl azides **SZ1-SZ38**. (A) The extracted LC-MS/MS trace of the hit compound **SZ10TA25** after incubation of thio acids **TA21-25** and sulfonyl azides **SZ1-SZ38** in the absence of Mcl-1. (B) The extracted LC-MS/MS trace of the hit compound after incubation of thio acids **TA21-25** and sulfonyl azides **SZ1-SZ38** in the presence of Mcl-1 (10 μM). (C) LC-MS/MS trace of an authentic sample of **SZ10TA25** synthesized and characterized as described below in the experimental section.

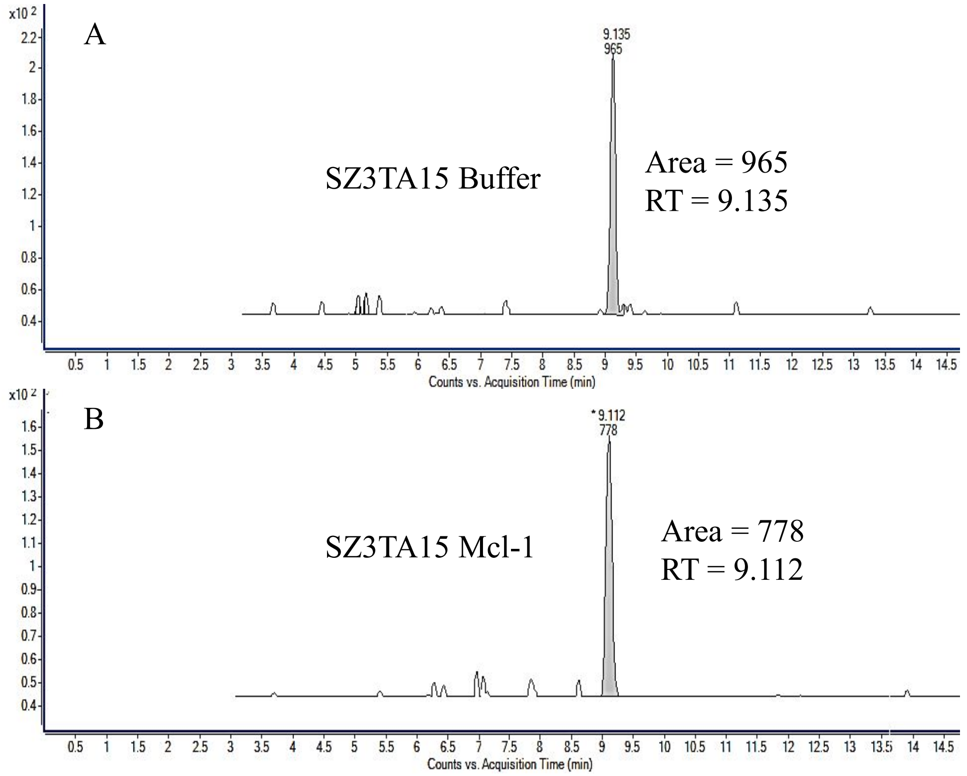

Figure S10. Example of a non-hit combination, **SZ3TA15**, by LC-MS/MS analysis of the KTGS incubation sample containing thio acids **TA11-15** and sulfonyl azides **SZ1**-**SZ38**. (A) The extracted LC-MS/MS trace of compound **SZ3TA15** after incubation of thio acids **TA11-15** and sulfonyl azides **SZ1**-**SZ38** in the absence of Mcl-1. (B) The extracted LC-MS/MS trace of compound **SZ3TA15** after incubation of thio acids **TA11-15** and sulfonyl azides **SZ1**-**SZ38** in the presence of Mcl-1 (10 μM)

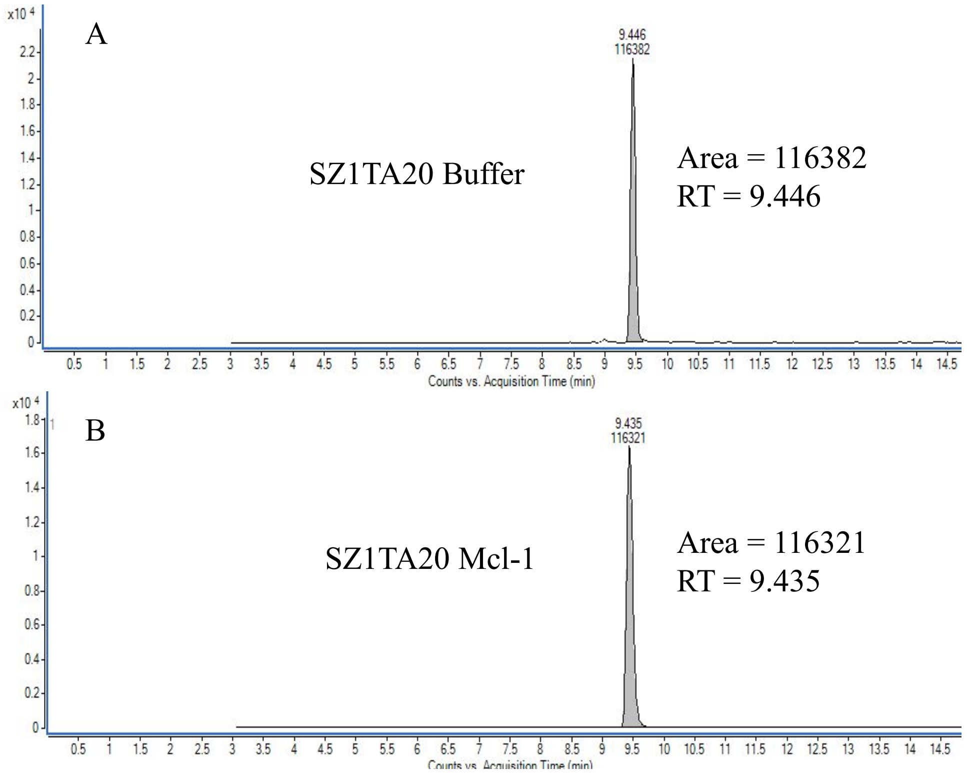

Figure S11. Example of a non-hit combination **SZ1TA20** by LC-MS/MS analysis of the KTGS incubation sample containing thio acids **TA16-20** and sulfonyl azides **SZ1-SZ38**. (A) The extracted LC-MS/MS trace of compound **SZ1TA20** after incubation of thio acids **TA16-20** and sulfonyl azides **SZ1-SZ38** in the absence of Mcl-1. (B) The extracted LC-MS/MS trace of compound **SZ1TA20** after incubation of thio acids **TA16-20** and sulfonyl azides **SZ1-SZ38** in the presence of Mcl-1 (10 μM).

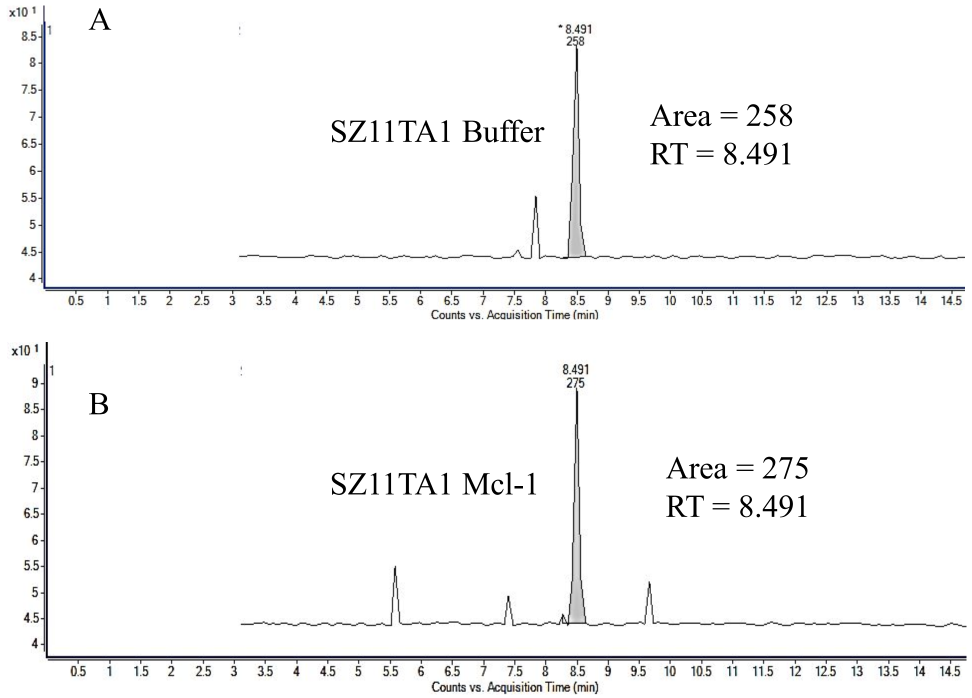

Figure S12. Example of non-hit compound, **SZ11TA1**, by LC-MS/MS analysis of the KTGS incubation sample containing thio acids **TA1-5** and sulfonyl azides **SZ1-SZ38**. (A) The extracted LC-MS/MS trace of compound **SZ11TA1** after incubation of thio acids **TA1-5** and sulfonyl azides **SZ1-SZ38** in the absence of Mcl-1. (B) The extracted LC-MS/MS trace of compound **SZ11TA1** after incubation of thio acids **TA1-5** and sulfonyl azides **SZ1-SZ38** in the presence of Mcl-1 (10 μM).

5. Clustering and Docking

Clustering. Murcko scaffolds of all 51 KTGS hits were generated using a workflow in KNIME. The corresponding structures were then clustered in StarDrop (Optibrium) using 2-dimenstional path-based fingerprints and a minimum Tanimoto coefficient threshold of 0.8. The aforementioned process generated 11 clusters and 8 singletons (**see Mcl-1_KTGS_Hits_Clusters.xlsx**).

Docking. Docking studies were performed using the co-crystal structure of Mcl-1 and compound **60** (PDB ID: **5**FDR) to predict the binding modes of identified KTGS hits.[1] The original co- crystal structure was refined using the Protein Preparation Wizard implemented in the Maestro 11.1 (Schrödinger Release 2017-2) interface, and invalid atom types were corrected using this same wizard. A receptor grid was generated from the refined structure using default values. The docked models for compound 60 were in good agreement with the reported crystal structures coordinates (see Fig 5B). KTGS hits were docked into the grid using Glide 7.438 in standard precision (SP) mode, without any constraints. The proposed binding pose of **SZ17TA3** within the Mcl-1 active site is shown in Fig. 5C.

Table S3. Amplification coefficients and initial FP data of 51 KTGS hits identified in a screen against Mcl-1.

Compound	Amplification coefficient 5TA x 38SZ	Amplification coefficient 1TA x 1SZ	% Inhibition (50 μM) Mcl-1
**SZ9TA17**	22	18	>99
**SZ11TA40**	8	5.5	>99
**SZ17TA3**	25	12	>99
**SZ31TA3**	15	7	>99
**SZ15TA5**	9	4	>99
**SZ17TA8**	8	4	>99
**SZ31TA8**	7	4	>99
**SZ9TA5**	8	4.5	99
**SZ15TA7**	8	9	98.4
**SZ16TA44**	12	4	98.1
**SZ9TA1**	14	4	97.3
**SZ15TA8**	7	6	96.5
**SZ15TA3**	10	5	95.9
**SZ9TA7**	23	6	94.1
**SZ10TA25**	12	5	93.6
**SZ17TA25**	7	4	93.6
**SZ14TA40**	24	15	93.1
**SZ15TA1**	13	5	92.5
**SZ32TA42**	10	6	91
**SZ15TA17**	7	7	89.5
**SZ12TA42**	9	6	89.4
**SZ35TA17**	9	9	89.2
**SZ15TA25**	15	4.5	89
**SZ35TA7**	25	4	87.8
**SZ17TA7**	10	12	87.3
**SZ31TA24**	7	15	82.4
**SZ31TA15**	72	6	80.7
**SZ4TA30**	20	4	80.7
**SZ35TA24**	9	8	76.4
**SZ17TA24**	9	5	74.1
**SZ15TA24**	20	15	70.8
**SZ10TA34**	7	6	64.1
**SZ11TA30**	12	7	63.6
**SZ10TA44**	12	9	63.4
**SZ17TA15**	13	4	60.6
**SZ31TA14**	10	4	59.5
**SZ15TA14**	7	6	58.8
**SZ12TA2**	7	4	54.1
**SZ28TA45**	8	5	54
**SZ15TA15**	9	10	53.6
**SZ9TA25**	15	8	47.6
**SZ4TA17**	8	10	44.7
**SZ21TA23**	7	4	42.8
**SZ17TA31**	21	9	42.5
**SZ31TA45**	25	6	40.2
**SZ10TA15**	10	8	37.4
**SZ31TA44**	10	12	36.5
**SZ17TA45**	16	20	34.5
**SZ8TA20**	20	10	19.2
**SZ10TA41**	20	40	12.4
**SZ31TA40**	56	8	-52.4

NA = Not Available
ND = Not Detected

6. Synthetic Procedures and Compound Characterization

Synthesis of Reactive Building Blocks and Acylsulfonamides

General Information. All reagents and solvents were obtained from Sigma-Aldrich, Oakwood Products, Inc. or TCI America and used without further purification. Analytical thin layer chromatography (TLC) was performed on silica gel 60 F254 pre-coated plates (0.25 mm) from EMD Chemical Inc. and components were visualized by ultraviolet light (254 nm) and TLC staining solutions (phosphomolybdic acid (PMA), $KMnO_4$ solution, and/or a solution of $Ce(SO_4)_2$/ammonium phosphomolybdate/10% H_2SO_4 followed by heating). Reported R_f values were determined for TLC. EMD silica gel 60 (particle size 40-63 μm) 230 – 400 mesh was used for column chromatography. ^{1}H-NMR spectra were recorded at ambient temperature on a 250 MHz Bruker, 400 MHz Varian, 500 MHz Varian or 600 MHz Varian NMR spectrometer in the indicated solvent. All ^{1}H NMR experiments are reported in δ units, parts per million (ppm) downfield of TMS and were measured relative to the signals for chloroform (7.26 ppm), methanol (3.31 ppm) and dimethylsulfoxide (2.50 ppm). ^{13}C-NMR spectra were recorded at ambient temperature at 62.5 MHz, 100 MHz Varian, 125 MHz or 150 MHz in the solvent indicated. All ^{13}C NMR spectra are reported in ppm relative to the signals for chloroform (77.16 ppm), methanol (49 ppm) or dimethylsulfoxide (39.5 ppm) with ^{1}H decoupled observation. Data for ^{1}H NMR are reported as follows: chemical shift (δ ppm), multiplicity (s = singlet, d = doublet, t = triplet, q = quartet, p = pentet, sext = sextet, sept = septet, oct = octet, m = multiplet), integration and coupling constant (Hz), whereas ^{13}C NMR analyses are reported in terms of chemical shift. NMR data was analyzed by using MestReNova Software ver. 6.0.2-5475. The purity of the final compounds was determined to be ≥ 95% by high pressure liquid chromatography (HPLC) using an Agilent 1100 LC instrument coupled to an Agilent G1946D MSD-VL instrument with electrospray ionization. Low resolution mass spectra were acquired on an Agilent G1946D MSD-VL with electrospray ionization, whereas high resolution mass spectra (HRMS) were acquired on an Agilent 6540 LC/MSD TOF system.

Synthesis of Key Intermediates Required for the Preparation of Reactive Fragments

Key Compounds **1-9** are intermediates that are common to numerous thioesters, carboxylic acids, sulfonamides and sulfonyl azides throughout this section. Their synthesis is as follows.

Synthesis of 4-(bromomethyl)benzene-1-sulfonyl azide (1)and 4-(bromomethyl)benzene-1-sulfonamide (2)[2]

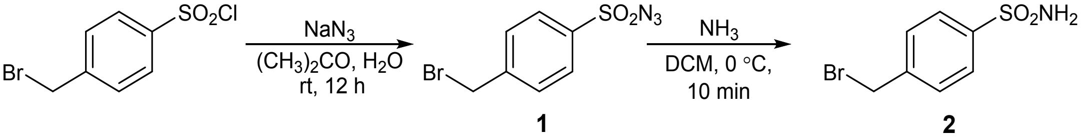

Synthesis of 2-(phenylsulfanyl)ethylamine (3)[3]

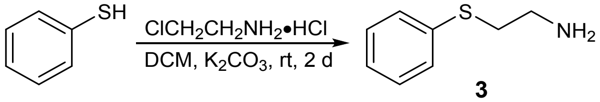

3

Synthesis of (9H-fluoren-9-yl)methanethiol (FmSH) (4)[4]

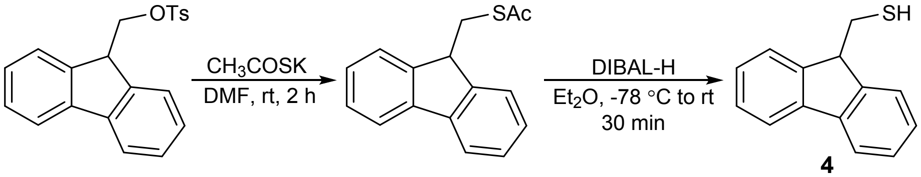

4

Synthesis of 6,7-dimethoxy-1-phenyl-1,2,3,4-tetrahydroisoquinoline (5)[5]

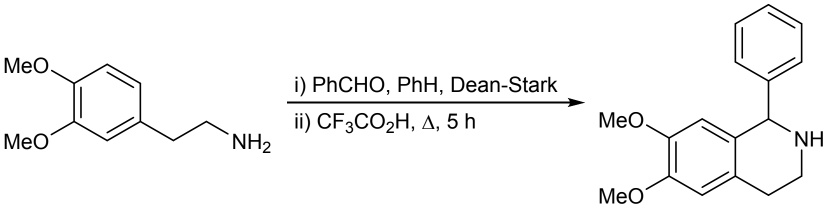

5

Synthesis of 3,4-bis(2,4-difluorophenyl)-1H-pyrrole-2,5-dione (6)[6]

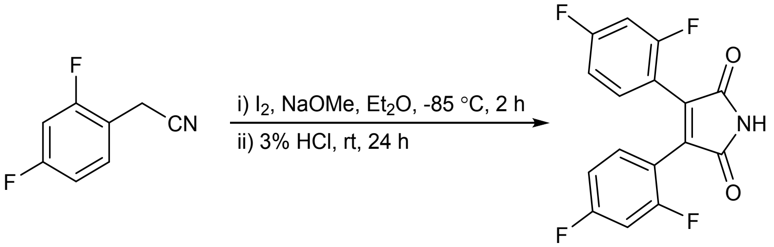

6

Synthesis of 5-bromo-1-methyl-1H-indole-3-carbaldehyde (7)[7]

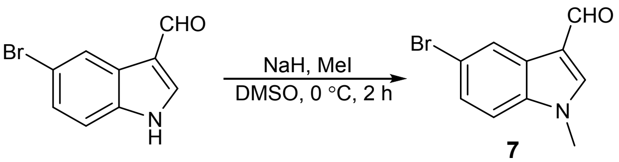

7

Synthesis of 1-isopropyl-1H-indole (8)[8]

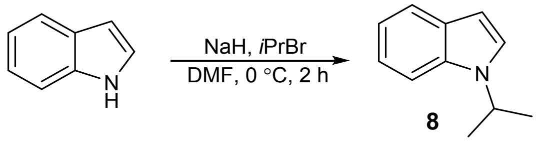

Synthesis of methyl 1-methyl-1H-indole-5-carboxylate (9)[9]

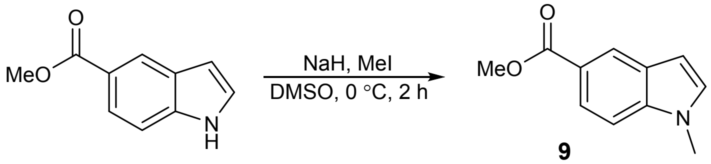

The protocol for the synthesis of **9** is identical to that carried out for the synthesis of **7**. **Methyl 1-methyl-1H-indole-5-carboxylate:** $R_f = 0.49$ in hexanes: EtOAc = 2:1. ^{1}H NMR (400 MHz, $CDCl_3$) δ ppm 8.4 (s, 1H) 7.9 (d, $J = 8.6$ Hz, 1H) 7.3 (d, $J = 9.4$ Hz, 1H) 7.1 (d, $J = 3.1$ Hz, 1H) 6.6 (d, $J = 3.9$ Hz, 1H) 3.9 (s, 3H) 3.8 (s, 3H). ^{13}C NMR (101 MHz, $CDCl_3$) δ ppm 168.2, 139.0, 130.2, 127.9, 123.9, 122.8, 121.3, 108.8, 102.6, 51.7, 32.9.

Synthesis of Thioesters

Compounds **TE1-TE45** are thioesters that were used in this study for the sulfo-click reactions. The synthesis of these compounds and their intermediates are as follows. Thioesters **TE1-TE5, TE7, TE8, TE11, TE13** have been previously reported. All thioesters were synthesized from the corresponding carboxylic acids (**CA**s) following the literature procedure.[10] Products were purified using flash column chromatography or recrystallization. Many of the CA compounds are commercially available or their syntheses have been previously reported.

S-((9H-fluoren-9-yl)methyl) benzothioate (TE1)

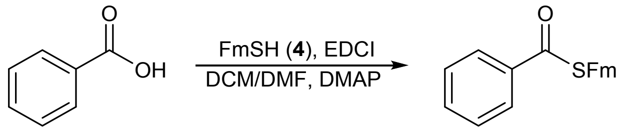

CA1 **TE1**

TE1 was synthesized from **CA1** (commercially available) and thiol **4** as previously described.[11]

S-((9H-fluoren-9-yl)methyl) 4-(4,4-dimethylpiperidin-1-yl)benzothioate (TE2)

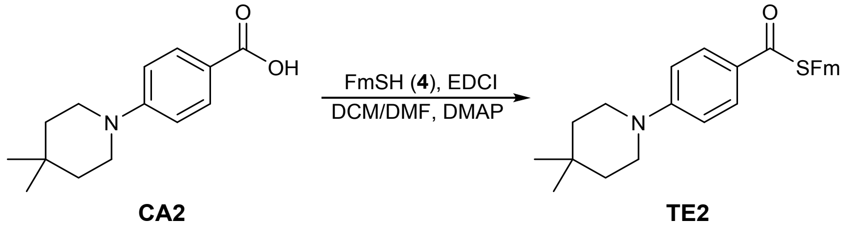

TE2 was synthesized from **CA2** (commercially available) and thiol **4** as previously described.[11]

S-((9H-fluoren-9-yl)methyl) 4-methyl-2-phenylthiazole-5-carbothioate (TE3)

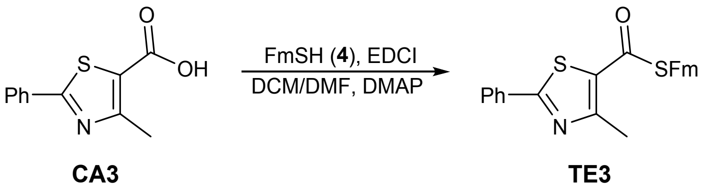

TE3 was synthesized from **CA3** (commercially available) and thiol **4** as previously described.[11]

S-((9H-fluoren-9-yl)methyl) 2-(6,7-dimethoxy-1-phenyl-3,4-dihydroisoquinolin-2(1H) yl)ethanethioate (TE4)

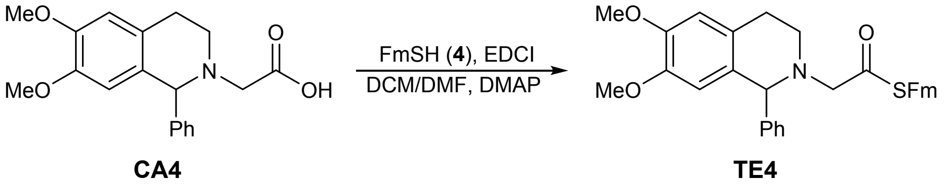

TE4 was synthesized from **CA4**[12] and thiol **4** as previously described.[11]

S-((9H-fluoren-9-yl)methyl) 3,5-dimethoxybenzothioate (TE5)

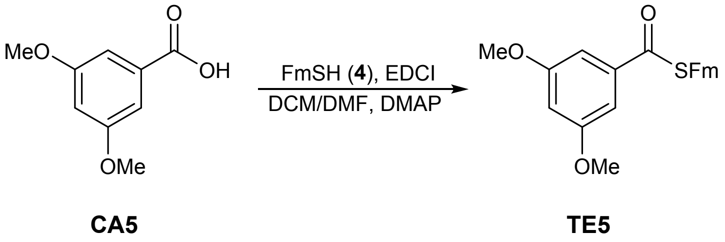

TE5 was synthesized from **CA5** (commercially available) and thiol **4** as previously described.[11]

S-((9H-fluoren-9-yl)methyl) 3-nitrobenzothioate (TE6)

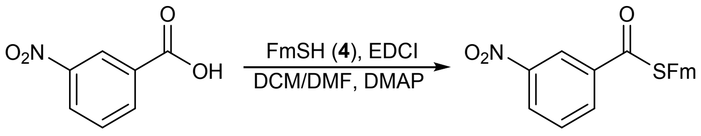

CA6 **TE6**

TE6 was synthesized from **CA6** (commercially available) and thiol **4** using the literature procedure.[10] Yield = 80%. R_f = 0.71 in hexanes : EtOAc = 3:1. ^{1}H NMR (400 MHz, $CDCl_3$) δ 8.70 (t, *J* = 1.9 Hz, 1H), 8.36 (ddd, *J* = 8.2, 2.2, 1.0 Hz, 1H), 8.19 – 8.15 (m, 1H), 7.76 (d, *J* = 7.4 Hz, 2H), 7.71 (d, *J* = 7.5 Hz, 2H), 7.58 (t, *J* = 8.0 Hz, 1H), 7.41 (t, *J* = 7.2 Hz, 2H), 7.38 – 7.32 (m, 2H), 4.29 (t, *J* = 5.7 Hz, 1H), 3.78 (d, *J* = 5.8 Hz, 2H). ^{13}C NMR (101 MHz, $CDCl_3$) δ 189.7, 148.3, 145.1, 141.2, 138.2, 132.7, 129.8, 128.0, 127.5, 127.3, 124.7, 122.2, 120.1, 46.5, 33.0. HRMS (ESI) calcd for $C_{21}H_{15}NO_3S$ $[M+H]^+$: 362.0845, found: 362.0848.

S-((9H-fluoren-9-yl)methyl) naphthalene-1-carbothioate (TE7)

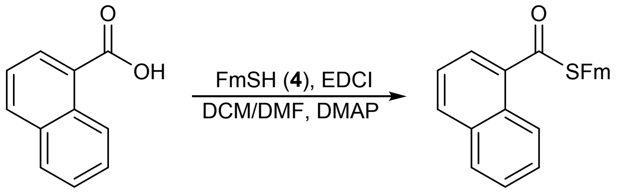

CA7 **TE7**

TE7 was synthesized from **CA7** (commercially available) and thiol **4** as previously described.[11]

S-((9H-fluoren-9-yl)methyl) 4-(trifluoromethoxy)benzothioate (TE8)

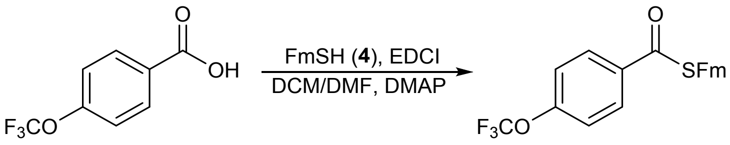

CA8 **TE8**

TE8 was synthesized from **CA8** (commercially available) and thiol **4** as previously described.[11]

S-((9H-fluoren-9-yl)methyl) furan-2-carbothioate (TE9)

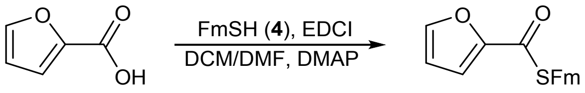

CA9 **TE9**

TE9 was synthesized from **CA9** (commercially available) and thiol **4** using the literature procedure.[10] Yield = 84%. R_f = 0.75 in hexanes : EtOAc = 2:1. ^{1}H NMR (400 MHz, $CDCl_3$) δ 7.78 – 7.71 (m, 4H), 7.55 – 7.54 (m, 1H), 7.44 – 7.31 (m, 4H), 7.18 – 7.16 (m, 1H), 6.50 (dd, *J* = 3.6, 1.7 Hz, 1H), 4.26 (t, *J* = 6.0 Hz, 1H), 3.68 (d, *J* = 6.0 Hz, 2H). ^{13}C NMR (101 MHz, $CDCl_3$) δ 180.3, 150.9, 146.3, 145.6, 141.2, 127.9, 127.3, 124.8, 120.0, 115.7, 112.3, 46.9, 31.7. HRMS (ESI) calcd for $C_{19}H_{14}O_2S$ $[M+Na]^+$: 329.0607, found: 329.0611.

S-((9H-fluoren-9-yl)methyl) isoxazole-5-carbothioate (TE10)

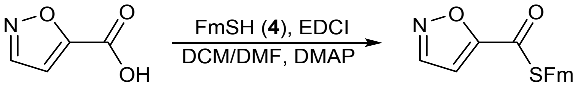

CA10 **TE10**

TE10 was synthesized from **CA10** (commercially available) and thiol **4** using the literature procedure.[10] Yield = 75%. R_f = 0.63 in hexanes : EtOAc = 3:1. ^{1}H NMR (400 MHz, $CDCl_3$) δ 8.32 – 8.29 (m, 1H), 7.76 (d, *J* = 7.6 Hz, 2H), 7.68 (d, *J* = 7.5 Hz, 2H), 7.44 – 7.39 (m, 2H), 7.37 – 7.31 (m, 2H), 6.84 (m, 1H), 4.27 (t, *J* = 5.7 Hz, 1H), 3.74 (d, *J* = 5.8 Hz, 2H). ^{13}C NMR (101 MHz, $CDCl_3$) δ 179.6, 164.4, 150.7, 144.9, 141.1, 128.0, 127.3, 124.6, 120.1, 105.9, 46.3, 32.2. HRMS (ESI) calcd for $C_{18}H_{13}NO_2S$ $[M+Na]^+$: 330.0559, found: 330.0558.

S-((9H-fluoren-9-yl)methyl) 2,2-diphenylethanethioate (TE11)

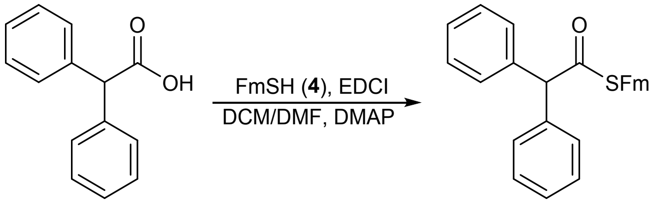

CA11 **TE11**

TE11 was synthesized from **CA11** (commercially available) and thiol **4** as previously described.[11]

S-((9H-fluoren-9-yl)methyl) 2-(1H-indol-3-yl)ethanethioate (TE12)

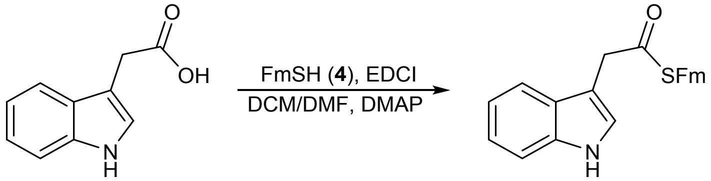

CA12 **TE12**

TE12 was synthesized from **CA12** (commercially available) and thiol **4** using the literature procedure.[10] ^{1}H NMR (500 MHz, $CDCl_3$) δ 8.10 (s, 1H), 7.75 (d, *J* = 7.6 Hz, 2H), 7.61 (d, *J* = 7.5 Hz, 2H), 7.56 (d, *J* = 7.9 Hz, 1H), 7.42 – 7.35 (m, 3H), 7.31 – 7.26 (m, 2H), 7.23 (t, *J* = 7.5 Hz, 1H), 7.16 (t, *J* = 7.4 Hz, 1H), 7.07 (s, 1H), 4.15 (t, *J* = 6.0 Hz, 1H), 3.96 (s, 2H), 3.50 (d, *J* = 6.1 Hz, 2H). HRMS (ESI) calcd for $C_{24}H_{19}NOS$ $[M+H]^+$: 370.1260, found: 370.1256

S-((9H-fluoren-9-yl)methyl)2-(1,3-dimethyl-2,6-dioxo-1,2,3,6-tetrahydro-7H-purin-7 yl)ethanethioate (TE13)

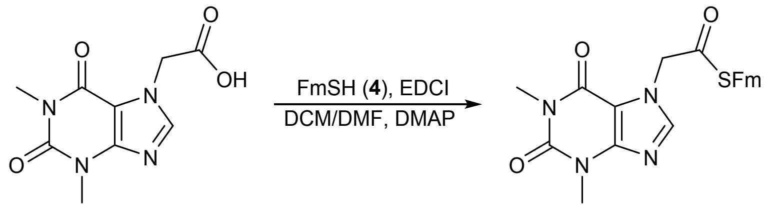

CA13 **TE13**

TE13 was synthesized from **CA13** (commercially available) and thiol **4** as previously described.[11]

S-((9H-fluoren-9-yl)methyl) 4'-fluoro-[1,1'-biphenyl]-4-carbothioate (TE14)

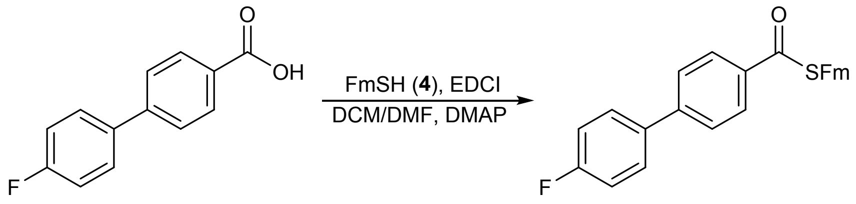

CA14 **TE14**

TE14 was synthesized from **CA14** (commercially available) and thiol **4** using the literature procedure.[10] ^{1}H NMR (400 MHz, $CDCl_3$) δ 8.01 (d, *J* = 8.1 Hz, 2H), 7.77 (t, *J* = 6.8 Hz, 4H), 7.60 (d, *J* = 7.9 Hz, 2H), 7.58 – 7.55 (m, 2H), 7.45 – 7.39 (m, 2H), 7.38 – 7.33 (m, 2H), 7.16 (t, *J* = 8.4 Hz, 2H), 4.30 (t, *J* = 6.0 Hz, 1H), 3.72 (d, *J* = 6.0 Hz, 2H). ^{13}C NMR (101 MHz, $CDCl_3$) δ 191.1, 163.0 (d, *J* = 249 Hz), 145.6, 145.1, 141.0, 135.9, 135.7, 128.9, 128.8, 127.8, 127.7, 127.1, 127.0, 124.7, 119.9, 115.9, 46.8, 32.6. HRMS (ESI) calcd for $C_{27}H_{19}FOS$ $[M+H]^+$: 411.1214, found: 411.1210

S-((9H-fluoren-9-yl)methyl) 2-(9H-carbazol-9-yl)ethanethioate (TE15)

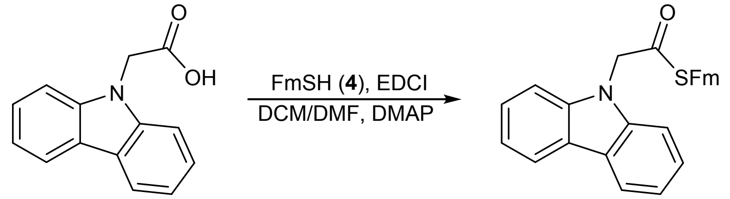

CA15 **TE15**

TE15 was synthesized from **CA15** (commercially available) and thiol **4** using the literature procedure.[10] ^{1}H NMR (500 MHz, $CDCl_3$) δ 8.12 (d, *J* = 7.3 Hz, 2H), 7.71 (d, *J* = 7.3 Hz, 2H), 7.49 – 7.45 (m, 4H), 7.39 – 7.35 (m, 2H), 7.32 – 7.28 (m, 2H), 7.26 – 7.20 (m, 4H), 5.03 (s, 2H), 4.09 (t, *J* = 6.1 Hz, 1H), 3.46 (d, *J* = 6.4 Hz, 2H). ^{13}C NMR (101 MHz, $CDCl_3$) δ 145.0, 141.0, 140.6, 127.7, 127.0, 126.1, 124.7, 123.4, 120.5, 120.0, 119.8, 110.0, 108.5, 52.6, 46.5, 32.0. HRMS (ESI) calcd for $C_{28}H_{21}NOS$ $[M+H]^+$: 420.1417, found: 420.1410

S-((9H-fluoren-9-yl)methyl) 2,2-di(1H-indol-3-yl)ethanethioate (TE16)

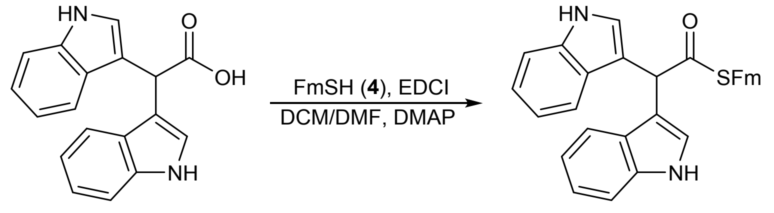

CA16 **TE16**

TE16 was synthesized from **CA16**[13] and thiol **4** using the literature procedure.[10] ^{1}H NMR (400 MHz, $CDCl_3$) δ 7.89 (br. s., 2H), 7.75 (d, *J* = 7.4 Hz, 2H), 7.6 (d, *J* = 7.4 Hz, 2H), 7.52 (d, *J* = 7.8 Hz, 2H), 7.38 (t, *J* = 7.4 Hz, 2H), 7.33 – 7.29 (m, 2H), 7.26 – 7.17 (m, 4H), 7.12 – 7.06 (m, 2H), 6.73 (br. s., 2H), 5.60 (s, 1H), 4.19 (t, *J* = 5.5 Hz, 1H), 3.59 (d, *J* = 5.5 Hz, 2H). ^{13}C NMR (101 MHz, $CDCl_3$) δ 145.4, 141.1, 136.2, 127.5, 127.1, 126.6, 124.8, 123.7, 122.2, 119.8, 119.7, 119.2, 113.1, 111.2, 77.2, 49.0, 46.8, 32.3. HRMS (ESI) calcd for $C_{32}H_{24}N_2OS$ $[M+H]^+$: 485.1682, found: 485.1657

S-((9H-fluoren-9-yl)methyl) 4-(2-(pyridin-2-yl)ethyl)benzothioate (TE17)

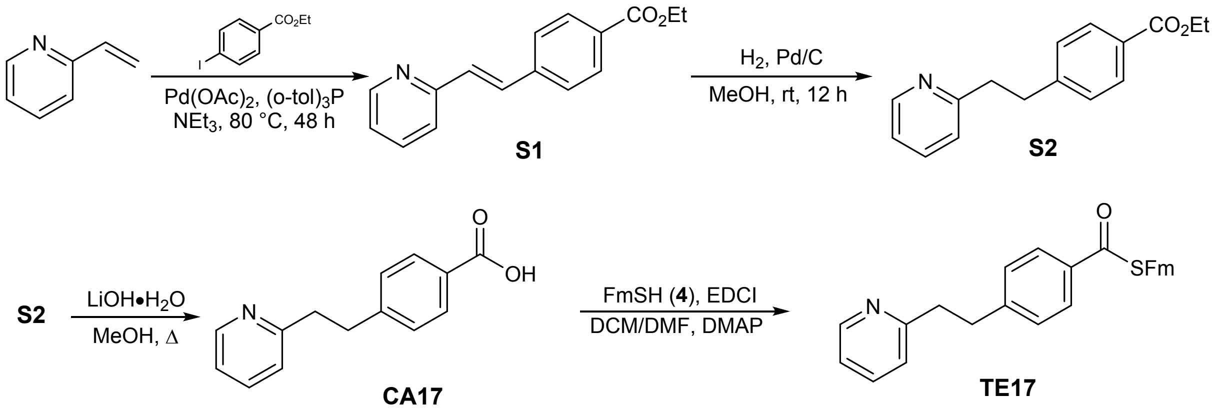

A flame dried sealed tube was charged with 2-vinylpyridine (1.00 g, 9.51 mmol), ethyl 4-iodobenzoate (2.63 g 9.51 mmol), $Pd(OAc)_2$ (64.0 mg, 3.0 mol%), tri-o-tolylphosphine (145 mg, 5.0 mol%), triethylamine (12 mL), a magnetic stir bar and purged with argon. The reaction was heated at 100 °C for 48 h. The reaction mixture was cooled, diluted with CH_2Cl_2 and filtered through Celite. The solution was dried over Na_2SO_4 and the solvent evaporated under reduced pressure. The product (**S1**) was purified using flash column chromatography resulting in a pale yellow oil. **S1** was then subjected to catalytic hydrogenation using an H_2 balloon, Pd/C and methanol. The reaction was allowed to stir for 12 h at rt. The reaction mixture was filtered through Celite and solvent evaporated under reduced pressure. **S2** was used without further purification. Hydrolysis of the ethyl ester was carried out using LiOH monohydrate in a refluxing ethanol/water (9:1) mixture. After 3 h the reaction was judged complete via TLC analysis and the reaction mixture was acidified using 6 M HCl. The resulting acid **CA17** precipitated upon cooling and was recovered by vacuum filtration and used without further purification. Carboxylic acid **CA17** was transformed into **TE17** using the literature procedure.[10] The four synthetic steps gave **TE17** with a 41% overall yield.

4-(2-(pyridin-2-yl)ethyl)benzoic acid (CA17) ^{1}H NMR (400 MHz, $CDCl_3$) δ 8.72 – 8.68 (m, 1H), 8.05 (d, *J* = 7.8 Hz, 2H), 7.66 (dt, *J* = 1.6, 7.6 Hz, 1H), 7.32 (d, *J* = 7.8 Hz, 2H), 7.25 – 7.20 (m,

1H), 7.16 (dd, J = 0.8, 7.8 Hz, 1H), 3.23 – 3.11 (m, 4H). ^{13}C NMR (126 MHz, $CDCl_3$) δ 170.3, 160.2, 148.6, 147.0, 137.3, 130.3, 128.6, 128.3, 123.4, 121.7, 39.3, 36.3.

S-((9H-fluoren-9-yl)methyl) 4-(2-(pyridin-2-yl)ethyl)benzothioate (TE17) ^{1}H NMR (500 MHz, $CDCl_3$) δ 8.59 (d, J = 4.4 Hz, 1H), 7.88 (d, J = 7.8 Hz, 2H), 7.78 (t, J = 6.9 Hz, 4H), 7.60 – 7.55 (m, 1H), 7.45 – 7.40 (m, 2H), 7.38 – 7.34 (m, 2H), 7.26 (d, J = 7.8 Hz, 2H), 7.14 (dd, J = 4.9, 7.3 Hz, 1H), 7.06 (d, J = 7.8 Hz, 1H), 4.28 (t, J = 6.1 Hz, 1H), 3.69 (d, J = 5.9 Hz, 2H), 3.17 – 3.08 (m, 4H). ^{13}C NMR (126 MHz, $CDCl_3$) δ 191.1, 160.3, 149.2, 147.6, 145.5, 140.9, 136.3, 134.8, 128.6, 127.6, 127.3, 127.0, 124.7, 122.9, 121.3, 119.8, 46.7, 39.4, 35.7, 32.5. HRMS (ESI) calcd for $C_{28}H_{23}NOS$ $[M+H]^+$: 422.1573, found: 422.1559

(Z)-S-((9H-fluoren-9-yl)methyl)4-(4-oxo-2-thioxo-5-(2,3,4-trimethoxybenzylidene)thiazolidin-3-yl)benzothioate (TE18)

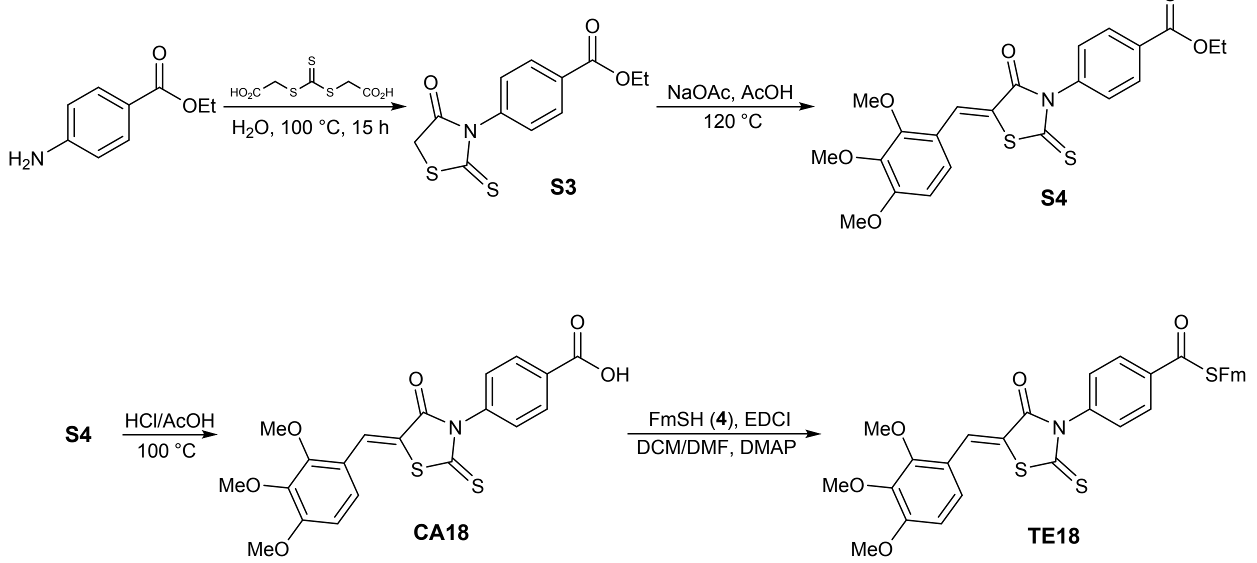

Ethyl 4-aminobenzoate (459 mg, 2.78 mmol), 2,2'-(thiocarbonylbis(sulfanediyl))diacetic acid (629 mg, 2.78 mmol) and 10 mL of water were stirred and heated to reflux for 15 h. The reaction mass was filtered through Celite, extracted using EtOAc and purified using flash column chromatography. **S3** (0.89 mmol) was then combined with 2,3,4-trimethoxybenzaldehyde (175 mg, 0.89 mmol) and a mixture of NaOAc/AcOH (3.5 mL, 40 mg/mL) and heated to 120°C. The resulting precipitate was filtered and recrystallized from AcOH providing pure **S4**. The ester function of **S4** (0.55 mmol) was hydrolyzed using HCl/AcOH (1:1, 4 mL) under reflux conditions. The resulting acid **CA18** was filtered and washed with AcOH and used without further purification. Carboxylic acid **CA18** was transformed into **TE18** using the literature procedure. The four synthetic steps gave **TE18** with a 9.4% overall yield.

(Z)-4-(4-oxo-2-thioxo-5-(2,3,4-trimethoxybenzylidene)thiazolidin-3-yl)benzoic acid (CA18) ^{1}H NMR (400 MHz, DMSO-d_6) δ 11.96 (br. s., 1H), 8.12 (d, J = 8.6 Hz, 2H), 7.85 (s, 1H), 7.6 (d, J = 8.2 Hz, 2H), 7.28 (d, J = 9.0 Hz, 1H), 7.05 (d, J = 9.0 Hz, 1H), 3.90 (s, 6H), 3.80 (s, 3H). ^{13}C NMR (101 MHz, DMSO-d_6) δ 194.0, 166.8, 165.0, 156.6, 153.1, 141.8, 139.3, 130.8, 130.0, 129.3, 128.0, 125.5, 121.1, 119.3, 108.9, 61.8, 60.5, 56.2.

(Z)-S-((9H-fluoren-9-yl)methyl)4-(4-oxo-2-thioxo-5-(2,3,4-trimethoxybenzylidene)thiazolidin-3-yl)benzothioate (TE18) ^{1}H NMR (400 MHz, $CDCl_3$) δ 8.15 – 8.08 (m, 2H), 8.06 (s, 1H), 7.78 (d,

J = 7.4 Hz, 2H), 7.74 (d, J = 7.8 Hz, 2H), 7.45 – 7.38 (m, 4H), 7.38 – 7.33 (m, 2H), 7.21 (d, J = 9.0 Hz, 1H), 6.80 (d, J = 9.0 Hz, 1H), 4.29 (t, J = 5.9 Hz, 1H), 3.99 (s, 3H), 3.95 (s, 3H), 3.91 (s, 3H), 3.74 (d, J = 5.9 Hz, 2H). ^{13}C NMR (101 MHz, $CDCl_3$) δ 193.6, 190.5, 167.3, 156.8, 154.0, 145.3, 142.3, 141.0, 139.2, 137.5, 129.9, 128.8, 128.3, 127.7, 127.1, 125.7, 124.7, 120.9, 120.5, 120.3, 119.9, 107.8, 61.9, 60.9, 56.2, 46.6, 32.7. HRMS (ESI) calcd for $C_{34}H_{27}NO_5S_3$ $[M+H]^+$: 626.1124, found: 626.1105

S-((9H-fluoren-9-yl)methyl) 1-methyl-5-(pyrimidin-5-yl)-1H-indole-3-carbothioate (TE19)

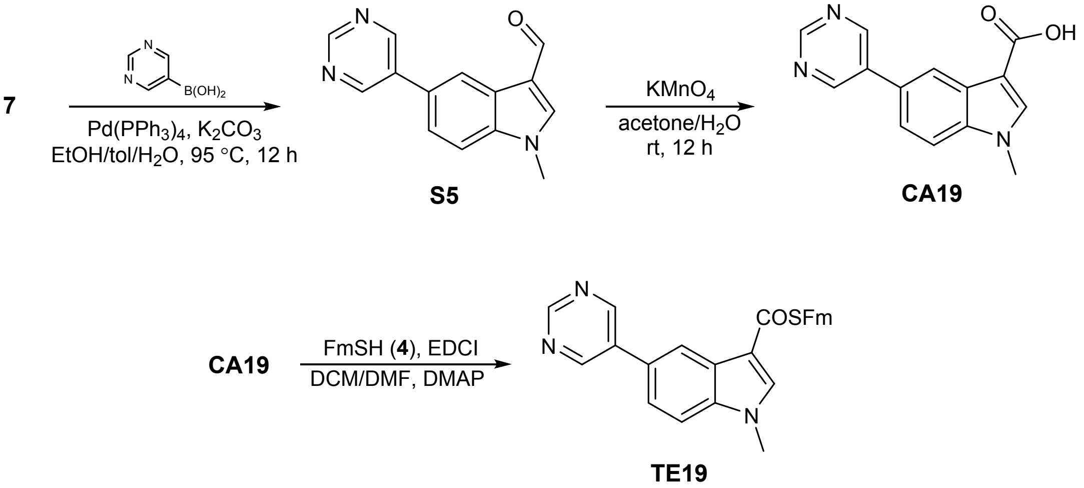

A flame dried sealed tube was charged with **7** (383 mg, 1.61 mmol), pyrimidin-5-ylboronic acid (350 mg, 2.82 mmol), $Pd(PPh_3)_4$ (93.0 mg, 5.0 mol%), K_2CO_3 (890 mg, 6.44 mmol), EtOH, H_2O, toluene solvent mixture (3:3:1, 35.0 mL), a magnetic stir bar and purged with argon. The reaction was heated at 95 °C for 12 h. The reaction mixture was cooled, diluted with CH_2Cl_2 and filtered through Celite. The solution was dried over Na_2SO_4 and the solvent evaporated under reduced pressure. The product **S5** was purified using flash column chromatography resulting in a yellow solid. Aldehyde **S5** was then dissolved in acetone (30 mL), aqueous $KMnO_4$ (481 mg, 0.32 mM) was added dropwise and stirred at rt for three hours. The reaction was judged complete via TLC analysis, quenched with 15% H_2O_2, filtered through Celite, solvent removed under reduced pressure and acidified with 3 M HCl. Upon standing at 0°C, the product **CA19** crystallized and was recovered using vacuum filtration and used without further purification. Carboxylic acid **CA19** was transformed into **TE19** using the literature procedure. The three synthetic steps gave **TE19** with a 11% overall yield.

1-methyl-5-(pyrimidin-5-yl)-1H-indole-3-carboxylic acid (CA19) ^{1}H NMR (500 MHz, DMSO-d_6) δ 9.17 (s, 1H), 9.12 (s, 2H), 8.31 (d, J = 1.0 Hz, 1H), 8.13 (s, 1H), 7.74 – 7.70 (m, 1H), 7.69 – 7.64 (m, 1H), 3.90 (s, 3H). ^{13}C NMR (101 MHz, DMSO-d_6) δ 165.4, 156.6, 154.7, 137.3, 137.2, 134.5, 127.2, 127.0, 121.4, 119.2, 111.7, 106.7, 33.2.

S-((9H-fluoren-9-yl)methyl) 1-methyl-5-(pyrimidin-5-yl)-1H-indole-3-carbothioate (TE19) ^{1}H NMR (400 MHz, $CDCl_3$) δ 9.21 (s, 1H), 9.02 (s, 2H), 8.50 (d, J = 1.2 Hz, 1H), 7.82 – 7.74 (m, 5H), 7.50 – 7.45 (m, 1H), 7.43 – 7.37 (m, 3H), 7.36 – 7.31 (m, 2H), 4.29 (t, J = 5.9 Hz, 1H), 3.80 (s, 3H), 3.72 (d, J = 6.3 Hz, 2H). ^{13}C NMR (101 MHz, $CDCl_3$) δ 183.6, 157.0, 155.0, 145.7, 141.3, 137.2, 135.2, 134.8, 128.7, 127.6, 127.0, 126.2, 124.8, 122.3, 120.6, 119.8, 115.8, 110.8, 47.1, 33.6, 31.6. HRMS (ESI) calcd for $C_{28}H_{21}N_3OS$ $[M+H]^+$: 448.1478, found: 448.1474

S-((9H-fluoren-9-yl)methyl) 4-(3,4-bis(2,4-difluorophenyl)-2,5-dioxo-2,5-dihydro-1H-pyrrol-1-yl)butanethioate (TE20)

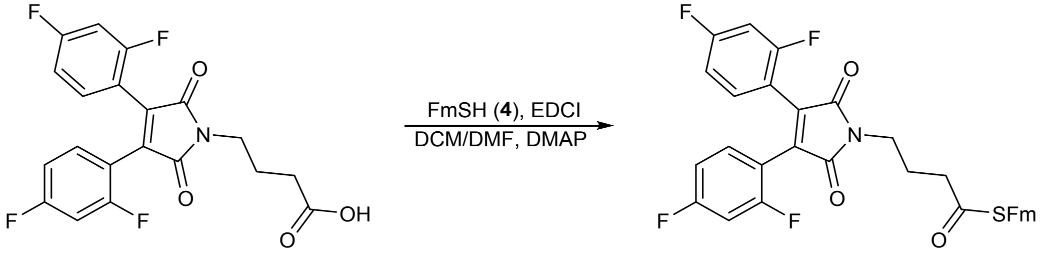

CA20 **TE20**

TE20 was synthesized from **CA20**[6] and thiol **4** using the literature procedure.[10] Yield = 62%. Rf = 0.48 in hexanes : EtOAc = 85 : 25. ^{1}H NMR (250 MHz, $CDCl_3$) δ 7.63 – 7.44 (m, 4H), 7.41 – 7.30 (m, 2H), 7.26 – 7.11 (m, 4H), 6.80 (ddd, J = 8.0, 2.5, 1.3 Hz, 2H), 6.63 (ddd, J = 11.1, 7.2, 2.5 Hz, 2H), 4.05 – 3.92 (m, 1H), 3.51 (dd, J = 8.4, 5.1 Hz, 2H), 3.41 – 3.29 (m, 2H), 2.44 (t, J = 7.3 Hz, 2H), 1.92 – 1.79 (m, 2H). ^{13}C NMR (63 MHz, $CDCl_3$) δ 198.02, 169.46, 164.24 (dd, J = 254.3, 14.3 Hz), 160.66 (dd, J = 254.31, 12.4 Hz), 145.42, 141.15, 133.21, 132.46 (dd, J = 10.2, 4.8 Hz), 127.79, 127.19, 124.75, 119.96, 113.54 (dd, J = 12.7, 3.0 Hz), 111.95 (dd, J = 21.3, 3.4 Hz), 104.66 (t, J = 25.8 Hz), 46.74, 41.40, 37.97, 32.30, 24.28. HRMS (ESI) calcd for $C_{34}H_{23}F_4NO_3S$ $[M+H]^+$: 602.1408, found: 602.1365

S-((9H-fluoren-9-yl)methyl) 2'-methoxy-[1,1'-biphenyl]-4-carbothioate (TE21)

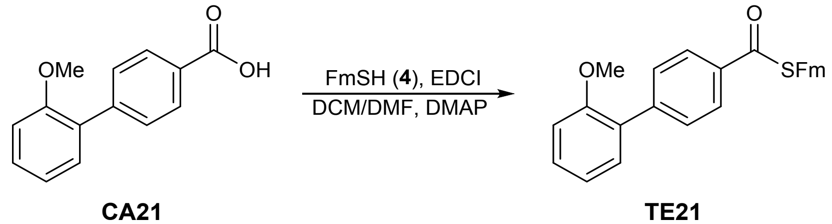

CA21 **TE21**

TE21 was synthesized from **CA21** (commercially available) and thiol **4** using the literature procedure.[10] ^{1}H NMR (400 MHz, $CDCl_3$) δ 8.02 (d, J = 8.2 Hz, 2H), 7.80 (d, J = 8.2 Hz, 4H), 7.63 (d, J = 8.2 Hz, 2H), 7.47 – 7.41 (m, 2H), 7.41 (br. s., 4H), 7.07 (t, J = 7.4 Hz, 1H), 7.02 (d, J = 8.2 Hz, 1H), 4.31 (t, J = 6.1 Hz, 1H), 3.83 (s, 3H), 3.73 (d, J = 5.9 Hz, 2H). ^{13}C NMR (101 MHz, $CDCl_3$) δ 191.2, 156.4, 145.6, 143.9, 141.0, 135.3, 130.6, 129.7, 129.5, 129.3, 127.6, 127.1, 126.9, 124.7, 120.9, 119.9, 111.3, 55.5, 46.8, 32.5. HRMS (ESI) calcd for $C_{28}H_{22}O_2S$ $[M+Na]^+$: 445.1233, found: 445.1216

S-((9H-fluoren-9-yl)methyl) 4-(1-ethylpiperidin-4-yl)benzothioate (TE22)

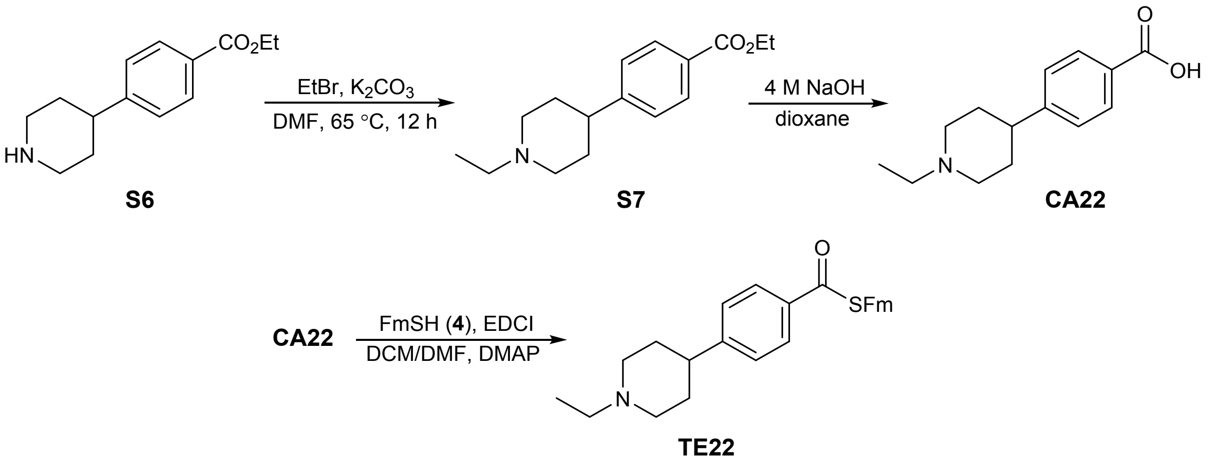

Previously reported **S6**[14] (300 mg, 1.29 mmol) was combined with bromoethane (140 mg, 1.29 mmol), K_2CO_3 (343 mg, 2.57 mmol) in DMF (5 mL) and stirred at 65 °C for 12 h. The reaction was judged complete via TLC analysis. The reaction mixture was diluted with EtOAc, filtered through Celite, washed successively with water and brine, and the solvent was removed under reduced pressure. The product **S7** was purified using flash column chromatography. **S7** was hydrolyzed using 4 M NaOH in dioxane. The reaction mixture was acidified with 3 M HCl. The resulting precipitate containing **CA22** was collected by vacuum filtration and used without further purification. Carboxylic acid **CA22** was transformed into **TE22** using the literature procedure. The three synthetic steps gave **TE22** with a 9.5% overall yield.

4-(1-ethylpiperidin-4-yl)benzoic acid (CA22)[15]

S-((9H-fluoren-9-yl)methyl) 4-(1-ethylpiperidin-4-yl)benzothioate (TE22) ^{1}H NMR (400 MHz, $CDCl_3$) δ 7.87 (d, J = 8.2 Hz, 2H), 7.73 (d, J = 7.4 Hz, 2H), 7.7 (d, J = 7.8 Hz, 2H), 7.40 – 7.34 (m, 2H), 7.33 – 7.28 (m, 2H), 7.22 (m, 2H), 4.23 (s, 1H), 3.75 (d, J = 11.7 Hz, 2H), 3.65 (d, J = 6.3 Hz, 2H), 3.12 (dd, J = 3.9, 7.0 Hz, 2H), 2.75 (d, J = 9.4 Hz, 3H), 2.21 (d, J = 12.1 Hz, 2H), 2.02 (d, J = 14.1 Hz, 2H), 1.37 (t, J = 7.2 Hz, 3H). ^{13}C NMR (101 MHz, $CDCl_3$) δ 191.0, 148.3, 145.5, 141.0, 136.0, 127.8, 127.7, 127.1, 126.9, 124.7, 119.9, 77.2, 52.6, 52.5, 46.7, 40.3, 32.6, 29.9. HRMS (ESI) calcd for $C_{28}H_{29}NOS$ $[M+H]^+$: 428.2043, found: 428.2022

S-((9H-fluoren-9-yl)methyl) 5-(4-ethoxy-3-fluorophenyl)-1-methyl-1H-indole-3-carbothioate (TE23)

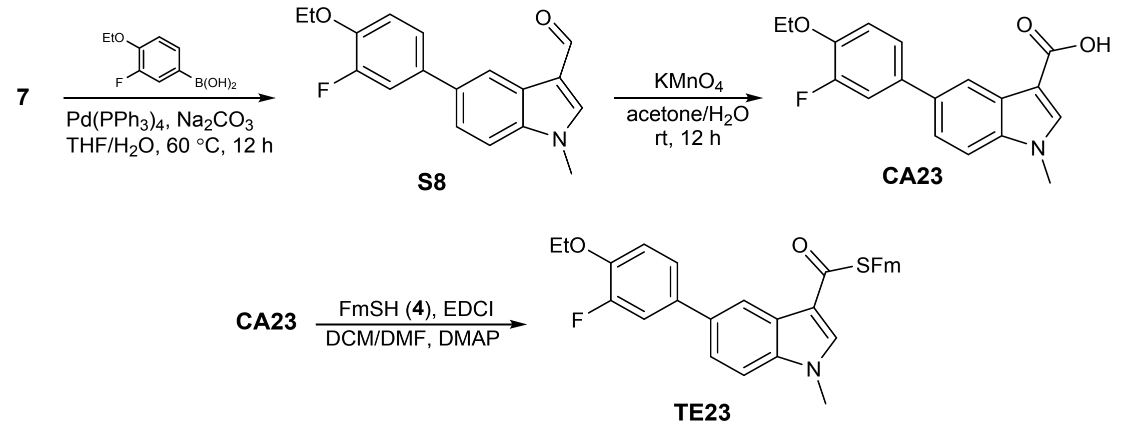

A flame dried sealed tube was charged with **7** (860 mg, 3.61 mmol), (4-ethoxy-3-fluorophenyl)boronic acid (997 mg, 5.42 mmol), $Pd(PPh_3)_4$ (208 mg, 5.0 mol%), Na_2CO_3 (1.92 g, 18.5 mmol), THF, H_2O solvent mixture (3:1, 18.0 mL), a magnetic stir bar and purged with argon. The reaction was heated at 60 °C for 12 h. The reaction mixture was cooled, diluted with EtOAc and filtered through Celite. The solution was dried over Na_2SO_4 and the solvent evaporated under reduced pressure. The product **S8** was purified using flash column chromatography resulting in a yellow solid. Aldehyde **S8** was then dissolved in acetone (35 mL), aqueous $KMnO_4$ (561 mg, 0.32 mM) was added dropwise and stirred at rt for three hours. The reaction was judged complete via TLC analysis, quenched with 15% H_2O_2, filtered through Celite, solvent removed under reduced pressure and acidified with 3 M HCl. Upon standing at 0 °C the product **CA23** crystallized, was recovered using vacuum filtration, and recrystallized from MeOH. Carboxylic acid **CA23** was transformed into **TE23** using the literature procedure. The three synthetic steps gave **TE23** with a 13% overall yield.

5-(4-ethoxy-3-fluorophenyl)-1-methyl-1H-indole-3-carboxylic acid (CA23) ^{1}H NMR (400 MHz, DMSO-d$_6$) δ 12.03 (br. s., 1H), 8.19 (br. s., 1H), 8.06 (br. s., 1H), 7.62 – 7.34 (m, 4H), 7.23 (t, J = 8.2 Hz, 1H), 4.14 (d, J = 6.6 Hz, 2H), 3.87 (br. s., 3H), 1.37 (t, J = 6.4 Hz, 3H). ^{13}C NMR (101 MHz, DMSO-d$_6$) δ 165.6, 152.0 (d, J = 244 Hz), 145.3 (d, J = 10 Hz), 136.8, 136.5, 134.5 (d, J = 6 Hz), 132.5, 127.0, 122.8 (d, J = 3 Hz), 121.3, 118.3, 115.3, 114.2 (d, J = 18 Hz), 111.2, 106.5, 64.4, 33.1, 14.7.

S-((9H-fluoren-9-yl)methyl) 5-(4-ethoxy-3-fluorophenyl)-1-methyl-1H-indole-3-carbothioate (TE23) ^{1}H NMR (400 MHz, $CDCl_3$) δ 8.46 (d, J = 1.2 Hz, 1H), 7.83 (s, 1H), 7.80 (d, J = 7.9 Hz, 2H), 7.77 (d, J = 7.4 Hz, 2H), 7.50 (dd, J = 1.8, 8.4 Hz, 1H), 7.43 (dd, J = 2.2, 12.7 Hz, 1H), 7.39 (d, J = 7.4 Hz, 4H), 7.36 – 7.31 (m, J = 1.2, 7.4 Hz, 2H), 7.04 (t, J = 8.4 Hz, 1H), 4.31 (t, J = 6.1 Hz, 1H), 4.18 (q, J = 7.0 Hz, 2H), 3.85 (s, 3H), 3.71 (d, J = 6.3 Hz, 2H), 1.50 (t, J = 7.0 Hz, 3H). HRMS (ESI) calcd for $C_{32}H_{26}FNO_2S$ $[M+H]^+$: 508.1741, found: 508.1719

S-((9H-fluoren-9-yl)methyl) 4-(2,3,4-trimethoxybenzyl)benzothioate (TE24)

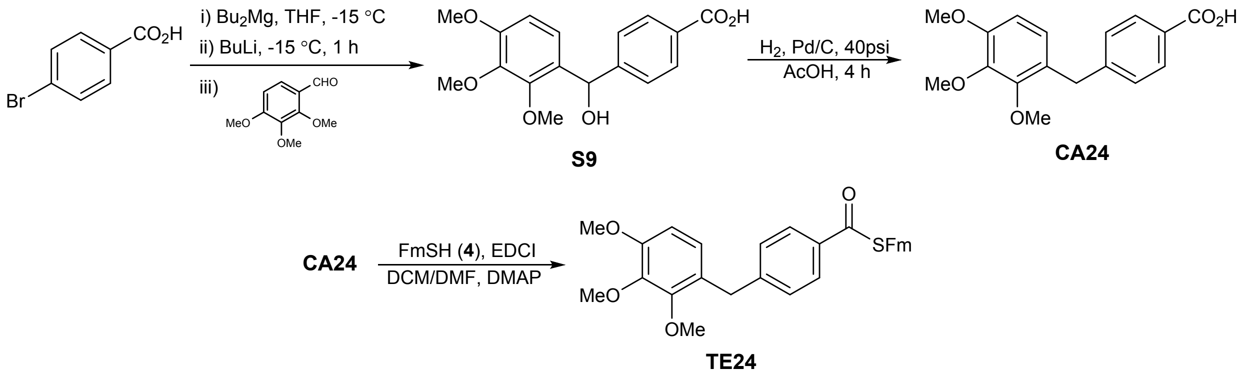

A flame dried flask was charged with 4-bromobenzoic acid (1.00 g, 4.97 mmol), THF (5 mL), a magnetic stir bar, purged with argon and cooled to -15 °C. A 1.0 M solution of dibutylmagnesium in heptane (2.58 mL, 2.59 mmol) was added dropwise followed by the addition of a 2.5 M solution of *n*-butyllithium (2.20 mL, 5.32 mmol) over the course of 20 min and stirred for 1 h. A solution of 2,3,4-trimethoxybenzaldehyde (1.95 g, 4.97 mmol) in THF (2 mL) was added and stirred for 1 h. The resulting reaction mixture was quenched with 1 M HCl, allowed to come to rt and stirred overnight. The resulting solution containing **S9** was extracted with EtOAc, dried with Na_2SO_4, concentrated under reduced pressure and purified using flash column chromatography. Catalytic hydrogenation was performed on **S9** by dissolving the residue in AcOH, adding the solution to a Parr hydrogenator pressure bottle along with Pd/C. The pressure bottle was purged with hydrogen gas three times, charged with a hydrogen atmosphere to 40 psi and shaken for 4 h. The reaction was judged to be complete via TLC analysis. The reaction mass was then filtered through Celite, washed with MeOH, concentrated under reduced pressure and crude **CA24** purified using flash column chromatography. Carboxylic acid **CA24** was transformed into **TE24** using the literature procedure. The three synthetic steps gave **TE24** with a 25% overall yield.

4-(2,3,4-trimethoxybenzyl)benzoic acid (CA24) ^{1}H NMR (400 MHz, $CDCl_3$) δ 8.02 (d, *J* = 6.3 Hz, 2H), 7.30 (d, *J* = 6.6 Hz, 2H), 6.81 (d, *J* = 7.4 Hz, 1H), 6.62 (d, *J* = 7.4 Hz, 1H), 3.99 (br. s., 2H), 3.89 – 3.80 (m, 6H), 3.73 (br. s., 3H).

S-((9H-fluoren-9-yl)methyl) 4-(2,3,4-trimethoxybenzyl)benzothioate (TE24) ^{1}H NMR (400 MHz, $CDCl_3$) δ 7.90 – 7.86 (m, 2H), 7.76 (t, *J* = 6.4 Hz, 4H), 7.44 – 7.38 (m, 2H), 7.37 – 7.31 (m, 2H), 7.29 – 7.25 (m, 2H), 6.80 (d, *J* = 8.6 Hz, 1H), 6.62 (d, *J* = 8.6 Hz, 1H), 4.26 (t, *J* = 6.1 Hz, 1H), 3.96 (s, 2H), 3.89 (s, 3H), 3.86 (s, 3H), 3.75 (s, 3H), 3.67 (d, *J* = 6.3 Hz, 2H). ^{13}C NMR (101 MHz, $CDCl_3$) δ 191.1, 152.6, 151.8, 147.8, 145.6, 142.4, 141.0, 134.8, 128.8, 127.6, 127.3, 127.0, 126.0, 124.7, 124.4, 119.8, 107.1, 60.7, 60.6, 55.9, 46.8, 35.9, 32.5. HRMS (ESI) calcd for $C_{31}H_{28}O_4S$ $[M+H]^+$: 497.1781, found: 497.1732

S-((9H-fluoren-9-yl)methyl) 4-(pyridin-2-yl)benzothioate (TE25)

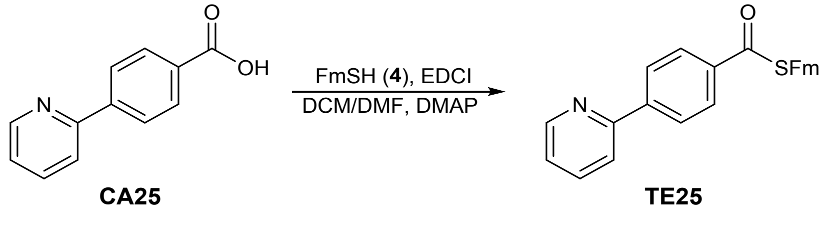

TE25 was synthesized from **CA25** (commercially available) and thiol **4** using the literature procedure.[10] ^{1}H NMR (400 MHz, $CDCl_3$) δ 8.76 – 8.70 (m, 1H), 8.09 – 8.03 (m, 4H), 7.80 – 7.75 (m, 6H), 7.45 – 7.39 (m, 2H), 7.38 – 7.33 (m, 2H), 7.30 – 7.28 (m, 1H), 4.30 (t, *J* = 6.1 Hz, 1H), 3.72 (d, *J* = 6.3 Hz, 2H). ^{13}C NMR (101 MHz, $CDCl_3$) δ 191.2, 155.8, 149.9, 145.5, 143.9, 141.0, 137.0, 136.7, 127.68, 127.66, 127.1, 126.9, 124.7, 122.9, 120.9, 119.9, 46.8, 32.7. HRMS (ESI) calcd for $C_{26}H_{19}NOS$ $[M+H]^+$: 394.1260, found: 394.1256

S-((9H-fluoren-9-yl)methyl) 1-methyl-5-(4-(methylsulfonyl)phenyl)-1H-indole-3-carbothioate (TE26)

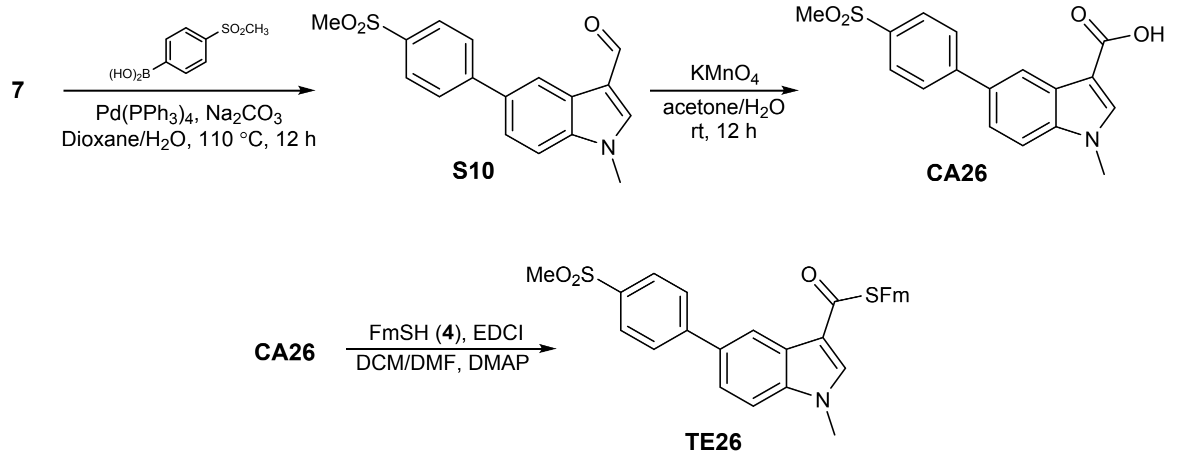

A flame dried sealed tube was charged with **7** (500 mg, 2.10 mmol), (4-(methylsulfonyl)phenyl)boronic acid (630 mg, 2.65 mmol), $Pd(PPh_3)_4$ (146 mg, 6.0 mol%), Na_2CO_3 (1.11 g, 10.5 mmol), dioxane/H_2O solvent mixture (4:1, 30.0 mL), a magnetic stir bar and purged with argon. The reaction was heated at 110 °C for 12 h. The reaction mixture was cooled, diluted with CH_2Cl_2 and filtered through Celite. The solution was dried over Na_2SO_4 and the solvent evaporated under reduced pressure. The product **S10** was purified using flash column chromatography resulting in a yellow solid. Aldehyde **S10** was then dissolved in acetone (21 mL), aqueous $KMnO_4$ (524 mg, 0.30 mM) was added dropwise, and stirred at rt for three hours. The reaction was judged complete via TLC analysis, quenched with 15% H_2O_2, filtered through Celite, solvent removed under reduced pressure and acidified with 3 M HCl. Upon standing at 0 °C the product **CA26** crystallized, was recovered using vacuum filtration and used without further purification. Carboxylic acid **CA26** was transformed into **TE26** using the literature procedure. The three synthetic steps gave **TE26** with a 22% overall yield.

1-methyl-5-(4-(methylsulfonyl)phenyl)-1H-indole-3-carboxylic acid (CA26) ^{1}H NMR (400 MHz, DMSO-d_6) δ 12.11 (br. s., 1H), 8.35 (s, 1H), 8.12 (s, 1H), 8.03 – 7.99 (m, 2H), 7.97 – 7.92 (m, 2H), 7.70 – 7.62 (m, 2H), 3.89 (s, 3H), 3.25 (s, 3H). ^{13}C NMR (101 MHz, DMSO-d_6) δ 165.5, 146.3, 138.8, 137.21, 137.17, 131.9, 127.7, 127.6, 127.0, 121.7, 119.4, 111.5, 106.7, 43.7, 33.1.

S-((9H-fluoren-9-yl)methyl) 1-methyl-5-(4-(methylsulfonyl)phenyl)-1H-indole-3-carbothioate (TE26) ^{1}H NMR (400 MHz, $CDCl_3$) δ 8.54 (s, 1H), 8.00 (d, *J* = 8.6 Hz, 2H), 7.87 (s, 1H), 7.84 (d, *J* = 6.7 Hz, 2H), 7.78 (t, *J* = 7.6 Hz, 4H), 7.55 (d, *J* = 8.2 Hz, 1H), 7.43 – 7.37 (m, 3H), 7.37 – 7.30 (m, 2H), 4.30 (t, *J* = 6.1 Hz, 1H), 3.83 (s, 3H), 3.72 (d, *J* = 5.9 Hz, 2H), 3.11 (s, 3H). ^{13}C NMR (101 MHz, $CDCl_3$) δ 147.2, 145.8, 141.0, 138.5, 137.3, 135.3, 133.7, 128.2, 127.8, 127.6, 127.1, 126.1,

124.8, 122.9, 121.0, 119.8, 115.9, 110.5, 47.2, 44.7, 33.7, 31.6, 29.8. HRMS (ESI) calcd for $C_{31}H_{25}NO_3S_2$ $[M+H]^+$: 524.1349, found: 524.1331

S-((9H-fluoren-9-yl)methyl) 2,2-bis(1-isopropyl-1H-indol-3-yl)ethanethioate (TE27)

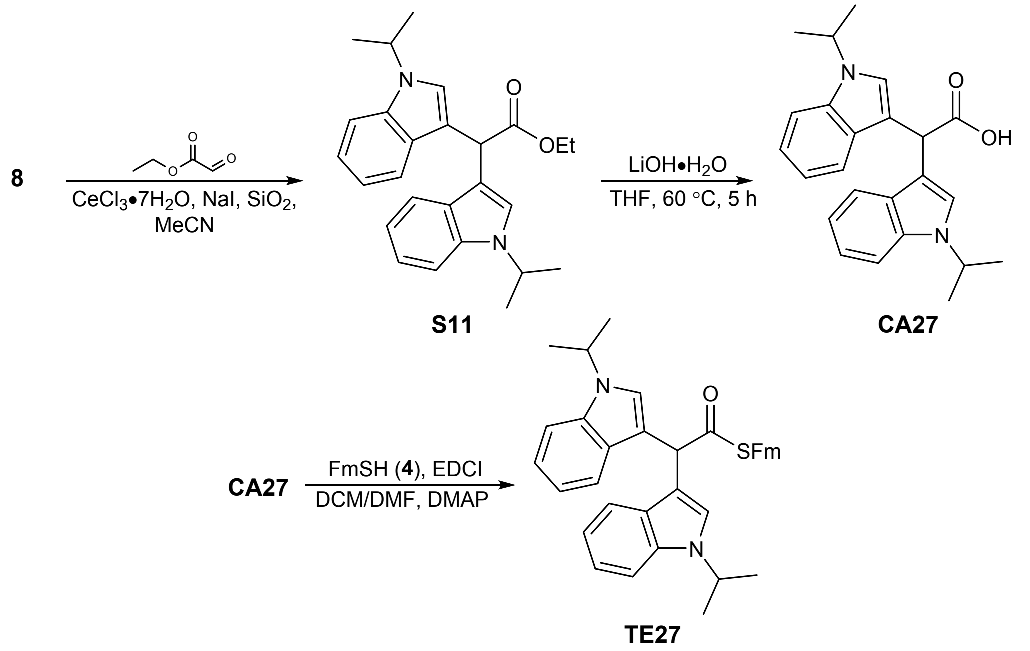

To a round bottom flask was added silica gel (SiO_2, 1.92 g), $CeCl_3$•$7H_2O$ (143 mg, 0.383 mmol), NaI (57.5 mg, 0.383 mmol), MeCN (8.5 mL) and a magnetic stir bar. The mixture was stirred for 12 h and concentrated under reduced pressure resulting in a yellow solid. To the flask was added 1-isopropyl-1*H*-indole (600 mg, 3.77 mmol), ethyl glyoxalate solution in toluene (192 mg, 1.90 mmol) and additional MeCN (5 mL). The reaction was stirred for 4 h, diluted with Et_2O and filtered through Celite. The solvent was removed under reduced pressure and the crude **S11** was purified using flash column chromatography. Ester **S11** underwent hydrolysis using LiOH•H_2O (157 mg, 3.75 mmol) and THF (6 mL) at reflux. The reaction mixture was acidified and the precipitate of **CA27** recovered using vacuum filtration. Carboxylic acid **CA27** was transformed into **TE27** using the literature procedure. The three synthetic steps gave **TE27** with a 13% overall yield.

2,2-bis(1-isopropyl-1H-indol-3-yl)acetic acid (CA27) ^1H NMR (400 MHz, $CDCl_3$) δ 7.54 (d, J = 7.8 Hz, 2H), 7.24 (d, J = 8.2 Hz, 2H), 7.11 (s, 2H), 7.08 (t, J = 7.4 Hz, 2H), 6.99 – 6.92 (m, 2H), 5.43 (s, 1H), 4.56 – 4.44 (m, 2H), 1.35 (dd, J = 7.2, 8.8 Hz, 12H). ^{13}C NMR (101 MHz, $CDCl_3$) δ 179.0, 135.8, 127.2, 123.0, 121.3, 119.5, 119.0, 111.5, 109.5, 47.0, 40.8, 22.64, 22.57.

S-((9H-fluoren-9-yl)methyl) 2,2-bis(1-isopropyl-1H-indol-3-yl)ethanethioate (TE27) ^1H NMR (400 MHz, $CDCl_3$) δ 7.75 (d, J = 7.4 Hz, 2H), 7.61 (t, J = 7.2 Hz, 4H), 7.42 – 7.36 (m, 4H), 7.27 – 7.21 (m, 4H), 7.14 (s, 2H), 7.13 – 7.07 (m, 2H), 5.69 (s, 1H), 4.66 (spt, J = 6.7 Hz, 2H), 4.19 (t, J = 6.1 Hz, 1H), 3.51 (d, J = 6.3 Hz, 2H), 1.51 (t, J = 6.8 Hz, 12H). ^{13}C NMR (101 MHz, $CDCl_3$) δ 199.1, 145.6, 141.0, 136.0, 127.5, 127.3, 127.0, 124.8, 123.3, 121.4, 119.73, 119.65, 119.1, 111.8, 109.6, 49.5, 47.2, 46.7, 32.8, 22.7. HRMS (ESI) calcd for $C_{38}H_{36}N_2OS$ $[M+H]^+$: 569.2621, found: 569.2600

S-((9H-fluoren-9-yl)methyl) 2-(3-oxo-3,4-dihydro-2H-benzo[b][1,4]oxazin-2-yl)ethanethioate (TE28)

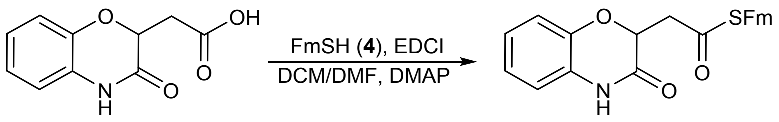

TE28 was synthesized from **CA28** (commercially available) and thiol **4** using the literature procedure.[10] Yield = 64 % yield. Rf = 0.55 in hexanes : EtOAc = 95 : 15. ^{1}H NMR (400 MHz, $CDCl_3$) δ 8.68 (s, 1H), 7.74 (d, *J* = 7.5 Hz, 2H), 7.68 – 7.61 (m, 2H), 7.41 – 7.35 (m, 2H), 7.30 (qd, *J* = 7.4 Hz, 1.2, 2H), 6.96 – 6.85 (m, 3H), 6.80 – 6.74 (m, 1H), 4.99 (dd, *J* = 8.1, 3.8 Hz, 1H), 4.18 (t, *J* = 5.9 Hz, 1H), 3.57 (d, *J* = 6.0 Hz, 2H), 3.24 (dd, *J* = 16.1, 3.8 Hz, 1H), 3.12 (dd, *J* = 16.1, 8.1 Hz, 1H). ^{13}C NMR (101 MHz, $CDCl_3$) δ 194.80, 166.23, 145.27, 142.78, 141.03, 127.73, 127.12, 126.03, 124.68, 124.28, 122.91, 119.87, 117.26, 115.72, 73.37, 46.56, 44.26, 32.52. HRMS (ESI) calcd for $C_{24}H_{19}NO_3S$ $[M+H]^+$: 402.1159, found: 402.1156

S-((9H-fluoren-9-yl)methyl) 1-phenyl-3-(trifluoromethyl)-1H-pyrazole-5-carbothioate (TE29)

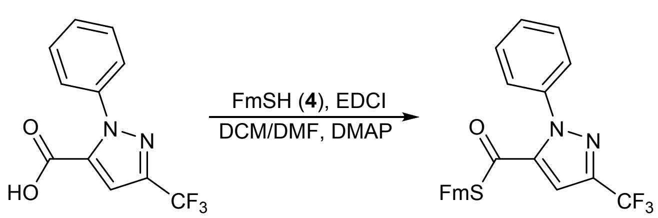

TE29 was synthesized from **CA29** (commercially available) and thiol **4** using the literature procedure.[10] ^{1}H NMR (250 MHz, $CDCl_3$) δ 7.69 (d, *J* = 7.4 Hz, 2H), 7.53 (d, *J* = 7.6 Hz, 2H), 7.44 – 7.33 (m, 4H), 7.29 – 7.17 (m, 5H), 7.05 (s, 1H), 4.17 (t, *J* = 5.5 Hz, 1H), 3.63 (d, *J* = 5.5 Hz, 2H). ^{13}C NMR (101 MHz, $CDCl_3$) δ 192.5, 145.4, 144.7, 129.7, 129.5, 129.0, 128.1, 127.9, 127.2, 125.8, 124.7, 123.8, 120.0, 109.3, 108.2, 46.7, 32.4. HRMS (ESI) calcd $C_{25}H_{17}F_3N_2OS$ $[M+H]^+$: 451.1087, found: 451.1035

S-((9H-fluoren-9-yl)methyl) 1-(2,4-bis(trifluoromethyl)benzyl)-1H-indole-5-carbothioate (TE30)

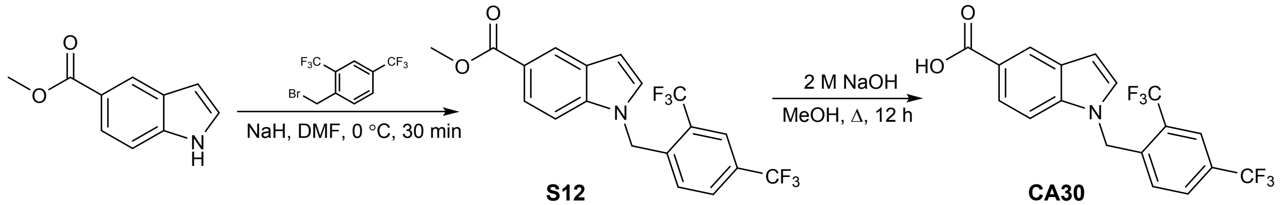

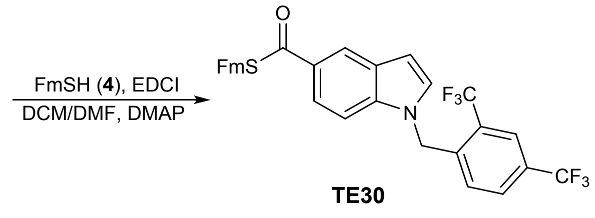

A round bottom flask was charged with methyl 1*H*-indole-5-carboxylate (1.00 g, 5.71 mmol), DMF (10 mL), a magnetic stir bar and cooled to 0 °C. NaH (302 mg, 7.55 mmol, 60% dispersion) was added portion wise and allowed to stir for 30 min. To the reaction was added 1-(bromomethyl)-2,4-bis(trifluoromethyl)benzene (1.75 g, 5.71 mmol) and the reaction was allowed to stir for an additional 30 min. The reaction was quenched with ice, extracted with EtOAc, dried with Na_2SO_4, solvent removed under reduced pressure and purified using flash column chromatography. Ester **S12** underwent hydrolysis using NaOH (150 mg, 3.75 mmol) and MeOH (6 mL) at reflux for 12 h. The reaction mixture was acidified and the precipitate containing **CA30** was recovered using vacuum filtration. Carboxylic acid **CA30** was transformed into **TE30** using the literature procedure. The three synthetic steps gave **TE30** with a 13% overall yield.

1-(2,4-bis(trifluoromethyl)benzyl)-1H-indole-5-carboxylic acid (CA30) ^{1}H NMR (400 MHz, DMSO-d_6) δ 12.55 (br. s., 1H), 8.34 (s, 1H), 8.06 (s, 1H), 7.88 (d, *J* = 8.6 Hz, 1H), 7.75 (dd, *J* = 1.6, 8.6 Hz, 1H), 7.59 (d, *J* = 3.1 Hz, 1H), 7.30 (d, *J* = 8.6 Hz, 1H), 6.76 (d, *J* = 3.1 Hz, 1H), 6.64 (d, *J* = 8.2 Hz, 1H), 5.78 (s, 2H).

S-((9H-fluoren-9-yl)methyl) 1-(2,4-bis(trifluoromethyl)benzyl)-1H-indole-5-carbothioate (TE30) (Rf = 0.55 in hexanes : EtOAc = 9 : 1). ^{1}H NMR (250 MHz, $CDCl_3$) δ 8.28 (d, *J* = 1.3 Hz, 1H), 7.89 (s, 1H), 7.76 – 7.59 (m, 4H), 7.47 (d, *J* = 8.0 Hz, 1H), 7.35 – 6.97 (m, 7H), 6.64 (d, *J* = 3.0 Hz, 1H), 6.49 (d, *J* = 8.1 Hz, 1H), 5.48 (s, 2H), 4.17 (t, *J* = 6.0 Hz, 1H), 3.58 (d, *J* = 6.1 Hz, 2H). ^{13}C NMR (101 MHz, $CDCl_3$) δ 191.3, 145.8, 141.0, 139.9, 138.8, 130.0 (d, *J* = 3 Hz), 129.9, 129.4, 128.5 (d, *J* = 32), 128.3, 127.9, 127.6, 127.1, 126.5,124.8, 123.4 (d, *J* = 273 Hz), 122.5, 122.1, 121.7, 119.9, 109.2, 104.7, 47.0, 46.4 (d, *J* = 4 Hz), 32.6. HRMS (ESI) calcd for $C_{32}H_{21}F_6NOS$ $[M+H]^+$: 582.1321, found: 582.1308

S-((9H-fluoren-9-yl)methyl) 4-(di(1H-indol-3-yl)methyl)benzothioate (TE31)

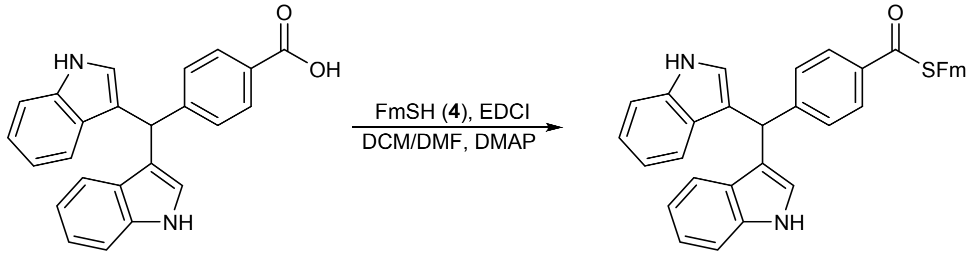

CA31 **TE31**

TE31 was synthesized from **CA31**[8] and thiol **4** using the literature procedure.[10] ^{1}H NMR (400 MHz, $CDCl_3$) δ 7.92 (br. s., 2H), 7.88 (d, *J* = 8.2 Hz, 2H), 7.76 (t, *J* = 7.0 Hz, 4H), 7.45 – 7.30 (m, 10H), 7.20 (t, *J* = 7.8 Hz, 2H), 7.06 – 6.99 (m, 2H), 6.62 (d, *J* = 2.3 Hz, 2H), 5.94 (s, 1H), 4.26 (s, 1H), 3.67 (d, *J* = 5.9 Hz, 2H). ^{13}C NMR (101 MHz, $CDCl_3$) δ 191.4, 150.2, 145.7, 141.0, 136.6, 135.1, 128.9, 127.7, 127.4, 127.1, 126.8, 124.8, 123.6, 122.1, 119.9, 119.7, 119.4, 118.6, 111.1, 46.8, 39.7, 32.6. HRMS (ESI) calcd for $C_{38}H_{28}N_2OS$ $[M+H]^+$: 561.1995, found: 561.1982

S-((9H-fluoren-9-yl)methyl) 3-(6-chloro-3-oxo-2H-benzo[b][1,4]oxazin-4(3H)-yl)propanethioate (TE32)

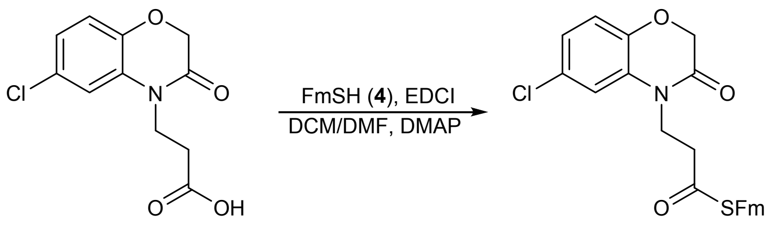

CA32 **TE32**

TE32 was synthesized from **CA32** (commercially available) and thiol **4** using the literature procedure.[10] Yield = 75%. Rf = 0.50 in hexanes : EtOAc = 85 : 15. ^{1}H NMR (250 MHz, $CDCl_3$) δ 7.61 (d, *J* = 7.4 Hz, 2H), 7.48 (d, *J* = 7.3 Hz, 2H), 7.22 (tt, *J* = 11.2, 5.6 Hz, 4H), 6.92 – 6.74 (m, 3H), 4.50 – 4.33 (m, 2H), 4.12 – 3.92 (m, 3H), 3.58 – 3.34 (m, 2H), 2.85 – 2.56 (m, 2H). ^{13}C NMR (101 MHz, $CDCl_3$) δ 196.2, 164.0, 145.1, 143.9, 141.1, 129.5, 128.0, 127.8, 127.1, 124.6, 123.8, 119.9, 118.2, 114.9, 67.4, 46.5, 40.9, 37.6, 32.5. HRMS (ESI) calcd for $C_{25}H_{20}ClNO_3S$ $[M+H]^+$: 450.0925, found: 450.0900.

(E)-S-((9H-fluoren-9-yl)methyl) 3-(benzo[d][1,3]dioxol-5-yl)prop-2-enethioate (TE33)

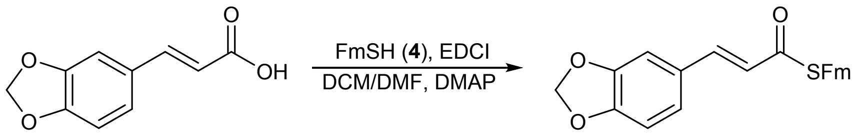

CA33 **TE33**

TE33 was synthesized from **CA33** (commercially available) and thiol **4** using the literature procedure.[10] ^{1}H NMR (500 MHz, $CDCl_3$) δ 7.77 (d, *J* = 7.8 Hz, 2H), 7.72 (d, *J* = 7.8 Hz, 2H), 7.51 (d, *J* = 15.7 Hz, 1H), 7.43 – 7.39 (m, 2H), 7.36 – 7.32 (m, 2H), 7.03 – 7.00 (m, 2H), 6.81 (d, *J* = 8.8 Hz, 1H), 6.51 (d, *J* = 15.7 Hz, 1H), 6.01 (s, 2H), 4.25 (t, *J* = 6.1 Hz, 1H), 3.64 (d, *J* = 5.9 Hz, 2H). ^{13}C NMR (126 MHz, $CDCl_3$) δ 189.2, 149.9, 148.4, 145.5, 141.0, 140.3, 128.4, 127.7, 127.1, 125.1, 124.7, 122.9, 119.9, 108.6, 106.5, 101.6, 46.8, 32.3. HRMS (ESI) calcd for $C_{24}H_{18}O_3S$ $[M+H]^+$: 387.1050, found: 387.1060

S-((9H-fluoren-9-yl)methyl) 2-(3-oxo-3,4-dihydro-2H-benzo[b][1,4]thiazin-2-yl)ethanethioate (TE34)

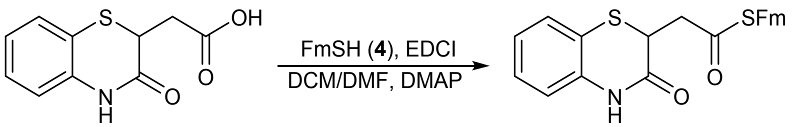

CA34 **TE34**

TE34 was synthesized from **CA34** (commercially available) and thiol **4** using the literature procedure.[10] Yield = 70 % yield. Rf = 0.55 in hexanes : EtOAc = 85 : 15. ^{1}H NMR (400 MHz, $CDCl_3$) δ 8.88 (s, 1H), 7.81 – 7.71 (m, 2H), 7.66 – 7.60 (m, 2H), 7.46 – 7.19 (m, 5H), 7.13 (td, *J* = 7.6, 1.4 Hz, 1H), 7.07 – 6.95 (m, 1H), 6.83 (dt, *J* = 33.9 Hz, 18.9Hz, 1H), 4.27 – 4.14 (m, 1H), 3.99 (dt, *J* = 8.6, 5.4 Hz, 1H), 3.55 (dt, *J* = 8.7, 6.2 Hz, 2H), 3.20 (dt, *J* = 16.0, 5.4 Hz, 1H), 2.82 – 2.68

(m, 1H). ^{13}C NMR (101 MHz, $CDCl_3$) δ 195.1, 166.6, 145.2, 141.1, 135.8, 128.2, 127.8, 127.5, 127.2, 124.7, 124.1, 119.9, 118.8, 117.1, 46.6, 42.7, 38.1, 32.6. HRMS (ESI) calcd for $C_{24}H_{19}NO_2S_2$ $[M+H]^+$: 418.0930, found: 418.0897

S-((9H-fluoren-9-yl)methyl) 3-(5-phenyl-1,3,4-oxadiazol-2-yl)propanethioate (TE35)

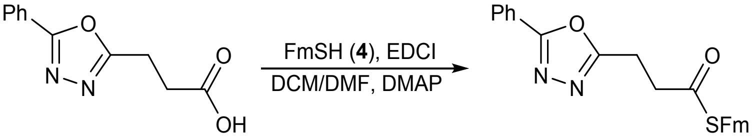

CA35 **TE35**

TE35 was synthesized from **CA35** (commercially available) and thiol **4** using the literature procedure.[10] Yield = 70 % yield. Rf = 0.50 in hexanes : EtOAc = 85 : 15. ^1H NMR (250 MHz, $CDCl_3$) δ 7.85 (dd, J = 7.7, 1.8 Hz, 2H), 7.57 (d, J = 7.1 Hz, 2H), 7.47 (d, J = 7.3 Hz, 2H), 7.36 – 7.10 (m, 7H), 4.09 – 3.96 (m, 1H), 3.50 – 3.34 (m, 2H), 3.08 – 2.98 (m, 2H), 2.96 – 2.85 (m, 2H). ^{13}C NMR (63 MHz, $CDCl_3$) δ 196.4, 165.0, 164.8, 145.1, 141.0, 131.6, 129.0, 127.7, 127.1, 126.7, 124.6, 123.8, 119.9, 46.64, 39.69, 32.35, 21.07. HRMS (ESI) calcd for $C_{25}H_{20}N_2O_2S$ $[M+H]^+$: 413.1318 , found: 413.1313

S-((9H-fluoren-9-yl)methyl) 4-(5,6-dichloro-1H-benzo[d]imidazol-2-yl)benzothioate (TE36)

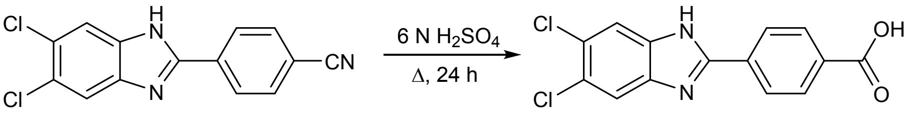

CA36

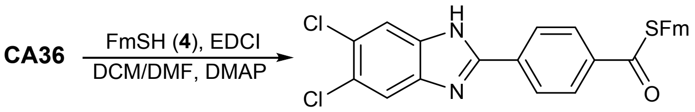

TE36

TE36 was synthesized from **CA36**[16] and thiol **4** using the literature procedure.[10] ^1H NMR (500 MHz, DMSO-d_6) δ 13.4 (s,1H) 8.23 (d, J = 8.3 Hz, 2H), 7.94 (d, J = 8.3 Hz, 2H), 7.88 (d, J = 7.3 Hz, 2H), 7.99 – 7.75 (m, 2H), 7.72 (d, J = 7.3 Hz, 2H), 7.43 – 7.37 (m, 2H), 7.36 – 7.31 (m, 2H), 4.43 (t, J = 5.1 Hz, 1H), 3.91 (d, J = 4.9 Hz, 2H. ^{13}C NMR (126 MHz, DMSO-d_6) δ 190.1, 152.3, 145.0, 140.7, 137.0, 133.8, 127.7, 127.5, 127.20, 127.22, 124.6, 120.1, 70.1, 68.3, 63.3, 45.7, 31.4. HRMS (ESI) calcd for $C_{28}H_{18}Cl_2N_2OS$ $[M+H]^+$: 501.0590, found: 501.0585

***S*-((9H-fluoren-9-yl)methyl) 4-(bis(1-isopropyl-1H-indol-3-yl)methyl)benzothioate (TE37)**

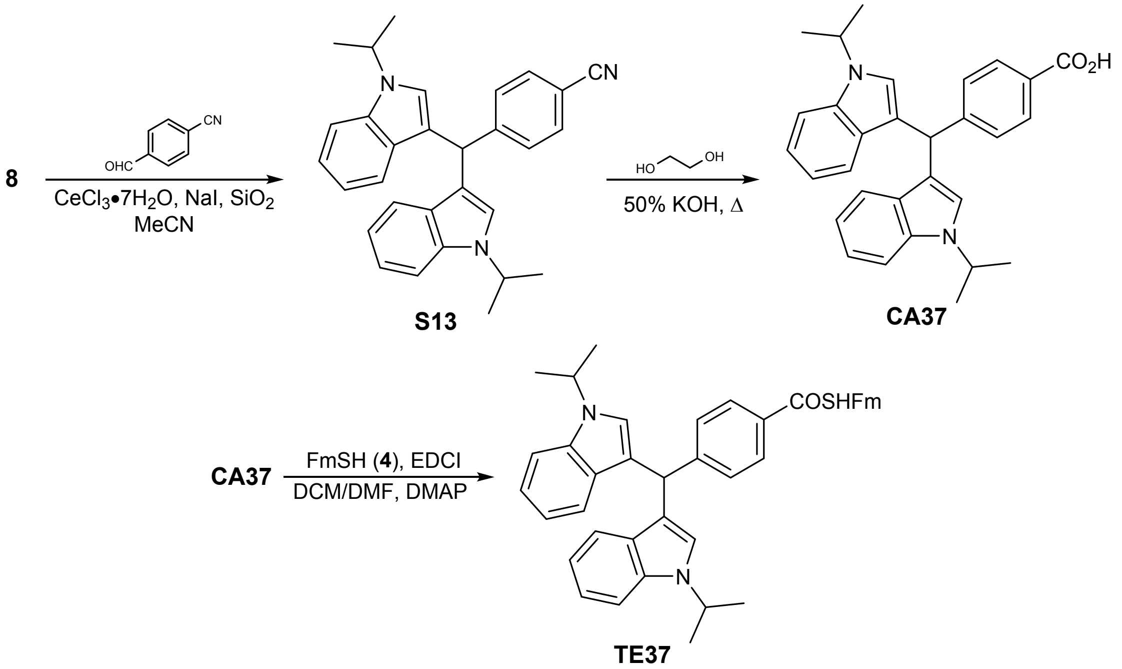

To a round bottom flask was added silica gel (SiO_2, 1.92 g), $CeCl_3$•$7H_2O$ (143 mg, 0.383 mmol), NaI (57.5 mg, 0.383 mmol), MeCN (8.5 mL) and a magnetic stir bar. The mixture was stirred for 12 h and concentrated under reduced pressure resulting in a yellow solid. To the flask was added 1-isopropyl-1*H*-indole (500 mg, 3.14 mmol), 4-formylbenzonitrile (206 mg, 1.57 mmol) and additional MeCN (15 mL). The reaction was stirred for 4 h, diluted with Et_2O, and filtered through Celite. The solvent was removed under reduced pressure and the crude **S13** was purified using flash column chromatograpy. Nitrile **S13** underwent hydrolysis using 50% NaOH (1.5 mL) and ethylene glycol (6.5 mL) at reflux. The reaction mixture was acidified and the precipitate **CA37** recovered using vacuum filtration. Carboxylic acid **CA37** was transformed into **TE37** using the literature procedure.[10] The three synthetic steps gave **TE37** with a 19% overall yield.

4-(bis(1-isopropyl-1H-indol-3-yl)methyl)benzoic acid (CA37) ^{1}H NMR (400 MHz, $CDCl_3$) δ 8.02 (br. s., 2H), 7.46 (d, *J* = 6.3 Hz, 2H), 7.35 (d, *J* = 14.4 Hz, 4H), 7.19 (br. s., 2H), 6.98 (br. s., 2H), 6.71 (br. s., 2H), 5.96 (br. s., 1H), 4.63 (br. s., 2H), 1.44 (br. s., 12H). ^{13}C NMR (101 MHz, $CDCl_3$) δ 173.9, 146.5, 136.0, 135.5, 129.0, 128.1, 127.5, 123.2, 120.9, 120.0, 118.4, 117.8, 109.3, 46.8, 40.3, 22.4, 22.3.

S-((9H-fluoren-9-yl)methyl) 4-(bis(1-isopropyl-1H-indol-3-yl)methyl)benzothioate (TE37) ^{1}H NMR (400 MHz, $CDCl_3$) δ 7.91 (d, *J* = 7.8 Hz, 2H), 7.78 (d, *J* = 7.8 Hz, 4H), 7.46 – 7.32 (m, 10H), 7.24 – 7.18 (m, 2H), 7.04 – 6.98 (m, 2H), 6.73 (s, 2H), 5.95 (s, 1H), 4.71 – 4.58 (m, 2H), 4.28 (t, *J* = 6.1 Hz, 1H), 3.67 (d, *J* = 6.3 Hz, 2H), 1.46 (dd, *J* = 2.5, 6.4 Hz, 12H). ^{13}C NMR (101 MHz, $CDCl_3$) δ 191.4, 150.8, 145.7, 141.0, 136.2, 134.9, 129.0, 127.6, 127.4, 127.3, 127.1, 124.8, 123.2, 121.2, 120.0, 119.9, 118.7, 117.2, 109.5, 47.1, 46.9, 40.7, 32.6, 22.7. HRMS (ESI) calcd for $C_{44}H_{40}N_2OS$ $[M+H]^+$: 645.2934, found: 645.2890

S-((9H-fluoren-9-yl)methyl) 3-(cyclohexylamino)benzothioate (TE38)

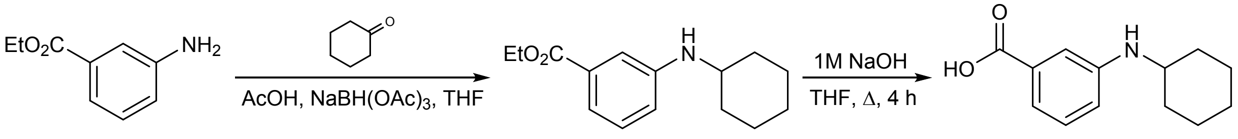

S14 **CA38**

CA38 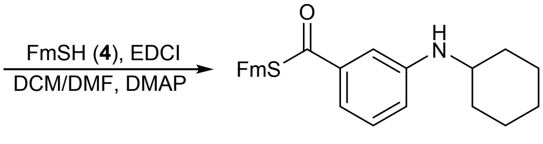

TE38

A round bottom flask was charged with ethyl 3-aminobenzoate (500 mg, 3.03 mmol), cyclohexanone (326 mg, 3.33 mmol), THF (22.5 mL), AcOH (318 mg, 3.63 mmol), a magnetic stir bar and stirred for 4 h. When imine formation was judged complete via TLC, $NaBH(OAc)_3$ (962 mg, 4.54 mmol) was added portion wise and the reaction mass was allowed to stir for 1 h at rt. The reaction was cooled to 0 °C, quenched with saturated $NaHCO_3$, extracted with EtOAc, dried with Na_2SO_4 and solvent removed under reduced pressure. Crude **S14** was purified using flash column chromatography. Ester **S14** was hydrolyzed in 1 M NaOH in refluxing THF for 4 h. The reaction mixture was cooled, acidified with 1 M HCl and allowed to solidfy upon standing. The product **CA38** was collected by vacuum filtration and used without further purification. Carboxylic acid **CA38** was transformed into **TE38** using the literature procedure.[13] The three synthetic steps gave **TE38** with a 19% overall yield.

3-(cyclohexylamino)benzoic acid (CA38) ^{1}H NMR (500 MHz, $CDCl_3$) δ 8.09 (br. s., 1H), 7.43 (d, J = 7.8 Hz, 1H), 7.35 – 7.32 (m, 1H), 7.27 – 7.23 (m, 1H), 6.83 (dd, J = 2.2, 8.1 Hz, 1H), 3.34 (tt, J = 3.6, 10.2 Hz, 1H), 2.12 – 2.04 (m, 2H), 1.79 (td, J = 3.7, 13.6 Hz, 2H), 1.68 (td, J = 3.6, 13.0 Hz, 1H), 1.47 – 1.36 (m, 2H), 1.26 (tt, J = 3.6, 12.4 Hz, 1H), 1.23 – 1.14 (m, 2H). ^{13}C NMR (126 MHz, $CDCl_3$) δ 172.8, 147.3, 130.2, 129.2, 118.7, 118.5, 114.1, 51.6, 33.2, 25.8, 24.8.

S-((9H-fluoren-9-yl)methyl) 3-(cyclohexylamino)benzothioate (TE38) ^{1}H NMR (400 MHz, $CDCl_3$) δ 7.79 – 7.71 (m, 4H), 7.39 (t, J = 7.4 Hz, 2H), 7.35 – 7.29 (m, 2H), 7.26 – 7.21 (m, 1H), 7.20 – 7.14 (m, 1H), 7.11 – 7.08 (m, 1H), 6.76 – 6.71 (m, 1H), 4.24 (t, J = 6.3 Hz, 1H), 3.62 (d, J = 6.3 Hz, 2H), 3.28 (tt, J = 3.8, 10.1 Hz, 1H), 2.08 – 1.99 (m, 2H), 1.75 (td, J = 3.6, 13.5 Hz, 2H), 1.65 (td, J = 3.6, 12.7 Hz, 1H), 1.44 – 1.31 (m, 2H), 1.28 – 1.08 (m, 3H). ^{13}C NMR (101 MHz, $CDCl_3$) δ 192.2, 147.5, 145.7, 141.0, 138.1, 129.3, 127.6, 127.1, 124.8, 119.8, 117.8, 115.8, 110.9, 51.5, 46.9, 33.2, 32.6, 25.8, 24.9. HRMS (ESI) calcd for $C_{27}H_{27}NOS$ $[M+H]^+$: 414.1886, found: 414.1875.

S-((9H-fluoren-9-yl)methyl) 2-(4-(phenylethynyl)phenyl)ethanethioate (TE39)

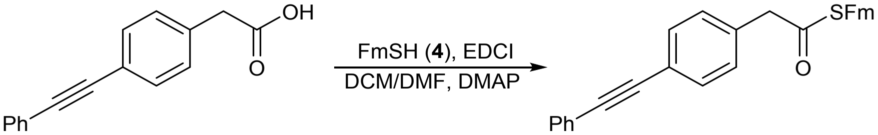

CA39 **TE39**

TE39 was synthesized from **CA39**[17] and thiol **4** using the literature procedure.[10] ^{1}H NMR (400 MHz, $CDCl_3$) δ 7.76 (d, J = 7.4 Hz, 2H), 7.61 (d, J = 7.4 Hz, 2H), 7.58 – 7.54 (m, 2H), 7.48 (d, J

= 7.8 Hz, 2H), 7.44 – 7.38 (m, 2H), 7.38 – 7.35 (m, 2H), 7.34 – 7.28 (m, 3H), 7.16 (d, *J* = 8.2 Hz, 2H), 4.18 (t, *J* = 5.7 Hz, 1H), 3.77 (s, 2H), 3.56 (d, *J* = 5.9 Hz, 2H). ^{13}C NMR (101 MHz, $CDCl_3$) δ 196.3, 145.2, 141.0, 133.7, 131.8, 131.6, 129.3, 128.27, 128.33, 127.7, 127.1, 124.6, 123.2, 122.3, 119.8, 89.7, 89.1, 50.2, 46.7, 32.5. HRMS (ESI) calcd for $C_{29}H_{20}OS$ [M+H]+: 417.1308, found: 417.1319

S-((9H-fluoren-9-yl)methyl) 3-(cyclohexanecarbonyl)-1-methyl-1H-indole-5-carbothioate (TE40)

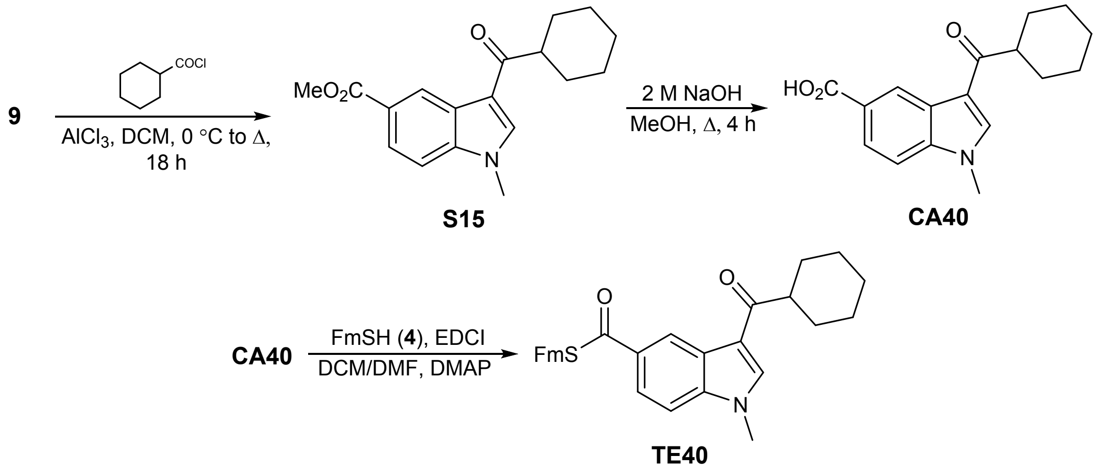

At 0 °C, a round bottom flask was charged with cyclohexanecarbonyl chloride (465 mg, 3.17 mmol), DCM (15 mL), $AlCl_3$ (423 mg, 3.17 mmol) and magnetic stir bar. The reaction was allowed to stir and come to room temperature. Indole **9** (200 mg, 1.06 mmol) was added as a DCM (6 mL) solution over the course of 25 min. The reaction was then stirred for 18 h. The reaction was quenched with saturated NH_4Cl, extracted with DCM, dried with Na_2SO_4, then solvent was removed under reduced pressure. The crude material was purified using flash column chromatography. Ester **S15** was hydrolyzed in 2 M NaOH in refluxing MeOH for 4 h. The reaction mass was cooled, acidified with 1 M HCl and allowed to solidfy upon standing. The product **CA40** was collected by vacuum filtration and used without further purification. Carboxylic acid **CA40** was transformed into **TE40** using the literature procedure.[10] The three synthetic steps gave **TE40** with a 62% overall yield.

3-(cyclohexanecarbonyl)-1-methyl-1H-indole-5-carboxylic acid (CA40) ^1H NMR (400 MHz, DMSO-d_6) δ 12.60 (br. s., 1H), 8.90 (s, 1H), 8.48 (s, 1H), 7.87 (dd, *J* = 1.4, 8.8 Hz, 1H), 7.58 (d, *J* = 9.0 Hz, 1H), 3.88 (s, 3H), 3.11 (t, *J* = 10.3 Hz, 1H), 1.78 (t, *J* = 11.5 Hz, 4H), 1.68 (d, *J* = 12.1 Hz, 1H), 1.52 – 1.30 (m, 4H), 1.26 – 1.14 (m, 1H). ^{13}C NMR (101 MHz, DMSO-d_6) δ 198.4, 168.1, 139.7, 138.7, 125.7, 124.4, 124.1, 123.9, 114.7, 110.4, 46.4, 33.3, 29.6, 25.6, 25.4.

S-((9H-fluoren-9-yl)methyl) 3-(cyclohexanecarbonyl)-1-methyl-1H-indole-5-carbothioate (TE40) ^1H NMR (500 MHz, $CDCl_3$) δ 9.09 (d, *J* = 1.5 Hz, 1H), 7.92 (dd, *J* = 1.7, 8.6 Hz, 1H), 7.80 (d, *J* = 7.3 Hz, 2H), 7.78 – 7.75 (m, 3H), 7.42 – 7.38 (m, 2H), 7.37 – 7.31 (m, 3H), 4.27 (t, *J* = 6.4 Hz, 1H), 3.85 (s, 3H), 3.68 (d, *J* = 6.4 Hz, 2H), 3.00 (tt, *J* = 3.0, 11.7 Hz, 1H), 1.96 – 1.85 (m, 4H), 1.77 (d, *J* = 12.2 Hz, 1H), 1.68 – 1.58 (m, 2H), 1.46 – 1.35 (m, 2H), 1.35 – 1.28 (m, 1H). ^{13}C NMR (126 MHz, $CDCl_3$) δ 199.2, 191.7, 145.8, 140.9, 140.1, 136.2, 131.8, 127.6, 127.1, 126.0, 124.8, 123.5, 122.2, 119.8, 116.5, 109.6, 48.0, 47.0, 33.7, 32.8, 29.8, 26.0, 25.9. HRMS (ESI) calcd for $C_{31}H_{29}NO_2S$ $[M+H]^+$: 480.1992, found: 480.1980

S-((9H-fluoren-9-yl)methyl) 2-phenylbenzofuran-5-carbothioate (TE41)

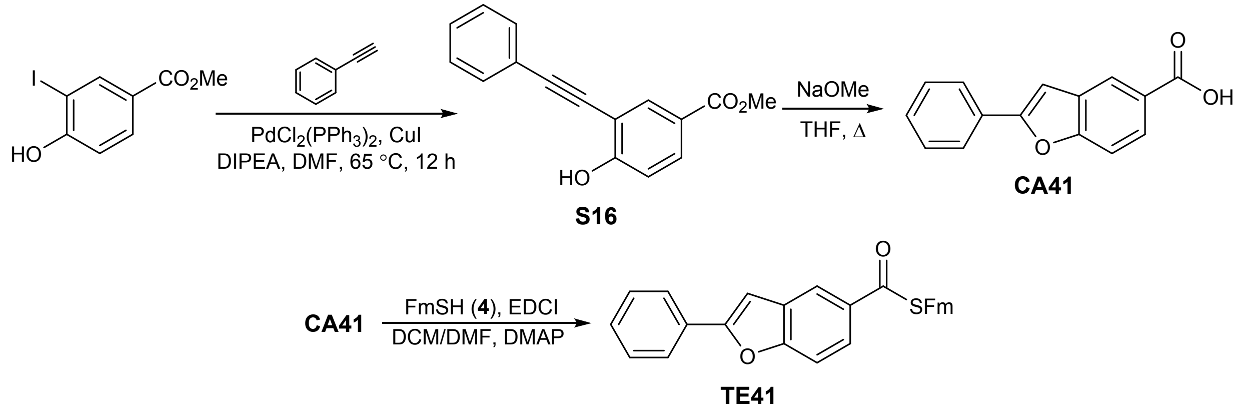

A flame dried flask was charged with previously reported[18] methyl 4-hydroxy-3-iodobenzoate (500 mg, 1.80 mmol), ethynylbenzene (790 mg, 7.73 mmol), $Pd(PPh_3)_4$ (125 mg, 6 mol%), CuI (62 mg, 18 mol%), DIPEA (582 mg, 4.50 mmol), DMF (20 mL), a magnetic stir bar, purged with argon and heated to 65°C for 12 h. The reaction was quenched with NH_4Cl, extracted with EtOAc, then the solvent was removed under reduced pressure and the product purified using flash column chromatography. Following a reported procedure,[19] hydroxy alkyne **S16** then underwent cyclization to the benzofuran along with concomitant hydrolysis of the ester function using NaOMe, yielding **CA41** which was used without further purification. Carboxylic acid **CA41** was transformed into **TE41** using the literature procedure.[10] The three synthetic steps gave **TE41** with a 27% overall yield (Rf = 0.48 in hexanes : EtOAc = 85 : 10).

2-phenylbenzofuran-5-carboxylic acid (CA41)[20]

S-((9H-fluoren-9-yl)methyl) 2-phenylbenzofuran-5-carbothioate (TE41) ^{1}H NMR (250 MHz, $CDCl_3$) δ 8.12 (s, 1H), 7.71 (ddd, J = 34.0, 17.8, 4.5 Hz, 7H), 7.44 – 7.21 (m, 8H), 6.95 (s, 1H), 4.20 (t, J = 6.0 Hz, 1H), 3.62 (d, J = 6.1 Hz, 2H). ^{13}C NMR (101 MHz, $CDCl_3$) δ 157.7, 145.7, 141.1, 134.7, 132.6, 129.7, 129.3, 128.9, 127.7, 127.1, 125.1, 124.8, 124.3, 123.9, 120.9, 120.3, 119.9, 111.2, 101.5, 46.9, 32.8. HRMS (ESI) calcd for $C_{29}H_{20}O_2S$ $[M+H]^+$: 433.1257, found: 433.1236

S*-((9*H*-fluoren-9-yl)methyl) 4-((3,4-bis(2,4-difluorophenyl)-2,5-dioxo-2,5-dihydro-1*H*-pyrrol-1-yl)methyl)benzothioate (TE42)

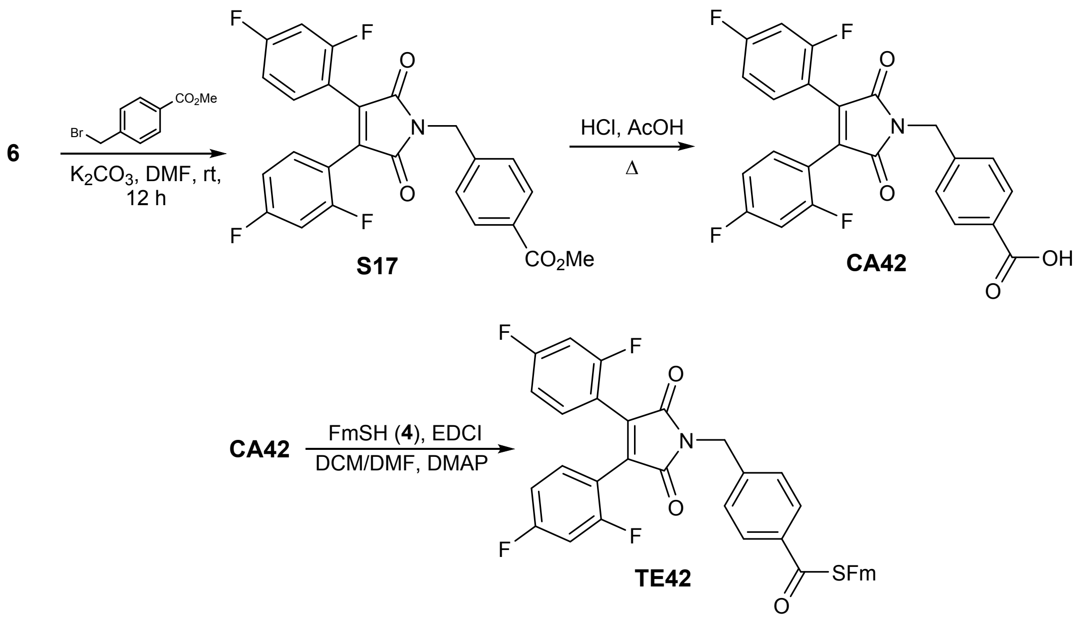

A round bottom flask was charged with **6** (250 mg, 0.78 mmol), methyl 4-(bromomethyl)benzoate (179 mg, 0.78 mmol), K_2CO_3 (215 mg, 1.56 mmol), DMF (8 mL), a magnetic stir bar and stirred at rt for 12 h. The reaction was quenched with water, extracted with EtOAc, washed with brine, dried with Na_2SO_4, solvent removed under reduced pressure and purified using flash column chromatography. The ester function of **S17** was hydrolyzed using HCl/AcOH (1:1, 6 mL) under reflux conditions. The product was filtered and washed with water to neutrality and used without further purification. Carboxylic acid **CA42** was transformed into **TE42** using the literature procedure.[10] The four synthetic steps gave **TE42** with a 72% overall yield.

4-((3,4-bis(2,4-difluorophenyl)-2,5-dioxo-2,5-dihydro-1H-pyrrol-1-yl)methyl)benzoic acid (CA42) ^{1}H NMR (500 MHz, DMSO-d$_6$) δ 12.94 (br. s., 1H), 7.93 (d, *J* = 8.3 Hz, 2H), 7.60 – 7.54 (m, 2H), 7.49 (d, *J* = 7.8 Hz, 2H), 7.38 – 7.31 (m, 2H), 7.23 (dt, *J* = 2.0, 8.6 Hz, 2H), 4.83 (s, 2H). ^{13}C NMR (126 MHz, DMSO-d$_6$) δ 168.7, 167.0, 162.6 (dd, *J* = 252, 14 Hz), 160.6 (dd, J = 253, 14 Hz), 141.3, 133.5, 133.0 (d, *J* = 5 Hz), 130.0, 129.6, 127.6, 113.5 (d, *J* = 15 Hz), 112.0 (d, *J* = 23 Hz), 104.7 (t, *J* = 26 Hz), 41.4.

S-((9H-fluoren-9-yl)methyl) 4-((3,4-bis(2,4-difluorophenyl)-2,5-dioxo-2,5-dihydro-1H-pyrrol-1-yl)methyl)benzothioate (TE42) ^{1}H NMR (500 MHz, $CDCl_3$) δ 7.92 (d, *J* = 8.3 Hz, 2H), 7.76 (d, *J* = 7.8 Hz, 2H), 7.72 (d, *J* = 7.3 Hz, 2H), 7.54 – 7.47 (m, 4H), 7.43 – 7.37 (m, 2H), 7.36 – 7.30 (m, 2H), 7.01 – 6.94 (m, 2H), 6.84 – 6.78 (m, 2H), 4.85 (s, 2H), 4.26 (t, *J* = 6.1 Hz, 1H), 3.68 (d, *J* = 5.9 Hz, 2H). ^{13}C NMR (101 MHz, $CDCl_3$) δ 191.0, 169.0, 163.8 (dd, *J* = 254, 12 Hz), 161.1 (dd, *J* = 258, 14 Hz), 145.5, 141.2, 141.0, 136.7, 133.3, 132.4 (dd, *J* = 10, 5 Hz), 128.9, 127.73, 127.69, 127.1, 124.7, 119.9, 112.0 (dd, *J* = 22, 4 Hz), 111.8, 104.7 (t, *J* = 25 Hz), 46.7, 41.9, 32.6. HRMS (ESI) calcd for $C_{38}H_{23}F_4NO_3S$ $[M+H]^+$: 650.1408, found: 650.1425

S-((9H-fluoren-9-yl)methyl) 4-(1-(isoxazole-5-carbonyl)piperidin-4-yl)benzothioate (TE43)

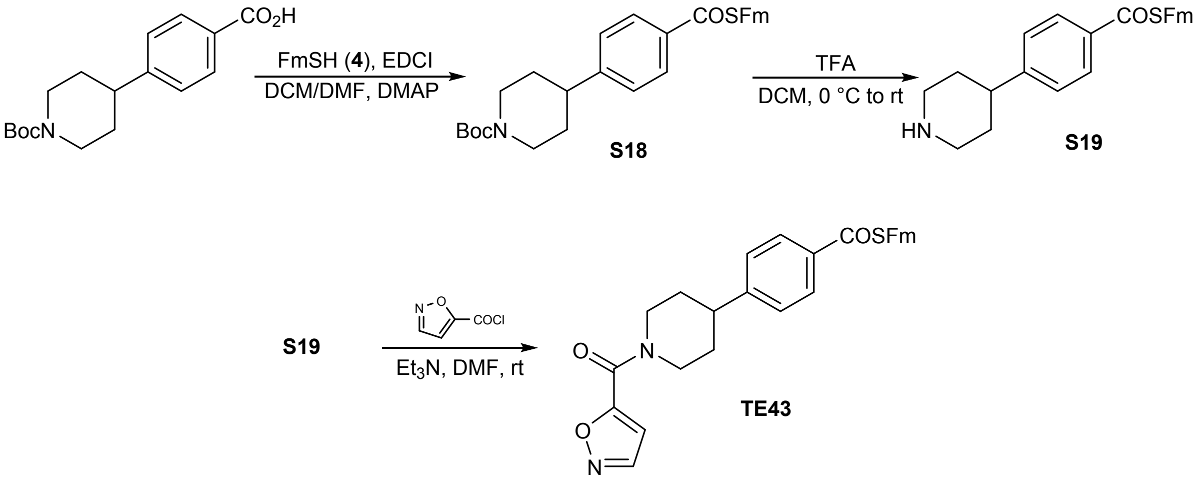

Commercially available 4-(1-(tert-butoxycarbonyl)piperidin-4-yl)benzoic acid was transformed into **S18** using the literature procedure. Deprotection of the secondary amine was accomplished using TFA in DCM resulting in **S19** which was subsequently transformed into **TE43** by acylating the secondary amine with isoxazole-5-carbonyl chloride in the presence of TEA and DMF. The three synthetic steps gave **TE43** with 32 % overall yield.

S-((9H-fluoren-9-yl)methyl) 4-(1-(isoxazole-5-carbonyl)piperidin-4-yl)benzothioate (TE43) ^{1}H NMR (400 MHz, $CDCl_3$) δ 8.34 (s, 1H), 7.89 (d, J = 8.2 Hz, 2H), 7.76 (d, J = 7.4 Hz, 2H), 7.74 (d, J = 7.4 Hz, 2H), 7.40 (t, J = 7.4 Hz, 2H), 7.36 – 7.31 (m, 2H), 7.27 (d, J = 8.6 Hz, 2H), 6.81 (s, 1H), 4.84 (d, J = 12.5 Hz, 1H), 4.31 (d, J = 14.1 Hz, 1H), 4.26 (t, J = 6.1 Hz, 1H), 3.68 (d, J = 6.3 Hz, 2H), 3.28 (t, J = 12.5 Hz, 1H), 2.99 – 2.82 (m, 2H), 1.98 (t, J = 14.8 Hz, 2H), 1.77 (q, J = 12.5 Hz, 2H). ^{13}C NMR (101 MHz, $CDCl_3$) δ 190.9, 163.7, 156.8, 150.3, 150.1, 145.4, 140.9, 135.4, 127.6, 127.5, 127.0, 126.8, 124.6, 119.8, 107.2, 47.0, 46.7, 43.3, 42.5, 33.4, 32.4, 32.3. HRMS (ESI) calcd for $C_{30}H_{26}N_2O_3S$ $[M+H]^+$: 495.1737, found: 495.1722

S-((9H-fluoren-9-yl)methyl) 3-(phenethylamino)benzothioate (TE44)

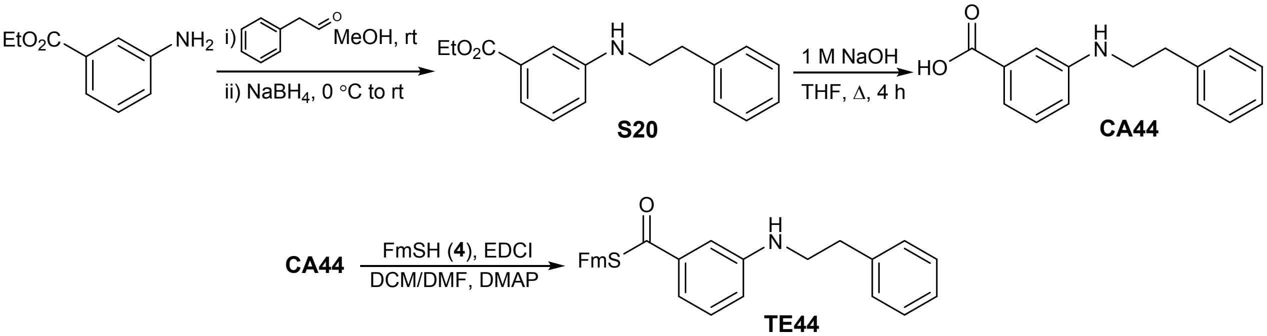

A round bottom flask was charged with ethyl 3-aminobenzoate (500 mg, 3.03 mmol), 2-phenylacetaldehyde (343 mg, 2.85 mmol), MeOH (14 mL), a magnetic stir bar and stirred for 4 h. When imine formation was judged complete via TLC, $NaBH_4$ (174 mg, 4.60 mmol) was added portion-wise, and the reaction mass was allowed to stir for 1 h at rt. The reaction was cooled to 0 °C, quenched with saturated NH_4Cl, extracted with EtOAc, dried with Na_2SfO_4 and solvent removed under reduced pressure. Crude **S20** was purified using flash column chromatography.

Ester **S20** was hydrolyzed in 1 M NaOH in refluxing THF for 4 h. The reaction mass was cooled, acidified with 1 M HCl and allowed to solidfy upon standing. The product **CA44** was collected by vacuum filtration and used without further purification. Carboxylic acid **CA44** was transformed into **TE44** using the literature procedure.[10] The three synthetic steps gave **TE44** with a 12% overall yield.

3-(phenethylamino)benzoic acid (CA44) ^{1}H NMR (400 MHz, DMSO-d$_6$) δ 7.33 – 7.24 (m, 5H), 7.19 (br. s., 3H), 6.83 (br. s., 1H), 5.95 (br. s., NH), 3.28 (br. s., 2H), 2.85 (br. s., 2H). ^{13}C NMR (101 MHz, DMSO-d$_6$) δ 168.1, 148.8, 139.8, 131.6, 129.1, 128.7, 128.3, 126.1, 116.7, 116.1, 112.8, 44.6, 34.8.

S-((9H-fluoren-9-yl)methyl) 3-(phenethylamino)benzothioate (TE44) ^{1}H NMR (400 MHz, CDCl$_3$) δ 7.78 (dd, *J* = 2.7, 7.8 Hz, 4H), 7.45 – 7.39 (m, 2H), 7.38 – 7.32 (m, 4H), 7.32 – 7.27 (m, 2H), 7.26 – 7.20 (m, 3H), 7.17 – 7.15 (m, 1H), 6.80 – 6.76 (m, 1H), 4.28 (t, *J* = 6.3 Hz, 1H), 3.67 (d, *J* = 5.9 Hz, 2H), 3.45 (t, *J* = 7.0 Hz, 2H), 2.94 (t, *J* = 7.0 Hz, 2H). ^{13}C NMR (101 MHz, CDCl$_3$) δ 192.1, 148.1, 145.7, 141.0, 138.9, 138.0, 129.4, 128.7, 128.6, 127.6, 127.1, 126.5, 124.8, 119.8, 117.8, 116.4, 110.6, 46.8, 44.8, 35.3, 32.6. HRMS (ESI) calcd for $C_{29}H_{25}NOS$ $[M+H]^+$: 436.1730, found: 436.1724

S-((9H-fluoren-9-yl)methyl) 1-methyl-3-(thiophene-2-carbonyl)-1H-indole-5-carbothioate (TE45)

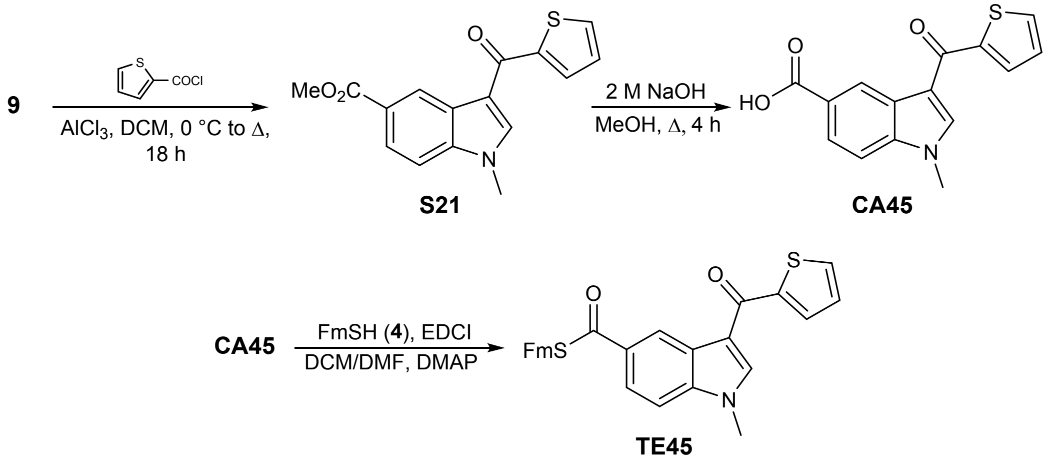

At 0 °C a round bottom flask was charged with thiophene-2-carbonyl chloride (232 mg, 1.59 mmol), DCM (7.5 mL), $AlCl_3$ (212 mg, 1.59 mmol) and magnetic stir bar. The reaction was allowed to stir and come to rt. Indole **9** (100 mg, 0.53 mmol) was added as a DCM (3 mL) solution over the course of 25 min. The reaction was then stirred for 18 h. The reaction was quenched with saturated NH_4Cl, extracted with DCM, dried with Na_2SO_4, solvent removed under reduced pressure and purified using flash column chromatography. Ester **S21** was hydrolyzed in 2 M NaOH in refluxing MeOH for 4 h. The reaction mass was cooled, acidified with 1 M HCl and allowed to solidfy upon standing. The product **CA45** was collected by vacuum filtration and used without further purification. Carboxylic acid **CA45** was transformed into **TE45** using the literature procedure.[10] The three synthetic steps gave **TE45** with a 36% overall yield.

1-methyl-3-(thiophene-2-carbonyl)-1H-indole-5-carboxylic acid (CA45) ^{1}H NMR (400 MHz, DMSO-d$_6$) δ 8.94 (s, 1H), 8.48 (s, 1H), 8.00 – 7.92 (m, 3H), 7.59 (d, *J* = 8.6 Hz, 1H), 7.27 (t, *J* =

4.1 Hz, 1H), 3.94 (s, 3H). ^{13}C NMR (101 MHz, DMSO-d_6) δ 180.2, 168.8, 145.0, 139.2, 139.1, 132.7, 131.6, 128.3, 126.8, 126.2, 124.5, 123.8, 114.1, 110.3, 33.4.

S-((9H-fluoren-9-yl)methyl) 1-methyl-3-(thiophene-2-carbonyl)-1H-indole-5-carbothioate (TE45) ^{1}H NMR (400 MHz, $CDCl_3$) δ 9.01 (d, *J* = 2.0 Hz, 1H), 7.83 (dd, *J* = 1.6, 8.6 Hz, 1H), 7.80 (d, *J* = 7.4 Hz, 2H), 7.76 (d, *J* = 7.4 Hz, 2H), 7.71 (s, 1H), 7.68 – 7.66 (m, 1H), 7.61 (dd, *J* = 0.8, 5.1 Hz,1H), 7.43 – 7.38 (m, 2H), 7.38 – 7.32 (m, 2H), 7.21 (d, *J* = 8.6 Hz, 1H), 7.13 (dd, *J* = 3.9, 4.7 Hz, 1H), 4.28 (t, *J* = 6.3 Hz, 1H), 3.78 (s, 3H), 3.71 (d, *J* = 6.3 Hz, 2H). ^{13}C NMR (101 MHz, $CDCl_3$) δ 191.4, 180.8, 145.8, 144.8, 141.0, 139.8, 137.0, 131.8, 131.6, 131.2, 127.7, 127.6, 127.1, 126.5, 124.8, 123.0, 122.5, 119.8, 116.2, 109.7, 47.0, 33.7, 32.6. HRMS (ESI) calcd for $C_{29}H_{21}NO_2S_2$ $[M+H]^+$: 480.1087, found: 480.1062

Synthesis of Sulfonyl Azides

Compounds **SZ1-SZ38** are the sulfonyl azides that were used in this study for the sulfo-click reactions. The synthesis of these compounds and their intermediates are as follows. Sulfonyl azides **SZ1-SZ11, SZ16, SZ21, SZ23, SZ28** have been previously reported.

4-((4-phenylpiperazin-1-yl)methyl)benzenesulfonyl azide (SZ1)[2]

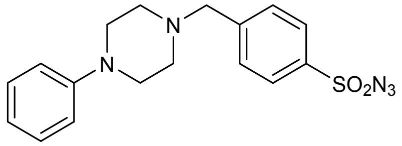

4-((methyl(phenethyl)amino)methyl)benzenesulfonyl azide (SZ2)[2]

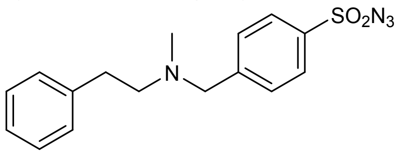

4-acetamidobenzenesulfonyl azide (SZ3)[2]

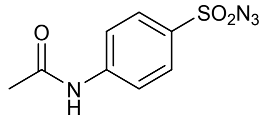

3-nitro-4-((2-(phenylthio)ethyl)amino)benzenesulfonyl azide (SZ4)[2]

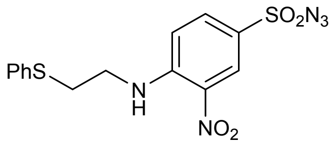

4-methylbenzenesulfonyl azide (SZ5)[2]

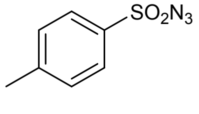

4-(((2-(phenylthio)ethyl)amino)methyl)benzenesulfonyl azide (SZ6)[2]

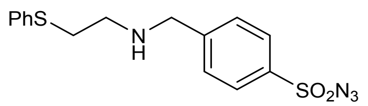

7-((2-(phenylthio)ethyl)amino)naphthalene-2-sulfonyl azide (SZ7)[12]

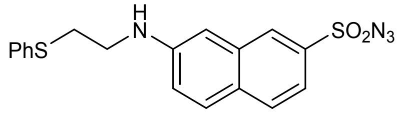

4-((6,7-dimethoxy-1-phenyl-3,4-dihydroisoquinolin-2(1H)-yl)methyl)benzenesulfonyl azide (SZ8)[12]

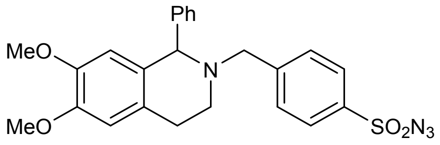

4,4'-(((2-(phenylthio)ethyl)azanediyl)bis(methylene))dibenzenesulfonyl azide (SZ9)[12]

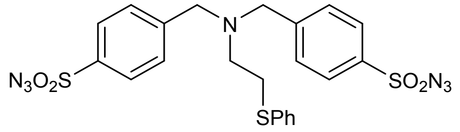

4-(((2-(phenylthio)ethyl)(4-sulfamoylbenzyl)amino)methyl)benzenesulfonyl azide (SZ10)[12]

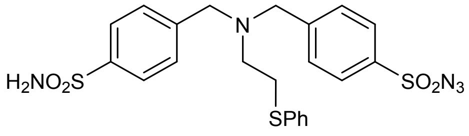

4'-(tert-butyl)-[1,1'-biphenyl]-4-sulfonyl azide (SZ11)[11]

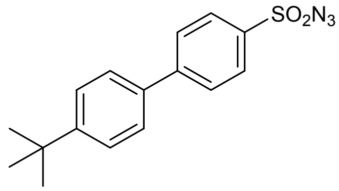

4-(((3-nitrophenyl)amino)methyl)benzenesulfonyl azide (SZ12)

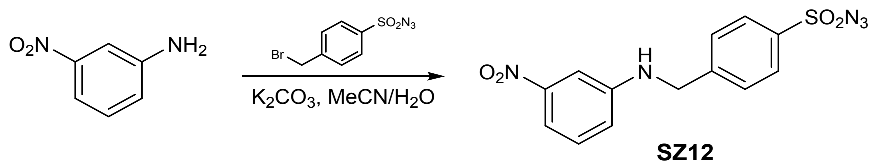

SZ12

A round bottom flask was charged with 3-nitroaniline (138 mg, 1.00 mmol), 4-(bromomethyl)benzenesulfonyl azide (275 mg, 1.00 mmol), K_2CO_3 (276 mg, 2.00 mmol), MeOH/$H_2$0 (9:1, 12.5 mL), a magnetic stir bar and stirred at rt for 12 h. The reaction was judged to be complete via TLC, quenched with water, extracted with EtOAc, dried over Na_2SO_4 and concentrated under reduced pressure. The crude residue was purified using flash column chromatography and **SZ12** was obtained in 67% yield. R_f = 0.35 in hexanes : EtOAc = 3:1. ^{1}H NMR (600 MHz, $CDCl_3$) δ 7.94 (d, J = 8.3 Hz, 2H), 7.61 (d, J = 8.2 Hz, 2H), 7.57 (dd, J = 8.1, 1.8 Hz, 1H), 7.40 (t, J = 2.1 Hz, 1H), 7.30 (t, J = 8.2 Hz, 1H), 6.86 (dd, J = 8.2, 2.2 Hz, 1H), 4.64 (s, 1H), 4.56 (s, 2H). ^{13}C NMR (101 MHz, $CDCl_3$) δ: 148.2, 146.4, 130.2, 128.4, 128.3, 128.2, 127.8,

118.9, 113.0, 106.9, 47.5. HRMS (ESI) calcd for $C_{13}H_{11}N_5O_4S$ $[M+K]^+$: 372.0163, found: 372.0145.

4-((2-nitrophenoxy)methyl)benzenesulfonyl azide (SZ13)

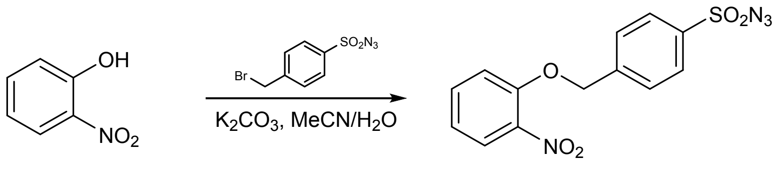

SZ13

A round bottom flask was charged with 2-nitrophenol (139 mg, 1.00 mmol), 4-(bromomethyl)benzenesulfonyl azide (275 mg, 1.00 mmol), K_2CO_3 (276 mg, 2.00 mmol), MeOH/H_20 (9:1, 12.5 mL), a magnetic stir bar and stirred at rt for 12 h. The reaction was judged to be complete via TLC, quenched with water, extracted with EtOAc, dried over Na_2SO_4 and concentrated under reduced pressure. The crude residue was purified using flash column chromatography and **SZ13** was obtained in 38% yield. $R_f = 0.44$ in hexanes : EtOAc = 3:1. ^1H NMR (400 MHz, $CDCl_3$) δ: 7.94 – 7.84 (m, 3H), 7.68 – 7.34 (m, 5H), 4.50 (s, 2H). HRMS (ESI) calcd for $C_{13}H_{10}N_4O_5S$ $[M+NH_4]^+$: 352.0716, found: 352.0719.

4-(((4-oxo-2-phenyl-4H-chromen-7-yl)oxy)methyl)benzenesulfonyl azide (SZ14)

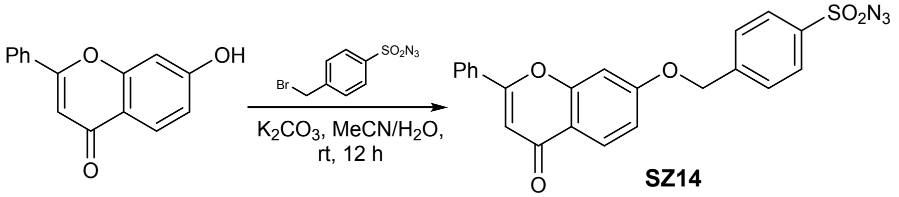

SZ14

A round bottom flask was charged with 7-hydroxy-2-phenyl-4*H*-chromen-4-one (238 mg, 1.00 mmol), 4-(bromomethyl)benzenesulfonyl azide (276 mg, 1.00 mmol), K_2CO_3 (276 mg, 2.00 mmol), MeOH/H_20 (9:1, 12.5 mL), a magnetic stir bar and stirred at rt for 12 h. The reaction was judged to be complete via TLC, quenched with water, extracted with EtOAc, dried over Na_2SO_4 and concentrated under reduced pressure. The crude residue was purified using flash column chromatography and **SZ14** was obtained in 45 % yield. $R_f = 0.21$ in hexanes : EtOAc = 3:1. ^1H NMR (400 MHz, $CDCl_3$) δ: 8.10 (dd, J = 8.6, 3.4 Hz, 1H), 7.91 – 7.82 (m, 4H), 7.51 (d, J = 7.4 Hz, 5H), 7.38 (d, J = 2.1 Hz, 1H), 6.93 (dd, J = 8.7, 1.9 Hz, 1H), 6.77 (d, J = 3.8 Hz, 1H) 4.49 (s, 2H). ^{13}C NMR (101 MHz, $CDCl_3$) δ: 177.2, 163.9, 156.5, 152.9, 143.0, 134.6, 132.0, 131.2, 129.2, 129.0, 128.7, 127.6, 126.3, 122.8, 119.5, 112.2, 107.7, 53.8. HRMS (ESI) calcd for $C_{22}H_{15}N_3O_5S$ $[M+H]^+$: 434.0805, found: 434.0796.

4-(((2-chloro-6-fluorobenzyl)(2-(phenylthio)ethyl)amino)methyl)benzenesulfonyl azide (SZ15)

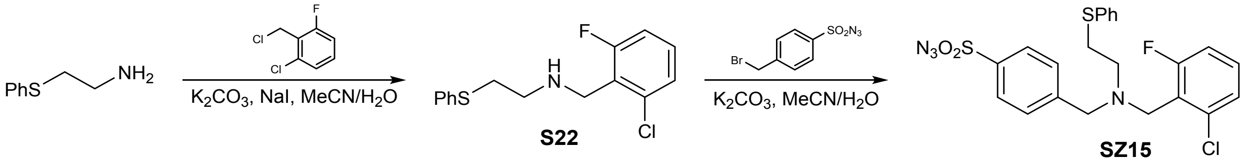

A round bottom flask was charged with amine **3** (153 mg, 1.00 mmol), 1-chloro-2-(chloromethyl)-3-fluorobenzene (238 mg, 1.00 mmol), K_2CO_3 (276 mg, 2.00 mmol), MeOH/H_20 (9:1, 12.5 mL), a magnetic stir bar and stirred at rt for 12 h. The reaction was judged to be complete via TLC, quenched with water, extracted with EtOAc, dried over Na_2SO_4 and concentrated under reduced pressure. The crude residue was purified using flash column chromatography to obtain **S22** in 43% yield. A round bottom was then charged with **S22** (199 mg, 0.67 mmol), 4-(bromomethyl)benzenesulfonyl azide (186 mg, 0.67 mmol), K_2CO_3 (186 mg, 1.35 mmol), MeOH/H_2O (9:1, 4.5 mL), a magnetic stir bar and stirred at rt for 12 h. The reaction was then quenched with water, extracted with EtOAc, dried over Na_2SO_4 and concentrated under reduced pressure. The crude residue was purified using flash column chromatography to obtain **SZ15** in 45% yield. $R_f = 0.62$ in hexanes : EtOAc = 5:1. ^{1}H NMR (400 MHz, $CDCl_3$) δ: 7.80 (d, $J = 8.1$ Hz, 2H), 7.54 (d, $J = 8.1$ Hz, 2H), 7.22 – 7.11 (m, 7H), 6.96 – 6.89 (m, 1H), 3.87 (s, 2H), 3.75 (s, 2H), 3.07 (t, $J = 7.6$ Hz, 2H), 2.79 (t, $J = 7.6$ Hz, 2H). ^{13}C NMR (101 MHz, $CDCl_3$) δ: 162.1 (d, $J = 249.0$ Hz), 147.9, 136.8, 136.5, 136.4, 136.0, 129.6, 129.5, 129.1 (d, $J = 26.0$ Hz), 127.3, 126.1, 125.6, 124.2 (d, $J = 16.8$ Hz), 114.1 (d, $J = 23.3$ Hz), 58.0, 53.7, 49.6, 31.3. HRMS (ESI) calcd for $C_{22}H_{20}ClFN_4O_2S_2$ $[M+H]^+$: 491.0779, found: 491.0771.

4'-(trifluoromethyl)-[1,1'-biphenyl]-4-sulfonyl azide (SZ16)[11]

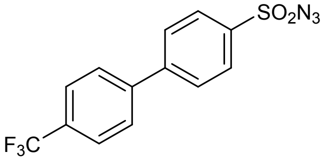

Synthesis of 4-(((3-methoxybenzyl)(2-(phenylthio)ethyl)amino)methyl)benzenesulfonyl azide (SZ17)

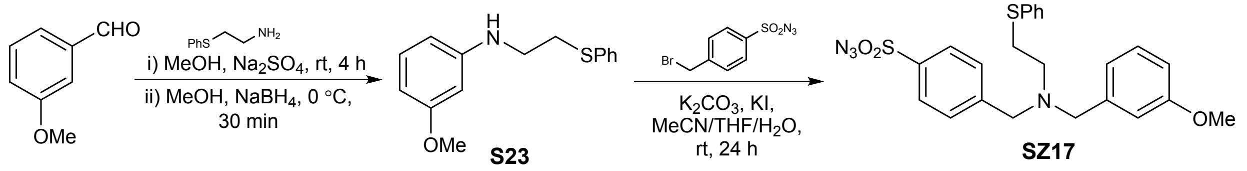

A round bottom flask was charged with 3-methoxybenzaldehyde (267 mg, 1.96 mmol), 2-(phenylthio)ethanamine (300 mg, 1.96 mmol), Na_2SO_4 (556 mg, 3.92 mmol), MeOH (5.0 mL), a magnetic stir bar, and was stirred for 4 h. $NaBH_4$ (222 mg, 5.88 mmol) was added portion wise at 0 °C and stirred for an additional 30 min. The reaction was quenched with water, extracted with CH_2Cl_2, dried with Na_2SO_4, solvent removed under reduced pressure and purified using flash column chromatography resulting in pure **S23**. The secondary amine **S23** (477 mg, 1.74 mmol) was added to a round bottom flask along with 4-(bromomethyl)benzenesulfonyl azide (434 mg, 1.57 mmol), K_2CO_3 (602 mg, 4.35 mmol), KI (145 mg, 0.87 mmol), MeOH/THF/H_2O (22:3:1, 22 mL), a magnetic stir bar and stirred at rt for 24 h. The reaction was judged to be complete via TLC,

quenched with water, extracted with EtOAc, dried over Na_2SO_4 and concentrated under reduced pressure. The crude residue was purified using flash column chromatography. The two synthetic steps gave sulfonyl azide **SZ17** in 77% yield. R_f = 0.43 in hexanes : EtOAc = 4:1. ^{1}H NMR (400 MHz, $CDCl_3$) δ: 7.86 (d, J = 8.3 Hz, 2H), 7.61 (d, J = 8.2 Hz, 2H), 7.28 – 7.12 (m, 6H), 7.00 – 6.95 (m, 2H), 6.82 (dd, J = 8.1, 1.7 Hz, 1H), 3.81 (s, 3H), 3.69 (s, 2H), 3.63 (s, 2H), 3.07 (t, J = 7.2 Hz, 2H), 2.77 (t, J = 7.2 Hz, 2H). ^{13}C NMR (101 MHz, $CDCl_3$) δ: 159.7, 147.8, 140.2, 136.7, 136.2, 129.6, 129.3, 128.8, 128.7, 127.4, 125.8, 121.0, 114.3, 112.5, 58.4, 57.7, 55.1, 52.8, 31.3. HRMS (ESI) calcd for $C_{23}H_{24}N_4O_3S_2$ $[M+H]^+$: 469.1368, found: 469.1378.

Synthesis of 4-chlorobenzenesulfonyl azide (SZ18)[21]

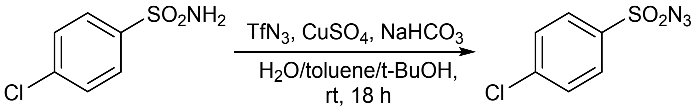

SZ18

An Erlenmeyer flask was charged with 4-chlorobenzenesulfonamide (100 mg, 0.52 mmol), $NaHCO_3$ (175 mg, 2.09 mmol), water (0.6 mL), 1 M $CuSO_4$ (0.02 mL of a 1 M solution, 4 mol%), a magnetic stir bar and stirred at rt. To the flask a freshly prepared solution of TfN_3 (1.0 mL of a 0.75 M solution, 0.78 mmol) in toluene followed by *t*-BuOH (4.0 mL) was added and stirred behind a blast shield for 18 h. The reaction mass was transferred to a round bottom flask and diluted with xylenes. Volatile solvents were removed under reduced pressure, the product precipitated and the solids recovered using vacuum filtration. The crude residue was purified using flash column chromatography and **SZ18** was obtained in 93% yield.

Synthesis of 4-methoxybenzenesulfonyl azide (SZ19)[22]

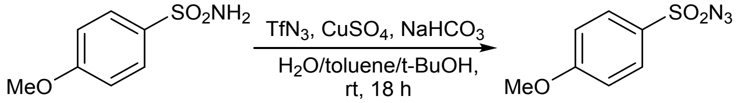

SZ19

The procedure for the synthesis of **SZ18** was used for the synthesis of **SZ19**. Yield = 80%.

Synthesis of 2-methylbenzenesulfonyl azide (SZ20)[23]

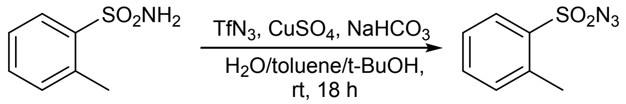

SZ20

The procedure for the synthesis of **SZ18** was used for the synthesis of **SZ20**. Yield = 89%.

5-(dimethylamino)naphthalene-1-sulfonyl azide (SZ21)[11]

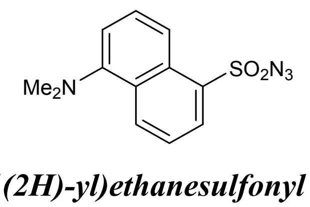

Synthesis of 2-(octahydroquinolin-1(2H)-yl)ethanesulfonyl azide (SZ22)

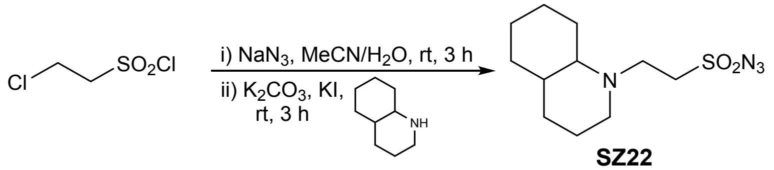

SZ22

A round bottom flask was charged with 2-chloro-ethanesulfonyl chloride (200 mg, 1.23 mmol), MeCN (4.0 mL) a magnetic stir bar and stirred at rt. To the reaction, via addition funnel, was added a saturated solution of NaN_3 (79.7 mg, 1.23 mmol) in water and the reaction allowed to stir while protected from light for 3 h. Then, a solution of a secondary amine (decahydroquinoline (215 mg, 1.23 mmol)) in $MeCN/H_2O$ (9:1, 2.5 mL), K_2CO_3 (340 mg, 2.45 mmol) and KI (41 mg, 0.25 mmol) were added, and the reaction stirred while protected from light for 3 h. The reaction was quenched with water, extracted with DCM, dried using Na_2SO_4, and the solvent was removed under reduced pressure. The crude residue was purified using flash column chromatography and **SZ22** was obtained in 15% yield. $R_f = 0.63$ in hexanes : EtOAc = 2:1 (stained with Ninhydrin). ^1H NMR (400 MHz, $CDCl_3$) δ 3.56 – 3.47 (m, 1H), 3.43 – 3.25 (m, 2H), 2.96 – 2.83 (m, 2H), 2.22 – 2.13 (m, 1H), 2.04 – 1.98 (m, 1H), 1.84 – 1.68 (m, 2H), 1.67 – 1.53 (m, 5H), 1.28 – 1.06 (m, 4H), 1.03 – 0.89 (m, 2H). ^{13}C NMR (101 MHz, $CDCl_3$) δ 66.2, 54.0, 52.7, 46.8, 42.0, 33.1, 32.4, 30.2, 25.9, 25.8, 25.7. HRMS (ESI) calcd for $C_{11}H_{20}N_4O_2S$ $[M+H]^+$: 273.1380, found: 273.1375.

2-(3,4-dihydroisoquinolin-2(1H)-yl)ethane-1-sulfonyl azide (SZ23)[11]

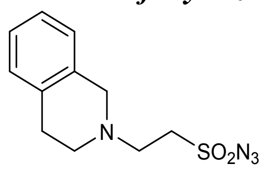

Synthesis of 2-(4-benzylpiperidin-1-yl)ethanesulfonyl azide (SZ24)

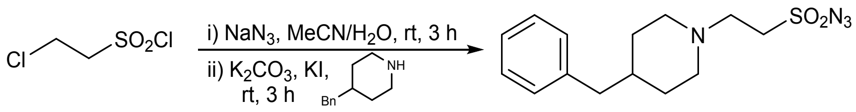

SZ24

The procedure for the synthesis of **SZ22** was used for the synthesis of **SZ24**. Yield = 26%. R_f = 0.73 in hexanes : EtOAc = 1:1. ^1H NMR (400 MHz, $CDCl_3$) δ 7.32 – 7.11 (m, 5H), 3.47 (t, J = 6.3 Hz, 2H), 2.91 (d, J = 11.1 Hz, 2H), 2.83 (t, J = 6.3 Hz, 2H), 2.52 (d, J = 7.0 Hz, 2H), 1.97 (m, 2H), 1.66 (d, J = 12.6 Hz, 2H), 1.60 – 1.48 (m, 1H), 1.38 – 1.23 (m, 2H). ^{13}C NMR (101 MHz, $CDCl_3$) δ 140.5, 129.2, 128.3, 126.0, 53.9, 53.8, 52.5, 43.2, 37.8, 32.2. HRMS (ESI) calcd for $C_{14}H_{20}N_4O_2S$ $[M+H]^+$: 309.1380, found: 309.1373.

Synthesis of 2-(4-(benzo[d][1,3]dioxol-5-ylmethyl)piperazin-1-yl)ethanesulfonyl azide (SZ25)

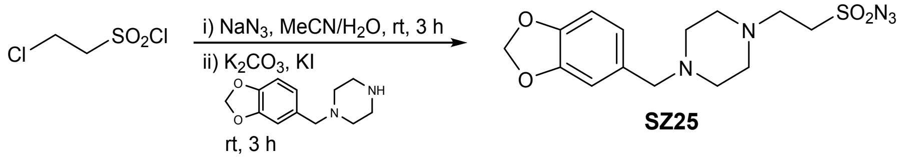

The procedure for the synthesis of **SZ22** was used for the synthesis of **SZ25**. Yield = 47%. R_f = 0.87 in DCM : MeOH = 20:1. ^{1}H NMR (400 MHz, $CDCl_3$) δ 6.82 (s, 1H), 6.72 (m, 2H), 5.91 (s, 2H), 3.48 (t, J = 6.3 Hz, 2H), 3.39 (s, 2H), 2.87 (t, J = 6.3 Hz, 2H), 2.56 – 2.42 (m, J = 22.7 Hz, 8H). ^{13}C NMR (101 MHz, $CDCl_3$) δ 147.7, 146.7, 131.9, 122.2, 109.4, 107.9, 100.9, 62.6, 53.5, 53.0, 52.8, 52.2. HRMS (ESI) calcd for $C_{14}H_{19}N_5O_4S$ $[M+H]^+$: 354.1231, found: 354.1226.

Synthesis of 2-(dibenzylamino)ethanesulfonyl azide (SZ26)

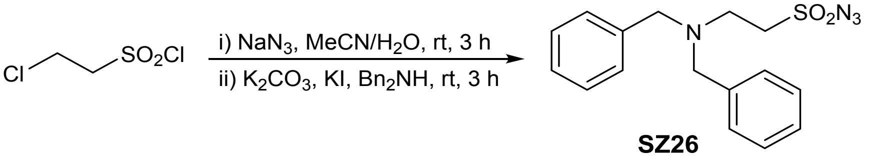

The procedure for the synthesis of **SZ22** was used for the synthesis of **SZ26**. Yield = 28%. R_f = 0.54 in hexanes : EtOAc = 4:1. ^{1}H NMR (400 MHz, $CDCl_3$) δ 7.38 – 7.23 (m, 10H), 3.65 (s, 4H), 3.34 (dd, J = 8.3, 6.1 Hz, 2H), 3.05 (dd, J = 8.3, 6.2 Hz, 2H). ^{13}C NMR (101 MHz, $CDCl_3$) δ 138.1, 129.0, 128.7, 127.7, 58.8, 53.8, 47.6. HRMS (ESI) calcd for $C_{16}H_{18}N_4O_2S$ $[M+H]^+$: 331.1223, found: 331.1218.

Synthesis of 2-(6,7-dimethoxy-1-phenyl-3,4-dihydroisoquinolin-2(1H)-yl)ethanesulfonyl azide (SZ27)[12]

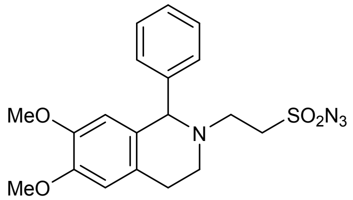

2-((benzo[d][1,3]dioxol-5-ylmethyl)(2-(phenylthio)ethyl)amino)ethane-1-sulfonyl azide (SZ28)[11]

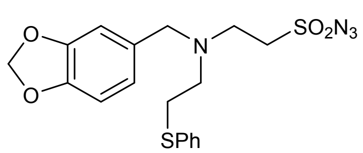

Synthesis of 4-((4-bromobenzyl)amino)benzenesulfonyl azide (SZ29)

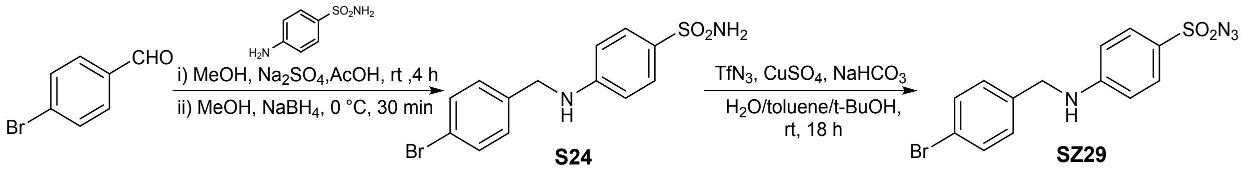

A round bottom flask was charged with 4-bromobenzaldehyde (300 mg, 1.62 mmol), 4-aminobenzenesulfonamide (280 mg, 1.62 mmol), Na_2SO_4 (1.0 g, 7.0 mmol), MeOH (5.0 mL), AcOH (0.3 mL), a magnetic stir bar and the mixture was stirred for 4 h. $NaBH_4$ (184 mg, 4.86 mmol) was added portion wise at 0 °C and stirred for an additional 30 min. The reaction was quenched with water, extracted with CH_2Cl_2, dried with Na_2SO_4, solvent removed under reduced pressure and purified using flash column chromatography resulting in pure **S24**. An Erlenmeyer flask was charged with sulfonamide **S24** (250 mg, 0.73 mmol), $NaHCO_3$ (246 mg, 2.93 mmol), water (0.9 mL), 1 M $CuSO_4$ (0.03 mL of a 1 M solution, 4 mol%), a magnetic stir bar and stirred at rt. To the flask was added a freshly prepared solution of TfN_3 (1.5 mL of a 0.75 *M* solution, 1.50 mmol) in toluene followed by *t*-BuOH (6.0 mL) and the mixture was stirred behind a blast shield for 18 h. The reaction mass was transferred to a round bottom flask and diluted with xylenes. Volatile solvents were removed under reduced pressure, the product precipitated and the solids recovered using vacuum filtration. The crude residue was purified using flash column chromatography and **SZ29** was obtained in 19% yield over two synthetic steps. $R_f = 0.51$ in hexanes : EtOAc = 4:1. ^1H NMR (400 MHz, $CDCl_3$) δ 7.69 (d, *J* = 8.8 Hz, 2H), 7.49 (d, *J* = 8.2 Hz, 2H), 7.21 (d, *J* = 8.2 Hz, 2H), 6.64 (d, *J* = 8.8 Hz, 2H), 4.91 (t, *J* = 5.3 Hz, 1H), 4.38 (d, *J* = 5.4 Hz, 2H). ^{13}C NMR (101 MHz, $CDCl_3$) δ 152.8, 136.6, 132.2, 130.0, 129.0, 125.2, 121.8, 112.3, 47.0.

Synthesis of 4-((4-(diethylamino)benzyl)amino)benzenesulfonyl azide (SZ30)

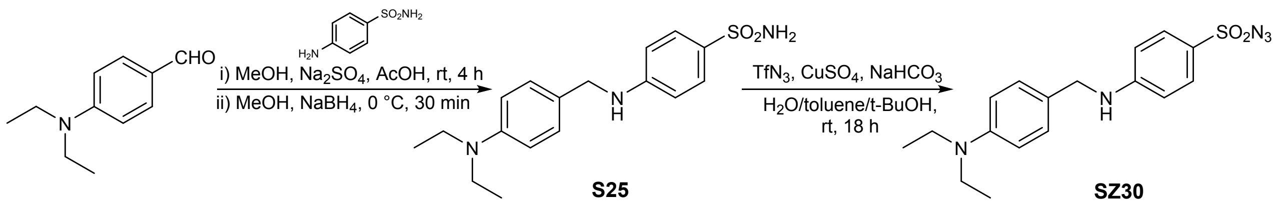

A round bottom flask was charged with 4-(diethylamino)benzaldehyde (300 mg, 1.69 mmol), 4-aminobenzenesulfonamide (292 mg, 1.69 mmol), Na_2SO_4 (1.0 g, 7.0 mmol), MeOH (15.0 mL), AcOH (0.3 mL), a magnetic stir bar and was stirred for 4 h. $NaBH_4$ (320 mg, 8.46 mmol) was added portion wise at 0 °C and stirred for an additional 30 min. The reaction was quenched with water, extracted with CH_2Cl_2, dried with Na_2SO_4, solvent removed under reduced pressure and purified using flash column chromatography resulting in pure **S25**. An Erlenmeyer flask was charged with sulfonamide **S25** (200 mg, 0.60 mmol), $NaHCO_3$ (202 mg, 2.40 mmol), water (0.7 mL), 1 M $CuSO_4$ (0.02 mL of a 1 M solution, 4 mol%), a magnetic stir bar and stirred at rt. To the flask was added a freshly prepared solution of TfN_3 (1.2 mL of a 0.75 *M* solution, 1.50 mmol) in toluene followed by *t*-BuOH (4.8 mL) and the mixture was stirred behind a blast shield for 18 h. The reaction mass was transferred to a round bottom flask and diluted with xylenes. Volatile solvents were removed under reduced pressure, the product precipitated and the solids recovered using vacuum filtration. The crude residue was purified using flash column chromatography and **SZ30** was obtained in 19% yield over two synthetic steps. $R_f = 0.48$ in hexanes : EtOAc = 4:1. ^1H NMR (400 MHz, $CDCl_3$) δ 7.69 (d, *J* = 8.6 Hz, 2H), 7.17 (d, *J* = 8.4 Hz, 2H), 6.70 – 6.59 (m, 4H), 4.73 (s, 1H), 4.24 (d, *J* = 4.8 Hz, 2H), 3.35 (q, *J* = 6.9 Hz, 4H), 1.16 (t, *J* = 7.0 Hz, 6H). ^{13}C NMR (101 MHz, $CDCl_3$) δ 153.3, 147.7, 130.1, 129.1, 124.1, 123.6, 112.1, 112.0, 47.4, 44.6, 12.7. HRMS (ESI) calcd for $C_{17}H_{21}N_5O_2S$ $[M+H]^+$: 360.1489, found: 360.1490.

Synthesis of 4-(((benzo[d][1,3]dioxol-5-ylmethyl)(2-(phenylthio)ethyl)amino)methyl)benzenesulfonyl azide (SZ31)

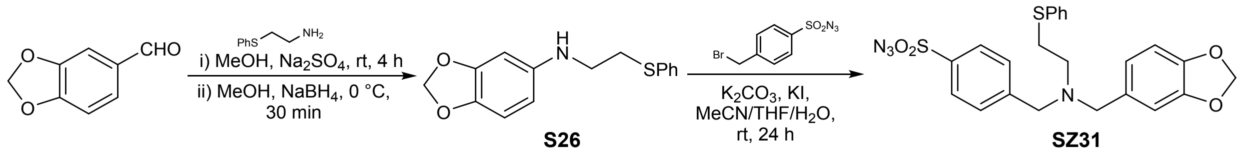

A round bottom flask was charged with benzo[d][1,3]dioxole-5-carbaldehyde (265 mg, 1.76 mmol), 2-(phenylthio)ethanamine (270 mg, 1.76 mmol), Na_2SO_4 (500 mg, 3.52 mmol), MeOH (4.4 mL), a magnetic stir bar and was stirred for 4 h. $NaBH_4$ (200mg, 5.28 mmol) was added portion wise at 0 °C and stirred for an additional 30 min. The reaction was quenched with water, extracted with CH_2Cl_2, dried with Na_2SO_4, solvent removed under reduced pressure and purified using flash column chromatography resulting in pure **S26**. The secondary amine (**S26**, 386 mg, 1.34 mmol) was added to a round bottom flask along with 4-(bromomethyl)benzenesulfonyl azide (323 mg, 1.21 mmol), K_2CO_3 (448 mg, 3.24 mmol), KI (108 mg, 0.65 mmol), MeOH/THF/H_20 (22:3:1, 16 mL), a magnetic stir bar and stirred at rt for 24 h. The reaction was judged to be complete via TLC, quenched with water, extracted with EtOAc, dried over Na_2SO_4 and concentrated under reduced pressure. The crude residue was purified using flash column chromatography. The two synthetic steps gave sulfonyl azide **SZ31** in 60% yield. R_f = 0.64 in hexanes : EtOAc = 2:1. ^{1}H NMR (400 MHz, $CDCl_3$) δ 7.87 (d, J = 8.3 Hz, 2H), 7.60 (d, J = 8.3 Hz, 2H), 7.22 (d, J = 4.3 Hz, 4H), 7.19 – 7.12 (m, 1H), 6.92 (s, 1H), 6.80 – 6.73 (m, 2H), 5.94 (s, 2H), 3.68 (s, 2H), 3.55 (s, 2H), 3.09 – 3.03 (m, 2H), 2.78 – 2.72 (m, 2H). ^{13}C NMR (101 MHz, $CDCl_3$) δ 147.81, 147.76, 146.77, 136.77, 136.16, 132.36, 129.58, 128.84, 128.80, 127.40, 125.89, 121.89, 109.05, 107.91, 100.94, 58.25, 57.60, 52.62, 31.29. HRMS (ESI) calcd for $C_{23}H_{22}N_4O_4S_2$ $[M+H]^+$: 483.1155, found: 483.1159.

Synthesis of 4-(3,5-dimethylisoxazol-4-yl)benzenesulfonyl azide (SZ32)

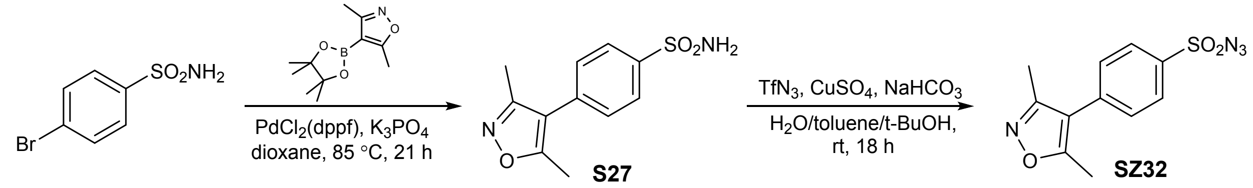

A flame dried sealed tube was charged with 4-bromobenzenesulfonamide (250 mg, 1.06 mmol), 3,5-dimethyl-4-(4,4,5,5-tetramethyl-1,3,2-dioxaborolan-2-yl)isoxazole (236 mg, 1.06 mmol), $PdCl_2$(dppf) (86.0 mg, 10.0 mol%), K_3PO_4 (674 mg, 3.18 mmol), dioxane (6.0 mL), a magnetic stir bar and purged with argon. The reaction was heated at 85 °C for 21 h. The reaction mixture was cooled, diluted with DCM, and filtered through Celite. The solution was dried over Na_2SO_4 and the solvent evaporated under reduced pressure. The product **S27** was purified using flash column chromatography resulting in a yellowish solid (240 mg, 85 %). R_f = 0.35 in hexanes : EtOAc = 1:1. ^{1}H NMR (400 MHz, DMSO-d_6) δ 7.91 (d, J = 8.4 Hz, 2H), 7.61 (d, J = 8.4 Hz, 2H), 7.42 (s, 2H), 2.43 (s, 3H), 2.25 (s, 3H). ^{13}C NMR (101 MHz, DMSO-d_6) δ 165.93, 158.02, 142.98, 133.45, 129.27, 126.14, 114.98, 11.39, 10.44.

An Erlenmeyer flask was charged with sulfonamide **S27** (200 mg, 0.79 mmol), $NaHCO_3$ (266 mg, 3.17 mmol), water (1.0 mL), 1 M $CuSO_4$ (0.03 mL of a 1 M solution, 4 mol%), a magnetic stir bar and stirred at rt. To the flask was added a freshly prepared solution of TfN_3 (1.6 mL of a 0.75 M solution, 1.50 mmol) in toluene followed by *t*-BuOH (6.3 mL) and the mixture was stirred behind a blast shield for 18 h. The reaction was transferred to a round bottom flask and diluted with xylenes. Volatile solvents were removed under reduced pressure, the product precipitated and the solids recovered using vacuum filtration. The crude residue was purified using flash column

chromatography and **SZ32** was obtained as a yellow solid (177 mg, 80 %). R_f = 0.71 in hexanes : EtOAc = 1:1. ^{1}H NMR (400 MHz, $CDCl_3$) δ 8.06 – 8.01 (m, 2H), 7.54 – 7.50 (m, 2H), 2.48 (s, 3H), 2.33 (s, 3H). ^{13}C NMR (101 MHz, $CDCl_3$) δ 166.5, 158.1, 137.7, 137.4, 130.0, 128.0, 115.1, 11.8, 10.8. HRMS (ESI) calcd for $C_{11}H_{10}N_4O_3S$ $[M+H]^+$: 279.0547, found: 279.0539.

Synthesis of 4-(2,3-dihydrobenzo[b][1,4]dioxin-6-yl)benzenesulfonyl azide (SZ33)

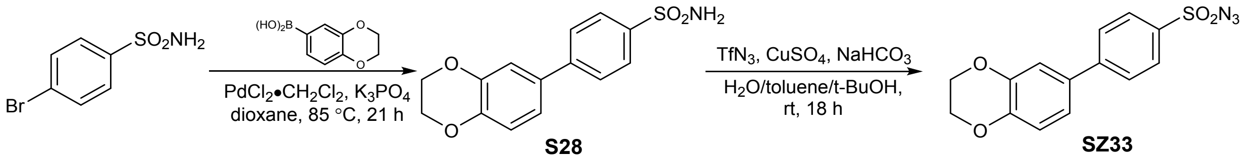

A flame dried sealed tube was charged with 4-bromobenzenesulfonamide (200 mg, 0.85 mmol), (2,3-dihydrobenzo[b][1,4]dioxin-6-yl)boronic acid (152 mg, 0.85 mmol), $PdCl_2$(dppf) (70.0 mg, 10.0 mol%), K_3PO_4 (540 mg, 2.54 mmol), dioxane (4.0 mL), a magnetic stir bar and purged with argon. The reaction was heated at 85 °C for 21 h. The reaction mixture was cooled, diluted with CH_2Cl_2 and filtered through Celite. The solution was dried over Na_2SO_4 and the solvent evaporated under reduced pressure. The product (**S28**) was purified using flash column chromatography resulting in a yellow solid (150 mg, 61 %). R_f = 0.43 in hexanes : EtOAc = 1:1. ^{1}H NMR (400 MHz, DMSO-d_6) δ 7.90 – 7.74 (m, 4H), 7.36 (s, 2H), 7.27 – 7.18 (m, 2H), 6.97 (d, J = 8.4 Hz, 1H), 4.29 (s, 4H). ^{13}C NMR (101 MHz, DMSO-d_6) δ 143.88, 143.78, 142.75, 142.33, 131.84, 126.55, 126.21, 119.89, 117.65, 115.49, 64.22, 64.10.
An Erlenmeyer flask was charged with sulfonamide **S28** (150 mg, 0.51 mmol), $NaHCO_3$ (173 mg, 2.06 mmol), water (0.6 mL), 1 *M* $CuSO_4$ (0.02 mL of a 1 *M* solution, 4 mol %), a magnetic stir bar and stirred at rt. To the flask was added a freshly prepared solution of TfN_3 (1.0 mL of a 0.75 *M* solution, 1.50 mmol) in toluene followed by *t*-BuOH (4.1 mL) and the mixture was stirred behind a blast shield for 18 h. The reaction mass was transferred to a round bottom flask and diluted with xylenes. Volatile solvents were removed under reduced pressure, the product precipitated and the solids recovered using vacuum filtration. The crude residue was purified using flash column chromatography and **SZ33** was obtained as a yellow solid (60 mg, 37%). R_f = 0.82 in hexanes : EtOAc = 1:1. ^{1}H NMR (400 MHz, $CDCl_3$) δ 8.01 – 7.96 (m, 2H), 7.77 – 7.72 (m, 2H), 7.18 – 7.15 (m, 1H), 7.13 (dd, J = 1.6, 8.2 Hz, 1H), 7.01 – 6.96 (m, 1H), 4.33 (s, 4H). ^{13}C NMR (101 MHz, $CDCl_3$) δ 147.2, 144.7, 144.0, 136.3, 131.9, 128.0, 127.6, 120.5, 118.0, 116.2, 64.5, 64.4. HRMS (ESI) calcd for $C_{14}H_{11}N_3O_4S$ $[M+H]^+$: 318.0543, found: 318.0541.

Synthesis of 3'-(dimethylcarbamoyl)-[1,1'-biphenyl]-4-sulfonyl azide (SZ34)

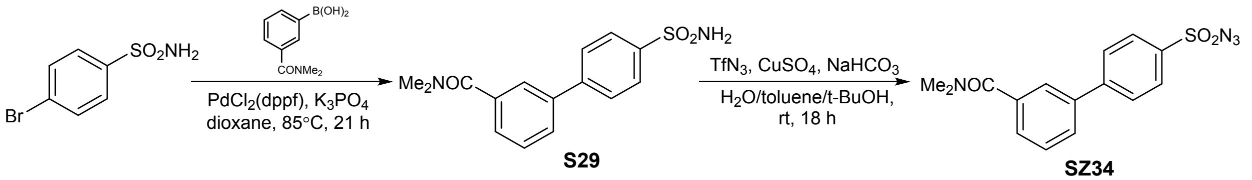

A flame dried sealed tube was charged with 4-bromobenzenesulfonamide (250 mg, 1.06 mmol), (3-(dimethylcarbamoyl)phenyl)boronic acid (205 mg, 1.06 mmol), $PdCl_2$(dppf) (86.5 mg, 10.0 mol%), K_3PO_4 (674 mg, 3.18 mmol), dioxane (5.0 mL), a magnetic stir bar and purged with argon. The reaction was heated at 85 °C for 21 h. The reaction mixture was cooled, diluted with DCM and filtered through Celite. The solution was dried over Na_2SO_4 and the solvent evaporated under

reduced pressure. The product (**S29**) was purified using flash column chromatography resulting in a yellowish solid (232 mg, 72 %). R_f = 0.43 in EtOAc. ^{1}H NMR (400 MHz, DMSO-d_6) δ 7.91 (s, 4H), 7.80 (d, *J* = 7.7 Hz, 1H), 7.73 (s, 1H), 7.57 (t, *J* = 7.7 Hz, 1H), 7.44 (d, *J* = 14.4 Hz, 3H), 2.98 (d, *J* = 26.3 Hz, 6H). ^{13}C NMR (101 MHz, DMSO-d_6) δ 169.69, 143.20, 142.53, 138.66, 137.39, 129.09, 127.81, 127.27, 126.71, 126.28, 125.33, 34.70.

An Erlenmeyer flask was charged with sulfonamide **S29** (230 mg, 0.76 mmol), $NaHCO_3$ (254 mg, 3.04 mmol), water (0.9 mL), 1 M $CuSO_4$ (0.03 mL of a 1 *M* solution, 4 mol %), a magnetic stir bar and stirred at rt. To the flask was added a freshly prepared solution of TfN_3 (1.5 mL of a 0.75 M solution, 1.50 mmol) in toluene followed by *t*-BuOH (6.0 mL) and the mixture was stirred behind a blast shield for 18 h. The reaction was transferred to a round bottom flask and diluted with xylenes. Volatile solvents were removed under reduced pressure, the product precipitated and the solids recovered using vacuum filtration. The crude residue was purified using flash column chromatography and **SZ34** was obtained as a yellow solid (224 mg, 90%). R_f = 0.68 in EtOAc. ^{1}H NMR (400 MHz, $CDCl_3$) δ 7.92 (d, *J* = 8.6 Hz, 2H), 7.74 (d, *J* = 8.6 Hz, 2H), 7.62 (s, 1H), 7.60 – 7.55 (m, 1H), 7.47 – 7.37 (m, 2H), 3.05 (br. s., 3H), 2.94 (br. s., 3H). ^{13}C NMR (101 MHz, $CDCl_3$) δ 170.4, 146.5, 138.5, 137.1, 136.8, 128.9, 128.0, 127.9, 127.7, 127.1, 125.7, 39.2, 34.9. HRMS (ESI) calcd for $C_{15}H_{14}N_4O_3S$ $[M+H]^+$: 331.0860, found: 331.0856

.

Synthesis of 4'-phenoxy-[1,1'-biphenyl]-4-sulfonyl azide (SZ35)

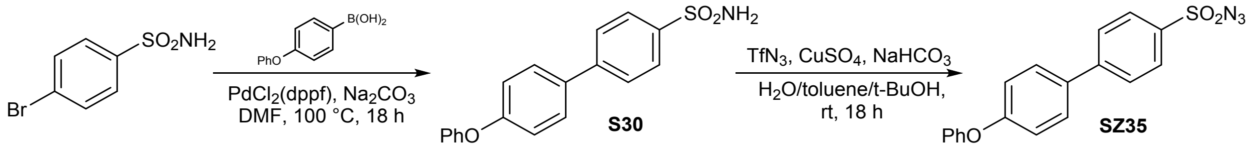

A flame dried sealed tube was charged with 4-bromobenzenesulfonamide (250 mg, 1.06 mmol), (4-phenoxyphenyl)boronic acid (250 mg, 1.17 mmol), $PdCl_2$(dppf) (70.0 mg, 8.0 mol%), 2 N Na_2CO_3 (0.75 mL, 3.18 mmol), DMF (8.5 mL), a magnetic stir bar and purged with argon. The reaction was heated at 100 °C for 18 h. The reaction mixture was cooled, diluted with DCM and filtered through Celite. The solution was dried over Na_2SO_4 and the solvent evaporated under reduced pressure. The product (**S30**) was purified using flash column chromatography resulting in a yellow solid (310 mg, 90 %). R_f = 0.56 in hexanes : EtOAc = 1:1. ^{1}H NMR (400 MHz, DMSO-d_6) δ 7.98 – 7.81 (m, 4H), 7.81 – 7.68 (m, 2H), 7.51 – 7.36 (m, 4H), 7.19 (t, *J* = 7.4 Hz, 1H), 7.10 (t, *J* = 8.9 Hz, 4H). ^{13}C NMR (101 MHz, DMSO-d_6) δ 157.19, 156.14, 142.59, 133.61, 130.10, 128.66, 126.75, 126.25, 123.83, 119.02, 118.74, 95.79.

An Erlenmeyer flask was charged with sulfonamide **S30** (310 mg, 0.95 mmol), $NaHCO_3$ (320 mg, 3.81 mmol), water (1.1 mL), 1 M $CuSO_4$ (0.04 mL of a 1 M solution, 4 mol %), a magnetic stir bar and stirred at rt. To the flask was added a freshly prepared solution of TfN_3 (1.9 mL of a 0.75 M solution, 1.50 mmol) in toluene followed by *t*-BuOH (7.6 mL) and the mixture was stirred behind a blast shield for 18 h. The reaction was transferred to a round bottom flask and diluted with xylenes. Volatile solvents were removed under reduced pressure, the product precipitated and the solids recovered using vacuum filtration. The crude residue was purified using flash column chromatography and **SZ35** was obtained as a yellow solid (234 mg, 70%). R_f = 0.86 in hexanes : EtOAc = 1:1. ^{1}H NMR (400 MHz, $CDCl_3$) δ 8.02 (d, *J* = 8.2 Hz, 2H), 7.82 – 7.76 (m, 2H), 7.63 – 7.58 (m, 2H), 7.44 – 7.37 (m, 2H), 7.22 – 7.16 (m, 1H), 7.15 – 7.08 (m, 4H). ^{13}C NMR (101 MHz, $CDCl_3$) δ 158.6, 156.3, 147.1, 136.5, 133.2, 129.9, 128.8, 128.1, 127.8, 124.0, 119.5, 118.9. HRMS (ESI) calcd for $C_{18}H_{13}N_3O_3S$ $[M+H]^+$: 352.0751, found: 352.0757

Synthesis of 4-(2,7a-dihydro-1H-indol-2-yl)benzenesulfonyl azide (SZ36)

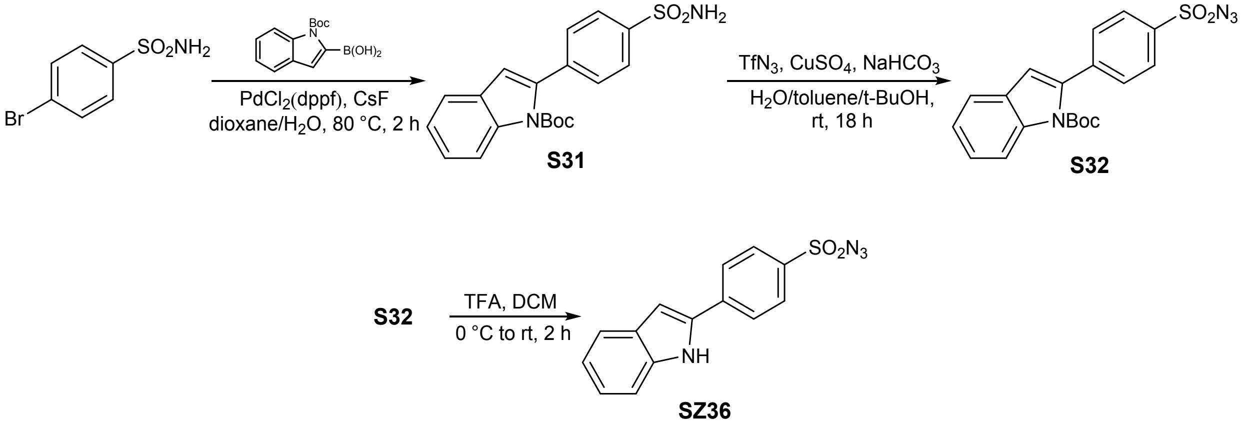

A flame dried sealed tube was charged with 4-bromobenzenesulfonamide (300 mg, 1.27 mmol), (1-(tert-butoxycarbonyl)-1*H*-indol-2-yl)boronic acid (498 mg, 1.90 mmol), $PdCl_2$(dppf) (93.0 mg, 10.0 mol%), CsF (289 mg, 1.90 mmol), dioxane/H_2O mixture (9:1, 18.0 mL), a magnetic stir bar and purged with argon. The reaction was heated at 80 °C for 2 h. The reaction mixture was cooled, diluted with DCM, and filtered through Celite. The solution was dried over Na_2SO_4 and the solvent evaporated under reduced pressure. The product **S31** was purified using flash column chromatography resulting in a yellow solid (388 mg, 82 %). R_f = 0.64 in hexanes : EtOAc = 1:1. ^{1}H NMR (400 MHz, $CDCl_3$) δ 8.17 (dd, *J* = 8.4, 0.9 Hz, 1H), 8.01 – 7.82 (m, 2H), 7.69 – 7.46 (m, 3H), 7.44 – 7.30 (m, 1H), 7.32 – 7.09 (m, 2H), 6.61 (d, *J* = 0.7 Hz, 1H), 1.36 (s, 9H). ^{13}C NMR (101 MHz, $CDCl_3$) δ 150.05, 140.95, 139.56, 138.56, 137.74, 129.34, 129.11, 126.11, 125.23, 123.42, 121.00, 115.56, 111.72, 84.41, 27.85.

An Erlenmeyer flask was charged with sulfonamide **S31** (300 mg, 0.81 mmol), $NaHCO_3$ (271 mg, 3.22 mmol), water (1.0 mL), 1 M $CuSO_4$ (0.03 mL of a 1 *M* solution, 4 mol %), a magnetic stir bar and stirred at rt. To the flask was added a freshly prepared solution of TfN_3 (1.6 mL of a 0.75 *M* solution, 1.50 mmol) in toluene followed by *t*-BuOH (6.4 mL) and the mixture was stirred behind a blast shield for 18 h. The reaction was transferred to a round bottom flask and diluted with xylenes. Volatile solvents were removed under reduced pressure, the product precipitated and the solids recovered using vacuum filtration. The crude residue was purified using flash column chromatography and **S32** was obtained as a yellow solid (315 mg, 98 %). R_f = 0.87 in hexanes : EtOAc = 1:1.. ^{1}H NMR (400 MHz, $CDCl_3$) δ 8.40 – 8.12 (m, 1H), 8.00 (d, *J* = 8.5 Hz, 2H), 7.76 – 7.63 (m, 2H), 7.65 – 7.54 (m, 1H), 7.51 – 7.35 (m, 1H), 7.34 – 7.27 (m, 1H), 6.70 (d, *J* = 0.8 Hz, 1H), 1.40 (s, 9H). ^{13}C NMR (101 MHz, $CDCl_3$) δ 149.87, 141.71, 137.93, 137.87, 137.16, 129.77, 129.00, 127.14, 125.56, 123.55, 121.14, 115.64, 112.36, 84.50, 27.80.

S32 (200 mg, 0.5 mmol) was then combined in a round bottom flask with DCM (8.0 mL), TFA (3.0 mL) at 0 °C and allowed to stir for 2 h. The solvent was evaporated, the crude material purified using prep HPLC and **SZ36** was obtained as a yellow solid (122 mg, 82 %). R_f = 0.84 in hexanes : EtOAc = 1:1. ^{1}H NMR (400 MHz, DMSO-d_6) δ 11.87 (s, 1H), 8.25 – 8.04 (m, 4H), 7.60 (d, *J* = 7.8 Hz, 1H), 7.46 (dd, *J* = 0.8, 8.2 Hz, 1H), 7.25 – 7.11 (m, 2H), 7.09 – 7.00 (m, 1H). ^{13}C NMR (101 MHz, DMSO-d_6) δ 203.3, 138.7, 137.9, 135.0, 128.3, 128.2, 125.8, 123.1, 120.8, 119.9, 111.7, 102.3. HRMS (ESI) calcd for $C_{14}H_{10}N_4O_2S$ $[M+H]^+$: 299.0597, found: 299.0592

Synthesis of 4-(2,7a-dihydrobenzo[b]thiophen-2-yl)benzenesulfonyl azide (SZ37)

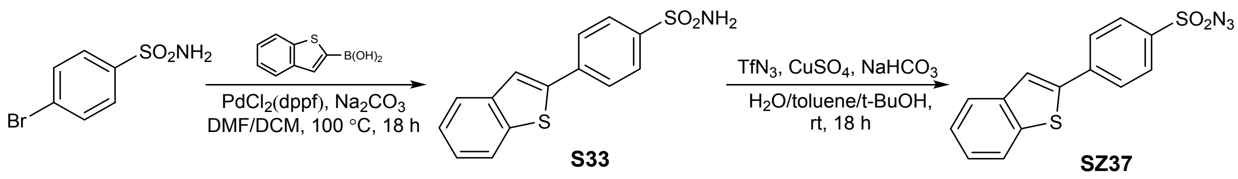

A flame dried sealed tube was charged with 4-bromobenzenesulfonamide (361 mg, 1.53 mmol), benzo[b]thiophen-2-ylboronic acid (300 mg, 1.68 mmol), $PdCl_2$(dppf) (98.0 mg, 8.0 mol%), 2 N Na_2CO_3 (1.12 mL, 2.29 mmol), DMF (9.0 mL), a magnetic stir bar and purged with argon. The reaction was heated at 100 °C for 18 h. The reaction mixture was cooled, diluted with DCM and filtered through Celite. The solution was dried over Na_2SO_4 and the solvent evaporated under reduced pressure. The product **S33** was purified using flash column chromatography resulting in a yellow solid (221 mg, 51 %). R_f = 0.69 in hexanes : EtOAc = 1:1. ^{1}H NMR (400 MHz, DMSO-d_6) δ 8.06 – 7.95 (m, 4H), 7.95 – 7.84 (m, 3H), 7.51 – 7.37 (m, 4H). ^{13}C NMR (101 MHz, DMSO-d_6) δ 143.47, 141.34, 140.12, 138.89, 136.48, 126.49, 126.28, 125.14, 124.91, 124.04, 122.46, 121.84. An Erlenmeyer flask was charged with sulfonamide **S33** (190 mg, 0.66 mmol), $NaHCO_3$ (220 mg, 2.62 mmol), water (0.8 mL), 1 M $CuSO_4$ (0.03 mL of a 1 *M* solution, 4 mol%), a magnetic stir bar and stirred at rt. To the flask was added a freshly prepared solution of TfN_3 (1.3 mL of a 0.75 M solution, 1.50 mmol) in toluene followed by *t*-BuOH (5.2 mL) and the mixture was stirred behind a blast shield for 18 h. The reaction was transferred to a round bottom flask and diluted with xylenes. Volatile solvents were removed under reduced pressure, the product precipitated and the solids recovered using vacuum filtration. The crude residue was purified using flash column chromatography and **SZ37** was obtained as a yellow solid (44 mg, 21 %). R_f = 0.78 in hexanes : EtOAc = 1:1. ^{1}H NMR (399 MHz, DMSO-d_6) δ 8.17 (s, 1H), 8.15 – 8.07 (m, 4H), 8.07 – 8.02 (m, 1H), 7.97 – 7.89 (m, 1H), 7.49 – 7.39 (m, 2H). ^{13}C NMR (100 MHz, DMSO) δ 140.38, 140.14, 139.97, 139.44, 136.44, 128.41, 127.31, 125.80, 125.22, 124.57, 123.63, 122.72. HRMS (ESI) calcd for $C_{14}H_9N_3O_2S_2[M+H]^+$: 316.0209, found: 316.0199

Synthesis of 4-(phenylsulfonyl)benzenesulfonyl azide (SZ38)

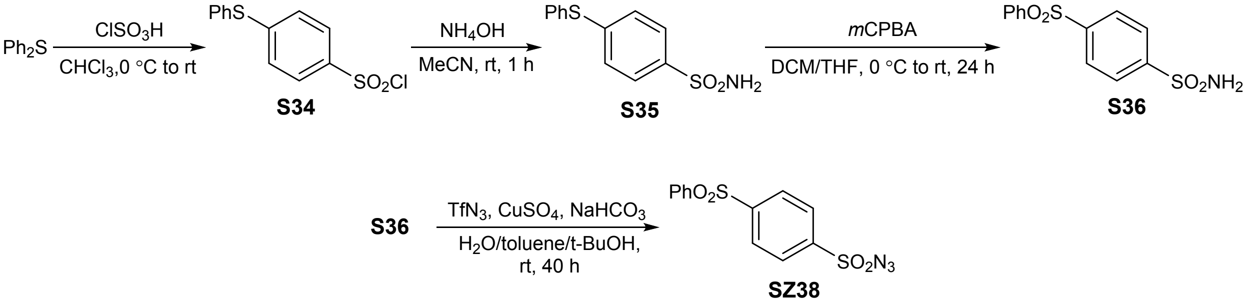

To a round bottom flask was added diphenylsulfane (14.2 g, 76.0 mmol), $CHCl_3$ (38 mL), a magnetic stir bar and cooled to 0 °C. Slowly, chlorosulfonic acid (8.86 g, 76.0 mmol) and the reaction was allowed to warm to rt while stirring for 2 h. The reaction was poured onto ice and the solid was collected using vacuum filtration, washed with water and dried resulting in crude **S34** which was used without further purification. Sulfonyl chloride **S34** (1g, 3.5 mmol) was dissolved in MeCN (18.0 mL) and NH_4OH (25 mL) was added and allowed to stir at rt for 1 h. The sulfonamide (**S35**) was purified using flash column chromatography to afford a yellow solid (723 mg, 78 %). R_f = 0.66 in hexanes : EtOAc = 1:1. ^{1}H NMR (400 MHz, DMSO-d_6) δ 7.86 – 7.68 (m,

2H), 7.56 – 7.40 (m, 5H), 7.41 – 7.29 (m, 4H). ^{13}C NMR (101 MHz, DMSO-d$_6$) δ 141.94, 141.35, 132.96, 131.86, 129.99, 128.84, 128.35, 126.61.

S35 (770 mg, 2.9 mmol) was then dissolved in a DCM/THF mixture at 0°C and *m*CPBA (1.0 g, 5.80 mmol) was added and allowed to stir for 24 h at rt. Crude **S36** was purified using flash column chromatography to afford a yellow solid (681 mg, 79 %). R_f = 0.36 in hexanes : EtOAc = 1:1.. ^{1}H NMR (400 MHz, DMSO-d$_6$) δ 8.22 – 8.13 (m, 2H), 8.06 – 7.96 (m, 4H), 7.77 – 7.70 (m, 1H), 7.69 – 7.56 (m, 4H). ^{13}C NMR (101 MHz, DMSO-d$_6$) δ 148.34, 143.85, 140.21, 134.16, 129.91, 128.32, 127.54, 127.04.

An Erlenmeyer flask was charged with sulfonamide **S36** (400 mg, 1.35 mmol), $NaHCO_3$ (452 mg, 5.39 mmol), water (1.6 mL), 1 M $CuSO_4$ (0.05 mL of a 1 *M* solution, 4 mol %), a magnetic stir bar and stirred at rt. To the flask was added a freshly prepared solution of TfN_3 (2.7 mL of a 0.75 M solution, 1.50 mmol) in toluene followed by *t*-BuOH (10.8 mL) and the mixture was stirred behind a blast shield for 40 h. The reaction was transferred to a round bottom flask and diluted with xylenes. Volatile solvents were removed under reduced pressure, the product precipitated and the solids recovered using vacuum filtration. The crude residue was purified using flash column chromatography and **SZ38** was obtained as a yellow solid (394 mg, 91 %). R_f = 0.90 in hexanes : EtOAc = 1:1. ^{1}H NMR (400 MHz, $CDCl_3$) δ 8.20 – 8.16 (m, 2H), 8.12 – 8.06 (m, 2H), 8.01 – 7.96 (m, 2H), 7.69 – 7.62 (m, 1H), 7.61 – 7.54 (m, 2H). ^{13}C NMR (101 MHz, $CDCl_3$) δ 147.6, 142.5, 139.8, 134.2, 129.7, 128.9, 128.4, 128.1. HRMS (ESI) calcd for $C_{12}H_9N_3O_4S_2$ $[M+H]^+$: 324.0107, found: 324.0110

Synthesis of acylsulfonamides. The synthesis of all acylsulfonamides was performed according to our previous report.[12]

4'-fluoro-N-((4-((4-phenylpiperazin-1-yl)methyl)phenyl)sulfonyl)-[1,1'-biphenyl]-4-carboxamide, SZ1TA14

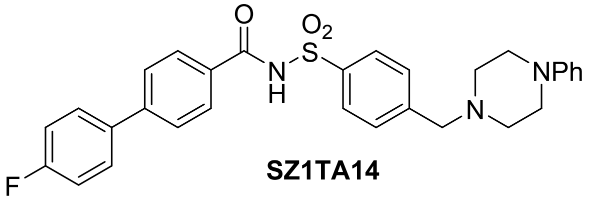

SZ1TA14

^{1}H NMR (400 MHz, acetone) δ 8.19 (d, *J* = 8.2 Hz, 2H), 8.06 (d, *J* = 8.2 Hz, 2H), 7.65 (dd, *J* = 5.5, 8.6 Hz, 2H), 7.55 (d, *J* = 8.2 Hz, 2H), 7.40 (d, *J* = 8.2 Hz, 2H), 7.21 – 7.12 (m, 4H), 6.89 – 6.84 (m, 2H), 6.75 (s, 1H), 3.52 (s, 2H), 3.12 – 3.06 (m, 4H), 2.52 – 2.46 (m, 4H). ^{13}C NMR (101 MHz, acetone) δ 173.14, 163.45 (d, *J* = 248.56), 152.41, 144.57, 143.14, 142.90, 137.82, 137.53 (d, *J* = 3.2), 130.86, 129.72 (d, *J* = 7.8), 129.71, 129.51, 127.18, 126.83, 119.84, 116.55, 116.43 (d, *J* = 21.06), 53.87, 49.61, 40.73. HRMS (ESI) calcd for $C_{30}H_{28}FN_3O_3S$ $[M+H]^+$: 530.1908, found: 530.1890

5-(4-ethoxy-3-fluorophenyl)-1-methyl-N-((4-((4-phenylpiperazin-1-yl)methyl)phenyl)sulfonyl)-1H-indole-3-carboxamide, SZ1TA23

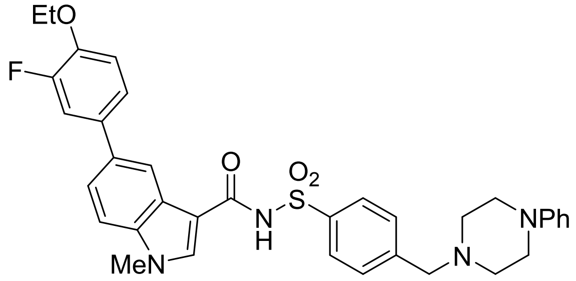

SZ1TA23

^{1}H NMR (400 MHz, DMSO-d$_6$) δ 8.29 (s, 1H), 8.18 (s, 1H), 7.96 (d, J = 8.2 Hz, 2H), 7.56 – 7.40 (m, 5H), 7.40 – 7.33 (m, 1H), 7.21 – 7.13 (m, 3H), 6.87 (d, J = 8.6 Hz, 2H), 6.78 – 6.71 (m, 1H), 4.11 (q, J = 6.9 Hz, 2H), 3.83 (s, 3H), 3.56 (s, 2H), 3.08 (br. s., 4H), 2.52 – 2.46 (m, 4H), 1.35 (t, J = 6.8 Hz, 3H). ^{13}C NMR (101 MHz, DMSO-d$_6$) δ 151.9 (d, J = 244), 150.9, 145.2 (d, J = 10), 142.5, 136.4, 135.7, 134.4 (d, J = 6), 132.3, 130.0, 129.2, 128.9, 128.8, 127.2, 125.6, 122.7 (d, J = 3), 121.3, 118.9, 118.8, 118.6, 115.3, 115.2, 114.1 (d, J = 19), 110.9, 64.4, 61.4, 52.6, 48.1, 33.2, 14.6. HRMS (ESI) calcd for $C_{35}H_{35}FN_4O_4S$ [M+H]$^+$: 627.2436, found: 627.2460

1-(2,4-bis(trifluoromethyl)benzyl)-N-((4-((4-phenylpiperazin-1-yl)methyl)phenyl)sulfonyl)-1H-indole-5-carboxamide, SZ1TA30

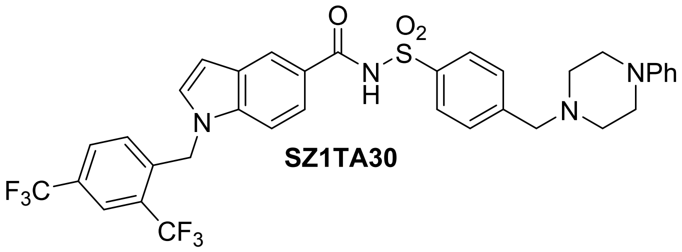

SZ1TA30

^{1}H NMR (500 MHz, DMSO-d$_6$) δ 8.29 (s, 1H), 8.10 (s, 1H), 7.91 (d, J = 8.3 Hz, 3H), 7.69 (d, J = 8.3 Hz, 1H), 7.56 (d, J = 3.4 Hz, 1H), 7.49 (d, J = 6.9 Hz, 2H), 7.24 (d, J = 8.8 Hz, 1H), 7.19 (t, J = 7.8 Hz, 2H), 6.90 (d, J = 8.3 Hz, 2H), 6.76 (t, J = 7.1 Hz, 1H), 6.71 (d, J = 3.0 Hz, 1H), 6.56 (d, J = 8.3 Hz, 1H), 3.64 (br. s., 2H), 3.14 (br. s., 4H), 2.57 (br. s., 4H), 2.50 (br. s., 2H). ^{13}C NMR (126 MHz, DMSO-d$_6$) δ 150.9, 141.5, 137.6, 130.7, 130.0, 128.9, 128.8, 128.7, 128.4, 127.7, 127.3, 127.0, 126.8, 126.7, 124.6, 124.3, 123.1, 122.5, 122.4, 122.3, 122.2, 118.9, 115.4, 109.1, 103.4, 61.2, 52.5, 48.0, 45.8. HRMS (ESI) calcd for $C_{35}H_{30}F_6N_4O_3S$ [M+H]$^+$: 701.2016, found: 701.2050

N-((3-nitro-4-((2-(phenylthio)ethyl)amino)phenyl)sulfonyl)-4-(2-(pyridin-2-yl)ethyl)benzamide, SZ4TA17

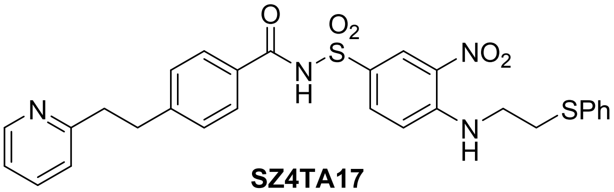

SZ4TA17

^{1}H NMR (400 MHz, DMSO-d_6) δ 8.62 (t, *J* = 5.86 Hz, 1H), 8.57 (d, *J* = 1.95 Hz, 1H), 8.47 (d, *J* = 4.30 Hz, 1H), 7.91 (dd, *J* = 2.15, 9.18 Hz, 1H), 7.79 (d, *J* = 8.20 Hz, 2H), 7.63 (dt, *J* = 1.76, 7.71 Hz, 1H), 7.38 (d, *J* = 7.42 Hz, 2H), 7.28 (t, *J* = 7.62 Hz, 2H), 7.14 - 7.23 (m, 5H), 7.06 (d, *J* = 9.37 Hz, 1H), 3.62 (q, *J* = 6.51 Hz, 2H), 3.27 (t, *J* = 6.64 Hz, 2H), 3.01 (s, 4H). ^{13}C NMR (126 MHz, DMSO-d_6) δ 168.0, 160.4, 148.9, 146.0, 145.1, 136.4, 135.0, 134.6, 133.7, 129.8, 129.6, 129.1, 128.7, 128.4, 127.9, 126.6, 126.1, 122.9, 121.3, 114.2, 41.8, 38.8, 34.8, 31.2. HRMS (ESI) calcd for $C_{28}H_{26}N_4O_5S_2$ $[M+H]^+$: 563.1362, found: 563.1364

1-(2,4-bis(trifluoromethyl)benzyl)-N-((3-nitro-4-((2-(phenylthio)ethyl)amino)phenyl)sulfonyl)-1H-indole-5-carboxamide, SZ4TA30

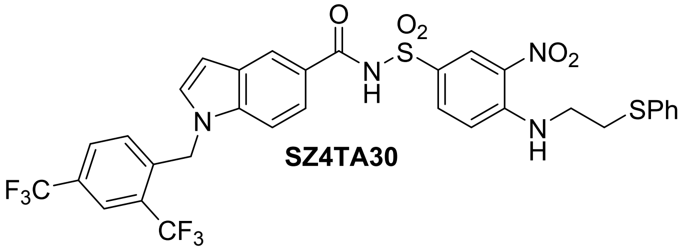

SZ4TA30

^{1}H NMR (400 MHz, DMSO-d_6) δ 12.27 (br. s., 1H), 8.76 (t, *J* = 5.5 Hz, 1H), 8.63 (br. s., 1H), 8.30 (s, 1H), 8.09 (br. s., 1H), 7.92 (dd, *J* = 8.6, 16.8 Hz, 2H), 7.70 – 7.57 (m, 2H), 7.41 – 7.30 (m, 3H), 7.26 (t, *J* = 7.4 Hz, 2H), 7.22 – 7.12 (m, 2H), 6.76 (d, *J* = 2.7 Hz, 1H), 6.57 (d, *J* = 8.2 Hz, 1H), 5.78 (br. s., 2H), 3.66 (q, *J* = 6.1 Hz, 2H), 3.34 – 3.24 (m, 2H). ^{13}C NMR (126 MHz, dmso) δ 166.33, 146.89, 141.29, 138.18, 134.97, 134.10, 131.40, 130.09, 129.79, 129.01, 128.65, 128.59 (d, *J* = 32.07), 128.36, 127.73, 126.89 (d, *J* = 32.07), 126.02, 123.64, 123.47 (d, *J* = 275.50), 123.25 (d, *J* = 275.50), 123.20, 122.63, 122.38, 122.16, 122.12, 115.04, 109.78, 103.63, 45.91 (d, *J* = 4.4), 41.95, 31.06. HRMS (ESI) calcd for $C_{32}H_{24}F_6N_4O_5S_2$ $[M+H]^+$: 723.1165, found: 723.1175

4-(4,4-dimethylpiperidin-1-yl)-N-((7-(N-(2-(phenylthio)ethyl)sulfamoyl)naphthalen-2-yl)sulfonyl)benzamide, SZ7TA2[12]

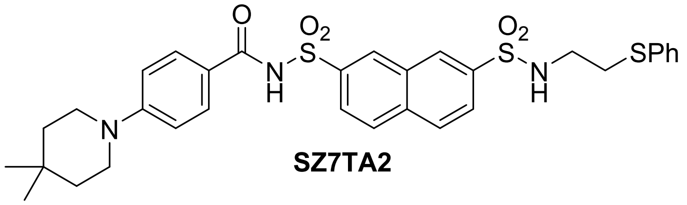

SZ7TA2

5-(4-ethoxy-3-fluorophenyl)-1-methyl-N-((7-(N-(2-(phenylthio)ethyl)sulfamoyl)naphthalen-2-yl)sulfonyl)-1H-indole-3-carboxamide, SZ7TA23

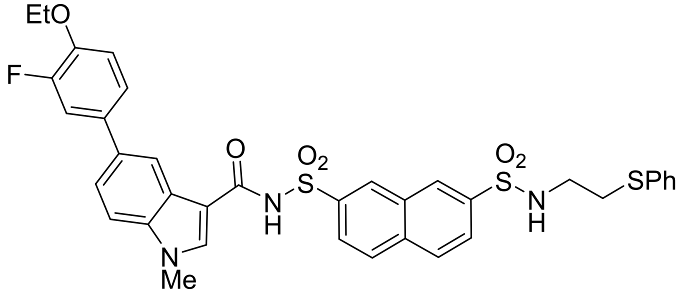

SZ7TA23

^{1}H NMR (500 MHz, DMSO-d$_6$) δ 8.78 (s, 1H), 8.60 (s, 1H), 8.31 (s, 1H), 8.21 – 8.12 (m, 4H), 8.10 – 8.06 (m, 1H), 7.92 (dd, *J* = 1.7, 8.6 Hz, 1H), 7.51 – 7.47 (m, 1H), 7.45 – 7.37 (m, 2H), 7.32 – 7.28 (m, 1H), 7.17 (d, *J* = 4.4 Hz, 4H), 7.12 – 7.05 (m, 2H), 4.07 (q, *J* = 7.0 Hz, 2H), 3.82 (s, 3H), 2.98 (d, *J* = 2.5 Hz, 4H), 1.33 (t, *J* = 7.1 Hz, 3H). ^{13}C NMR (126 MHz, DMSO-d$_6$) δ 162.3, 151.9 (d, *J* = 244 Hz), 145.1 (d, *J* = 10 Hz), 138.5, 136.4, 135.7, 135.1, 134.9, 134.5 (d, *J* = 6 Hz), 132.0, 130.7, 129.5, 129.1, 129.0, 128.6, 128.5, 128.4, 128.3, 127.3, 126.2, 126.0, 124.3, 122.6 (d, *J* = 3 Hz), 121.1, 119.0, 115.1, 115.0, 114.0 (d, *J* = 19 Hz), 110.8, 64.3, 41.9, 33.1, 32.0, 14.6. HRMS (ESI) calcd for $C_{36}H_{32}FN_3O_6S_3$ [M+H]$^+$: 718.1510, found: 718.1518

1-methyl-N-((7-(N-(2-(phenylthio)ethyl)sulfamoyl)naphthalen-2-yl)sulfonyl)-3-(thiophene-2-carbonyl)-1H-indole-5-carboxamide, SZ7TA45

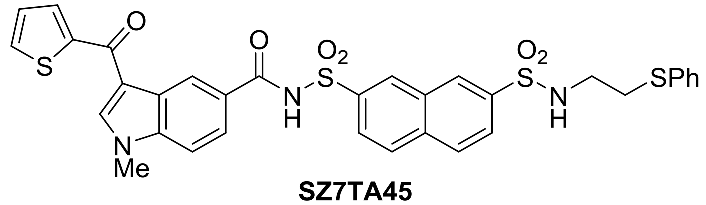

SZ7TA45

^{1}H NMR (500 MHz, DMSO-d$_6$) δ 13.00 – 12.72 (m, 1H), 8.88 (d, *J* = 1.5 Hz, 1H), 8.76 (br. s., 1H), 8.61 (br. s., 1H), 8.47 (s, 1H), 8.20 (d, *J* = 8.8 Hz, 1H), 8.18 – 8.14 (m, 2H), 8.08 (br. s., 1H), 7.97 (d, *J* = 4.4 Hz, 2H), 7.92 (d, *J* = 8.8 Hz, 1H), 7.87 (dd, *J* = 1.5, 8.3 Hz, 1H), 7.57 (d, *J* = 8.8 Hz, 1H), 7.30 – 7.25 (m, 1H), 7.22 – 7.16 (m, 4H), 7.13 – 7.08 (m, 1H), 3.92 (s, 3H), 2.99 (d, *J* = 2.5 Hz, 4H). ^{13}C NMR (126 MHz, DMSO-d$_6$) δ 180.1, 145.0, 139.0, 138.5, 135.2, 134.9, 132.7, 131.6, 130.6, 129.5, 129.0, 128.5, 128.3, 127.4, 126.2, 125.9 124.4, 123.8, 123.0, 114.1,113.8, 110.1, 41.9, 33.4, 32.0 HRMS (ESI) calcd for $C_{33}H_{27}N_3O_6S_4$ [M+H]$^+$: 690.0856, found: 690.0906

N-((4-((6,7-dimethoxy-1-phenyl-3,4-dihydroisoquinolin-2(1H)-yl)methyl)phenyl)sulfonyl)-4-(4,4-dimethylpiperidin-1-yl)benzamide, SZ8TA2

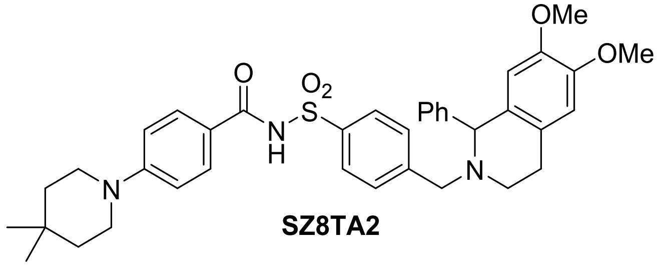

SZ8TA2

^{1}H NMR (500 MHz, DMSO-d$_6$) δ 12.10 – 11.95 (m, 1H), 7.93 (d, *J* = 8.3 Hz, 2H), 7.74 (d, *J* = 9.3 Hz, 2H), 7.52 (d, *J* = 8.3 Hz, 2H), 7.36 – 7.29 (m, 4H), 7.27 – 7.20 (m, 1H), 6.89 (d, *J* = 9.3 Hz, 2H), 6.70 (s, 1H), 6.19 (s, 1H), 4.60 (br. s., 1H), 3.71 (s, 3H), 3.66 (d, *J* = 14.2 Hz, 1H), 3.45 (s, 3H), 3.35 (br. s., 1H), 3.33 – 3.27 (m, 4H), 2.92 – 2.84 (m, 2H), 2.71 – 2.64 (m, 1H), 2.45 (br. s., 1H), 1.37 – 1.32 (m, 4H), 0.92 (s, 6H). ^{13}C NMR (126 MHz, DMSO-d$_6$) δ 164.6, 153.7, 147.3, 146.8, 145.2, 143.8, 138.6, 130.3, 129.3, 129.1, 128.4, 128.2, 127.7, 127.2, 126.5, 125.7, 118.7, 112.7, 111.9, 111.4, 67.0, 57.4, 55.40, 55.36, 46.5, 43.2, 37.4, 28.4, 27.7, 27.6. HRMS (ESI) calcd for $C_{38}H_{43}N_3O_5S$ [M+H]$^+$: 654.2996, found: 654.3015

4-(3,4-bis(2,4-difluorophenyl)-2,5-dioxo-2,5-dihydro-1H-pyrrol-1-yl)-N-((4-((6,7-dimethoxy-1-phenyl-3,4-dihydroisoquinolin-2(1H)-yl)methyl)phenyl)sulfonyl)butanamide, SZ8TA20

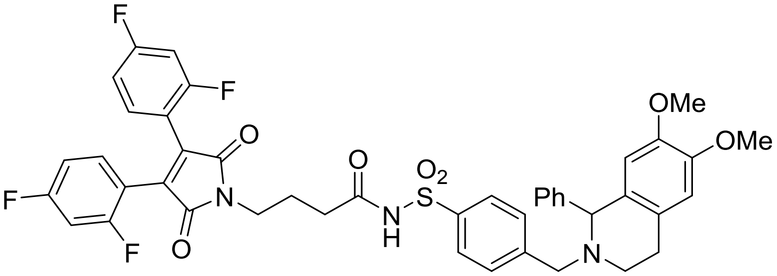

SZ8TA20

^{1}H NMR (500 MHz, DMSO-d$_6$) δ 7.87 (d, *J* = 7.8 Hz, 2H), 7.58 – 7.54 (m, 2H), 7.52 (d, *J* = 8.3 Hz, 2H), 7.37 – 7.29 (m, 6H), 7.26 – 7.18 (m, 3H), 6.70 (s, 1H), 6.20 (s, 1H), 4.60 (s, 1H), 3.71 (s, 3H), 3.67 (d, *J* = 14.7 Hz, 1H), 3.50 (t, *J* = 6.9 Hz, 2H), 3.48 – 3.43 (m, 4H), 2.87 – 2.82 (m, 2H), 2.68 (br. s., 1H), 2.45 (br. s., 1H), 2.32 (t, *J* = 7.3 Hz, 2H), 1.75 (t, *J* = 7.1 Hz, 2H). ^{13}C NMR (126 MHz, DMSO-d$_6$) δ 171.1, 168.9, 163.4 (dd, *J* = 252, 12 Hz), 159.9 (dd, *J* = 254, 12 Hz), 147.3, 146.8, 145.5, 143.9, 138.0, 133.2, 132.9 (dd, *J* = 11, 4 Hz), 129.4, 129.1, 128.5, 128.2, 127.6, 127.2, 126.5, 113.6 (dd, *J* = 14, 3 Hz), 112.0 (dd, *J* = 23, 3 Hz), 111.96, 111.4, 104.6 (t, *J* = 26 Hz), 67.0, 57.4, 55.4, 55.3, 46.5, 37.4, 32.8, 27.8, 22.8. HRMS (ESI) calcd for $C_{44}H_{37}F_4N_3O_7S$ $[M+H]^+$: 828.2361, found: 828.2350

4-(((4-(N-benzoylsulfamoyl)benzyl)(2-(phenylthio)ethyl)amino)methyl)benzenesulfonyl azide, SZ9TA1[12]

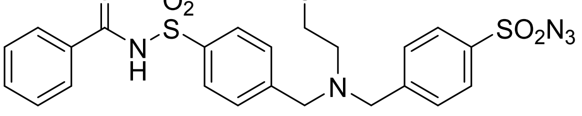

SZ9TA1

4-(((4-(N-(4-methyl-2-phenylthiazole-5-carbonyl)sulfamoyl)benzyl)(2-(phenylthio)ethyl)amino)methyl)benzenesulfonyl azide, SZ9TA3[12]

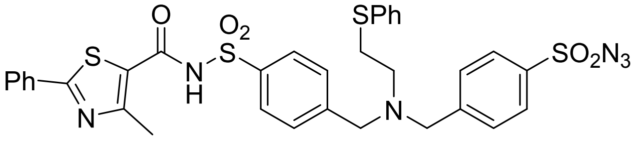

SZ9TA3

4-(((4-(N-(3,5-dimethoxybenzoyl)sulfamoyl)benzyl)(2-(phenylthio)ethyl)amino)methyl)benzenesulfonyl azide, SZ9TA5[12]

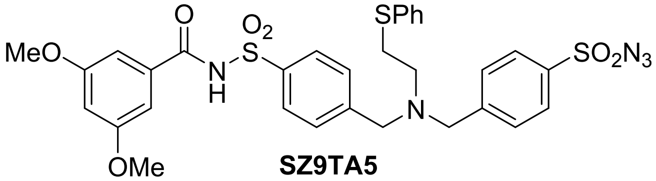

SZ9TA5

4-(((4-(N-(1-naphthoyl)sulfamoyl)benzyl)(2-(phenylthio)ethyl)amino)methyl)benzenesulfonyl azide, SZ9TA7[12]

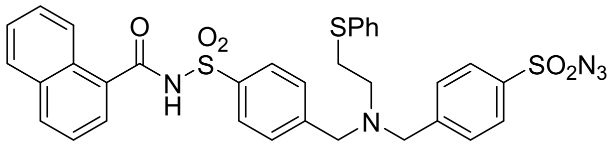

SZ9TA7

4-(((2-(phenylthio)ethyl)(4-(N-(4-(2-(pyridin-2-yl)ethyl)benzoyl)sulfamoyl)benzyl)amino)methyl)benzenesulfonyl azide, SZ9TA17

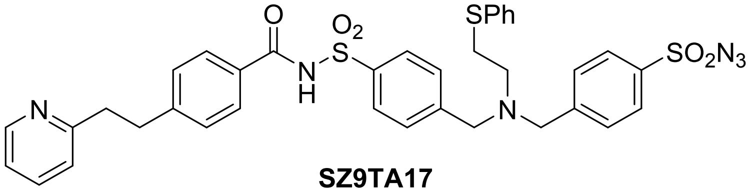

SZ9TA17

^{1}H NMR (500 MHz, DMSO-d$_6$) δ 8.48 (d, *J* = 4.9 Hz, 1H), 7.95 (d, *J* = 8.3 Hz, 2H), 7.89 (d, *J* = 8.3 Hz, 2H), 7.79 (d, *J* = 8.3 Hz, 2H), 7.73 (d, *J* = 8.3 Hz, 2H), 7.64 (dt, *J* = 2.0, 7.6 Hz, 1H), 7.53 (d, *J* = 8.3 Hz, 2H), 7.23 (d, *J* = 8.3 Hz, 2H), 7.21 – 7.14 (m, 6H), 7.02 (tt, *J* = 2.9, 5.7 Hz, 1H), 3.75 (s, 2H), 3.69 (s, 2H), 3.19 – 3.14 (m, 2H), 3.02 (s, 4H), 2.64 – 2.58 (m, 2H). ^{13}C NMR (126 MHz, DMSO-d$_6$) δ 167.2, 160.4, 148.9, 147.8, 145.6, 143.2, 136.4, 135.9, 135.8, 129.9, 128.8, 128.5, 128.3, 128.0, 127.6, 127.4, 127.3, 125.4, 123.9, 122.9, 121.3, 109.5, 57.0, 56.8, 51.9, 38.7, 34.8, 29.1. HRMS (ESI) calcd for $C_{36}H_{34}N_6O_5S_3$ [M+H]$^+$: 727.1826, found: 727.1867

4-(((4-(N-(4-(3,4-bis(2,4-difluorophenyl)-2,5-dioxo-2,5-dihydro-1H-pyrrol-1-yl)butanoyl)sulfamoyl)benzyl)(2-(phenylthio)ethyl)amino)methyl)benzenesulfonyl azide, SZ9TA20

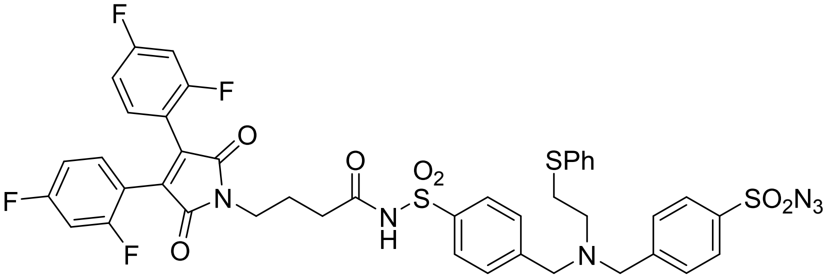

SZ9TA20

^{1}H NMR (500 MHz, DMSO-d$_6$) δ 12.07 (br. s., 1H), 7.94 (d, *J* = 8.3 Hz, 2H), 7.86 (s, 2H), 7.72 (d, *J* = 8.3 Hz, 2H), 7.61 (d, *J* = 8.3 Hz, 2H), 7.55 – 7.51 (m, 2H), 7.34 – 7.30 (m, 2H), 7.22 (dt, *J* = 2.0, 8.3 Hz, 2H), 7.19 – 7.15 (m, 4H), 7.10 – 7.07 (m, 1H), 3.75 (s, 2H), 3.72 (s, 2H), 3.49 – 3.46 (m, 2H), 3.16 (t, *J* = 7.1 Hz, 2H), 2.62 – 2.59 (m, 2H), 2.31 (t, *J* = 7.3 Hz, 2H), 1.73 (quin, *J* = 6.7 Hz, 2H). ^{13}C NMR (126 MHz, DMSO-d$_6$) δ 170.9, 168.9, 163 (dd, *J* = 252, 12 Hz), 159.9 (dd, *J* = 253, 12 Hz), 147.7, 145.3, 138.0, 135.9, 135.8, 133.1, 132.9 (dd, *J* = 10, 4 Hz), 129.9, 128.9, 128.8, 127.7, 127.5, 127.3, 125.4, 113.6 (dd, *J* = 15, 2 Hz), 112.0 (dd, *J* = 23, 3 Hz), 104.6 (t, *J* = 26 Hz), 56.9, 56.8, 51.9, 37.3, 32.7, 29.1, 22.7. HRMS (ESI) calcd for $C_{42}H_{34}F_4N_6O_7S_3$ [M+H]$^+$: 907.1660, found: 907.1675

4-(((2-(phenylthio)ethyl)(4-(N-(4-(pyridin-2-yl)benzoyl)sulfamoyl)benzyl)amino)methyl)benzenesulfonyl azide, SZ9TA25

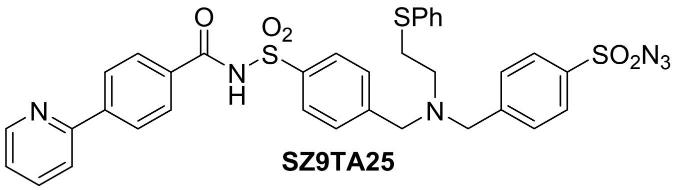

SZ9TA25

^{1}H NMR (400 MHz, DMSO-d$_6$) δ 8.68 (d, J = 4.3 Hz, 1H), 8.13 (d, J = 8.2 Hz, 2H), 8.05 – 7.97 (m, 3H), 7.97 – 7.87 (m, 5H), 7.74 (d, J = 8.2 Hz, 2H), 7.55 (d, J = 8.2 Hz, 2H), 7.38 (dd, J = 5.1, 7.4 Hz, 1H), 7.19 – 7.13 (m, 4H), 7.07 – 7.00 (m, 1H), 3.76 (s, 2H), 3.71 (s, 2H), 3.20 – 3.13 (m, 2H), 2.62 (t, J = 7.2 Hz, 2H). ^{13}C NMR (101 MHz, DMSO-d$_6$) δ 166.7, 155.0, 149.6, 147.8, 143.5, 141.6, 140.9, 137.3, 135.9, 135.8, 134.9, 129.9, 128.9, 128.8, 128.4, 127.7, 127.4, 127.3, 126.1, 125.4, 123.1, 120.7, 57.0, 56.8, 51.9, 29.1. HRMS (ESI) calcd for $C_{34}H_{30}N_6O_5S_3$ [M+H]$^+$: 699.1513, found: 699.1474

4-(4,4-dimethylpiperidin-1-yl)-N-((4-(((2-(phenylthio)ethyl)(4-sulfamoylbenzyl)amino)methyl)phenyl)sulfonyl)benzamide, SZ10TA2

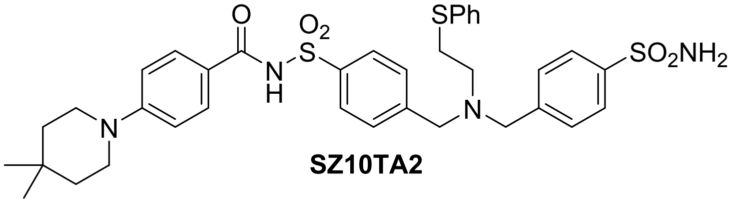

SZ10TA2

^{1}H NMR (500 MHz, DMSO-d$_6$) δ 12.00 (br. s., 1H), 7.94 (d, J = 8.8 Hz, 2H), 7.80 (d, J = 8.3 Hz, 2H), 7.75 (d, J = 8.8 Hz, 2H), 7.60 (d, J = 8.3 Hz, 2H), 7.57 (d, J = 8.3 Hz, 2H), 7.36 – 7.30 (m, 2H), 7.20 – 7.13 (m, 4H), 7.07 – 7.00 (m, 1H), 6.90 (d, J = 8.8 Hz, 2H), 3.70 (s, 2H), 3.67 (br. s., 2H), 3.34 – 3.27 (m, 4H), 3.19 – 3.12 (m, 2H), 2.64 – 2.57 (m, 2H), 1.37 – 1.31 (m, 4H), 0.92 (s, 6H). ^{13}C NMR (126 MHz, DMSO-d$_6$) δ 164.7, 153.6, 145.0, 143.2, 142.8, 138.8, 135.9, 130.3, 129.0, 128.9, 128.6, 127.7, 127.6, 125.7, 125.4, 118.9, 112.7, 56.9, 56.8, 51.8, 43.3, 37.4, 29.2, 28.5, 27.6. HRMS (ESI) calcd for $C_{36}H_{42}N_4O_5S_3$ [M+H]$^+$: 707.2390, found: 707.2403

2-(9H-carbazol-9-yl)-N-((4-(((2-(phenylthio)ethyl)(4-sulfamoylbenzyl)amino)methyl)phenyl)sulfonyl)acetamide, SZ10TA15

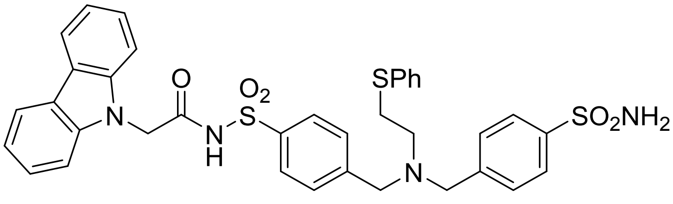

SZ10TA15

^{1}H NMR (500 MHz, DMSO-d$_6$) δ 8.10 (d, J = 7.8 Hz, 2H), 7.81 (d, J = 8.3 Hz, 2H), 7.78 (d, J = 8.3 Hz, 2H), 7.55 (d, J = 8.3 Hz, 2H), 7.44 (d, J = 7.8 Hz, 2H), 7.38 – 7.29 (m, 6H), 7.25 – 7.21 (m, 2H), 7.20 – 7.09 (m, 5H), 4.95 (s, 2H), 3.65 (s, 2H), 3.63 (s, 2H), 3.18 – 3.10 (m, 2H), 2.66 – 2.56 (m, 2H). ^{13}C NMR (126 MHz, DMSO-d$_6$) δ 170.32, 143.34, 142.82, 142.60, 142.29, 140.61, 136.08, 129.00, 128.90, 128.09, 127.62, 126.94, 125.69, 125.45, 125.44, 122.09, 120.01, 118.70,

109.31, 56.86, 56.83, 51.81, 47.35, 29.30.HRMS (ESI) cald for $C_{36}H_{34}N_4O_5S_3$ $[M+H]^+$: 699.1764, found: 699.1777

4-(3,4-bis(2,4-difluorophenyl)-2,5-dioxo-2,5-dihydro-1H-pyrrol-1-yl)-N-((4-(((2-(phenylthio)ethyl)(4-sulfamoylbenzyl)amino)methyl)phenyl)sulfonyl)butanamide, SZ10TA20

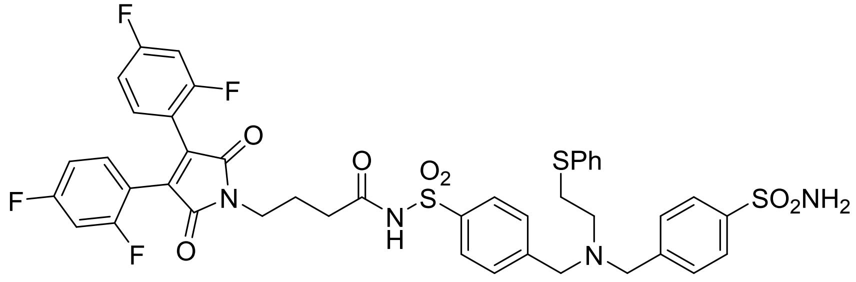

SZ10TA20

^{1}H NMR (500 MHz, DMSO-d6) δ 12.05 (br. s., 1H), 7.89 (d, *J* = 7.8 Hz, 2H), 7.80 (d, *J* = 7.8 Hz, 2H), 7.61 (d, *J* = 7.8 Hz, 2H), 7.59 – 7.50 (m, 4H), 7.37 – 7.28 (m, 4H), 7.26 – 7.13 (m, 6H), 7.12 – 7.06 (m, 1H), 3.69 (s, 4H), 3.50 (t, *J* = 6.6 Hz, 2H), 3.18 – 3.12 (m, 2H), 2.61 (t, *J* = 7.1 Hz, 2H), 2.32 (t, *J* = 7.3 Hz, 2H), 1.75 (quin, *J* = 6.9 Hz, 2H). ^{13}C NMR (126 MHz, DMSO-d6) δ 171.0, 168.9, 163.4 (dd, *J* = 251, 12 Hz), 159.8 (dd, *J* = 254, 15 Hz), 145.4, 143.2, 142.9, 138.0, 135.9, 133.1, 132.9 (dd, *J* = 10, 5 Hz), 129.0, 128.9, 128.8, 127.61, 127.56, 125.7, 125.4, 113.6 (dd, *J* = 16, 3 Hz), 112.0 (dd, *J* = 22, 4 Hz), 104.6 (t, *J* = 26 Hz), 56.9, 56.8, 51.8, 37.4, 32.7, 29.2, 22.8. HRMS (ESI) calcd for $C_{42}H_{36}F_4N_4O_7S_3$ $[M+H]^+$: 881.1755, found: 881.1710

2'-methoxy-N-((4-(((2-(phenylthio)ethyl)(4-sulfamoylbenzyl)amino)methyl)phenyl)sulfonyl)-[1,1'-biphenyl]-4-carboxamide, SZ10TA21

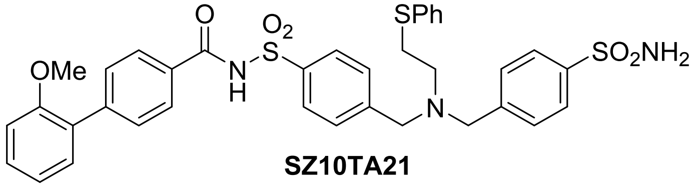

SZ10TA21

^{1}H NMR (500 MHz, DMSO-d6) δ 7.93 (d, *J* = 8.3 Hz, 2H), 7.85 (d, *J* = 8.3 Hz, 2H), 7.79 (d, *J* = 8.3 Hz, 2H), 7.57 (d, *J* = 8.3 Hz, 2H), 7.47 – 7.43 (m, 4H), 7.37 – 7.32 (m, 1H), 7.32 – 7.27 (m, 2H), 7.22 – 7.13 (m, 2H), 7.10 (d, *J* = 7.8 Hz, 2H), 7.07 – 7.00 (m, 2H), 3.75 (s, 3H), 3.70 (s, 2H), 3.71 – 3.68 (m, 2H), 3.65 (s, 2H), 3.19 – 3.12 (m, 2H), 2.65 – 2.59 (m, 2H). ^{13}C NMR (126 MHz, DMSO-d6) δ 156.12, 143.38, 142.76, 141.84, 140.46, 135.98, 130.34, 129.30, 129.17, 129.02, 128.96, 128.91, 128.58, 128.11, 128.07, 127.90, 127.55, 126.97, 125.67, 125.38, 120.81, 113.86, 111.81, 56.91, 56.85, 55.53, 51.81, 29.22. HRMS (ESI) calcd for $C_{36}H_{35}N_3O_6S_3$ $[M+H]^+$: 702.1761, found: 702.1776

N-((4-(((2-(phenylthio)ethyl)(4-sulfamoylbenzyl)amino)methyl)phenyl)sulfonyl)-4-(pyridin-2-yl)benzamide, SZ10TA25

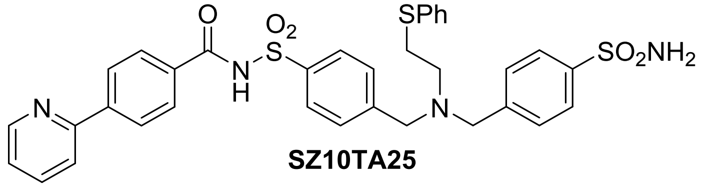

SZ10TA25

^{1}H NMR (400 MHz, DMSO-d_6) δ 8.69 – 8.65 (m, 1H), 8.09 – 8.04 (m, 2H), 8.03 – 7.96 (m, 3H), 7.91 – 7.82 (m, 3H), 7.78 (d, J = 8.6 Hz, 2H), 7.56 (d, J = 8.2 Hz, 2H), 7.43 (d, J = 8.2 Hz, 2H), 7.35 (dd, J = 4.7, 7.4 Hz, 1H), 7.29 (s, 2H), 7.23 – 7.12 (m, 4H), 7.08 – 7.02 (m, 1H), 3.69 (s, 2H), 3.64 (s, 2H), 3.19 – 3.11 (m, 2H), 2.66 – 2.59 (m, 2H). ^{13}C NMR (126 MHz, DMSO-d_6) δ 168.8, 155.5, 149.6, 144.0, 143.4, 142.7, 141.4 140.2, 138.8 137.3, 136.0, 129.0, 128.9, 128.8, 127.8, 127.5, 127.0, 125.72, 125.65, 125.4, 122.8, 120.5, 56.9, 56.8, 51.8, 29.2. HRMS (ESI) calcd for $C_{34}H_{32}N_4O_5S_3$ [M+H]$^+$: 672.1535, found: 673.1616

2-(3-oxo-3,4-dihydro-2H-benzo[b][1,4]thiazin-2-yl)-N-((4-(((2-(phenylthio)ethyl)(4-sulfamoylbenzyl)amino)methyl)phenyl)sulfonyl)acetamide, SZ10TA34

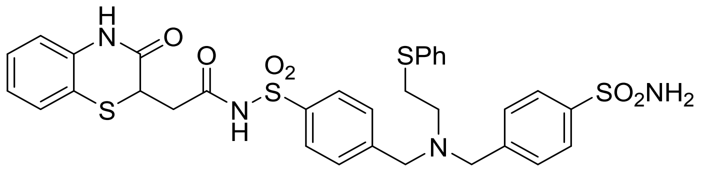

SZ10TA34

^{1}H NMR (500 MHz, DMSO-d_6) δ 10.56 (s, 1H), 7.77 (dd, J = 8.3, 15.2 Hz, 4H), 7.56 (d, J = 8.3 Hz, 2H), 7.44 (d, J = 8.3 Hz, 2H), 7.32 (s, 2H), 7.26 – 7.19 (m, 3H), 7.19 – 7.14 (m, 3H), 7.13 – 7.07 (m, 1H), 6.99 – 6.92 (m, 2H), 3.76 (dd, J = 4.4, 9.3 Hz, 1H), 3.68 (s, 2H), 3.65 (s, 2H), 3.19 – 3.13 (m, 2H), 2.67 (dd, J = 4.7, 15.9 Hz, 1H), 2.64 – 2.60 (m, 2H), 2.18 (dd, J = 9.1, 15.9 Hz, 1H). ^{13}C NMR (126 MHz, DMSO-d_6) δ 172.1, 166.5, 143.4, 142.8, 142.0, 140.4, 136.9, 136.0, 129.0, 128.9, 128.0, 127.0, 126.8, 125.7, 125.4, 125.0, 123.0, 120.0, 118.4, 116.9, 56.9, 56.9, 51.8, 38.3, 37.6, 29.3. HRMS (ESI) calcd for $C_{32}H_{32}N_4O_6S_4$ [M+H]$^+$: 697.1278, found: 697.1298

2-phenyl-N-((4-(((2-(phenylthio)ethyl)(4-sulfamoylbenzyl)amino)methyl)phenyl)sulfonyl)benzofuran-5-carboxamide, SZ10TA41

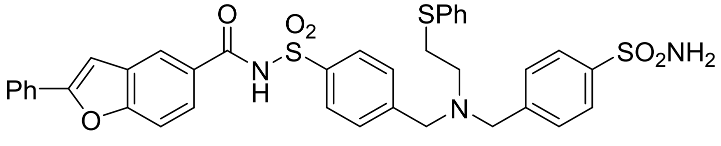

SZ10TA41

^{1}H NMR (400 MHz, DMSO-d_6) δ 8.24 (d, J = 1.7 Hz, 1H), 7.95 – 7.85 (m, 5H), 7.81 – 7.74 (m, 2H), 7.61 (d, J = 8.7 Hz, 1H), 7.59 – 7.55 (m, 2H), 7.54 – 7.46 (m, 5H), 7.46 – 7.38 (m, 1H), 7.29 (s, 2H), 7.22 – 7.10 (m, 4H), 7.09 – 6.99 (m, 1H), 3.70 (s, 2H), 3.66 (s, 2H), 3.22 – 3.08 (m, 2H), 2.67 – 2.56 (m, 2H).^{13}C NMR (101 MHz, DMSO-d_6) δ 209.71, 167.77, 156.15, 155.97, 143.30, 142.77, 135.93, 129.45, 129.08, 128.92, 128.44, 128.13, 127.58, 127.40, 127.30, 125.65, 125.45, 125.36, 124.73, 121.96, 114.21, 113.86, 110.42, 102.47, 56.87, 51.81, 29.21, 29.02. HRMS (ESI) calcd for $C_{37}H_{33}N_3O_6S_3$ [M+H]$^+$: 712.1604, found: 712.1612

3-(phenethylamino)-N-((4-(((2-(phenylthio)ethyl)(4-sulfamoylbenzyl)amino)methyl)phenyl)sulfonyl)benzamide, SZ10TA44

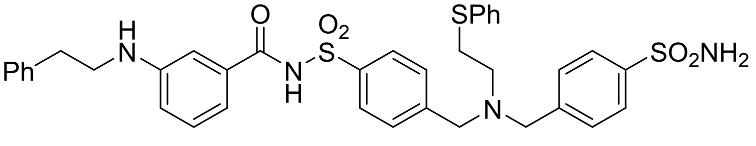

SZ10TA44

^{1}H NMR (400 MHz, DMSO-d$_6$) δ 7.91 (d, *J* = 8.2 Hz, 2H), 7.80 (d, *J* = 8.2 Hz, 2H), 7.60 – 7.52 (m, 4H), 7.36 – 7.24 (m, 7H), 7.24 – 7.00 (m, 10H), 6.77 (d, *J* = 8.2 Hz, 1H), 3.70 (s, 2H), 3.68 – 3.63 (m, 2H), 3.26 (t, *J* = 7.2 Hz, 2H), 3.19 – 3.12 (m, 2H), 2.83 (t, *J* = 7.4 Hz, 2H), 2.62 (t, *J* = 7.2 Hz, 2H). ^{13}C NMR (126 MHz, DMSO-d$_6$) δ 167.35, 148.58, 144.05, 143.28, 142.84, 142.82, 139.81, 135.92, 128.99, 128.93, 128.90, 128.72, 128.42, 128.32, 127.65, 127.61, 127.48, 126.05, 125.69, 125.41, 116.08, 115.86, 111.30, 56.90, 51.81, 44.63, 34.79, 30.72, 29.22. HRMS (ESI) calcd for $C_{37}H_{38}N_4O_5S_3$ [M+H]$^+$: 715.2077, found: 715.2121

N-((4'-(tert-butyl)-[1,1'-biphenyl]-4-yl)sulfonyl)-4-(pyridin-2-yl)benzamide, SZ11TA25

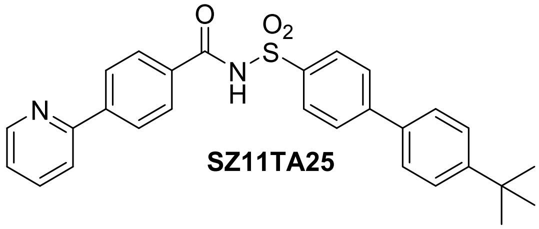

SZ11TA25

^{1}H NMR (400 MHz, DMSO-d$_6$) δ 8.69 – 8.66 (m, 1H), 8.10 – 8.05 (m, 2H), 8.04 – 7.97 (m, 3H), 7.94 (d, *J* = 8.2 Hz, 2H), 7.88 (dt, *J* = 1.8, 7.7 Hz, 1H), 7.71 (d, *J* = 8.2 Hz, 2H), 7.63 (d, *J* = 8.6 Hz, 2H), 7.50 (d, *J* = 8.6 Hz, 2H), 7.39 – 7.33 (m, 1H), 1.31 (s, 9H). ^{13}C NMR (101 MHz, DMSO-d$_6$) δ 168.6, 155.5, 150.4, 149.6, 143.7, 142.0, 140.3, 137.2, 136.5, 128.8, 127.7, 126.6, 125.9, 125.8, 125.8, 125.7, 122.8, 120.5, 34.3, 31.1. HRMS (ESI) calcd for $C_{28}H_{26}N_2O_3S$ [M+H]$^+$: 471.1737, found: 471.1748

1-(2,4-bis(trifluoromethyl)benzyl)-N-((4'-(tert-butyl)-[1,1'-biphenyl]-4-yl)sulfonyl)-1H-indole-5-carboxamide, SZ11TA30

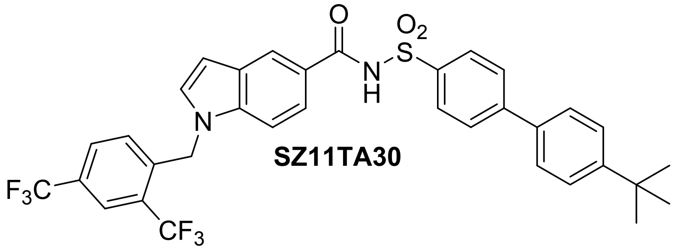

SZ11TA30

^{1}H NMR (500 MHz, DMSO-d$_6$) δ 12.39 (br. s., 1H), 8.32 (s, 1H), 8.11 (s, 1H), 8.06 (d, *J* = 8.8 Hz, 2H), 7.90 (d, *J* = 8.3 Hz, 3H), 7.72 – 7.60 (m, 4H), 7.52 (d, *J* = 8.3 Hz, 2H), 7.35 (d, *J* = 8.8 Hz, 1H), 6.77 (d, *J* = 2.5 Hz, 1H), 6.56 (d, *J* = 8.3 Hz, 1H), 3.34 (s, 2H), 1.31 (s, 9H). ^{13}C NMR (126 MHz, DMSO-d$_6$) δ 166.0, 151.2, 144.8, 141.3, 138.3, 138.2, 135.6, 131.5, 130.1, 128.7, 128.5, 128.4, 128.3, 127.7, 127.0, 127.0, 126.9, 126.8, 126.7, 126.3, 125.9, 125.9, 124.6, 124.3, 123.1,

122.7, 122.4, 122.2, 122.1, 109.9, 103.7, 45.9, 34.4, 31.0. HRMS (ESI) calcd for $C_{34}H_{28}F_6N_2O_3S$ $[M+Na]^+$: 681.1617, found: 681.1671

N-((4'-(tert-butyl)-[1,1'-biphenyl]-4-yl)sulfonyl)-3-(cyclohexanecarbonyl)-1-methyl-1H-indole-5-carboxamide, SZ11TA40

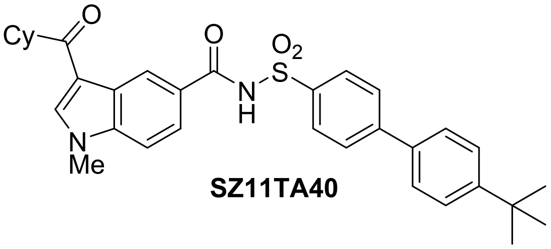

SZ11TA40

^{1}H NMR (500 MHz, DMSO-d$_6$) δ 12.72 – 12.57 (m, 1H), 8.86 (s, 1H), 8.52 (s, 1H), 8.08 (d, J = 8.8 Hz, 2H), 7.89 (d, J = 8.8 Hz, 2H), 7.77 (dd, J = 1.5, 8.8 Hz, 1H), 7.66 (d, J = 8.3 Hz, 2H), 7.60 (d, J = 8.8 Hz, 1H), 7.50 (d, J = 8.3 Hz, 2H), 3.88 (s, 3H), 3.19 – 3.10 (m, 1H), 1.78 (t, J = 12.2 Hz, 4H), 1.69 (d, J = 12.2 Hz, 1H), 1.51 – 1.34 (m, 5H), 1.30 (s, 9H). ^{13}C NMR (126 MHz, DMSO-d$_6$) δ 198.4, 166.4, 151.2, 144.7, 139.7, 139.0, 138.5, 135.6, 128.3, 126.9, 126.8, 125.9, 125.6, 125.4, 123.4, 123.1, 114.8, 110.6, 46.4, 34.3, 33.3, 31.0, 29.6, 25.6, 25.3. HRMS (ESI) calcd for $C_{33}H_{36}N_2O_4S$ $[M+H]^+$: 557.2469, found: 557.2480

4-(4,4-dimethylpiperidin-1-yl)-N-((4-(((3-nitrophenyl)amino)methyl)phenyl)sulfonyl)benzamide, SZ12TA2

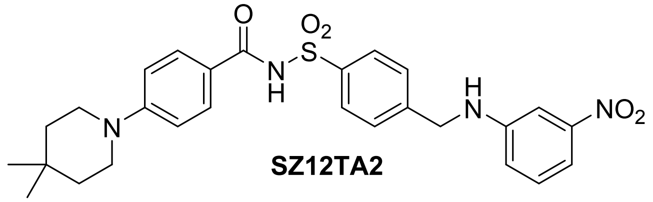

SZ12TA2

^{1}H NMR (500 MHz, DMSO-d$_6$) δ 12.05 (br. s., 1H), 7.95 (d, J = 8.8 Hz, 2H), 7.73 (d, J = 9.3 Hz, 2H), 7.59 (d, J = 8.3 Hz, 2H), 7.38 (t, J = 2.2 Hz, 1H), 7.36 – 7.33 (m, 1H), 7.32 – 7.28 (m, 1H), 7.11 (t, J = 5.9 Hz, 1H), 6.97 (dd, J = 1.5, 7.8 Hz, 1H), 6.89 (d, J = 9.3 Hz, 2H), 4.46 (d, J = 5.9 Hz, 2H), 3.34 – 3.28 (m, 4H), 1.38 – 1.32 (m, 4H), 0.92 (s, 6H). ^{13}C NMR (126 MHz, DMSO-d$_6$) δ 164.8, 153.6, 149.4, 148.8, 145.0, 139.0, 130.2, 130.1, 127.8, 127.4, 119.0, 118.3, 112.7, 110.3, 105.8, 45.8, 43.3, 37.4, 28.5, 27.6. HRMS (ESI) calcd for $C_{27}H_{30}N_4O_5S$ $[M+H]^+$: 523.2010, found: 523.2022

5-(4-ethoxy-3-fluorophenyl)-1-methyl-N-((4-(((3-nitrophenyl)amino)methyl)phenyl)sulfonyl)-1H-indole-3-carboxamide, SZ12TA23

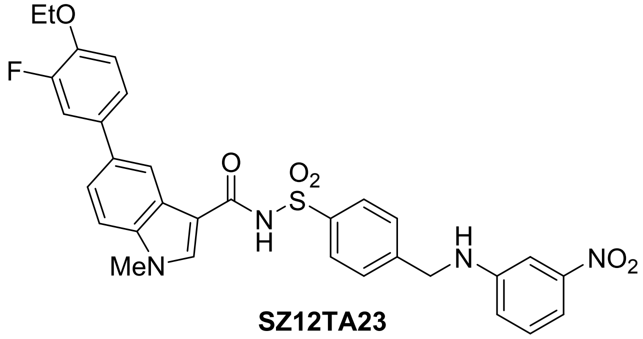

SZ12TA23

^{1}H NMR (500 MHz, DMSO-d$_6$) δ 8.30 (s, 1H), 8.19 (s, 1H), 7.97 (d, *J* = 8.3 Hz, 2H), 7.60 – 7.54 (m, 3H), 7.53 – 7.49 (m, 1H), 7.45 (dd, *J* = 2.2, 13.0 Hz, 1H), 7.39 – 7.35 (m, 2H), 7.34 – 7.30 (m, 1H), 7.29 – 7.25 (m, 1H), 7.20 (t, *J* = 8.8 Hz, 1H), 7.09 (t, *J* = 5.9 Hz, 1H), 6.95 (d, *J* = 7.8 Hz, 1H), 4.45 (d, *J* = 5.9 Hz, 2H), 4.13 (q, *J* = 7.2 Hz, 2H), 3.85 (s, 3H), 1.37 (t, *J* = 7.1 Hz, 3H). ^{13}C NMR (126 MHz, DMSO-d$_6$) δ 162.5, 151.9 (d, *J* = 243 Hz), 149.4, 148.8, 145.3 (d, *J* = 11 Hz), 144.7, 139.7, 136.4, 136.0, 134.2 (d, *J* = 7 Hz), 132.7, 130.1, 127.7, 127.4, 127.1, 122.8 (d, *J* = 3 Hz), 121.7, 118.5, 118.3, 115.2, 114.2 (d, *J* = 18 Hz), 111.2, 110.2, 105.8, 64.4, 45.8, 33.4, 14.6. HRMS (ESI) calcd for $C_{31}H_{27}FN_4O_6S$ [M+H]$^+$: 603.1708, found: 603.1713

4-((3,4-bis(2,4-difluorophenyl)-2,5-dioxo-2,5-dihydro-1H-pyrrol-1-yl)methyl)-N-((4-(((3-nitrophenyl)amino)methyl)phenyl)sulfonyl)benzamide, SZ12TA42

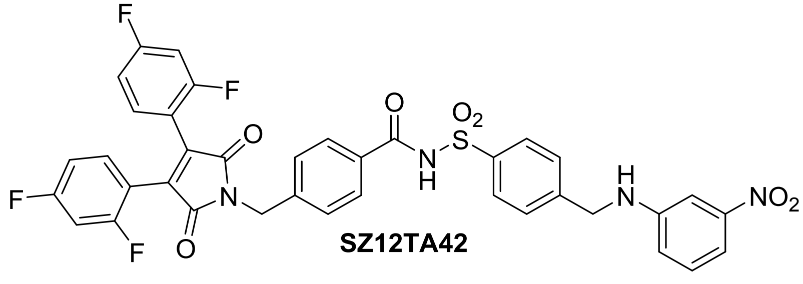

SZ12TA42

^{1}H NMR (500 MHz, DMSO-d$_6$) δ 7.89 – 7.84 (m, 4H), 7.56 (dt, *J* = 6.6, 8.4 Hz, 2H), 7.48 (d, *J* = 8.3 Hz, 2H), 7.39 – 7.32 (m, 6H), 7.31 – 7.28 (m, 1H), 7.22 (dt, *J* = 2.5, 8.6 Hz, 2H), 7.07 (t, *J* = 6.1 Hz, 1H), 6.97 (td, *J* = 1.2, 7.8 Hz, 1H), 4.79 (s, 2H), 4.42 (d, *J* = 6.4 Hz, 2H). ^{13}C NMR (126 MHz, DMSO-d$_6$) δ 168.7, 167.3, 162.6 (dd, *J* = 251, 13 Hz), 160.0 (dd, *J* = 254, 13 Hz), 149.5, 148.8, 143.2, 141.6, 139.8, 133.5, 133.0 (dd, *J* = 11, 4 Hz), 130.0, 128.7, 127.5, 127.1, 127.0, 118.3, 113.4 (dd, *J* = 16, 2 Hz), 112.1 (dd, *J* = 22, 3 Hz), 110.2, 109.56, 105.7, 104.7 (t, *J* = 26 Hz), 45.9, 41.4. HRMS (ESI) calcd for $C_{37}H_{24}F_4N_4O_7S$ [M+H]$^+$: 745.1375, found: 745.1410

3-(cyclohexanecarbonyl)-1-methyl-N-((4-(((4-oxo-2-phenyl-4H-chromen-7-yl)oxy)methyl)phenyl)sulfonyl)-1H-indole-5-carboxamide, SZ14TA40

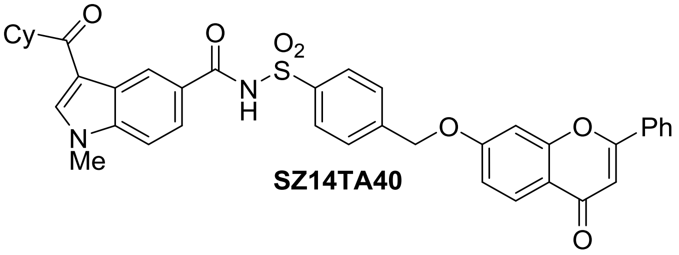

SZ14TA40

^{1}H NMR (500 MHz, DMSO-d$_6$) δ 12.69 – 12.56 (m, 1H), 8.81 (d, *J* = 1.5 Hz, 1H), 8.56 – 8.51 (m, 1H), 8.10 – 8.03 (m, 4H), 7.98 (d, *J* = 8.8 Hz, 1H), 7.79 – 7.71 (m, 3H), 7.62 – 7.55 (m, 4H), 7.46 (d, *J* = 2.5 Hz, 1H), 7.18 (dd, *J* = 2.5, 8.8 Hz, 1H), 6.98 (s, 1H), 5.42 (s, 2H), 3.88 (s, 3H), 3.13 (d, *J* = 2.9 Hz, 1H), 1.83 – 1.74 (m, 4H), 1.70 (d, *J* = 12.7 Hz, 1H), 1.51 – 1.34 (m, 5H). ^{13}C NMR (101 MHz, DMSO-d$_6$) δ 198.4, 176.4, 166.3, 162.6, 162.2, 157.4, 141.97, 141.96, 139.7, 139.4, 139.0, 131.7, 131.1, 129.1, 128.0, 127.9, 126.4, 126.2, 125.4, 123.4, 123.1, 117.5, 115.1, 114.7, 110.8, 106.9, 102.0, 69.1, 46.3, 33.3, 29.5, 25.6, 25.3. HRMS (ESI) calcd for $C_{39}H_{34}N_2O_7S$ [M+H]$^+$: 675.2160, found: 675.2218.

4-((3,4-bis(2,4-difluorophenyl)-2,5-dioxo-2,5-dihydro-1H-pyrrol-1-yl)methyl)-N-((4-(((4-oxo-2-phenyl-4H-chromen-7-yl)oxy)methyl)phenyl)sulfonyl)benzamide, SZ14TA42

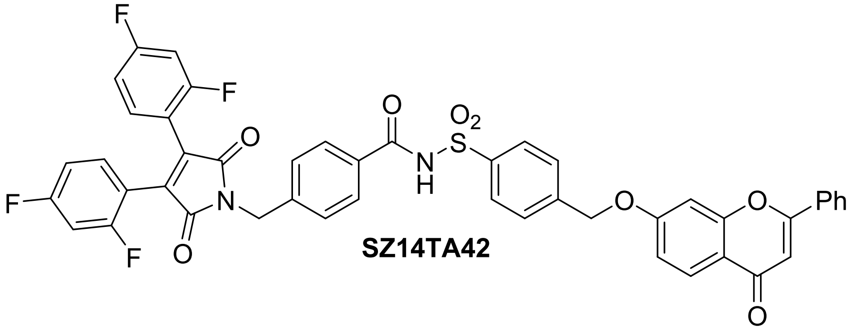

SZ14TA42

^{1}H NMR (500 MHz, DMSO-d$_6$) δ 8.12 – 8.07 (m, 2H), 7.96 (d, *J* = 8.8 Hz, 1H), 7.86 (dd, *J* = 8.3, 14.2 Hz, 4H), 7.63 – 7.53 (m, 5H), 7.50 (d, *J* = 7.8 Hz, 2H), 7.45 (d, *J* = 2.5 Hz, 1H), 7.38 – 7.31 (m, 2H), 7.29 (d, *J* = 7.8 Hz, 2H), 7.22 (dt, *J* = 2.0, 8.3 Hz, 2H), 7.16 (dd, *J* = 2.0, 8.8 Hz, 1H), 6.97 (s, 1H), 5.30 (s, 2H), 4.75 (s, 2H). ^{13}C NMR (126 MHz, DMSO-d$_6$) δ 176.4, 169.3, 168.7, 162.9, 162.4, 162.2, 157.4, 146.1, 138.7, 138.0, 137.6, 133.5, 133.0, 133.0, 131.7, 131.2, 129.1, 128.6, 127.1, 127.0, 126.6, 126.3, 126.2, 117.4, 115.2, 113.4, 112.0, 106.8, 104.7, 102.0, 69.7, 41.5. HRMS (ESI) calcd for $C_{46}H_{28}F_4N_2O_8S$ [M+Na]$^+$: 867.1394, found: 867.1398.

N-((4-(((2-chloro-6-fluorobenzyl)(2-(phenylthio)ethyl)amino)methyl)phenyl)sulfonyl)benzamide, SZ15TA1

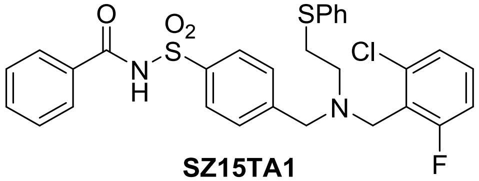

SZ15TA1

Yield = 61%. R_f = 0.6 in DCM : MeOH = 20:1. ^{1}H NMR (600 MHz, DMSO-d_6) δ 7.88 – 7.84 (m, 4H), 7.56 (t, *J* = 7.3 Hz, 1H), 7.49 (d, *J* = 8.1 Hz, 2H), 7.44 (t, *J* = 7.7 Hz, 2H), 7.32 – 7.26 (m, 2H), 7.15 (t, *J* = 7.8 Hz, 3H), 7.09 (d, *J* = 7.4 Hz, 2H), 7.01 (t, *J* = 7.2 Hz, 1H), 3.80 (s, 2H), 3.72 (s, 2H), 3.11 – 3.06 (m, 2H), 2.66 – 2.62 (m, 2H). ^{13}C NMR (101 MHz, CD_3CN) δ: 166.3, 163.1 (d, *J* = 249.0 Hz), 160.9, 160.5, 141.3, 138.0, 137.3, 134.5, 134.0, 133.7 (d, *J* = 10.0 Hz), 132.4, 132.3, 131.0, 130.3, 129.7, 129.2, 128.1, 127.1, 115.7 (d, *J* = 22.5 Hz), 58.2, 53.8, 49.8, 28.7. HRMS (ESI) calcd for $C_{29}H_{26}ClFN_2O_3S_2$ [M+H]$^+$: 569.1136, found: 569.1144.

N-((4-(((2-chloro-6-fluorobenzyl)(2-(phenylthio)ethyl)amino)methyl)phenyl)sulfonyl)-4-methyl-2-phenylthiazole-5-carboxamide, SZ15TA3

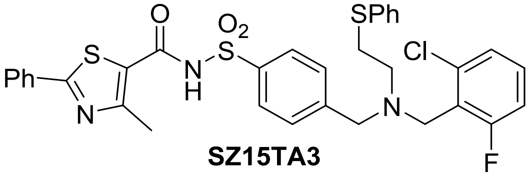

SZ15TA3

Yield = 55%. R_f = 0.29 in DCM : MeOH = 20:1. ^{1}H NMR (600 MHz, DMSO-d_6) δ 7.88 – 7.84 (m, 2H), 7.71 (d, *J* = 8.0 Hz, 2H), 7.45 – 7.40 (m, 3H), 7.32 – 7.25 (m, 4H), 7.17 – 7.10 (m, 3H), 7.03 – 6.95 (m, 3H), 3.76 (s, 2H), 3.63 (s, 2H), 3.04 – 2.98 (m, 2H), 2.60 – 2.55 (m, 2H), 2.52 (s, 3H). ^{13}C NMR (63 MHz, $CDCl_3$) δ: 169.9, 160.4 (d, *J* = 145.0 Hz), 160.3, 140.0, 137.0 (d, *J* = 12.5 Hz), 136.9, 133.0, 132.6 (d, *J* = 10.3 Hz), 132.1, 131.8, 131.1, 130.9, 129.5, 129.4, 129.3, 127.7, 127.1, 126.4, 122.1, 117.2, 116.9, 115.0 (d, *J* = 22.5 Hz), 57.6, 52.8, 49.0, 28.9, 17.8. HRMS (ESI) calcd for $C_{33}H_{29}ClFN_3O_3S_3$ [M+H]$^+$: 666.1116, found: 666.1097.

N-((4-(((2-chloro-6-fluorobenzyl)(2-(phenylthio)ethyl)amino)methyl)phenyl)sulfonyl)-3,5-dimethoxybenzamide , SZ15TA5

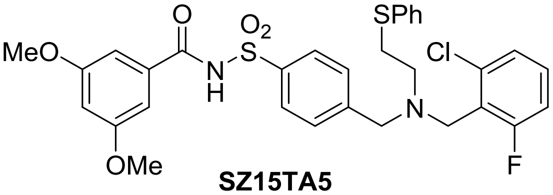

SZ15TA5

Yield = 44%. R_f = 0.57 in DCM : MeOH = 20:1. ^{1}H NMR (500 MHz, CDCl3) δ 8.01 (d, *J* = 8.3 Hz, 2H), 7.48 (d, *J* = 8.2 Hz, 2H), 7.19 – 7.04 (m, 7H), 6.93 – 6.90 (m, 1H), 6.90 – 6.87 (m, 2H), 6.61 (d, *J* = 2.2 Hz, 1H), 3.84 (s, 2H), 3.76 (s, 6H), 3.70 (s, 2H), 3.07 – 3.00 (m, 2H), 2.80 – 2.73 (m, 2H). HRMS (ESI) calcd for $C_{31}H_{30}ClFN_2O_5S_2$ [M+H]$^+$: 629.1342, found: 629.1343.

N-((4-(((2-chloro-6-fluorobenzyl)(2-(phenylthio)ethyl)amino)methyl)phenyl)sulfonyl)-1-naphthamide, SZ15TA7

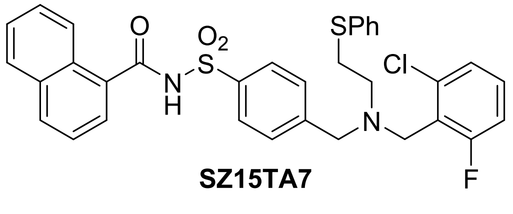

SZ15TA7

^{1}H NMR (400 MHz, CD_3OD) δ 8.14 (d, *J* = 8.3 Hz, 2H), 8.01 (d, *J* = 8.3 Hz, 1H), 7.97 (d, *J* = 8.5 Hz, 1H), 7.87 (d, *J* = 8.1 Hz, 1H), 7.72 – 7.66 (m, 3H), 7.48 (dd, *J* = 16.2, 8.7 Hz, 2H), 7.42 – 7.30 (m, 2H), 7.27 – 7.11 (m, 6H), 7.05 (t, *J* = 8.9 Hz, 1H), 4.30 (s, 2H), 4.24, (s, 2H), 3.28 – 3.21 (m, 2H), 3.16 – 3.09 (m, 2H). HRMS (ESI) calcd for $C_{33}H_{28}ClFN_2O_3S_2$ $[M+H]^+$: 619.12922, found 619.12990.

N-((4-(((2-chloro-6-fluorobenzyl)(2-(phenylthio)ethyl)amino)methyl)phenyl)sulfonyl)-4-(trifluoromethoxy)benzamide, SZ15TA8

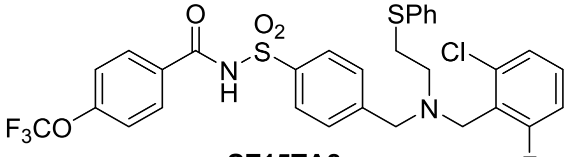

SZ15TA8

Yield = 82%. R_f = 0.52 in DCM : MeOH = 20:1. ^{1}H NMR (400 MHz, CDCl3) δ 8.00 (d, *J* = 8.2 Hz, 2H), 7.84 (d, *J* = 8.4 Hz, 2H), 7.48 (d, *J* = 8.0 Hz, 2H), 7.32 – 7.21 (m, 3H), 7.20 – 7.11 (m, 5H), 7.11 – 7.03 (m, 1H), 6.98 – 6.85 (m, 1H), 3.83 (s, 2H), 3.69 (s, 2H), 3.03 (t, *J* = 7.6 Hz, 2H), 2.74 (t, *J* = 7.7 Hz, 2H). ^{13}C NMR (101 MHz, $CDCl_3$) δ: 164.3, 162.2 (d, *J* = 250.0 Hz), 161.4 (d, *J* = 37.0 Hz), 152.9, 139.8, 137.8, 136.8, 133.2, 132.3 (d, *J* = 9.5 Hz), 131.0, 130.5, 129.6, 129.3, 129.2, 127.4, 126.2, 120.5, 120.3 (q, *J* = 259.3 Hz), 117.6 (d, *J* = 16.1 Hz), 114.8 (d, *J* = 22.6 Hz), 57.4, 52.7, 48.8, 28.7. HRMS (ESI) calcd for $C_{30}H_{25}ClF_4N_2O_4S_2$ $[M+H]^+$: 653.0953, found: 653.0935.

N-((4-(((2-chloro-6-fluorobenzyl)(2-(phenylthio)ethyl)amino)methyl)phenyl)sulfonyl)-4'-fluoro-[1,1'-biphenyl]-4-carboxamide, SZ15TA14

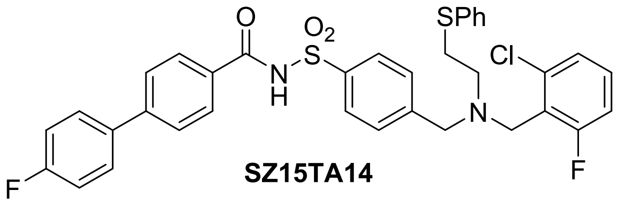

SZ15TA14

^{1}H NMR (400 MHz, $CDCl_3$) δ 8.06 (d, *J* = 7.8 Hz, 2H), 7.91 (d, *J* = 8.2 Hz, 2H), 7.59 (d, *J* = 8.2 Hz, 2H), 7.56 – 7.45 (m, 4H), 7.21 – 7.05 (m, 9H), 6.92 (d, *J* = 4.7 Hz, 1H), 3.86 (br. s., 2H), 3.72 (br. s., 2H), 3.05 (br. s., 2H), 2.78 (br. s., 2H). ^{13}C NMR (126 MHz, DMSO-d_6) δ 165.0, 163.5 (d, *J* = 240 Hz), 162.5 (d, *J* = 256 Hz), 160.5, 145.6, 143.5, 137.9, 135.7, 135.4 (d, *J* = 6 Hz), 135.1 (d, *J* = 4 Hz), 130.3, 130.1, 129.14, 129.08, 128.9, 128.8, 127.7, 127.5, 126.7, 125.6 (d, *J* = 4 Hz), 125.4, 115.9 (d, *J* = 23 Hz), 114.3 (d, *J* = 24 Hz), 56.9, 52.4, 48.9, 29.2. HRMS (ESI) calcd for $C_{35}H_{29}ClF_2N_2O_3S_2$ $[M+H]^+$: 663.1348, found: 663.1389

2-(9H-carbazol-9-yl)-N-((4-(((2-chloro-6-fluorobenzyl)(2-(phenylthio)ethyl)amino)methyl)phenyl)sulfonyl)acetamide, SZ15TA15

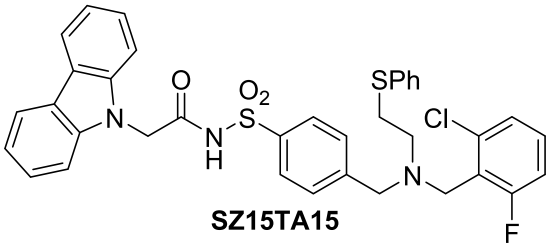

SZ15TA15

^{1}H NMR (500 MHz, DMSO-d$_6$) δ 8.09 (d, *J* = 7.8 Hz, 2H), 7.78 (d, *J* = 8.3 Hz, 2H), 7.44 (d, *J* = 8.3 Hz, 2H), 7.36 – 7.25 (m, 6H), 7.21 – 7.06 (m, 8H), 5.08 (s, 2H), 3.76 (s, 2H), 3.69 (s, 2H), 3.04 – 3.00 (m, 2H), 2.65 – 2.60 (m, 2H). ^{13}C NMR (126 MHz, DMSO-d$_6$) δ 168.5, 162.0 (d, *J* = 248 Hz), 145.0, 140.9, 139.7, 136.2, 135.9 (d, *J* = 6 Hz), 130.6 (d, *J* = 10 Hz), 129.4, 129.0, 128.2, 127.5, 126.04 (d, *J* = 4 Hz), 126.00, 125.9, 124.4 (d, *J* = 17 Hz), 122.6, 120.5, 119.4, 114.8 (d, *J* = 23 Hz), 109.5, 57.2, 52.6, 49.2, 46.5, 29.7. HRMS (ESI) calcd for $C_{36}H_{31}ClFN_3O_3S_2$ $[M+H]^+$: 672.1552, found: 672.1565

N-((4-(((2-chloro-6-fluorobenzyl)(2-(phenylthio)ethyl)amino)methyl)phenyl)sulfonyl)-4-(2-(pyridin-2-yl)ethyl)benzamide, SZ15TA17

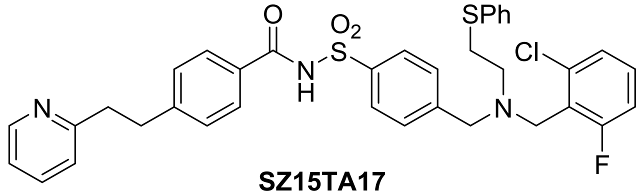

SZ15TA17

^{1}H NMR (500 MHz, DMSO-d$_6$) δ 12.53 – 12.29 (m, 1H), 8.48 (d, *J* = 3.9 Hz, 1H), 7.86 (d, *J* = 8.3 Hz, 2H), 7.77 (d, *J* = 7.8 Hz, 2H), 7.64 (dt, *J* = 2.0, 7.6 Hz, 1H), 7.49 (d, *J* = 8.3 Hz, 2H), 7.32 – 7.25 (m, 4H), 7.22 – 7.15 (m, 3H), 7.13 (d, *J* = 7.3 Hz, 2H), 7.11 – 7.06 (m, 2H), 7.02 – 6.97 (m, 1H), 3.79 (s, 2H), 3.71 (s, 2H), 3.11 – 3.06 (m, 2H), 3.05 – 3.01 (m, 4H), 2.66 – 2.61 (m, 2H). ^{13}C NMR (126 MHz, DMSO-d$_6$) δ 165.9, 161.5 (d, *J* = 248 Hz), 160.3, 148.9, 146.5, 144.7, 139.1, 136.4, 135.7, 135.5, 135.4 (d, *J* = 6 Hz), 130.1 (d, *J* = 10 Hz), 128.9, 128.5, 128.4, 128.4, 127.7, 127.4, 125.6 (d, *J* = 3 Hz), 125.4, 124.1 (d, *J* = 17 Hz), 122.9, 121.4, 114.3 (d, *J* = 23 Hz), 56.9, 52.3, 48.9, 38.5, 34.7, 29.2. HRMS (ESI) calcd for $C_{36}H_{33}ClFN_3O_3S_2$ $[M+H]^+$: 674.1709, found: 674.1751

N-((4-(((2-chloro-6-fluorobenzyl)(2-(phenylthio)ethyl)amino)methyl)phenyl)sulfonyl)-4-(2,3,4-trimethoxybenzyl)benzamide, SZ15A24

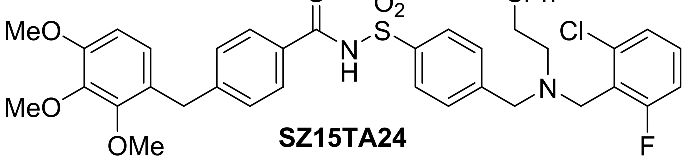

SZ15TA24

^{1}H NMR (500 MHz, DMSO-d$_6$) δ 12.51 – 12.26 (m, 1H), 7.90 – 7.86 (m, 2H), 7.80 – 7.75 (m, 2H), 7.52 (d, *J* = 8.3 Hz, 2H), 7.32 – 7.23 (m, 4H), 7.16 – 7.10 (m, 3H), 7.10 – 7.06 (m, 2H), 7.01 – 6.96 (m, 1H), 6.86 (d, *J* = 8.3 Hz, 1H), 6.72 (d, *J* = 8.3 Hz, 1H), 3.88 (s, 2H), 3.78 (s, 2H), 3.75 (s, 3H),

3.72 (s, 2H), 3.70 (s, 3H), 3.61 (s, 3H), 3.11 – 3.05 (m, 2H), 2.66 – 2.60 (m, 2H). ^{13}C NMR (126 MHz, DMSO-d$_6$) δ 165.2, 161.5 (d, *J* = 248 Hz), 152.3, 151.2, 147.2, 145.4, 141.9, 138.1, 135.7, 135.4 (d, *J* = 6 Hz), 130.1 (d, *J* = 10 Hz), 129.3, 128.8, 128.7, 128.6, 128.5, 127.7, 127.5, 125.8, 125.6 (d, *J* = 3 Hz), 125.4, 124.5, 124.0 (d, *J* = 17 Hz), 114.3 (d, *J* = 23 Hz), 107.7, 60.4, 60.2, 56.9, 55.8, 52.4, 48.9, 35.2, 29.2. HRMS (ESI) calcd for $C_{39}H_{38}ClFN_2O_6S_2$ [M+H]$^+$: 749.1917, found: 749.1938

N-((4-(((2-chloro-6-fluorobenzyl)(2-(phenylthio)ethyl)amino)methyl)phenyl)sulfonyl)-4-(pyridin-2-yl)benzamide, SZ15TA25

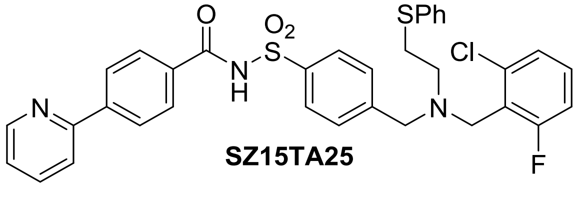

SZ15TA25

^{1}H NMR (500 MHz, DMSO-d$_6$) δ 8.69 (d, *J* = 4.9 Hz, 1H), 8.19 (d, *J* = 8.8 Hz, 2H), 8.03 (d, *J* = 8.3 Hz, 1H), 8.00 (d, *J* = 8.3 Hz, 2H), 7.94 – 7.86 (m, 3H), 7.52 (d, *J* = 8.3 Hz, 2H), 7.41 – 7.37 (m, 1H), 7.31 – 7.23 (m, 2H), 7.17 – 7.06 (m, 5H), 7.04 – 6.99 (m, 1H), 3.79 – 3.75 (m, 2H), 3.72 (s, 2H), 3.12 – 3.04 (m, 2H), 2.68 – 2.60 (m, 2H). ^{13}C NMR (126 MHz, DMSO-d$_6$) δ 165.4, 161.5 (d, *J* = 249 Hz), 154.6, 149.7, 145.1, 142.5, 138.6, 137.4, 135.7, 135.5 (d, *J* = 6 Hz), 132.5, 130.1 (d, *J* = 10 Hz), 128.9, 128.9, 128.7, 127.7, 127.5, 126.4, 125.6 (d, *J* = 3 Hz), 125.4, 124.0 (d, *J* = 17 Hz), 123.4, 120.9, 114.3 (d, *J* = 23 Hz), 56.9, 52.4, 48.9, 29.2. HRMS (ESI) calcd for $C_{34}H_{29}ClFN_3O_3S_2$ [M+H]$^+$: 646.1396, found: 646.1401

N-((4-(((2-chloro-6-fluorobenzyl)(2-(phenylthio)ethyl)amino)methyl)phenyl)sulfonyl)-2-(3-oxo-3,4-dihydro-2H-benzo[b][1,4]thiazin-2-yl)acetamide, SZ15TA34

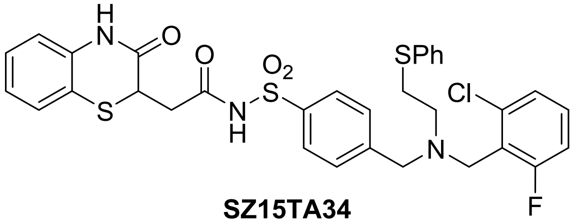

SZ15TA34

^{1}H NMR (500 MHz, DMSO-d$_6$) δ 10.63 (s, 1H), 7.79 (d, *J* = 8.3 Hz, 2H), 7.48 (d, *J* = 8.3 Hz, 2H), 7.37 – 7.30 (m, 1H), 7.30 – 7.26 (m, 1H), 7.23 (d, *J* = 7.8 Hz, 1H), 7.21 – 7.13 (m, 4H), 7.13 – 7.06 (m, 3H), 6.98 – 6.93 (m, 2H), 3.79 (s, 2H), 3.75 (dd, *J* = 5.9, 8.3 Hz, 1H), 3.72 (s, 2H), 3.12 – 3.05 (m, 2H), 2.82 (dd, *J* = 5.6, 16.4 Hz, 1H), 2.68 – 2.61 (m, 2H), 2.40 (dd, *J* = 8.3, 16.1 Hz, 1H). ^{13}C NMR (126 MHz, DMSO-d$_6$) δ 169.2, 165.8, 161.6, (d, *J* = 249 Hz), 144.5, 139.2, 136.8, 135.7, 135.5 (d, *J* = 6 Hz), 130.2 (d, *J* = 11 Hz), 128.9, 128.6, 127.7, 127.5, 127.2, 127.1, 125.6 (d, *J* = 3 Hz), 125.4, 124.0 (d, *J* = 17 Hz), 123.0, 118.2, 117.1, 114.4 (d, *J* = 23 Hz), 56.9, 52.2, 48.9, 37.1, 35.7, 29.3. HRMS (ESI) calcd for $C_{32}H_{29}ClFN_3O_4S_3$ [M+H]$^+$: 670.1066, found: 670.1025

3-(phenethylamino)-N-((4'-(trifluoromethyl)-[1,1'-biphenyl]-4-yl)sulfonyl)benzamide, SZ16TA44

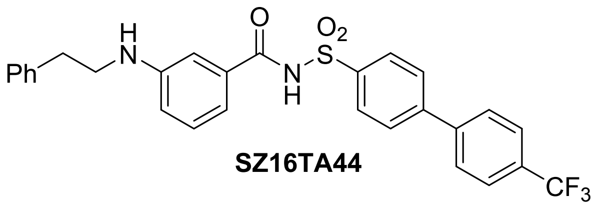

SZ16TA44

^{1}H NMR (500 MHz, DMSO-d$_6$) δ 8.10 (d, *J* = 8.3 Hz, 2H), 8.00 – 7.92 (m, 4H), 7.85 (d, *J* = 8.3 Hz, 2H), 7.33 – 7.24 (m, 4H), 7.22 – 7.14 (m, 2H), 7.09 – 7.04 (m, 2H), 6.85 – 6.78 (m, 1H), 6.12 – 5.79 (m, 1H), 3.27 (t, *J* = 7.3 Hz, 2H), 2.83 (t, *J* = 7.6 Hz, 2H). ^{13}C NMR (126 MHz, DMSO-d$_6$) δ 166.6, 148.8, 143.2, 142.6, 140.0, 139.8, 132.9, 129.1, 129.0, 128.8, 128.5, 128.4, 128.1, 127.7, 126.1, 126.0, 124.3 (q, J = 271 Hz), 116.9, 115.8, 111.0, 44.6, 34.8. HRMS (ESI) calcd for $C_{28}H_{23}F_3N_2O_3S$ [M+H]$^+$: 525.1454, found: 525.1491

N-((4-(((3-methoxybenzyl)(2-(phenylthio)ethyl)amino)methyl)phenyl)sulfonyl)-4-methyl-2-phenylthiazole-5-carboxamide, SZ17TA3

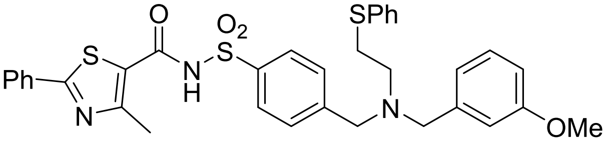

SZ17TA3

Yield = 80%. R$_f$ = 0.26 in DCM : MeOH = 20:1. ^{1}H NMR (600 MHz, DMSO-d$_6$) δ 7.88 – 7.84 (m, 2H), 7.75 (d, *J* = 7.8 Hz, 2H), 7.44 – 7.41 (m, 3H), 7.36 (d, *J* = 7.8 Hz, 2H), 7.21 – 7.12 (m, 3H), 7.09 (d, *J* = 8.0 Hz, 2H), 7.01 (t, *J* = 7.2 Hz, 1H), 6.94 – 6.88 (m, 2H), 6.76 (d, *J* = 7.9 Hz, 1H), 3.70 (s, 3H), 3.59 (s, 2H), 3.55 (s, 2H), 3.12 – 3.06 (m, 2H), 2.61 – 2.55 (m, 2H), 2.53 (s, 3H). ^{13}C NMR (101 MHz, CD_3OD) δ 170.7, 163.2, 161.7, 161.2, 143.3, 136.1, 134.1, 133.6, 132.8, 132.5, 132.1, 131.8, 131.6, 130.5, 130.3, 130.0, 128.8, 127.9, 125.6, 124.2, 117.4, 117.0, 59.3, 58.0, 56.0, 52.5, 28.8, 17.9. HRMS (ESI) calcd for $C_{34}H_{33}N_3O_4S_3$ [M+H]$^+$: 644.1706, found: 644.1730

N-((4-(((3-methoxybenzyl)(2-(phenylthio)ethyl)amino)methyl)phenyl)sulfonyl)-1-naphthamide, SZ17TA7

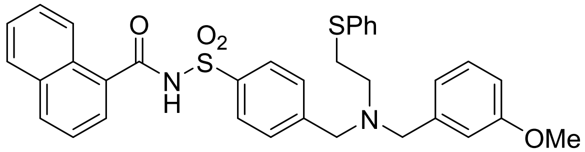

SZ17TA7

^{1}H NMR (400 MHz, DMSO-d$_6$) δ 8.48 (td, *J* = 0.9, 4.8 Hz, 1H), 7.89 (d, *J* = 7.8 Hz, 2H), 7.78 (d, *J* = 8.2 Hz, 2H), 7.64 (dt, *J* = 2.0, 7.6 Hz, 1H), 7.52 (d, *J* = 8.2 Hz, 2H), 7.26 – 7.12 (m, 8H), 7.04 – 6.99 (m, 1H), 6.97 – 6.92 (m, 2H), 6.80 (dd, *J* = 2.7, 8.2 Hz, 1H), 3.73 (s, 3H), 3.66 (s, 2H), 3.60 (s, 2H), 3.16 – 3.10 (m, 2H), 2.65 – 2.59 (m, 2H). HRMS (ESI) calcd for $C_{34}H_{32}N_2O_4S_2$ [M+H]$^+$: 597.1876, found: 597.1877

N-((4-(((3-methoxybenzyl)(2-(phenylthio)ethyl)amino)methyl)phenyl)sulfonyl)-4-(trifluoromethoxy)benzamide, SZ17TA8

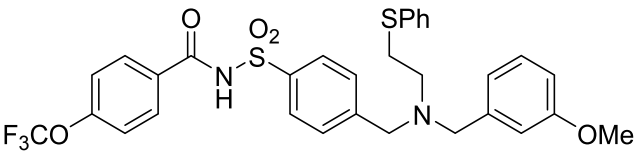

SZ17TA8

Yield = 68%. R_f = 0.46 in DCM : MeOH = 20:1. ^{1}H NMR (600 MHz, DMSO-d_6) δ 7.95 (d, *J* = 8.8 Hz, 2H), 7.81 (d, *J* = 8.2 Hz, 2H), 7.44 (d, *J* = 8.1 Hz, 2H), 7.31 (d, *J* = 8.3 Hz, 2H), 7.19 (t, *J* = 7.8 Hz, 1H), 7.14 – 7.07 (m, 4H), 6.98 (t, *J* = 7.2 Hz, 1H), 6.93 – 6.88 (m, 2H), 6.76 (d, *J* = 6.1 Hz, 1H), 3.70 (s, 3H), 3.62 (s, 2H), 3.57 (s, 2H), 3.12 – 3.06 (m, 2H), 2.61 – 2.55 (m, 2H). ^{13}C NMR (101 MHz, $CDCl_3$) δ 164.5, 161.9, 161.5, 160.6, 153.1, 140.7, 134.7, 132.5, 131.7, 131.3, 130.8, 130.6, 129.6, 129.6, 128.1, 123.0, 120.6, 120.4 (q, *J* = 259.8 Hz), 116.6, 115.9, 58.2, 57.0, 55.4, 51.3, 28.3. HRMS (ESI) calcd for $C_{31}H_{29}F_3N_2O_5S_2$ $[M+H]^+$: 631.1543, found: 631.1534.

4'-fluoro-N-((4-(((3-methoxybenzyl)(2-(phenylthio)ethyl)amino)methyl)phenyl)sulfonyl)-[1,1'-biphenyl]-4-carboxamide, SZ17TA14

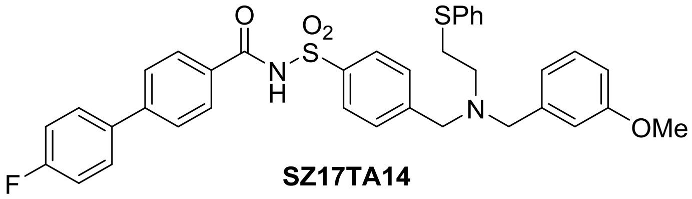

SZ17TA14

^{1}H NMR (400 MHz, $CDCl_3$) δ 8.11 (d, *J* = 8.20 Hz, 2H), 7.94 (d, *J* = 8.59 Hz, 2H), 7.56 (dd, *J* = 4.30, 8.20 Hz, 4H), 7.51 (dd, *J* = 5.27, 8.79 Hz, 2H), 7.19 - 7.24 (m, 1H), 7.16 - 7.19 (m, 4H), 7.11 (t, *J* = 8.79 Hz, 3H), 6.96 (s, 1H), 6.93 (d, *J* = 7.42 Hz, 1H), 6.79 (dd, *J* = 2.34, 8.20 Hz, 1H), 3.80 (s, 3H), 3.67 (s, 2H), 3.61 (s, 2H), 3.04 (t, *J* = 7.42 Hz, 2H), 2.75 (t, *J* = 7.40 Hz, 2H). ^{13}C NMR (101 MHz, $CDCl_3$) δ 164.4, 162.9, 162.9, 159.6, 144.9, 137.2, 136.1, 135.5, 129.9, 129.3, 128.9, 128.9, 128.8, 128.8, 128.7, 128.7, 128.5, 127.1, 125.8, 121.0, 115.8, 114.3, 112.6, 58.4, 57.8, 55.1, 52.7, 31.2. HRMS (ESI) calcd for $C_{36}H_{33}FN_2O_4S_2$ $[M+H]^+$: 641.1939, found: 641.1977

2-(9H-carbazol-9-yl)-N-((4-(((3-methoxybenzyl)(2-(phenylthio)ethyl)amino)methyl)phenyl)sulfonyl)acetamide, SZ17TA15

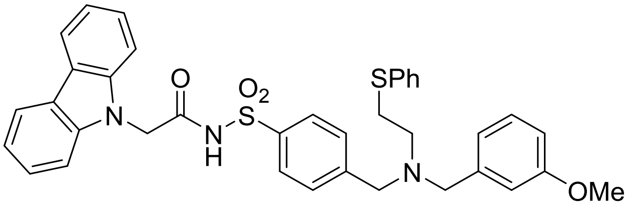

SZ17TA15

^{1}H NMR (500 MHz, DMSO-d_6) δ 8.12 – 8.08 (m, 2H), 7.73 (d, *J* = 8.3 Hz, 2H), 7.40 – 7.35 (m, 3H), 7.35 (br. s., 3H), 7.27 – 7.20 (m, 3H), 7.19 – 7.15 (m, 2H), 7.15 – 7.08 (m, 3H), 7.00 – 6.97 (m, 1H), 6.95 (d, *J* = 7.3 Hz, 1H), 6.83 (dd, *J* = 2.0, 8.3 Hz, 1H), 4.81 (s, 2H), 3.76 (s, 3H), 3.65 – 3.60 (m, 2H), 3.57 (s, 2H), 3.17 – 3.11 (m, 2H), 2.67 – 2.61 (m, 2H). ^{13}C NMR (126 MHz, DMSO-d_6) δ 172.1, 159.3, 144.4, 141.4, 140.8, 140.7, 136.3, 129.3, 128.9, 127.7, 127.4, 126.5, 125.3,

125.3, 122.0, 120.7, 119.9, 118.4, 113.9, 112.4, 109.4, 57.3, 56.9, 54.9, 51.7, 48.3, 29.4. HRMS (ESI) calcd for $C_{37}H_{35}N_3O_4S_2$ $[M+H]^+$: 650.2142, found: 650.2160

N-((4-(((3-methoxybenzyl)(2-(phenylthio)ethyl)amino)methyl)phenyl)sulfonyl)-4-(2-(pyridin-2-yl)ethyl)benzamide, SZ17TA17

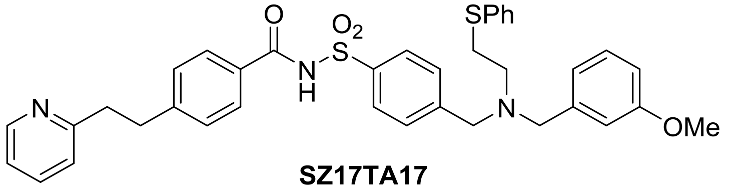

SZ17TA17

^{1}H NMR (400 MHz, DMSO-d$_6$) δ 8.48 (td, *J* = 0.9, 4.8 Hz, 1H), 7.89 (d, *J* = 7.8 Hz, 2H), 7.78 (d, *J* = 8.2 Hz, 2H), 7.64 (dt, *J* = 2.0, 7.6 Hz, 1H), 7.52 (d, *J* = 8.2 Hz, 2H), 7.26 – 7.22 (m, 3H), 7.20 (d, *J* = 7.0 Hz, 2H), 7.18 – 7.11 (m, 4H), 7.05 – 6.99 (m, 1H), 6.97 – 6.92 (m, 2H), 6.80 (dd, *J* = 2.7, 8.2 Hz, 1H), 3.73 (s, 3H), 3.66 (s, 2H), 3.60 (s, 2H), 3.16 – 3.10 (m, 2H), 3.02 (s, 4H), 2.65 – 2.58 (m, 2H). ^{13}C NMR (126 MHz, DMSO-d$_6$) δ 166.9, 160.4, 159.3, 148.9, 145.7, 143.8, 140.6, 136.4, 136.1, 129.3, 128.9, 128.5, 128.3, 128.1, 127.44, 127.36, 125.2, 122.9, 121.3, 120.7, 114.0, 112.4, 57.4, 56.9, 54.9, 51.8, 38.7, 34.8, 29.2. HRMS (ESI) calcd for $C_{37}H_{37}N_3O_4S_2$ $[M+H]^+$: 652.2298, found: 652.2335

4-(3,4-bis(2,4-difluorophenyl)-2,5-dioxo-2,5-dihydro-1H-pyrrol-1-yl)-N-((4-(((3-methoxybenzyl)(2-(phenylthio)ethyl)amino)methyl)phenyl)sulfonyl)butanamide, SZ17TA20

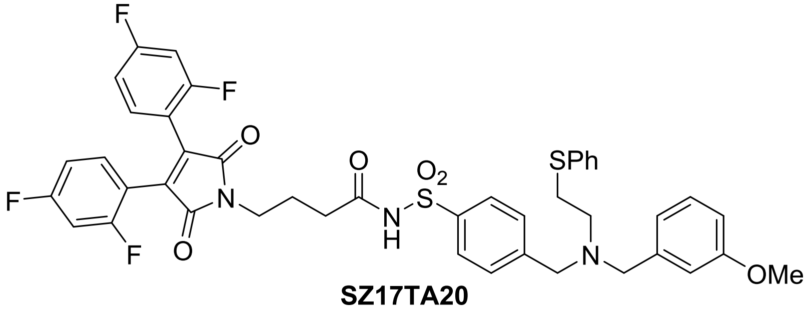

SZ17TA20

^{1}H NMR (500 MHz, DMSO-d$_6$) δ 12.12 – 11.89 (m, 1H), 7.89 (d, *J* = 8.3 Hz, 2H), 7.60 (d, *J* = 7.8 Hz, 2H), 7.58 – 7.51 (m, 2H), 7.35 – 7.29 (m, 2H), 7.26 – 7.12 (m, 7H), 7.11 – 7.06 (m, 1H), 6.95 (s, 1H), 6.92 (d, *J* = 7.3 Hz, 1H), 6.79 (dd, *J* = 2.5, 8.3 Hz, 1H), 3.73 (s, 3H), 3.68 (s, 2H), 3.58 (s, 2H), 3.48 (t, *J* = 6.9 Hz, 2H), 3.16 – 3.10 (m, 2H), 2.65 – 2.59 (m, 2H), 2.32 (t, *J* = 7.3 Hz, 2H), 1.79 – 1.70 (m, 2H). ^{13}C NMR (126 MHz, DMSO-d$_6$) δ 171.0, 168.9, 162.6 (dd, *J* = 251, 13 Hz), 160.6 (dd, *J* = 255, 13 Hz), 159.3, 145.7, 140.6, 137.9, 136.1, 133.1, 132.9 (dd, *J* = 10, 4 Hz), 129.2, 128.8, 128.8, 127.5, 127.5, 125.3, 120.7, 114.0, 113.6 (dd, *J* = 15, 2 Hz), 112.4, 112.0 (dd, *J* = 22, 3 Hz), 104.6 (t, *J* = 26 Hz), 57.4, 56.8, 54.9, 51.9, 37.4, 32.7, 29.3, 22.8. HRMS (ESI) calcd for $C_{43}H_{37}F_4N_3O_6S_2$ $[M+H]^+$: 832.2133, found: 832.2080

N-((4-(((3-methoxybenzyl)(2-(phenylthio)ethyl)amino)methyl)phenyl)sulfonyl)-4-(2,3,4-trimethoxybenzyl)benzamide, SZ17TA24

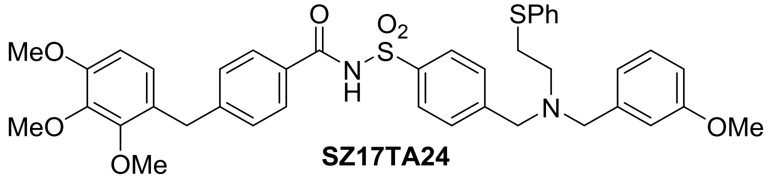

SZ17TA24

^{1}H NMR (400 MHz, DMSO-d$_6$) δ 7.92 (d, J = 8.2 Hz, 2H), 7.79 (d, J = 8.6 Hz, 2H), 7.58 (d, J = 8.6 Hz, 2H), 7.27 – 7.19 (m, 3H), 7.18 – 7.11 (m, 4H), 7.04 – 6.97 (m, 1H), 6.97 – 6.91 (m, 2H), 6.85 (d, J = 8.6 Hz, 1H), 6.80 (dd, J = 2.2, 8.0 Hz, 1H), 6.71 (d, J = 8.6 Hz, 1H), 3.88 (s, 2H), 3.75 (s, 3H), 3.73 (s, 3H), 3.71 (s, 3H), 3.68 (s, 2H), 3.61 (s, 5H), 3.17 – 3.10 (m, 2H), 2.61 (t, J = 7.2 Hz, 2H). ^{13}C NMR (101 MHz, DMSO-d$_6$) δ 165.8, 159.3, 152.3, 151.2, 146.7, 144.9, 141.8, 140.5, 139.0, 136.0, 129.2, 128.8, 128.6, 128.49, 128.46, 127.6, 127.5, 125.9, 125.2, 124.5, 120.7, 114.0, 112.4, 107.7, 60.4, 60.2, 57.4, 56.8, 55.8, 54.9, 51.8, 35.1, 29.2. HRMS (ESI) calcd for $C_{40}H_{42}N_2O_7S_2$ [M+H]$^+$: 727.2506, found: 727.2536

N-((4-(((3-methoxybenzyl)(2-(phenylthio)ethyl)amino)methyl)phenyl)sulfonyl)-4-(pyridin-2-yl)benzamide, SZ17TA25

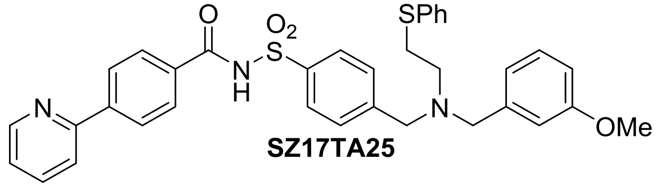

SZ17TA25

^{1}H NMR (400 MHz, DMSO-d$_6$) δ 8.69 (d, J = 4.7 Hz, 1H), 8.17 (d, J = 8.6 Hz, 2H), 8.05 – 7.98 (m, 3H), 7.95 (d, J = 8.2 Hz, 2H), 7.90 (dt, J = 1.6, 7.8 Hz, 1H), 7.58 (d, J = 8.2 Hz, 2H), 7.39 (dd, J = 4.7, 7.4 Hz, 1H), 7.25 – 7.19 (m, 1H), 7.19 – 7.12 (m, 4H), 7.06 – 7.00 (m, 1H), 6.98 – 6.92 (m, 2H), 6.80 (dd, J = 2.3, 8.2 Hz, 1H), 3.73 (s, 3H), 3.69 (s, 2H), 3.61 (s, 2H), 3.14 (t, J = 7.2 Hz, 2H), 2.63 (t, J = 7.4 Hz, 2H). ^{13}C NMR (126 MHz, DMSO-d$_6$) δ 166.0, 162.3, 159.3, 154.8, 149.7, 142.1, 140.5, 139.6, 137.4, 136.0, 129.3, 128.93, 128.86, 128.5, 127.6, 127.5, 126.3, 125.3, 123.3, 120.8, 120.7, 114.0, 112.4, 57.4, 56.8, 54.9, 51.8, 29.2. HRMS (ESI) calcd for $C_{35}H_{33}N_3O_4S_2$ [M+H]$^+$: 624.1985, found: 624.1980

4-(di(1H-indol-3-yl)methyl)-N-((4-(((3-methoxybenzyl)(2-(phenylthio)ethyl)amino)methyl)phenyl)sulfonyl)benzamide, SZ17TA31

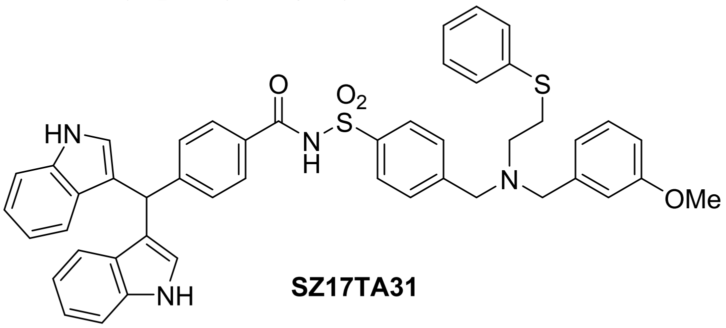

SZ17TA31

^{1}H NMR (500 MHz, CDCl$_3$) δ 8.05 (d, J = 8.1 Hz, 2H), 7.94 (s, 2H), 7.59 (d, J = 8.0 Hz, 2H), 7.51 (d, J = 8.1 Hz, 2H), 7.32 (d, J = 8.1 Hz, 4H), 7.28 (d, J = 8.0 Hz, 2H), 7.21 (t, J = 7.8 Hz, 1H), 7.18

– 7.12 (m, 6H), 7.10 – 7.04 (m, 1H), 6.97 (t, *J* = 7.5 Hz, 2H), 6.95 – 6.89 (m, 2H), 6.81 – 6.75 (m, 1H), 6.54 (s, 2H), 5.86 (s, 1H), 3.79 (s, 3H), 3.62 (s, 2H), 3.58 (s, 2H), 3.01 (dd, *J* = 8.6, 6.1 Hz, 2H), 2.72 (dd, *J* = 8.6, 6.1 Hz, 2H).

3-(cyclohexanecarbonyl)-N-((4-(((3-methoxybenzyl)(2-(phenylthio)ethyl)amino)methyl)phenyl)sulfonyl)-1-methyl-1H-indole-5-carboxamide, SZ17TA40

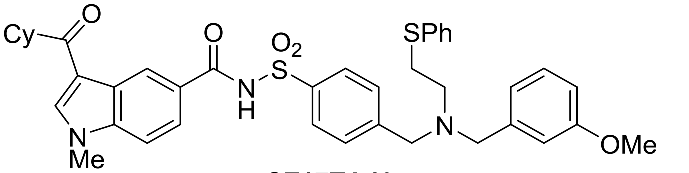

SZ17TA40

^{1}H NMR (500 MHz, DMSO-d_6) δ 12.55 (br. s., 1H), 8.84 (s, 1H), 8.51 (s, 1H), 7.97 (d, *J* = 8.3 Hz, 2H), 7.75 (d, *J* = 8.8 Hz, 1H), 7.65 – 7.58 (m, 3H), 7.26 – 7.18 (m, 1H), 7.18 – 7.09 (m, 4H), 7.02 (t, *J* = 6.4 Hz, 1H), 6.98 – 6.91 (m, 2H), 6.79 (d, *J* = 7.8 Hz, 1H), 3.87 (s, 3H), 3.76 – 3.71 (m, 3H), 3.69 (br. s., 2H), 3.64 – 3.58 (m, 2H), 3.14 (t, *J* = 7.1 Hz, 3H), 2.62 (t, *J* = 6.6 Hz, 2H), 1.78 (t, *J* = 11.5 Hz, 4H), 1.69 (d, *J* = 12.2 Hz, 1H), 1.53 – 1.32 (m, 5H). ^{13}C NMR (126 MHz, DMSO-d_6) δ 198.4, 166.2, 159.3, 140.5, 139.7, 139.0, 138.4, 136.0, 129.2, 128.8, 128.7, 127.7, 127.5, 127.2, 125.6, 125.4, 125.3, 123.4, 123.1, 120.7, 114.8, 114.0, 112.4, 110.6, 57.4, 56.8, 51.8, 46.4, 33.3, 30.7, 29.6, 29.2, 25.6, 25.3. HRMS (ESI) calcd for $C_{40}H_{43}N_3O_5S_2$ $[M+H]^+$: 710.2717, found: 710.2779

N-((4-(((3-methoxybenzyl)(2-(phenylthio)ethyl)amino)methyl)phenyl)sulfonyl)-1-methyl-3-(thiophene-2-carbonyl)-1H-indole-5-carboxamide, SZ17TA45

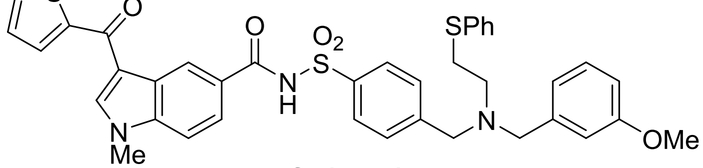

SZ17TA45

^{1}H NMR (400 MHz, DMSO-d_6) δ 12.77 – 12.33 (m, 1H), 8.86 (br. s., 1H), 8.52 (s, 1H), 7.97 (d, *J* = 8.6 Hz, 4H), 7.82 (d, *J* = 7.0 Hz, 1H), 7.69 – 7.56 (m, 3H), 7.29 (br. s., 1H), 7.22 (t, *J* = 7.6 Hz, 1H), 7.14 (br. s., 4H), 7.02 (br. s., 1H), 6.98 – 6.90 (m, 2H), 6.79 (d, *J* = 6.3 Hz, 1H), 3.93 (br. s., 3H), 3.73 (s, 3H), 3.69 (br. s., 2H), 3.61 (br. s., 2H), 3.14 (br. s., 2H), 2.62 (br. s., 2H). ^{13}C NMR (101 MHz, DMSO-d_6) δ 180.1, 166.3, 162.3, 159.2, 144.8, 140.4, 139.43, 139.37, 139.28, 138.54, 136.0, 132.9, 131.7, 129.2, 128.8, 128.6, 128.4, 127.6, 127.5, 126.0, 125.3, 123.5, 123.2, 120.7, 114.1, 114.0, 112.4, 110.7, 57.4, 56.8, 51.8, 35.8, 33.4, 29.2. HRMS (ESI) calcd for $C_{38}H_{35}N_3O_5S_3$ $[M+H]^+$: 710.1812, found: 710.1830

N-((5-(dimethylamino)naphthalen-1-yl)sulfonyl)-5-(4-ethoxy-3-fluorophenyl)-1-methyl-1H-indole-3-carboxamide, SZ21TA23

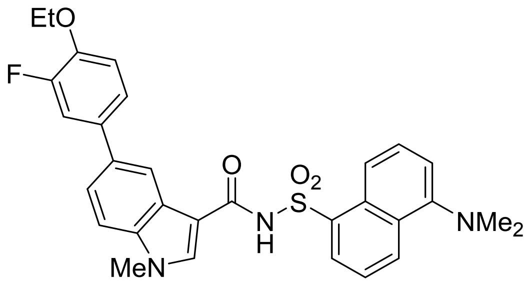

SZ21TA23

^{1}H NMR (500 MHz, DMSO-d$_6$) δ 12.37 – 12.19 (m, 1H), 8.52 (d, *J* = 8.3 Hz, 1H), 8.43 (d, *J* = 8.3 Hz, 1H), 8.37 (d, *J* = 7.3 Hz, 1H), 8.27 (br. s., 1H), 8.18 (s, 1H), 7.67 (t, *J* = 7.8 Hz, 1H), 7.54 (t, *J* = 8.3 Hz, 1H), 7.51 – 7.46 (m, 1H), 7.45 – 7.35 (m, 2H), 7.27 (d, *J* = 8.3 Hz, 1H), 7.16 (d, *J* = 7.3 Hz, 1H), 7.10 (t, *J* = 8.8 Hz, 1H), 4.07 (q, *J* = 6.9 Hz, 2H), 3.82 (s, 3H), 2.79 – 2.72 (m, 6H), 1.33 (t, *J* = 6.9 Hz, 3H). ^{13}C NMR (126 MHz, DMSO-d$_6$) δ 151.8 (d, *J* = 245 Hz) 151.3, 145.1 (d, *J* = 11 Hz), 136.3, 135.6 (d, *J* = 2 Hz), 134.3 (d, *J* = 7 Hz), 132.3, 129.7, 129.6, 129.3, 129.2, 128.9, 127.5, 127.1, 123.6, 122.6 (d, *J* = 3 Hz), 121.3, 119.3, 118.7, 115.07, 115.06, 114.8, 114.0 (d, *J* = 19 Hz), 110.9, 109.5, 64.3, 45.0, 33.3, 14.6. HRMS (ESI) calcd for $C_{30}H_{28}FN_3O_4S$ [M+H]$^+$: 546.1858, found: 546.1870

4-((3,4-bis(2,4-difluorophenyl)-2,5-dioxo-2,5-dihydro-1H-pyrrol-1-yl)methyl)-N-((2-(6,7-dimethoxy-1-phenyl-3,4-dihydroisoquinolin-2(1H)-yl)ethyl)sulfonyl)benzamide, SZ27TA42

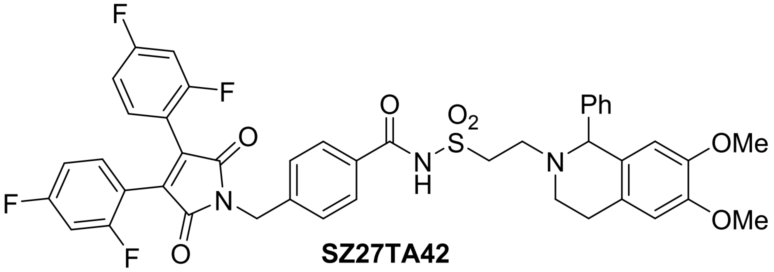

SZ27TA42

^{1}H NMR (500 MHz, DMSO-d$_6$) δ 7.86 (d, *J* = 8.3 Hz, 2H), 7.58 (dt, *J* = 6.6, 8.4 Hz, 2H), 7.42 (d, *J* = 8.3 Hz, 2H), 7.38 – 7.32 (m, 2H), 7.27 – 7.15 (m, 7H), 6.72 (s, 1H), 6.17 (s, 1H), 4.87 (s, 1H), 4.83 (s, 2H), 3.83 – 3.74 (m, 1H), 3.71 (s, 3H), 3.62 – 3.51 (m, 1H), 3.45 (s, 3H), 3.12 – 3.03 (m, 1H), 3.01 – 2.86 (m, 3H), 2.85 – 2.74 (m, 2H). ^{13}C NMR (126 MHz, DMSO-d$_6$) δ 168.8, 167.5, 163.4 (dd, *J* = 251, 13 Hz), 159.9 (dd, *J* = 255, 13 Hz), 147.6, 146.9, 141.6, 140.5, 133.5, 133.0 (dd, *J* = 14, 4 Hz), 129.4, 128.7, 128.2, 127.7, 127.5, 127.2, 127.1, 125.8, 113.4 (dd, *J* = 15, 6 Hz), 112.1 (dd, *J* = 21, 4 Hz), 111.7, 111.4, 104.7 (t, *J* = 25 Hz), 65.4, 55.39, 55.36, 48.4, 48.0, 45.7, 41.4, 26.6. HRMS (ESI) calcd for $C_{43}H_{35}F_4N_3O_7S$ [M+H]$^+$: 814.2205, found: 814.2180

N-((2-((benzo[d][1,3]dioxol-5-ylmethyl)(2-(phenylthio)ethyl)amino)ethyl)sulfonyl)-1-(2,4-bis(trifluoromethyl)benzyl)-1H-indole-5-carboxamide, SZ28TA30

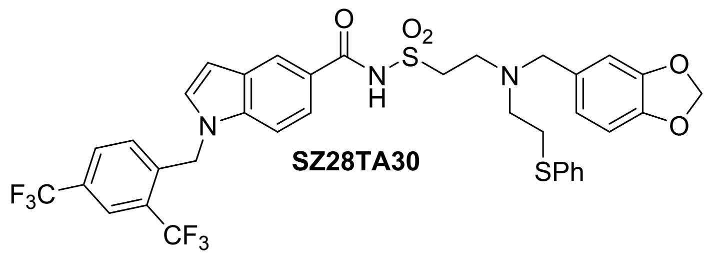

SZ28TA30

^{1}H NMR (500 MHz, DMSO-d$_6$) δ 8.34 (s, 1H), 8.12 (s, 1H), 7.89 (d, J = 8.8 Hz, 1H), 7.72 (d, J = 8.8 Hz, 1H), 7.64 (d, J = 3.4 Hz, 1H), 7.36 (d, J = 8.8 Hz, 1H), 7.23 – 7.16 (m, 4H), 7.13 – 7.07 (m, 1H), 6.88 (s, 1H), 6.78 (d, J = 3.4 Hz, 1H), 6.74 – 6.65 (m, 2H), 6.62 (d, J = 8.3 Hz, 1H), 5.92 (s, 2H), 5.81 (s, 2H), 3.75 – 3.69 (m, 2H), 3.55 (s, 2H), 3.06 – 3.00 (m, 2H), 2.96 (t, J = 6.9 Hz, 2H), 2.67 (t, J = 7.1 Hz, 2H). ^{13}C NMR (126 MHz, DMSO-d$_6$) δ 167.25, 147.21, 146.22, 141.33, 138.27, 136.16, 132.07, 131.42, 130.05 (d, J = 3 Hz), 128.88, 128.65 (d, J = 32 Hz), 128.42, 127.78, 127.65, 126.97 (d, J = 32 Hz), 125.36, 123.65 (d, J = 273 Hz), 123.57, 123.51 (d, J = 273 Hz), 123.22, 122.75, 122.21, 121.88, 109.74, 109.03, 107.70, 103.67, 100.74, 56.56, 51.50, 48.99, 46.71, 45.95 (d, J = 4 Hz), 29.62. HRMS (ESI) calcd for $C_{36}H_{31}F_6N_3O_5S_2$ [M+H]$^+$: 764.1682, found: 764.1733

N-((2-((benzo[d][1,3]dioxol-5-ylmethyl)(2-(phenylthio)ethyl)amino)ethyl)sulfonyl)-4-(di(1H-indol-3-yl)methyl)benzamide, SZ28TA31

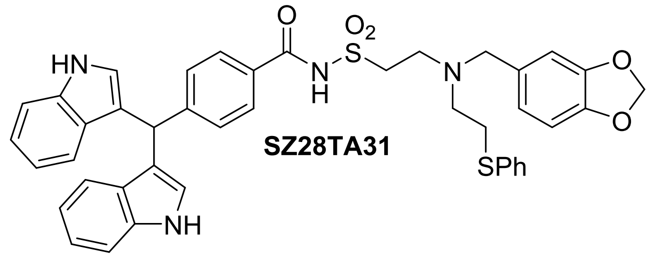

SZ28TA31

^{1}H NMR (500 MHz, DMSO-d$_6$) δ 10.88 (d, J = 2.5 Hz, 2H), 7.84 (d, J = 8.3 Hz, 2H), 7.44 (d, J = 8.3 Hz, 2H), 7.37 (d, J = 8.3 Hz, 2H), 7.29 (d, J = 7.8 Hz, 2H), 7.22 – 7.19 (m, 4H), 7.12 – 7.07 (m, 1H), 7.07 – 7.02 (m, 2H), 6.90 – 6.84 (m, 5H), 6.71 (s, 2H), 5.92 (s, 1H), 5.91 (s, 2H), 3.64 – 3.57 (m, 2H), 3.55 (s, 2H), 3.08 – 3.01 (m, 2H), 2.96 – 2.89 (m, 2H), 2.71 – 2.64 (m, 2H). ^{13}C NMR (126 MHz, DMSO-d$_6$) δ 147.2, 146.2, 136.6, 136.1, 132.0, 129.3, 128.9, 128.7, 128.4, 128.1, 127.7, 126.5, 125.4, 123.7, 121.9, 121.0, 119.0, 118.3, 117.4, 111.5, 109.0, 107.8, 100.7, 56.6, 51.5, 48.7, 46.8, 39.6, 29.6. HRMS (ESI) calcd for $C_{42}H_{38}N_4O_5S_2$ [M+Na]$^+$: 765.2176, found: 765.2180

N-((2-((benzo[d][1,3]dioxol-5-ylmethyl)(2-(phenylthio)ethyl)amino)ethyl)sulfonyl)-1-methyl-3-(thiophene-2-carbonyl)-1H-indole-5-carboxamide, SZ28TA45

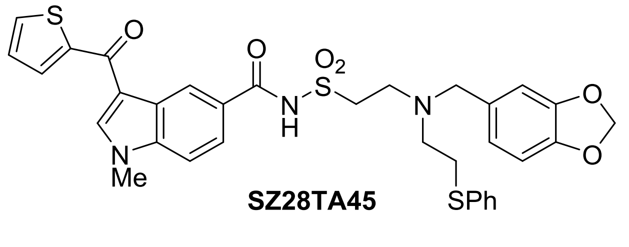

SZ28TA45

^{1}H NMR (500 MHz, DMSO-d$_6$) δ 8.92 (s, 1H), 8.52 (s, 1H), 8.04 – 7.95 (m, 2H), 7.91 (d, J = 8.8 Hz, 1H), 7.69 – 7.58 (m, 1H), 7.29 (br. s., 1H), 7.25 – 7.16 (m, 4H), 7.14 – 7.05 (m, 1H), 6.87 (s, 1H), 6.79 – 6.63 (m, 2H), 5.93 (s, 2H), 3.95 (s, 3H), 3.67 (br. s., 2H), 3.57 (br. s., 2H), 3.04 (t, J = 7.1 Hz, 2H), 2.99 (d, J = 6.4 Hz, 2H), 2.68 (t, J = 6.6 Hz, 2H). ^{13}C NMR (101 MHz, DMSO-d$_6$) δ 180.1, 147.2, 146.2, 144.9, 139.3, 139.2, 136.1, 132.8, 132.0, 131.6, 128.9, 128.4, 127.7, 127.0, 126.1, 125.4, 123.6, 123.2, 121.9, 114.2, 110.4, 109.5, 109.0, 107.7, 100.7, 56.6, 51.4, 48.8, 46.8, 33.4, 29.6. HRMS (ESI) calcd for $C_{33}H_{31}N_3O_6S_3$ [M+Na]$^+$: 684.1267, found: 684.1251

N-((4-(((benzo[d][1,3]dioxol-5-ylmethyl)(2-(phenylthio)ethyl)amino)methyl)phenyl)sulfonyl)-4-methyl-2-phenylthiazole-5-carboxamide, SZ31TA3

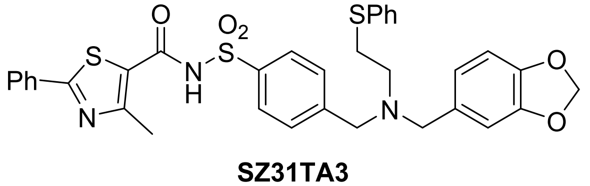

SZ31TA3

Yield = 60%. R$_f$ = 0.4 in DCM : MeOH = 20:1. ^{1}H NMR (600 MHz, DMSO) δ 7.88 – 7.84 (m, 2H), 7.77 – 7.72 (m, 2H), 7.44 – 7.41 (m, 3H), 7.35 (d, J = 7.9 Hz, 2H), 7.16 (t, J = 7.5 Hz, 2H), 7.10 (d, J = 7.8 Hz, 2H), 7.02 (t, J = 7.1 Hz, 1H), 6.90 (s, 1H), 6.80 – 6.75 (m, 2H), 5.94 (s, 2H), 3.57 (s, 2H), 3.48 (s, 2H), 3.10 – 3.04 (m, 2H), 2.58 – 2.54 (m, 2H), 2.53 (s, 3H). ^{13}C NMR (101 MHz, $CDCl_3$) δ 170.0, 161.5, 159.8, 149.6, 148.8, 140.8, 134.8, 132.6, 132.1, 131.8, 131.6, 131.5, 129.6, 129.6, 129.4, 128.2, 127.2, 125.6, 122.5, 121.2, 110.9, 109.1, 102.0, 58.1, 56.8, 50.8, 28.5, 17.9. HRMS (ESI) calcd for $C_{34}H_{31}N_3O_5S_3$ [M+Na]$^+$: 680.1318, found: 680.1344.

N-((4-(((benzo[d][1,3]dioxol-5-ylmethyl)(2-(phenylthio)ethyl)amino)methyl)phenyl)sulfonyl)-4-(trifluoromethoxy)benzamide, SZ31TA8

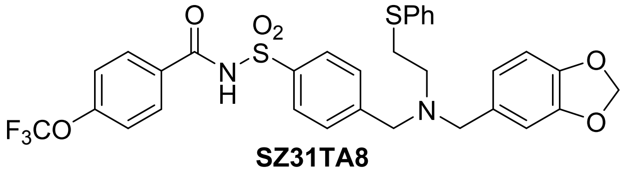

SZ31TA8

Yield = 62%. R$_f$ = 0.57 in DCM : MeOH = 20:1. ^{1}H NMR (400 MHz, DMSO) δ 7.99 – 7.90 (m, 3H), 7.75 (d, J = 8.2 Hz, 2H), 7.34 (d, J = 8.2 Hz, 2H), 7.24 (d, J = 8.1 Hz, 2H), 7.18 – 7.08 (m, 4H), 7.00 (t, J = 7.1 Hz, 1H), 6.81 – 6.74 (m, 2H), 5.95 (s, 2H), 3.57 (s, 2H), 3.48 (s, 2H), 3.11 – 3.04 (m, 2H), 2.59 – 2.52 (m, 2H). ^{13}C NMR (101 MHz, $CDCl_3$) δ 164.4, 153.1, 149.6, 148.8, 140.6, 134.9, 132.6, 131.6, 131.5, 130.6, 129.6, 129.6, 128.1, 125.6, 121.2, 120.6, 120.4 (q, J = 259.6 Hz), 110.8, 109.1, 102.0, 58.1, 56.9, 50.8, 28.5. HRMS (ESI) calcd for $C_{31}H_{27}F_3N_2O_6S_2$ [M+H]$^+$: 645.1335, found: 645.1352.

N-((4-(((benzo[d][1,3]dioxol-5-ylmethyl)(2-(phenylthio)ethyl)amino)methyl)phenyl)sulfonyl)-4'-fluoro-[1,1'-biphenyl]-4-carboxamide, SZ31TA14

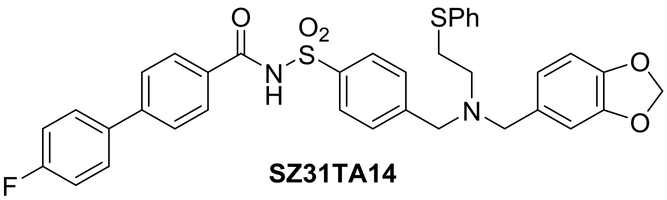

SZ31TA14

^{1}H NMR (500 MHz, DMSO-d_6) δ 7.98 (d, J = 8.3 Hz, 2H), 7.90 – 7.85 (m, 2H), 7.78 – 7.71 (m, 2H), 7.69 – 7.65 (m, 2H), 7.50 – 7.45 (m, 2H), 7.32 – 7.26 (m, 2H), 7.21 – 7.16 (m, 2H), 7.16 – 7.12 (m, 2H), 7.07 – 7.02 (m, 1H), 6.94 (d, J = 1.0 Hz, 1H), 6.84 – 6.78 (m, 2H), 5.98 (s, 2H), 3.63 (s, 2H), 3.52 (s, 2H), 3.15 – 3.09 (m, 2H), 2.62 – 2.57 (m, 2H). ^{13}C NMR (126 MHz, DMSO-d_6) δ 163.1, 161.2, 147.3, 146.2, 141.6, 136.1, 135.8, 132.7, 129.1, 128.9, 128.9, 128.8, 128.1, 127.5, 127.2, 126.1, 125.3, 121.7, 115.9, 115.7, 108.8, 107.9, 100.8, 57.1, 56.8, 51.5, 29.2. HRMS (ESI) calcd for $C_{36}H_{31}FN_2O_5S_2$ $[M+H]^+$: 655.1731, found: 655.1760

N-((4-(((benzo[d][1,3]dioxol-5-ylmethyl)(2-(phenylthio)ethyl)amino)methyl)phenyl)sulfonyl)-2-(9H-carbazol-9-yl)acetamide, SZ31TA15

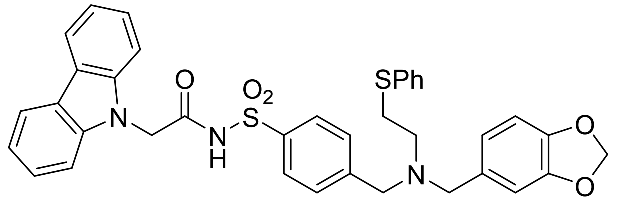

SZ31TA15

^{1}H NMR (500 MHz, DMSO-d_6) δ 8.09 (d, J = 7.8 Hz, 2H), 7.88 (d, J = 8.8 Hz, 2H), 7.56 (d, J = 8.3 Hz, 2H), 7.33 – 7.29 (m, 4H), 7.23 – 7.07 (m, 7H), 6.94 (d, J = 1.5 Hz, 1H), 6.86 – 6.82 (m, 1H), 6.81 – 6.77 (m, 1H), 6.00 (s, 2H), 5.18 (s, 2H), 3.66 (br. s., 2H), 3.50 (br. s., 2H), 3.15 – 3.10 (m, 2H), 2.59 (t, J = 7.1 Hz, 2H). ^{13}C NMR (126 MHz, DMSO-d_6) δ 167.4, 147.3, 146.3, 140.3, 138.0, 136.0, 132.5, 128.9, 128.8, 127.6, 127.4, 127.4, 125.6, 125.4, 122.2, 121.8, 120.1, 119.1, 109.0, 108.9, 107.9, 100.8, 57.1, 56.6, 51.5, 45.5, 29.2. HRMS (ESI) calcd for $C_{37}H_{33}N_3O_5S_2$ $[M+H]^+$: 664.1935, found: 664.1952

N-((4-(((benzo[d][1,3]dioxol-5-ylmethyl)(2-(phenylthio)ethyl)amino)methyl)phenyl)sulfonyl)-4-(2-(pyridin-2-yl)ethyl)benzamide, SZ31TA17

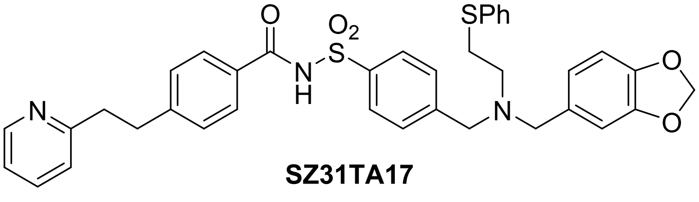

SZ31TA17

^{1}H NMR (500 MHz, DMSO-d_6) δ 8.50 – 8.46 (m, 1H), 7.88 (d, J = 8.3 Hz, 2H), 7.81 (d, J = 8.3 Hz, 2H), 7.63 (dt, J = 2.0, 7.8 Hz, 1H), 7.48 (d, J = 8.8 Hz, 2H), 7.22 (d, J = 8.3 Hz, 2H), 7.20 – 7.12 (m, 6H), 7.05 – 7.00 (m, 1H), 6.94 (d, J = 1.0 Hz, 1H), 6.83 – 6.78 (m, 2H), 5.98 (s, 2H), 3.63 (s, 2H), 3.51 (s, 2H), 3.14 – 3.08 (m, 2H), 3.02 (s, 4H), 2.62 – 2.56 (m, 2H). ^{13}C NMR (126 MHz, DMSO-d_6) δ 167.7, 162.3, 160.4, 148.9, 147.3, 146.2, 145.3, 143.3, 141.7, 136.3, 136.1, 132.7,

128.9, 128.5, 128.1, 127.9, 127.5, 127.2, 125.3, 122.9, 121.7, 121.3, 108.8, 107.8, 100.8, 57.1, 56.7, 54.9, 51.5, 34.8, 29.2. HRMS (ESI) calcd for $C_{37}H_{35}N_3O_5S_2$ $[M+H]^+$: 666.2091, found: 666.2122

N-((4-(((benzo[d][1,3]dioxol-5-ylmethyl)(2-(phenylthio)ethyl)amino)methyl)phenyl)sulfonyl)-4-(2,3,4-trimethoxybenzyl)benzamide, SZ31TA24

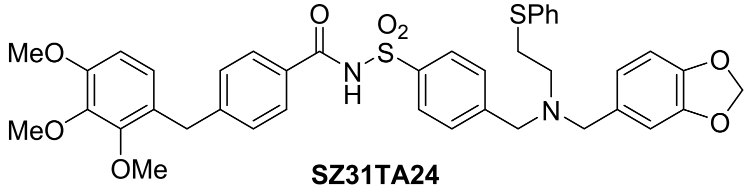

SZ31TA24

^{1}H NMR (400 MHz, DMSO-d6) δ 7.89 (d, *J* = 8.2 Hz, 2H), 7.81 (d, *J* = 8.2 Hz, 2H), 7.51 (d, *J* = 8.2 Hz, 2H), 7.24 – 7.18 (m, 2H), 7.17 – 7.11 (m, 4H), 7.04 – 6.98 (m, 1H), 6.94 (s, 1H), 6.87 – 6.77 (m, 3H), 6.71 (d, *J* = 8.6 Hz, 1H), 5.97 (s, 2H), 3.87 (s, 2H), 3.75 (s, 3H), 3.71 (s, 3H), 3.64 (s, 2H), 3.61 (s, 3H), 3.52 (s, 2H), 3.11 (t, *J* = 7.4 Hz, 2H), 2.59 (t, *J* = 7.2 Hz, 2H). ^{13}C NMR (126 MHz, DMSO-d6) δ 166.9, 152.2, 151.2, 147.3, 146.2, 145.8, 144.0, 141.9, 140.6, 136.0, 132.7, 128.8, 128.5, 128.4, 128.2, 127.5, 127.4, 127.1, 126.0, 125.3, 124.4, 121.7, 108.8, 107.8, 107.7, 100.8, 60.4, 60.2, 57.1, 56.7, 55.8, 51.5, 35.2, 29.2. HRMS (ESI) calcd for $C_{40}H_{40}N_2O_8S_2$ $[M+H]^+$: 741.2299, found: 741.2310

N-((4-(((benzo[d][1,3]dioxol-5-ylmethyl)(2-(phenylthio)ethyl)amino)methyl)phenyl)sulfonyl)-1-(2,4-bis(trifluoromethyl)benzyl)-1H-indole-5-carboxamide, SZ31TA30

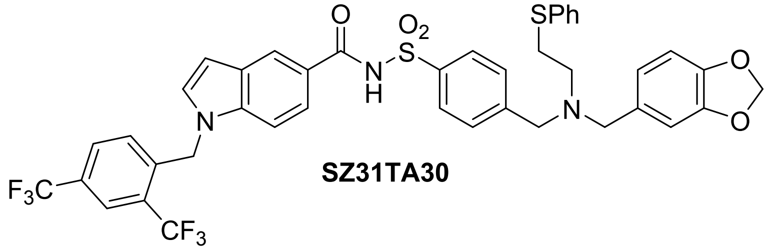

SZ31TA30

^{1}H NMR (500 MHz, DMSO-d6) δ 12.30 (br. s., 1H), 8.30 (s, 1H), 8.10 (s, 1H), 7.93 (d, *J* = 8.3 Hz, 2H), 7.90 (d, *J* = 8.3 Hz, 1H), 7.66 (d, *J* = 8.8 Hz, 1H), 7.60 (d, *J* = 3.4 Hz, 1H), 7.58 – 7.53 (m, 2H), 7.30 (d, *J* = 8.8 Hz, 1H), 7.20 – 7.11 (m, 4H), 7.05 – 6.99 (m, 1H), 6.94 (s, 1H), 6.84 – 6.78 (m, 2H), 6.74 (d, *J* = 2.9 Hz, 1H), 6.57 (d, *J* = 8.3 Hz, 1H), 5.97 (s, 2H), 5.78 (br. s., 2H), 3.68 – 3.63 (m, 2H), 3.52 (s, 2H), 3.16 – 3.08 (m, 2H), 2.62 – 2.56 (m, 2H). ^{13}C NMR (126 MHz, DMSO-d6) δ 147.3, 146.2, 141.4, 138.0, 136.0, 132.7, 131.2, 130.1, 128.8, 128.5, 128.4, 127.7, 127.5, 127.5, 127.0, 126.8, 126.5, 125.6, 125.3, 124.6, 124.3, 123.2, 122.5, 122.4, 122.2, 121.7, 119.7, 109.5, 108.8, 107.8, 103.5, 100.8, 57.1, 56.7, 51.5, 45.9, 29.2. HRMS (ESI) calcd for $C_{41}H_{33}F_6N_3O_5S_2$ $[M+H]^+$: 826.1839, found: 826.1868

N-((4-(((benzo[d][1,3]dioxol-5-ylmethyl)(2-(phenylthio)ethyl)amino)methyl)phenyl)sulfonyl)-4-(di(1H-indol-3-yl)methyl)benzamide, SZ31TA31

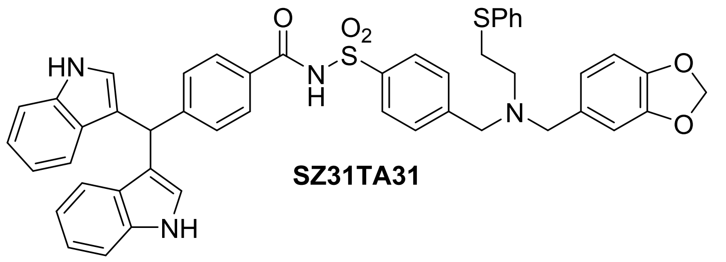

SZ31TA31

^{1}H NMR (500 MHz, DMSO-d$_6$) δ 10.89 (d, J = 2.0 Hz, 2H), 7.94 (d, J = 8.3 Hz, 2H), 7.84 (d, J = 8.3 Hz, 2H), 7.60 – 7.54 (m, 2H), 7.48 – 7.42 (m, 2H), 7.37 (d, J = 7.8 Hz, 2H), 7.28 (d, J = 7.8 Hz, 2H), 7.16 – 7.12 (m, 4H), 7.05 (t, J = 7.6 Hz, 2H), 7.01 – 6.97 (m, 1H), 6.96 (s, 1H), 6.90 – 6.84 (m, 4H), 6.84 – 6.78 (m, 2H), 5.98 (s, 2H), 5.92 (s, 1H), 3.65 (s, 2H), 3.53 (s, 2H), 3.15 – 3.09 (m, 2H), 2.63 – 2.57 (m, 2H). ^{13}C NMR (126 MHz, DMSO-d$_6$) δ 206.5, 166.1, 162.3, 150.1, 147.3, 146.2, 136.6, 136.0, 132.6, 128.8, 128.5, 128.4, 128.3, 127.6, 127.5, 126.5, 125.3, 123.7, 121.8, 121.0, 119.0, 118.3, 117.3, 111.5, 108.9, 107.9, 100.8, 57.2, 56.7, 51.6, 39.6, 29.2. HRMS (ESI) calcd for $C_{47}H_{40}N_4O_5S_2$ $[M+Na]^+$: 827.2332, found: 827.2340

N-((4-(((benzo[d][1,3]dioxol-5-ylmethyl)(2-(phenylthio)ethyl)amino)methyl)phenyl)sulfonyl)-3-(cyclohexanecarbonyl)-1-methyl-1H-indole-5-carboxamide, SZ31TA40

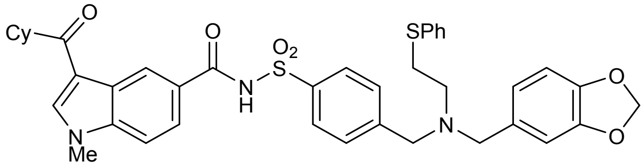

SZ31TA40

^{1}H NMR (500 MHz, DMSO-d$_6$) δ 8.85 (s, 1H), 8.48 – 8.41 (m, 1H), 7.91 (d, J = 8.3 Hz, 2H), 7.81 (d, J = 8.8 Hz, 1H), 7.51 (d, J = 7.8 Hz, 3H), 7.20 – 7.11 (m, 4H), 7.06 – 7.00 (m, 1H), 6.94 (s, 1H), 6.84 – 6.77 (m, 2H), 5.97 (s, 2H), 3.86 (s, 3H), 3.63 (s, 2H), 3.51 (s, 2H), 3.16 – 3.06 (m, 3H), 2.63 – 2.54 (m, 2H), 1.83 – 1.73 (m, 4H), 1.69 (d, J = 13.2 Hz, 1H), 1.51 – 1.33 (m, 5H). ^{13}C NMR (126 MHz, DMSO-d$_6$) δ 198.3, 166.9, 162.3, 147.3, 146.2, 139.3, 138.5, 136.0, 132.7, 128.8, 128.2, 127.5, 127.3, 125.4, 125.3, 123.4, 123.2, 121.7, 114.7, 109.9, 108.8, 107.8, 100.8, 57.1, 56.7, 51.5, 46.4, 35.8, 33.2, 29.6, 29.2, 25.6, 25.4. HRMS (ESI) calcd for $C_{40}H_{41}N_3O_6S_2$ $[M+H]^+$: 724.2510, found: 724.2506

N-((4-(((benzo[d][1,3]dioxol-5-ylmethyl)(2-(phenylthio)ethyl)amino)methyl)phenyl)sulfonyl)-3-(phenethylamino)benzamide, SZ31TA44

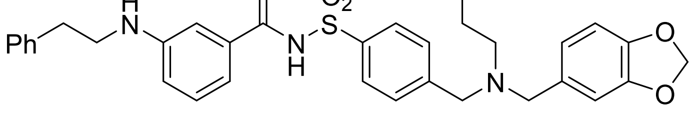

SZ31TA44

^{1}H NMR (500 MHz, DMSO-d$_6$) δ 12.29 (br. s., 1H), 7.93 (d, J = 8.3 Hz, 2H), 7.60 (d, J = 7.8 Hz, 2H), 7.30 – 7.24 (m, 4H), 7.22 – 7.17 (m, 2H), 7.17 – 7.12 (m, 5H), 7.06 – 7.00 (m, 3H), 6.96 – 6.92 (m, 1H), 6.85 – 6.77 (m, 3H), 5.98 (s, 2H), 3.67 (br. s., 2H), 3.54 (br. s., 2H), 3.26 (t, J = 7.1

Hz, 2H), 3.16 – 3.08 (m, 2H), 2.86 – 2.80 (m, 2H), 2.63 – 2.56 (m, 2H). ^{13}C NMR (126 MHz, DMSO-d_6) δ 166.1, 148.8, 147.3, 146.2, 139.7, 138.3, 135.9, 132.5, 129.0, 128.9, 128.8, 128.7, 128.7, 128.3, 127.7, 127.5, 126.0, 125.6, 125.3, 121.8, 117.0, 115.6, 110.7, 108.9, 107.9, 100.8, 57.1, 56.7, 51.5, 44.5, 34.7, 29.1. HRMS (ESI) calcd for $C_{38}H_{37}N_3O_5S_2$ [M+H]$^+$: 680.2248, found: 680.2256

N-((4-(((benzo[d][1,3]dioxol-5-ylmethyl)(2-(phenylthio)ethyl)amino)methyl)phenyl)sulfonyl)-1-methyl-3-(thiophene-2-carbonyl)-1H-indole-5-carboxamide, SZ31TA45

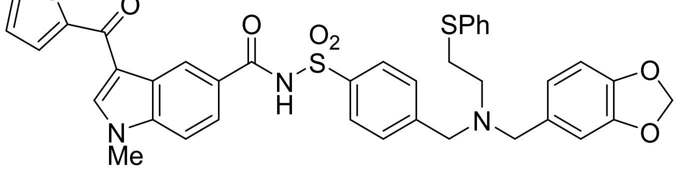

SZ31TA45

^{1}H NMR (500 MHz, DMSO-d_6) δ 8.85 (d, *J* = 1.5 Hz, 1H), 8.54 (s, 1H), 8.01 – 7.99 (m, 1H), 7.97 (d, *J* = 8.3 Hz, 2H), 7.81 (dd, *J* = 1.5, 8.8 Hz, 1H), 7.71 (td, *J* = 2.9, 5.5 Hz, 1H), 7.69 – 7.65 (m, 2H), 7.61 (d, *J* = 8.3 Hz, 2H), 7.29 (dd, *J* = 3.9, 4.9 Hz, 1H), 7.19 – 7.12 (m, 4H), 7.06 – 6.99 (m, 1H), 6.95 (s, 1H), 6.83 – 6.80 (m, 1H), 5.97 (s, 2H), 3.94 (s, 3H), 3.68 (br. s., 2H), 3.54 (br. s., 2H), 3.17 – 3.10 (m, 2H), 2.59 (t, *J* = 6.9 Hz, 2H). HRMS (ESI) calcd for $C_{38}H_{33}N_3O_6S_3$ [M+H]$^+$: 724.1604, found: 724.1651

4-((3,4-bis(2,4-difluorophenyl)-2,5-dioxo-2,5-dihydro-1H-pyrrol-1-yl)methyl)-N-((4-(3,5-dimethylisoxazol-4-yl)phenyl)sulfonyl)benzamide, SZ32TA42

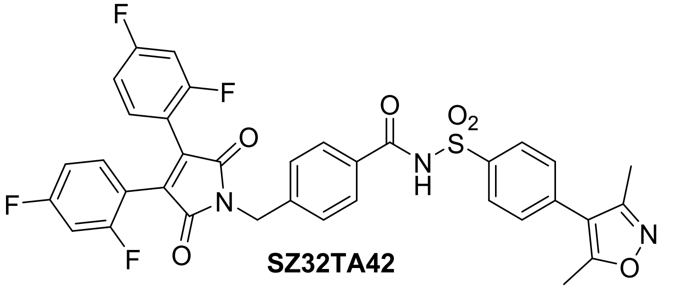

SZ32TA42

^{1}H NMR (500 MHz, DMSO-d_6) δ 7.99 (d, *J* = 8.3 Hz, 2H), 7.90 (d, *J* = 8.3 Hz, 2H), 7.61 – 7.51 (m, 4H), 7.40 (d, *J* = 8.3 Hz, 2H), 7.37 – 7.30 (m, 2H), 7.22 (dt, *J* = 2.5, 8.6 Hz, 2H), 4.80 (s, 2H), 2.43 (s, 3H), 2.25 (s, 3H). ^{13}C NMR (126 MHz, DMSO-d_6) □ 168.7, 167.4, 165.9, 164.4, 162.4, 160.9, 158.8, 158.1, 140.0, 133.5, 133.3, 133.0, 128.8, 128.6, 127.8, 127.2, 115.1, 113.4, 112.1, 104.7, 41.4, 11.5, 10.5. HRMS (ESI) calcd for $C_{35}H_{23}F_4N_3O_6S$ [M+H]$^+$: 690.1317, found: 690.1341

4'-(N-(4-((3,4-bis(2,4-difluorophenyl)-2,5-dioxo-2,5-dihydro-1H-pyrrol-1-yl)methyl)benzoyl)sulfamoyl)-N,N-dimethyl-[1,1'-biphenyl]-3-carboxamide, SZ34TA42

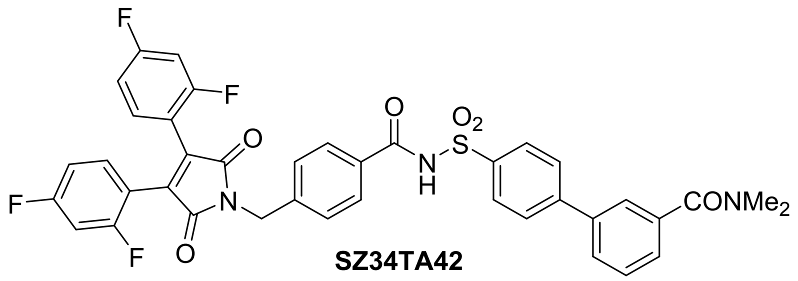

SZ34TA42

^{1}H NMR (400 MHz, DMSO-d_6) δ 7.95 (d, J = 8.2 Hz, 2H), 7.91 (d, J = 8.2 Hz, 2H), 7.77 (d, J = 8.2 Hz, 3H), 7.69 (s, 1H), 7.61 – 7.51 (m, 3H), 7.41 (d, J = 7.4 Hz, 1H), 7.38 – 7.29 (m, 4H), 7.22 (dt, J = 2.3, 8.4 Hz, 2H), 4.78 (s, 2H), 3.00 (br. s., 3H), 2.93 (br. s., 3H). ^{13}C NMR (126 MHz, DMSO-d_6) δ 169.8, 168.7, 168.5, 164.4, 162.4, 160.9, 158.8, 141.5, 139.2, 139.0, 137.3, 133.5, 133.0, 129.1, 128.7, 127.8, 127.7, 126.9, 126.5, 126.4, 125.3, 113.5, 112.1, 104.7, 41.5, 34.8, 30.7. HRMS (ESI) calcd for $C_{39}H_{27}F_4N_3O_6S$ $[M+H]^+$: 742.1630, found: 742.1650

4-methyl-N-((4'-phenoxy-[1,1'-biphenyl]-4-yl)sulfonyl)-2-phenylthiazole-5-carboxamide, SZ35TA3

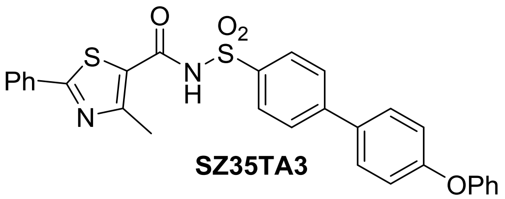

SZ35TA3

^{1}H NMR (500 MHz, DMSO-d_6) δ 7.95 – 7.88 (m, 4H), 7.74 – 7.68 (m, 4H), 7.50 – 7.45 (m, 3H), 7.44 – 7.39 (m, 2H), 7.19 – 7.15 (m, 1H), 7.12 – 7.06 (m, 4H), 2.61 (s, 3H). ^{13}C NMR (126 MHz, DMSO-d_6) δ 165.6, 164.9, 156.8, 156.3, 154.5, 144.5, 141.0, 134.5, 133.3, 132.4, 130.2, 130.2, 129.2, 128.6, 127.6, 126.0, 125.8, 123.8, 119.0, 118.8, 16.9. HRMS (ESI) calcd for $C_{29}H_{22}N_2O_4S_2$ $[M+H]^+$: 527.1094, found: 527.1090

N-((4'-phenoxy-[1,1'-biphenyl]-4-yl)sulfonyl)-1-naphthamide, SZ35TA7

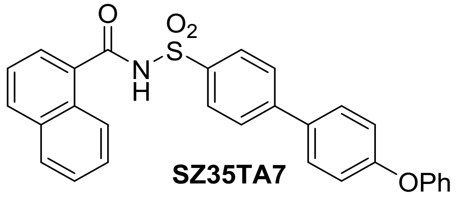

SZ35TA7

^{1}H NMR (500 MHz, DMSO-d_6) δ 13.03 – 12.74 (m, 1H), 8.18 – 8.14 (m, 2H), 8.09 (d, J = 8.3 Hz, 1H), 8.04 – 8.00 (m, 1H), 7.99 – 7.95 (m, 3H), 7.83 – 7.79 (m, 3H), 7.59 – 7.51 (m, 3H), 7.46 – 7.41 (m, 2H), 7.22 – 7.17 (m, 1H), 7.15 – 7.07 (m, 4H). ^{13}C NMR (126 MHz, DMSO-d_6) δ 167.6, 157.6, 156.1, 144.3, 138.3, 133.3, 133.1, 131.8, 131.0, 130.2, 129.5, 128.9, 128.5, 128.4, 127.5, 127.3, 126.9, 126.5, 124.8, 124.4, 124.0, 119.2, 118.8. HRMS (ESI) calcd for C29H21NO4S [M+H]+: 480.1264, found: 480.1272

N-((4'-phenoxy-[1,1'-biphenyl]-4-yl)sulfonyl)-4-(2-(pyridin-2-yl)ethyl)benzamide, SZ35TA17

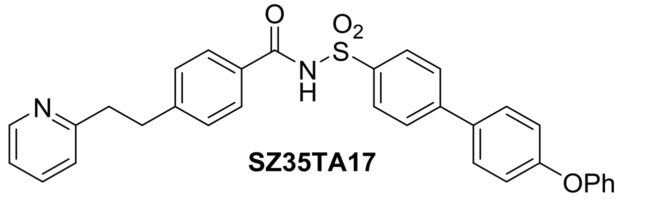

^{1}H NMR (500 MHz, DMSO-d$_6$) δ 8.48 (d, *J* = 4.9 Hz, 1H), 7.98 (d, *J* = 8.3 Hz, 2H), 7.85 (d, *J* = 7.8 Hz, 2H), 7.76 – 7.69 (m, 4H), 7.66 – 7.60 (m, 1H), 7.41 (t, *J* = 8.1 Hz, 2H), 7.23 – 7.14 (m, 5H), 7.11 – 7.05 (m, 4H), 3.01 (s, 4H). ^{13}C NMR (126 MHz, DMSO-d$_6$) δ 168.8, 160.5, 157.0, 156.2, 149.0, 144.7, 142.8, 141.9, 136.4, 134.6, 134.2, 130.2, 128.7, 128.6, 127.8, 127.7, 126.0, 123.8, 122.9, 121.3, 119.1, 118.8, 38.9, 34.9. HRMS (ESI) calcd for $C_{29}H_{21}NO_4S$ [M+H]$^+$: 535.1686, found: 535.1708

N-((4'-phenoxy-[1,1'-biphenyl]-4-yl)sulfonyl)-4-(2,3,4-trimethoxybenzyl)benzamide, SZ35TA24

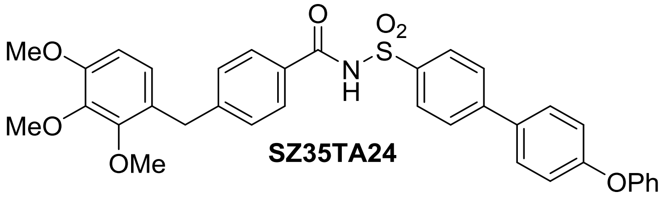

^{1}H NMR (400 MHz, DMSO-d$_6$) δ 8.04 – 8.00 (m, 2H), 7.85 (d, *J* = 8.6 Hz, 2H), 7.80 (d, *J* = 8.6 Hz, 2H), 7.77 – 7.73 (m, 2H), 7.46 – 7.39 (m, 2H), 7.24 (d, *J* = 8.2 Hz, 2H), 7.21 – 7.16 (m, 1H), 7.12 – 7.07 (m, 4H), 6.86 (d, *J* = 8.6 Hz, 1H), 6.72 (d, *J* = 8.6 Hz, 1H), 3.88 (s, 2H), 3.75 (s, 3H), 3.71 (s, 3H), 3.61 (s, 3H). ^{13}C NMR (101 MHz, DMSO-d$_6$) δ 166.0, 157.4, 156.1, 152.3, 151.2, 146.7, 143.7, 141.9, 139.1, 133.5, 130.4, 130.2, 128.8, 128.5, 128.5, 128.2, 126.7, 125.9, 124.5, 123.9, 119.2, 118.7, 107.8, 60.4, 60.2, 55.8, 35.2. HRMS (ESI) calcd for $C_{35}H_{31}NO_7S$ [M+H]$^+$: 610.1894, found: 610.1911

N-((4-(1H-indol-2-yl)phenyl)sulfonyl)-4-(4,4-dimethylpiperidin-1-yl)benzamide, SZ36TA2

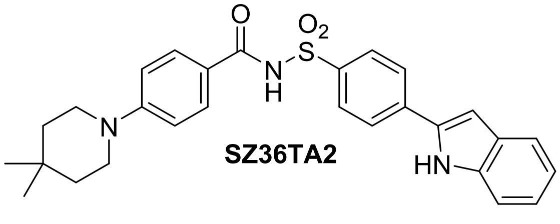

^{1}H NMR (500 MHz, DMSO-d$_6$) δ 12.08 (br. s., 1H), 11.75 (s, 1H), 8.11 – 8.02 (m, 4H), 7.75 (d, *J* = 9.3 Hz, 2H), 7.58 (d, *J* = 7.8 Hz, 1H), 7.44 (d, *J* = 8.3 Hz, 1H), 7.16 (t, *J* = 7.3 Hz, 1H), 7.10 (d, *J* = 1.5 Hz, 1H), 7.03 (t, *J* = 7.3 Hz, 1H), 6.91 (d, *J* = 9.3 Hz, 2H), 3.35 – 3.28 (m, *J* = 11.3 Hz, 4H), 1.38 – 1.32 (m, 4H), 0.92 (s, 6H). ^{13}C NMR (126 MHz, DMSO-d$_6$) δ 164.6, 153.7, 137.9, 137.6, 136.8, 135.7, 130.3, 128.4, 128.4, 125.0, 122.6, 120.6, 119.8, 118.4, 112.7, 111.6, 101.3, 43.2, 37.4, 28.5, 27.6. HRMS (ESI) calcd for $C_{28}H_{29}N_3O_3S$ [M+H]$^+$: 488.2003, found: 488.2021

7. HPLC Traces of Select Hit Compounds

HPLC of SZ4TA30 (purity = 100%)

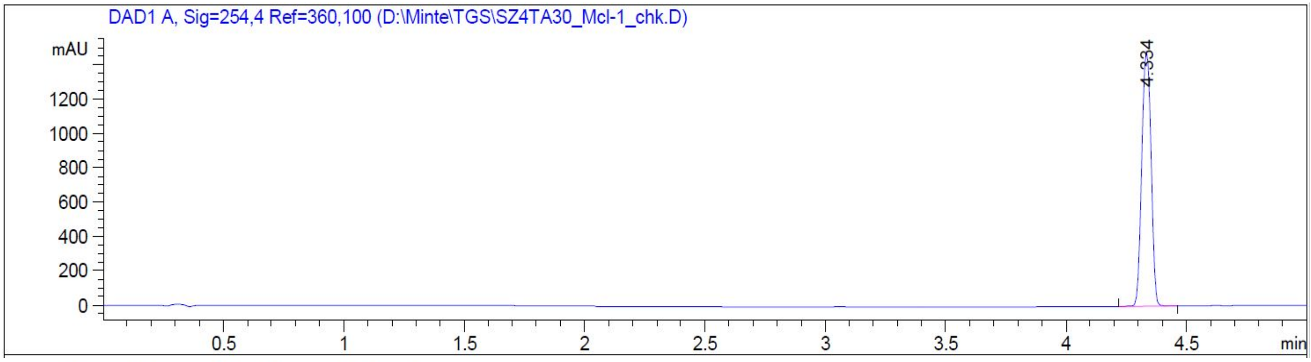

Signal 1: DAD1 A, Sig=254,4 Ref=360,100

Peak #	RetTime [min]	Type	Width [min]	Area [mAU*s]	Height [mAU]	Area %
1	4.334	BB	0.0426	3889.62305	1485.25366	100.0000

Totals : 3889.62305 1485.25366

HPLC of SZ11TA40 (purity = 97.15%)

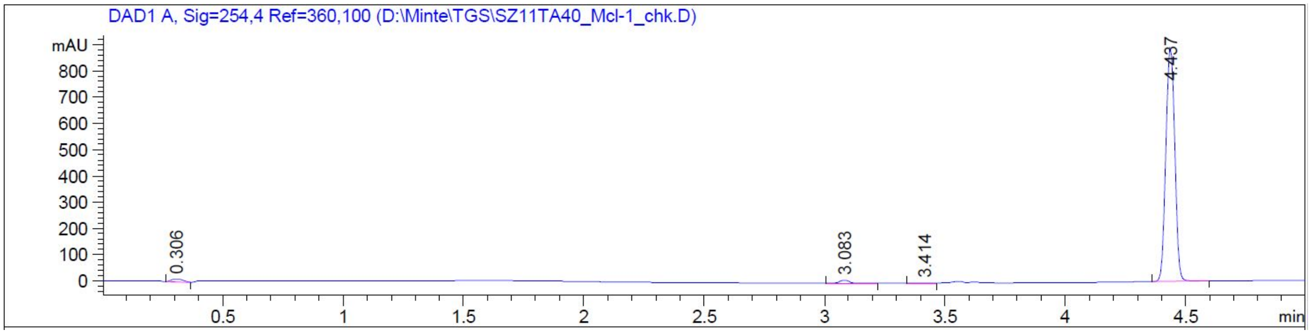

Signal 1: DAD1 A, Sig=254,4 Ref=360,100

Peak #	RetTime [min]	Type	Width [min]	Area [mAU*s]	Height [mAU]	Area %
1	0.306	BB	0.0570	37.28512	11.15277	1.5347
2	3.083	BB	0.0444	28.94370	10.43178	1.1914
3	3.414	BB	0.0624	3.09344	8.50328e-1	0.1273
4	4.437	BB	0.0428	2360.11499	893.04425	97.1466

Totals : 2429.43724 915.47912

HPLC of SZ15TA8 (purity = 100%)

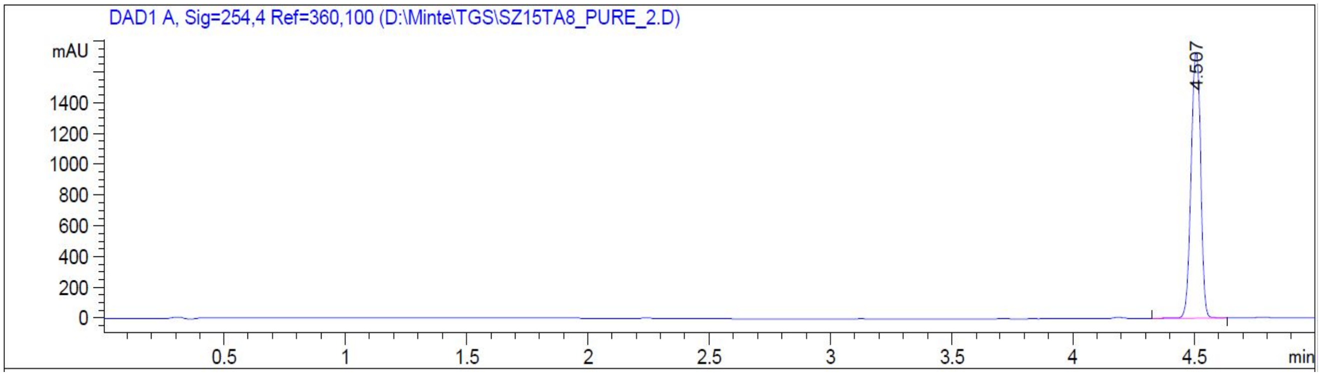

Signal 1: DAD1 A, Sig=254,4 Ref=360,100

Peak #	RetTime [min]	Type	Width [min]	Area [mAU*s]	Height [mAU]	Area %
1	4.507	BB	0.0438	4737.65039	1740.24207	100.0000
Totals :				4737.65039	1740.24207	

HPLC of SZ15TA17 (purity = 98.86%)

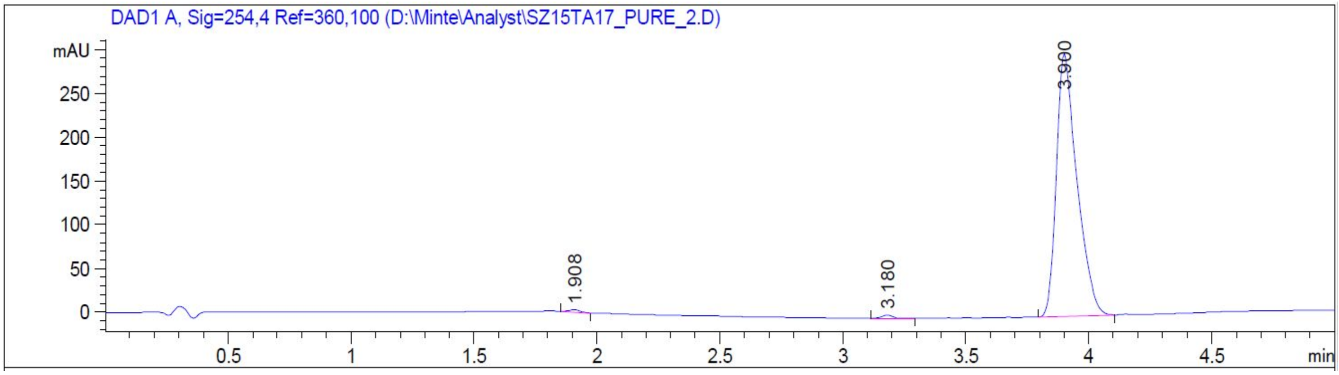

Signal 1: DAD1 A, Sig=254,4 Ref=360,100

Peak #	RetTime [min]	Type	Width [min]	Area [mAU*s]	Height [mAU]	Area %
1	1.908	BB	0.0487	8.95139	2.84800	0.5098
2	3.180	BB	0.0464	11.12632	3.77625	0.6336
3	3.900	BB	0.0834	1735.84851	300.66757	98.8566
Totals :				1755.92622	307.29182	

HPLC of SZ31TA8 (purity = 98.08%)

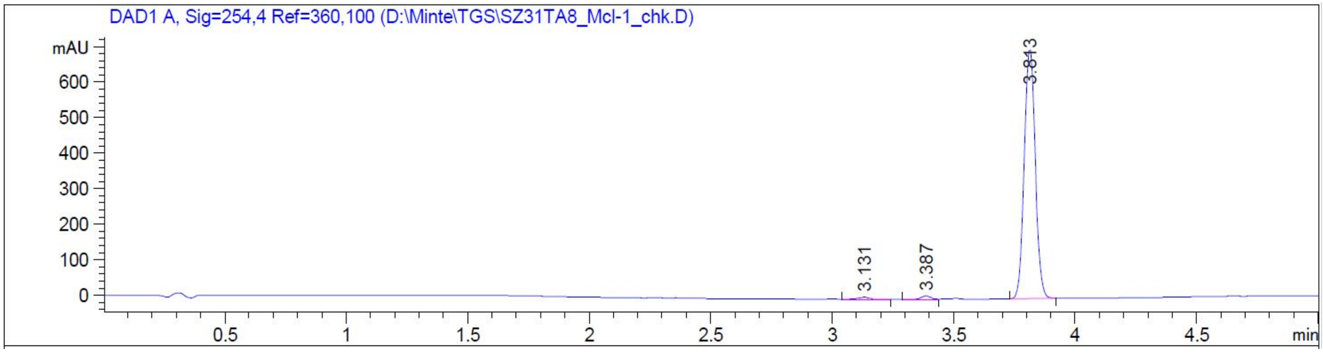

Signal 1: DAD1 A, Sig=254,4 Ref=360,100

Peak #	RetTime [min]	Type	Width [min]	Area [mAU*s]	Height [mAU]	Area %
1	3.131	BB	0.0488	18.58638	5.89744	0.8273
2	3.387	BB	0.0426	24.48523	9.33222	1.0898
3	3.813	BB	0.0484	2203.64038	706.78015	98.0829
Totals :				2246.71199	722.00981	

HPLC of SZ31TA24 (purity = 96.97%)

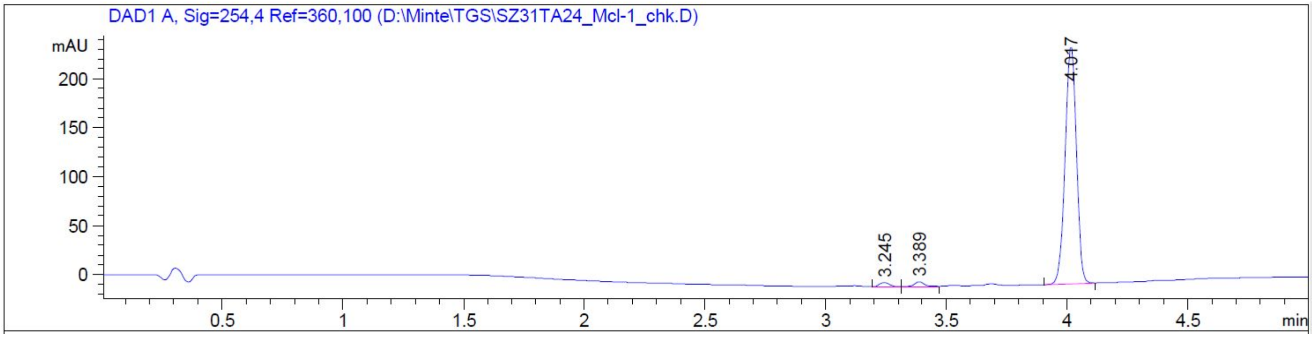

Signal 1: DAD1 A, Sig=254,4 Ref=360,100

Peak #	RetTime [min]	Type	Width [min]	Area [mAU*s]	Height [mAU]	Area %
1	3.245	BB	0.0433	11.11877	4.14772	1.3888
2	3.389	BB	0.0452	13.10836	4.60366	1.6373
3	4.017	BB	0.0492	776.36914	243.27792	96.9739
Totals :				800.59627	252.02931	

HPLC of SZ15TA1 (purity = 100%)

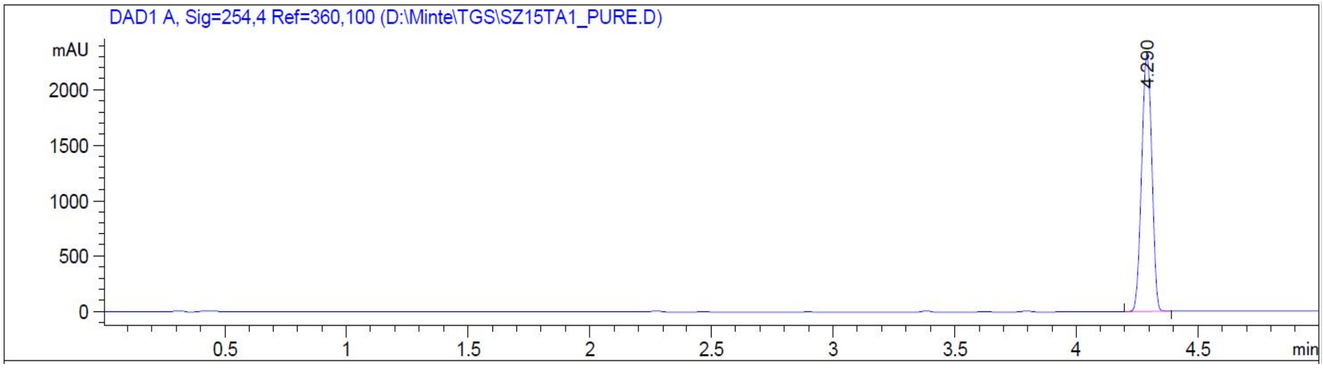

Signal 1: DAD1 A, Sig=254,4 Ref=360,100

Peak #	RetTime [min]	Type	Width [min]	Area [mAU*s]	Height [mAU]	Area %
1	4.290	BB	0.0470	6612.17969	2339.35205	100.0000
Totals :				6612.17969	2339.35205	

HPLC of SZ15TA5 (purity = 100%)

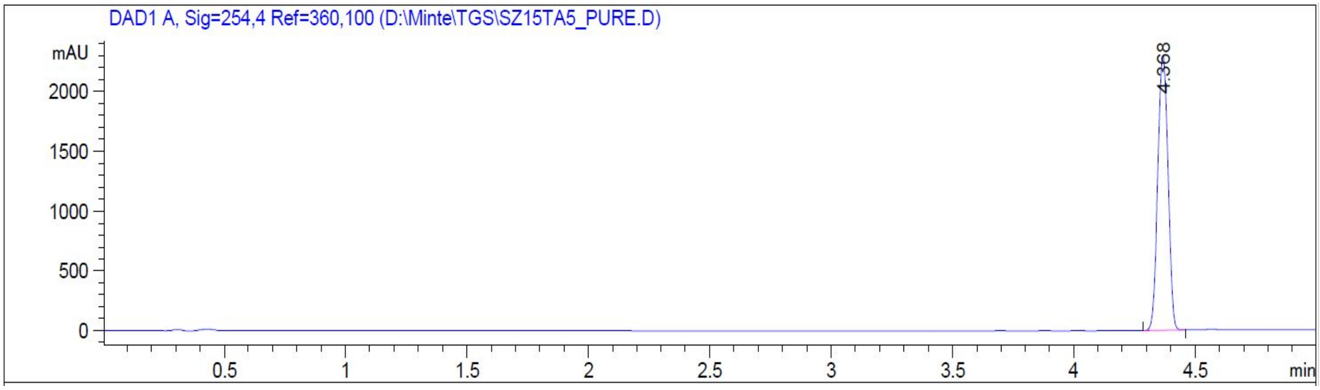

Signal 1: DAD1 A, Sig=254,4 Ref=360,100

Peak #	RetTime [min]	Type	Width [min]	Area [mAU*s]	Height [mAU]	Area %
1	4.368	BB	0.0458	6729.68115	2325.74097	100.0000
Totals :				6729.68115	2325.74097	

HPLC of SZ31TA15 (purity = 95.41%)

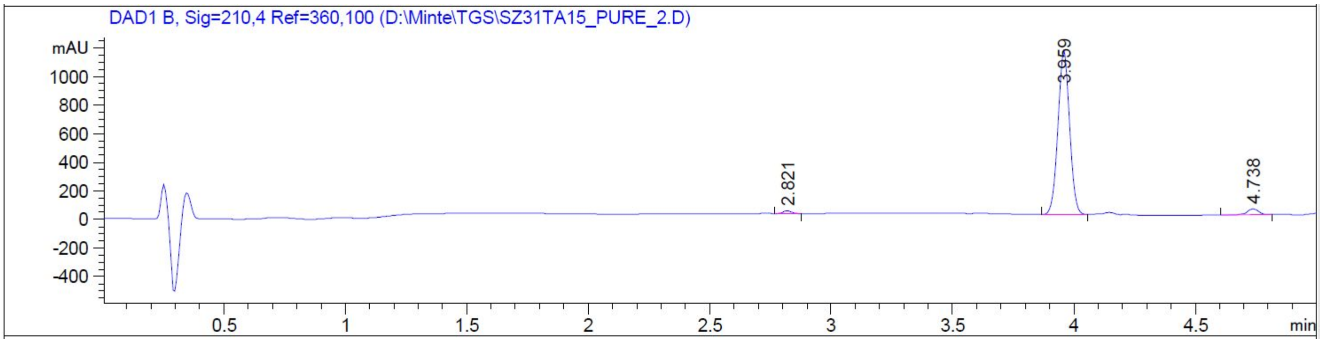

Signal 2: DAD1 B, Sig=210,4 Ref=360,100

Peak #	RetTime [min]	Type	Width [min]	Area [mAU*s]	Height [mAU]	Area %
1	2.821	BB	0.0430	50.17318	18.90651	1.2383
2	3.959	BB	0.0532	3865.84521	1150.39197	95.4074
3	4.738	BB	0.0507	135.91750	40.92565	3.3544

Totals : 4051.93589 1210.22413

References

1. Pelz, N. F.; Bian, Z.; Zhao, B.; Shaw, S.; Tarr, J. C.; Belmar, J.; Gregg, C.; Camper, D. V.; Goodwin, C. M.; Arnold, A. L.; Sensintaffar, J. L.; Friberg, A.; Rossanese, O. W.; Lee, T.; Olejniczak, E. T.; Fesik, S. W., Discovery of 2-Indole-acylsulfonamide Myeloid Cell Leukemia 1 (Mcl-1) Inhibitors Using Fragment-Based Methods. *J. Med. Chem.* **2016,** *59* (5), 2054-66.
2. Hu, X.; Sun, J.; Wang, H. G.; Manetsch, R., Bcl-X_L-templated assembly of its own protein-protein interaction modulator from fragments decorated with thio acids and sulfonyl azides. *J. Am. Chem. Soc.* **2008,** *130* (42), 13820-1.
3. Katritzky, A. R.; Xu, Y.-J.; He, H.-Y.; Mehta, S., Syntheses of 1,4-Benzothiazepines and 1,4-Benzoxazepines via Cyclizations of 1-[2-Arylthio(oxy)ethyl]-5-benzotriazolyl-2-pyrrolidinones and 3-Benzotriazolyl-2-[2-arylthio(oxy)ethyl]-1-isoindolinones. *J. Org. Chem.* **2001,** *66* (16), 5590-5594.
4. Crich, D.; Sana, K.; Guo, S., Amino acid and peptide synthesis and functionalization by the reaction of thioacids with 2,4-dinitrobenzenesulfonamides. *Org. Lett.* **2007,** *9* (22), 4423-6.
5. Paul, R.; Coppola, J. A.; Cohen, E., 1-phenyl-2-phenethyl-1,2,3,4-tetrahydroisoquinolines. A new series of nonsteroidal female antifertility agents. *J. Med. Chem.* **1972,** *15* (7), 720-6.
6. Nacheva, K. P.; Maza, W. A.; Myers, D. Z.; Fronczek, F. R.; Larsen, R. W.; Manetsch, R., Fluorescent properties and resonance energy transfer of 3,4-bis(2,4-difluorophenyl)-maleimide. *Org. Biomol. Chem.* **2012,** *10* (38), 7840-6.
7. Chakrabarty, M.; Basak, R.; Harigaya, Y.; Takayanagi, H., Reaction of 3/2-formylindoles with TOSMIC: formation of indolyloxazoles and stable indolyl primary enamines. *Tetrahedron* **2005,** *61* (7), 1793-1801.
8. Dawson, M. I.; Ye, M.; Cao, X.; Farhana, L.; Hu, Q. Y.; Zhao, Y.; Xu, L. P.; Kiselyuk, A.; Correa, R. G.; Yang, L.; Hou, T.; Reed, J. C.; Itkin-Ansari, P.; Levine, F.; Sanner, M. F.; Fontana, J. A.; Zhang, X. K., Derivation of a retinoid X receptor scaffold from peroxisome proliferator-activated receptor gamma ligand 1-Di(1H-indol-3-yl)methyl-4-trifluoromethylbenzene. *ChemMedChem* **2009,** *4* (7), 1106-19.
9. Guney, T.; Lee, J. J.; Kraus, G. A., First inverse electron-demand Diels-Alder methodology of 3-chloroindoles and methyl coumalate to carbazoles. *Org. Lett.* **2014,** *16* (4), 1124-7.
10. Crich, D.; Sasaki, K., Reaction of thioacids with isocyanates and isothiocyanates: a convenient amide ligation process. *Org. Lett.* **2009,** *11* (15), 3514-7.
11. Namelikonda, N. K.; Manetsch, R., Sulfo-click reaction via in situ generated thioacids and its application in kinetic target-guided synthesis. *Chem. Commun.* **2012,** *48* (10), 1526-8.
12. Kulkarni, S. S.; Hu, X.; Doi, K.; Wang, H. G.; Manetsch, R., Screening of protein-protein interaction modulators via sulfo-click kinetic target-guided synthesis. *ACS Chem. Biol.* **2011,** *6* (7), 724-32.
13. Sato, S.; Sato, T., A mild and environmentally friendly scandium(III) trifluoromethanesulfonate-catalyzed synthesis of bis(3'-indolyl)alkanes and bis(3'-indolyl)-1-deoxyalditols. *Carbohydr. Res.* **2005,** *340* (14), 2251-5.
14. Kaneko, T.; Clark, R. S.; Ohi, N.; Ozaki, F.; Kawahara, T.; Kamada, A.; Okano, K.; Yokohama, H.; Ohkuro, M.; Muramoto, K.; Takenaka, O.; Kobayashi, S., Piperidine

carboxylic acid derivatives of 10H-pyrazino[2,3-b][1,4]benzothiazine as orally-active adhesion molecule inhibitors. *Chem. Pharm. Bull. (Tokyo)* **2004,** *52* (6), 675-87.

15. Quibell, M.; Watts, J. P. Tetrahydrofuro [3,2-b]pyrrol-3-ones as cathepsin K inhibitors and their preparation and use in the treatment of diseases. PCT Int. Appl. WO 2008007114 A1, January 17, 2008.
16. Bommegowda, Y. K.; Lingaraju, G. S.; Thamas, S.; Vinay Kumar, K. S.; Pradeepa Kumara, C. S.; Rangappa, K. S.; Sadashiva, M. P., Weinreb amide as an efficient reagent in the one pot synthesis of benzimidazoles and benzothiazoles. *Tetrahedron Lett.* **2013,** *54* (21), 2693-2695.
17. Christiansen, E.; Due-Hansen, M. E.; Urban, C.; Grundmann, M.; Schmidt, J.; Hansen, S. V.; Hudson, B. D.; Zaibi, M.; Markussen, S. B.; Hagesaether, E.; Milligan, G.; Cawthorne, M. A.; Kostenis, E.; Kassack, M. U.; Ulven, T., Discovery of a potent and selective free fatty acid receptor 1 agonist with low lipophilicity and high oral bioavailability. *J. Med. Chem.* **2013,** *56* (3), 982-92.
18. Chouhan, G.; Alper, H., Domino ring-opening/carboxamidation reactions of N-tosyl aziridines and 2-halophenols/pyridinol: efficient synthesis of 1,4-benzo- and pyrido-oxazepinones. *Org. Lett.* **2010,** *12* (1), 192-5.
19. Christiansen, E.; Due-Hansen, M. E.; Urban, C.; Merten, N.; Pfleiderer, M.; Karlsen, K. K.; Rasmussen, S. S.; Steensgaard, M.; Hamacher, A.; Schmidt, J.; Drewke, C.; Petersen, R. K.; Kristiansen, K.; Ullrich, S.; Kostenis, E.; Kassack, M. U.; Ulven, T., Structure-Activity Study of Dihydrocinnamic Acids and Discovery of the Potent FFA1 (GPR40) Agonist TUG-469. *ACS Med. Chem. Lett.* **2010,** *1* (7), 345-9.
20. Bylund, J.; Ek, M.; Holenz, J.; Johansson, M.H., Kers, A.; Narhi, K. Bis-(Sulfonylamino) Derivatives in Therapy 065. U.S. Patent WO 2009064250, May 22, 2009.
21. Kiani, A.; Akhlaghinia, B.; Rouhi-Saadabad, H.; Bakavoli, M., Direct synthesis of sulfonyl azides from sulfonic acids. *J. Sulfur Chem.* **2013,** *35* (2), 119-127.
22. Ruppel, J. V.; Jones, J. E.; Huff, C. A.; Kamble, R. M.; Chen, Y.; Zhang, X. P., A highly effective cobalt catalyst for olefin aziridination with azides: hydrogen bonding guided catalyst design. *Org. Lett.* **2008,** *10* (10), 1995-8.
23. Stevens, M. Y.; Sawant, R. T.; Odell, L. R., Synthesis of sulfonyl azides via diazotransfer using an imidazole-1-sulfonyl azide salt: scope and ^{15}N NMR labeling experiments. *J. Org. Chem.* **2014,** *79* (11), 4826-31.
